# Divergent total syntheses of ITHQ-type bis-β-carboline alkaloids by regio-selective formal aza-[4 + 2] cycloaddition and late-stage C–H functionalization†

**DOI:** 10.1039/d3sc03722c

**Published:** 2023-09-13

**Authors:** Qixuan Wang, Fusheng Guo, Jin Wang, Xiaoguang Lei

**Affiliations:** a Peking-Tsinghua Center for Life Sciences, Academy for Advanced Interdisciplinary Studies, Peking University Beijing 100871 P. R. China xglei@pku.edu.cn; b Beijing National Laboratory for Molecular Sciences, Key Laboratory of Bioorganic Chemistry and Molecular Engineering of Ministry of Education, Department of Chemical Biology, College of Chemistry and Molecular Engineering, Synthetic and Functional Biomolecules Center, Peking University Beijing 100871 P. R. China; c Institute for Cancer Research, Shenzhen Bay Laboratory Shenzhen 518107 P. R. China

## Abstract

We herein report the first total syntheses of several bis-β-carboline alkaloids, picrasidines G, S, R, and T, and natural product-like derivatives in a divergent manner. Picrasidines G, S, and T feature an indolotetrahydroquinolizinium (ITHQ) skeleton, while picrasidine R possesses a 1,4-diketone linker between two β-carboline fragments. The synthesis of ITHQ-type bis-β-carboline alkaloids could be directly achieved by a late-stage regio-selective aza-[4 + 2] cycloaddition of vinyl β-carboline alkaloids, suggesting that this remarkable aza-[4 + 2] cycloaddition might be involved in the biosynthetic pathway. Computational studies revealed that such aza-[4 + 2] cycloaddition is a stepwise process and explained the unique regioselectivity (ΔΔ*G* = 3.77 kcal mol^−1^). Moreover, the successful application of iridium-catalyzed C–H borylation on β-carboline substrates enabled the site-selective C-8 functionalization for efficient synthesis and structural diversification of this family of natural products. Finally, concise synthesis of picrasidine R by the thiazolium-catalyzed Stetter reaction was also accomplished.

## Abstract 2

The first biomimetic total syntheses of indolotetrahydroquinolizinium-type bis-β-carboline alkaloids were accomplished *via* a unique umpolung type-III aza-[4 + 2] cycloaddition and late-stage C–H functionalization strategy.

## Graphical abstract

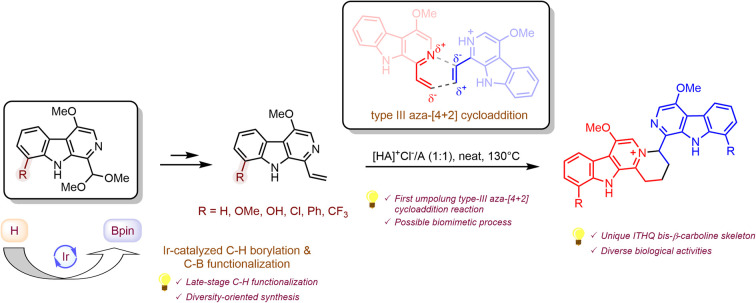

## Introduction

**Scheme 1:**
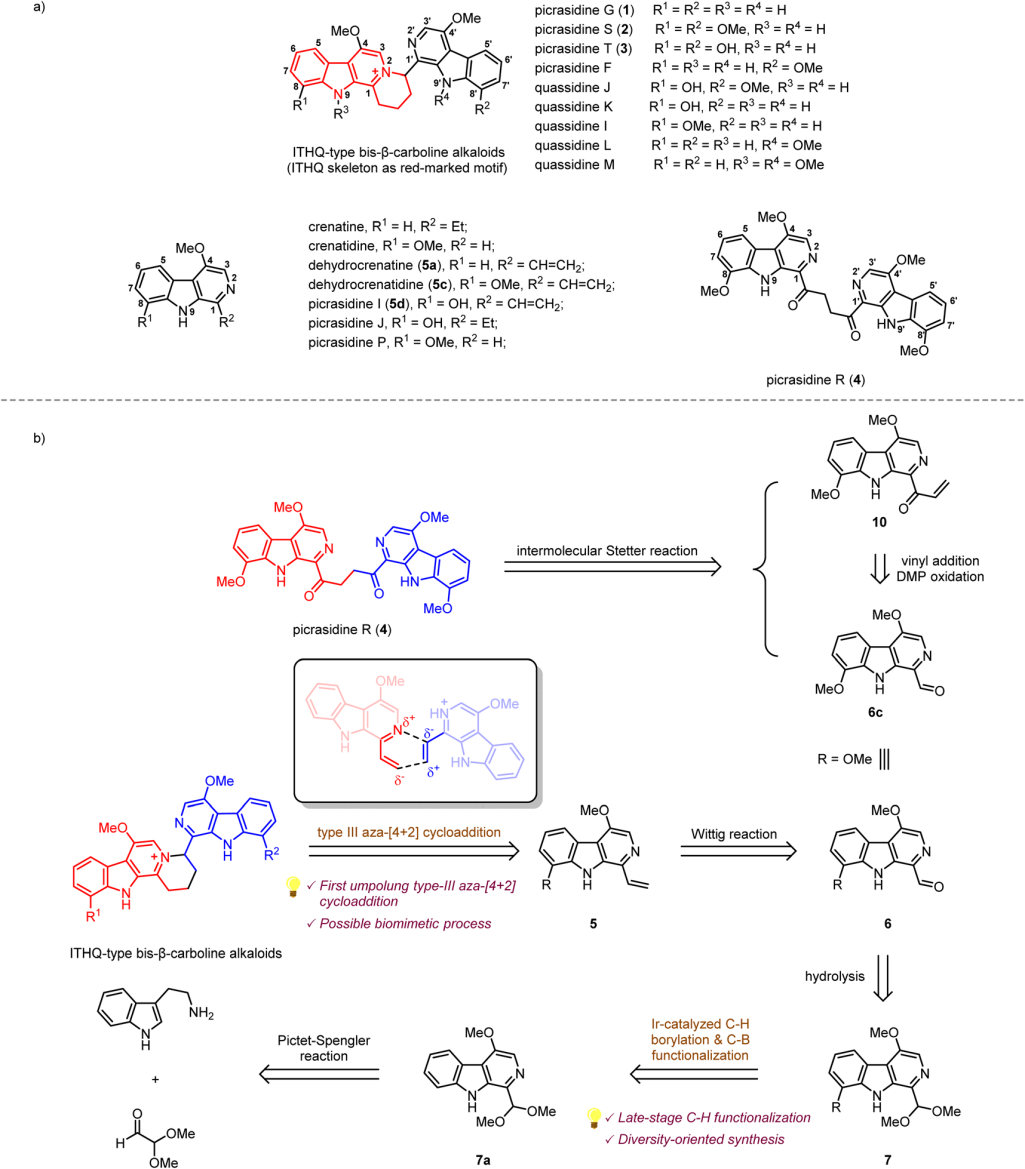
(a) Structures of several β-carboline alkaloids from *Picrasma quassioides*. (b) Retrosynthetic analysis of ITHQ-type bis-β-carboline alkaloids and picrasidine R.

β-Carboline alkaloids widely exist in a variety of animals^1^ and plants.^2^ These alkaloids have been discovered to possess diverse biological activities, such as anti-cancer, anti-inflammatory, and anti-malaria.^3^ Since the 1980s, a large number of bis-β-carboline alkaloids have been isolated by Ohmoto,^4–17^ Yao,^18–21^ Song,^22^ Tian^23^*et al.* from plants of the *Simaroubaceae* family. Interestingly, some of these alkaloids, such as picrasidines G (1), S (2), and T (3), feature a unique indolotetrahydroquinolizinium (ITHQ) skeleton (Scheme 1a). All these ITHQ-type bis-β-carboline alkaloids bear a methoxy group at the C-4 position on each β-carboline ring, and some of them bear a hydroxy group or methoxy group at the C-8 or N-9 position. Despite the presence of a stereogenic center in ITHQ-type bis-β-carboline alkaloids, racemization can easily occur at room temperature in chloroform solution.^20^ Most of these ITHQ-type bis-β-carboline alkaloids show cytotoxic bioactivity. In 2015, Lin and coworkers reported the cytotoxicity and antibacterial bioactivities of picrasidines G (1), S (2), and F.^24^ Picrasidine F shows selective cytotoxicity against the HeLa cancer cell line (IC_50_ = 16.65 μM) compared to the MKN-28 (IC_50_ = 145.50 μM) and B-16 (IC_50_ = 95.48 μM) cancer cell lines, while picrasidines G (1) and S (2) show cytotoxicity against all three cancer cell lines (IC_50_ = 4.95–19.00 μM). These three alkaloids also show antibacterial bioactivity against MRSA P0172, MRSA H0117, MSSA P0171 and MSSA H0180 (MIC = 4–16 μg mL^−1^). These authors also discovered that the other two ITHQ-type bis-β-carboline alkaloids (±) quassidines I and J show cytotoxicity against the HeLa, MNK-28 and B-16 cancer cell lines (IC_50_ = 4.03–15.4 μM).^20^ In 2018, Tian and coworkers reported cytotoxicity of (±) quassidine K against the HeLa cancer cell line (IC_50_ = 15.8–20.1 μM).^23^ It is interesting to note that (+)-*S* configurated ITHQ-type bis-β-carboline alkaloids are generally more cytotoxic than the corresponding (−)-*R* configurated ones, but the difference in activity is only about 1.5 to 2-fold, probably due to the spontaneous racemization.^20,23^ In 2017, Kanno and coworkers reported that picrasidine G increases the caspase-dependent cell apoptosis by inhibiting the EGF-induced STAT3 phosphorylation, thus decreasing the viability of EGFR-overexpressing triple-negative breast cancer cells.^25^

The biosynthetic pathway of ITHQ-type bis-β-carboline alkaloids is still unknown. Yao and Gao proposed a putative skeleton rearrangement mechanism. They envisaged that the ITHQ skeleton might be rearranged from a four-membered ring, which could be generated by nucleophilic substitution^18^ or [2 + 2] cycloaddition^21,26^ (Scheme S1†). However, such a hypothesis lacks corresponding experimental evidence. Here we proposed another possible synthetic route towards ITHQ-type bis-β-carboline alkaloids—aza-[4 + 2] cycloaddition. We speculated that ITHQ-type bis-β-carboline alkaloids might be generated *via* a late-stage regio-selective aza-[4 + 2] cycloaddition of two vinyl substituted monomeric β-carboline alkaloids, some of which were identified as natural products as well, such as dehydrocrenatine (5a), picrasidine I (5c) and dehydrocrenatidine (5d). Therefore, it would be intriguing to test such aza-[4 + 2] cycloaddition for a biomimetic synthesis.

No synthetic studies toward ITHQ-type bis-β-carboline alkaloids have been reported to date. However, several studies on the syntheses of 1-vinyl substituted monomeric β-carboline alkaloids have been reported (Scheme S2†). In 1982, Cook and coworkers reported the total syntheses of crenatine with the C-4 methoxy substituted β-carboline skeleton by a Pictet–Spengler reaction and DDQ oxidation strategy.^27^ In 2005, Ihara and coworkers reported the total synthesis of dehydrocrenatine (5a) *via* the application of Cook's strategy.^28^ In 1999, Murakami and coworkers reported the syntheses C-4 and C-8 disubstituted β-carboline alkaloids picrasidines I (5d), J and P, as well as crenatidine and dehydrocrenatidine (5c) by Fischer indole synthesis.^29^ In 2005, Murakami and coworkers reported a more concise Fischer indole synthesis strategy for the syntheses of C-4 and C-6 disubstituted β-carboline alkaloids.^30^

Our retrosynthetic analysis of ITHQ-type bis-β-carboline alkaloids is illustrated in Scheme 1b. As mentioned above, the ITHQ skeleton could be directly constructed by a late-stage aza-[4 + 2] cycloaddition of 1-vinyl substituted monomeric β-carboline alkaloids. The vinyl group at the C-1 position could be installed from 7 by hydrolysis of dimethyl acetal followed by a Wittig reaction. Substituent groups at the C-8 position could be installed by the iridium-catalyzed C–H bond borylation followed by C–B bond functionalization. The synthesis of precursor 7a should be achieved *via* a Pictet–Spengler reaction and DDQ oxidation inspired by Cook and Ihara's studies.^27,28^ Moreover, another bis-β-carboline alkaloid picrasidine R (4) could be synthesized *via* a Stetter reaction from vinyl ketone 10 and aldehyde 6c. The vinyl ketone 10 could also be obtained from aldehyde 6c by vinyl addition and a subsequent oxidation.

## Results and discussion

### Total syntheses of bis-β-carboline alkaloids and derivatives

**Scheme 2:**
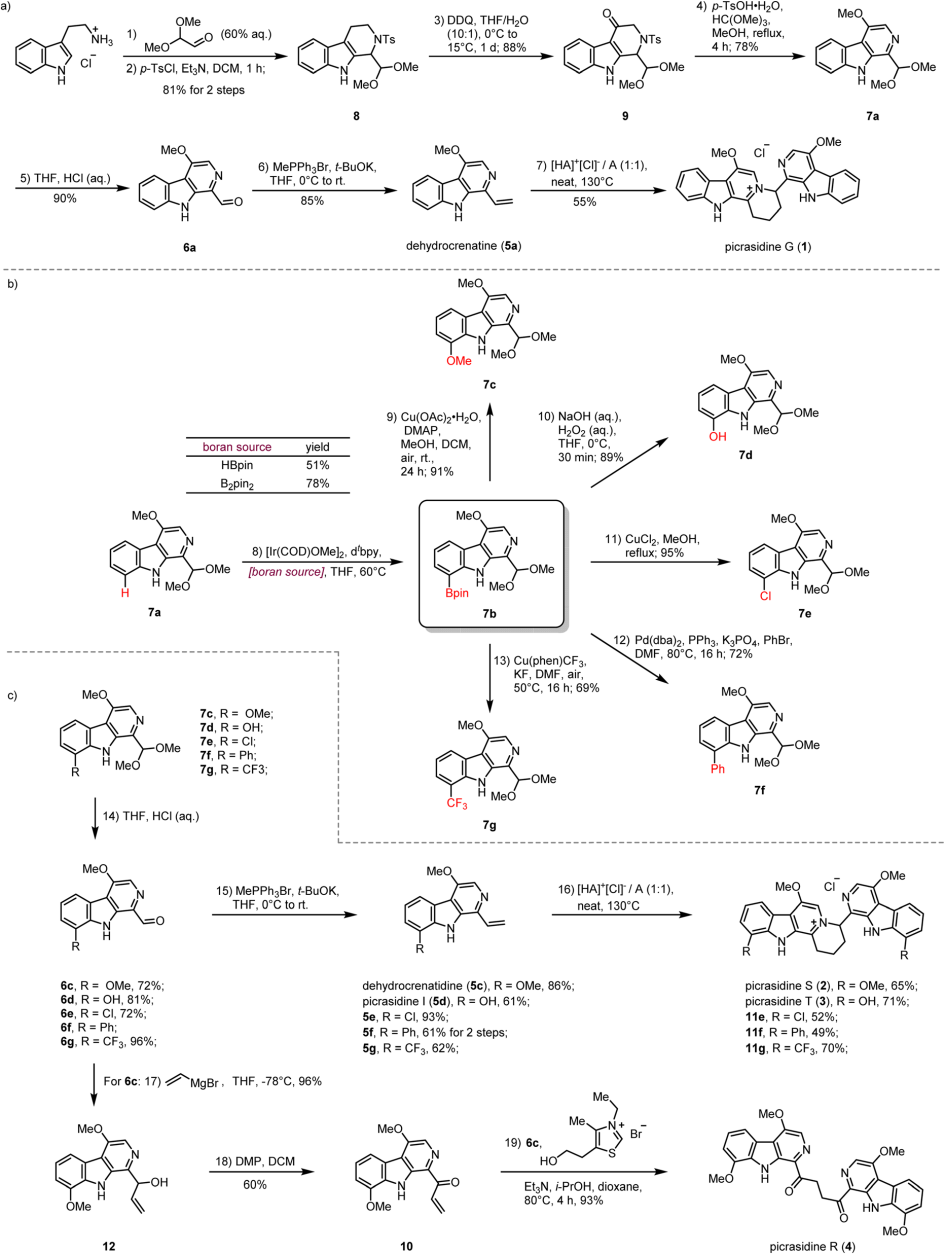
(a) Syntheses of picrasidines G (1) by the aza-[4 + 2] cycloaddition. (b) Late-stage C–H functionalization strategy for diversity-oriented syntheses of C-8 substituted β-carboline alkaloids. (c) Total syntheses of picrasidines S (2), T (3), R (4) and 11e, 11f, 11g.

Our synthesis commenced with the preparation of the monomeric C-1 vinyl substituted β-carboline dehydrocrenatine (5a) (Scheme 2a). The tetrahydro-β-carboline skeleton of compound 8 was constructed by a Pictet–Spengler reaction between tryptamine hydrochloride and 2,2-dimethoxyacetaldehyde,^31^ followed by tosyl protection of the secondary amine. Then benzyl oxidation of compound 8 by DDQ afforded ketone 9.^27,28^ Acetalmethylation of ketone 9 by the treatment with trimethyl orthoformate in acidic methanol solution led to *in situ* elimination of the tosyl group and aromatization to afford the key synthetic building block 7a, bearing the β-carboline structure with a methoxy group at the C-4 position. Compared with the acetyl group, usage of the tosyl group could avoid extra addition of oxidant for aromatization.^28,30^ After hydrolysis of the dimethyl acetal to form the aldehyde group and installation of the vinyl group by the Wittig reaction, we successfully prepared the desired C-1 vinyl substituted β-carboline dehydrocrenatine (5a).

To explore the feasibility of the proposed regio-selective aza-[4 + 2] cycloaddition, dehydrocrenatine (5a) was chosen as a model substrate. We found that reactions performed in water solution or dioxane solution could only lead to trace formation of the dimerization product (Table 1, entries 1–5). However, upon heating an equimolar mixture of free alkaloid and its hydrochloride salt to 100 °C for 4 h under neat conditions, 40% of the reactant could be converted to the dimerization product (Table 1, entry 6). Raising the temperature to 130 °C could give almost full conversion of the reactant, with 55% isolated yield (Table 1, entry 9). The product was confirmed as the ITHQ-type bis-β-carboline alkaloid picrasidine G (1), demonstrating the feasibility of the aza-[4 + 2] cycloaddition. Although using free alkaloid as the reactant alone could also form a small amount of the dimerization product, the conversion was relatively low (Table 1, entry 11). On the other hand, using hydrochloride salt as the reactant alone could only lead to trace amounts of the dimerization product (Table 1, entry 10). This result showed that such aza-[4 + 2] cycloadditions were prone to occur between one molecule of free alkaloid and another molecule of hydrochloride salt.

**Table 1:** Reaction screening of the aza-[4 + 2] cycloaddition

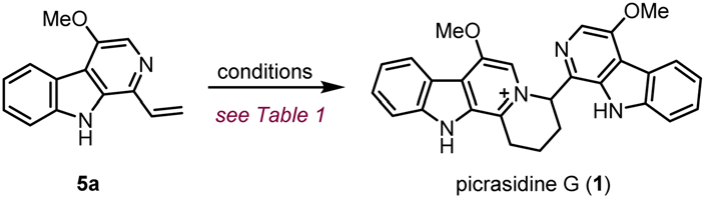				
Entry	Reactant^a^	Solvent^b^	*T*/°C	Yield^g^
1	[HA]^+^Cl^−^/A (1 : 1)	H_2_O	100	<5%
2	[HA]^+^Cl^−^	pH = 6 buffer^c^	100	<5%
3	[HA]^+^Cl^−^	pH = 7 buffer^d^	100	<5%
4	[HA]^+^Cl^−^	pH = 8 buffer^e^	100	<5%
5	[HA]^+^Cl^−^/A (1 : 1)	1,4-Dioxane	100	<5%
6	[HA]^+^Cl^−^/A (1 : 1)	(Neat)^f^	100	40%
7	[HA]^+^[AcO]^−^	(Neat)^f^	100	23%
8	[HA]^+^[AcO]^−^/A (1 : 1)	(Neat)^f^	100	9%
**9**	**[HA]** ^**+**^ **Cl** ^**−**^ **/A (1** **:** **1)**	**(Neat)^f^**	**130**	**86% (55%^h^)**
10	[HA]^+^Cl^−^	(Neat)^f^	130	<5%
11	A	(Neat)^f^	130	31%

a Reactions were performed using dehydrocrenatine (5a, represented by A) and its corresponding salt (represented by [HA]^+^X^−^) as reactants.

b Unless otherwise stated, reactions were performed at 10 mM concentration.

c In 25 mM KPi buffer (pH = 6).

d In 25 mM Tris buffer (pH = 7).

e In 25 mM HEPES buffer (pH = 8).

f Reaction was performed in the solid state without any solvent.

g Unless otherwise stated, yields were based on ^1^H-NMR.

h Isolated yields.

Compared to the simplest ITHQ-type bis-β-carboline alkaloid picrasidine G (1), picrasidines S (2) and T (3) bear an additional oxidation state at the C-8 position. We envisaged a late-stage C–H borylation strategy for the functionalization of the C-8 position.^32–36^ Late-stage functionalization enables effective and flexible diversification at specific sites of natural products for drug discovery and chemical biology.^37–40^ Previously, Smith and coworkers reported the iridium-catalyzed C–H borylation at C-2 and C-7 positions of the indole ring.^41^ They found that if the C-2 position was blocked, the C–H borylation could only happen at the C-7 position. To our delight, such a methodology worked well for C-8 site-selective C–H borylation of 7a. Both HBpin and B_2_pin_2_ could be used as a boron source to prepare the boranate 7b, while B_2_pin_2_ showed higher yield compared to HBpin. Combined with C–B bond functionalization methodologies such as Chan–Lam coupling (7c),^42^ oxidation by hydrogen peroxide (7d),^43^ halogenation (7e),^43,44^ Suzuki coupling (7f)^44^ and trifloromethylation (7g),^45^ we could easily install diverse functional groups at the C-8 position (Scheme 2b). By the same route, all monomeric alkaloids 7c–g could be successfully transformed into the corresponding ITHQ-type bis-β-carboline alkaloids (Scheme 2c). Moreover, aldehyde 6c could be used for the concise synthesis of a bis-β-carboline alkaloid picrasidine R (4). Vinyl addition to the aldehyde 6c followed by Dess–Martin oxidation afforded the vinyl ketone 10, which could undergo a thiazolium-catalyzed Stetter reaction with the aldehyde 6c to afford picrasidine R (4) in 90% yield (Scheme 2c).^46^

### Computational studies of the aza-[4 + 2] cycloaddition

**Fig. 1:**
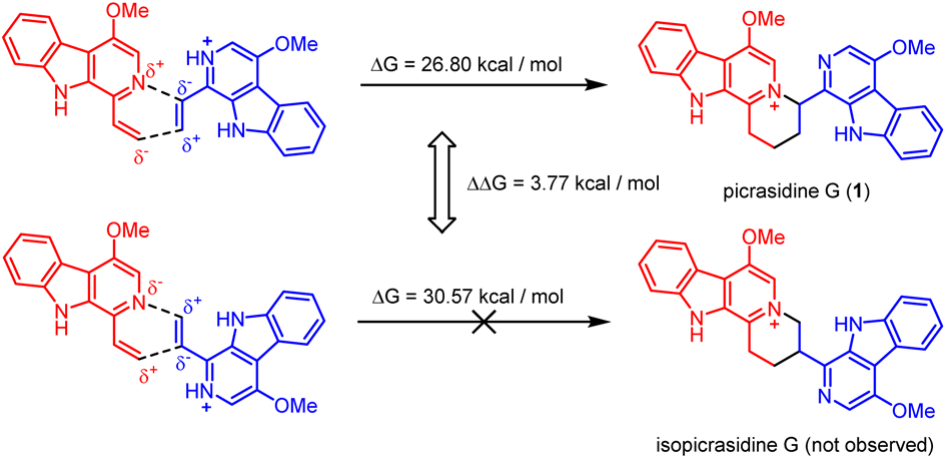
Two possible pathways of the aza-[4 + 2] cycloaddition.

**Scheme 3:**
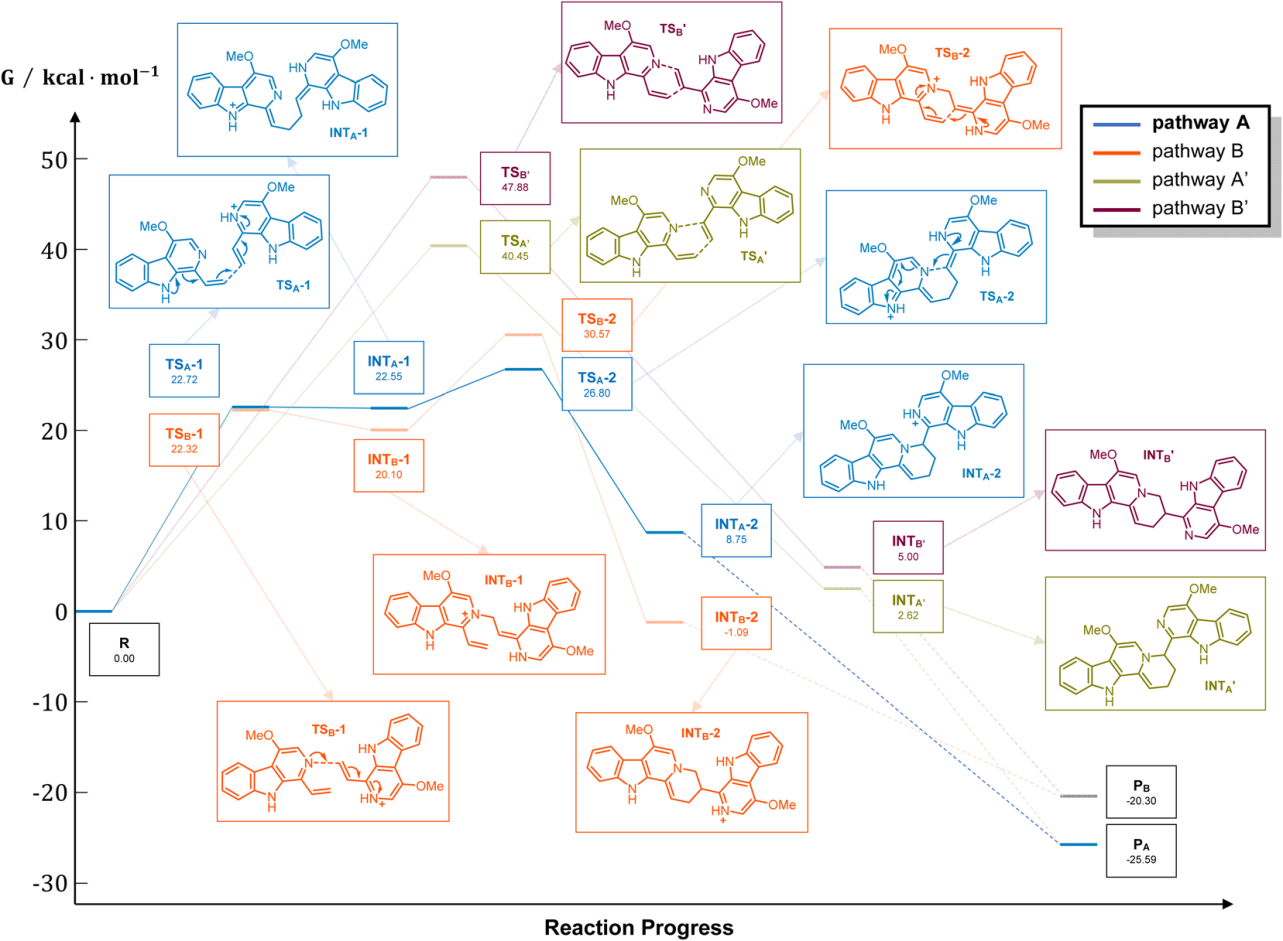
Energy profile of the aza-[4 + 2] cycloaddition. For pathways A and B, R = 1 × dehydrocrenatine (10a) and 1 × protonated dehydrocrenatine; for pathways A′ and B′, R = 2 × dehydrocrenatine. P_A_ = picrasidine G (1), P_B_ = isopicrasidine G. Calculation at WB97XD/6-311++G(2d,p)//WB97XD/6-31G(d) at 403.15 K. Gibbs free energies (Δ*G*) are in kcal mol^−1^.

It should be noticed that theoretically there may be another possible pathway of the aza-[4 + 2] cycloaddition, leading to isopicrasidine G as the product, as is shown in Fig. 1. However, isopicrasidine G was neither identified in the aza-[4 + 2] cycloaddition, nor from natural product isolation. Generally, for the type-III aza-[4 + 2] cycloaddition (Scheme S3†), the terminal alkene position of the vinyl imine structure is prone to act as an electrophile (Scheme S3,† Ihara's work as an example^47–49^). However, our observation of the reaction outcome was different. It is noteworthy that such a transformation is the first reported umpolung type-III aza-[4 + 2] cycloaddition, whose regioselectivity is different from those of all previously reported ones. To explain the unique regioselectivity and provide us mechanistic insights, the reaction profile was further investigated by DFT calculations (Scheme 3). Interestingly, if we select two molecules of free alkaloid (dehydrocrenatine, 5a) as the reactant, they will undergo a concerted aza-[4 + 2] cycloaddition (Scheme 3, pathways A′ and B′) with the activation Gibbs energies (Δ*G*) up to 40.45 kcal mol^−1^ for pathway A′ to form picrasidine G (1, P_A_) and 47.88 kcal mol^−1^ for pathway B′ to form isopicrasidine G (P_B_). However, if we select one molecule of free alkaloid and another molecule of the protonated alkaloid as reactants, they will undergo a stepwise, formal aza-[4 + 2] cycloaddition (Scheme 3, pathways A and B), and the activation Gibbs free energies decrease to 26.80 kcal mol^−1^ for pathway A and 30.57 kcal mol^−1^ for pathway B. Kinetically, the difference of activation Gibbs free energy (ΔΔ*G*) between pathways A and B is up to 3.77 kcal mol^−1^, which explains such excellent regioselectivity.

According to the computational result, the stepwise pathway A is the most favored mechanism under the experimental conditions, with just 26.80 kcal mol^−1^ activation Gibbs energies to overcome. For the free alkaloid, the electron-donating effect of the N-9 nitrogen atom and the C-4 methoxy group inverts the intrinsic polarity of the “pyridine ring”, making the terminal alkene nucleophilic rather than electrophilic. However for the protonated alkaloids, the “pyridine ring” maintained its intrinsic polarity due to the protonation, making the terminal alkene a Michael acceptor. First, the terminal alkene of the free alkaloid acts as a nucleophile to attack the terminal alkene of the protonated alkaloid, forming INT_A_-1 *via* TS_A_-1 (Δ*G* = 22.72 kcal mol^−1^). Then the succeeding annulation process of the unstable INT_A_-1 can easily take place *via* TS_A_-2 to form INT_A_-2, requiring only 4.25 kcal mol^−1^ energy expense. Finally, the intramolecular proton transfer decreases the total energy of the system, making the overall reaction thermodynamically favored. The computational results revealed that picrasidine G (1) is the more favorable product both kinetically and thermodynamically compared to the proposed isopicrasidine G, and the aza-[4 + 2] cycloaddition prefers a stepwise mechanism.

### Biological evaluation of the synthesized bis-β-carboline compounds

We also tested the anti-cancer activities of the synthesized bis-β-carboline compounds picrasidines G (1), S (2), T (3), R (4) and 11e–g on multiple human cancer cell lines, including THP-1 (monocytic leukaemia), MCF-7 (epithelial luminal), HepG2 (hepatoma), and A375 (melanoma) (Table 2). Most of the ITHQ type bis-β-carboline alkaloids showed cytotoxicity against the above-mentioned cancer cell lines. Besides, these ITHQ type bis-β-carboline compounds showed selectivity for different cancer cell lines. For example, picraisidine G (1) showed potent cytotoxicity to THP-1 (IC_50_ = 3.7 μM), but relatively weak cytotoxicity to HepG2 (IC_50_ = 64.6 μM). Picrasidine S (2) showed stronger cytotoxicity against THP-1 and HepG2 (IC_50_ = 3.3–5.5 μM) compared to MCF-7 and A375 (IC_50_ = 15.9–20.4 μM). In contrast, 11f and 11g showed potent cytotoxicity against all four cancer cell lines (IC_50_ = 3.1–8.1 μM).

**Table 2:** Cytotoxicity of compounds against multiple cancer cell lines^a^

Compounds	IC_50_ (μM, at 48 h)			
	THP-1	MCF-7	HepG2	A375
Picrasidine G (1)	3.7 ± 1.1	13.2 ± 2.2	64.6 ± 9.6	16.5 ± 3.5
Picrasidine S (2)	3.3 ± 0.6	20.4 ± 1.6	5.5 ± 0.7	15.9 ± 3.4
Picrasidine T (3)	13.2 ± 3.4	52.2 ± 9.2	19.5 ± 2.1	18.9 ± 2.8
Picrasidine R (4)	>100	81.2 ± 8.3	>100	>100
11e	19.3 ± 4.2	22.0 ± 3.0	80.8 ± 8.8	>100
11f	3.1 ± 0.7	5.8 ± 1.1	4.9 ± 1.4	8.1 ± 2.2
11g	3.1 ± 0.8	4.5 ± 1.4	3.8 ± 1.4	5.2 ± 1.4

a Cell viability was assessed using the CellTiter-Glo^®^ assay kit (Promega, USA).

## Conclusions

We have successfully accomplished the first total syntheses of three ITHQ-type bis-β-carboline alkaloids picrasidines G, S and T and several derivatives by the regio-selective aza-[4 + 2] cycloaddition. The C-8 oxidation state was efficiently installed by the iridium catalyzed site-selective C–H borylation and C–B bond functionalization strategy, enabling the diversity-oriented syntheses of several natural product-like derivatives.^50–52^ Computational studies revealed a stepwise mechanism of the aza-[4 + 2] cycloaddition and explained the origin of excellent regioselectivity. Another bis-β-carboline alkaloid picrasidine R was also efficiently synthesized *via* the thiazolium-catalyzed Stetter reaction. This work provides synthetic evidence for the proposed biosynthetic pathway of ITHQ-type bis-β-carboline alkaloids.

## Data availability

Additional experimental and computational data supporting this article are included in the ESI.†

## Author contributions

Prof. X. Lei and Q. Wang conceived the project. Q. Wang conducted the synthetic and computational studies. F. Guo conducted the bioactivity test. All experiments were conducted under the supervision of Prof. X. Lei. J. Wang provided helpful suggestions in the project. Q. Wang wrote the manuscript with input from Prof. X. Lei, F. Guo and J. Wang. Prof. X. Lei managed the whole project.

## Conflicts of interest

There are no conflicts to declare.

## Supplementary Material

SC-014-D3SC03722C-s001

## References

[cit1] Kleks G., Duffy S., Lucantoni L., Avery V. M., Carroll A. R. (2020). J. Nat. Prod..

[cit2] Han J., Lv T., Song S., Huang X. (2023). Biochem. Syst. Ecol..

[cit3] Chen Q., Ye X., Liu X., Liang Y., Feng W., Li C., Wang Z. (2022). Phytochemistry.

[cit4] Ohmoto T., Koike K. (1982). Chem. Pharm. Bull..

[cit5] Ohmoto T., Koike K. (1983). Chem. Pharm. Bull..

[cit6] Ohmoto T., Koike K. (1984). Chem. Pharm. Bull..

[cit7] Ohmoto T., Koike K. (1985). Chem. Pharm. Bull..

[cit8] Ohmoto T., Koike K. (1985). Chem. Pharm. Bull..

[cit9] Ohmoto T., Koike K., Higuchi T., Ikeda K. (1985). Chem. Pharm. Bull..

[cit10] Koike K., Ohmoto T. (1986). Chem. Pharm. Bull..

[cit11] Koike K., Ohmoto T., Ogata K. (1986). Chem. Pharm. Bull..

[cit12] Koike K., Ohmoto T. (1987). Chem. Pharm. Bull..

[cit13] Koike K., Ohmoto T., Higuchi T. (1987). Phytochemistry.

[cit14] Koike K., Ohmoto T. (1988). Phytochemistry.

[cit15] Koike K., Ohmoto T., Ikeda K. (1990). Phytochemistry.

[cit16] Murakami Y., Yokoyama Y., Aoki C., Suzuki H., Sakurai K., Shinohara T., Miyagi C., Kimura Y., Takahashi T., Watanabe T., Ohmoto T. (1991). Chem. Pharm. Bull..

[cit17] Li H., Koike K., Ohmoto T. (1993). Chem. Pharm. Bull..

[cit18] Jiao W., Gao H., Li C., Zhao F., Jiang R., Wang Y., Zhou G., Yao X. (2010). J. Nat. Prod..

[cit19] Jiao W., Gao H., Zhao F., Lin H., Pan Y.-M., Zhou G., Yao X. (2011). Chem. Pharm. Bull..

[cit20] Jiao W., Chen G., Gao H., Li J., Gu B.-B., Xu T., Yu H., Shi G., Yang F., Yao X., Lin H. (2015). J. Nat. Prod..

[cit21] Zhang J., Zhao S., Xie J., Yang J., Chen G., Hu D., Zhang W., Wang C., Yao X., Gao H. (2020). Bioorg. Chem..

[cit22] Zhao W., Zhou W., Chen J., Yao G., Lin B., Wang X., Huang X., Song S. (2019). Phytochemistry.

[cit23] Guo X., Li F., Zheng F., Gong N., Li Y., Feng W., Tian L. (2020). Nat. Prod. Res..

[cit24] Shi G., Jiao W., Yang F., Lin H. (2015). Chin. Tradit. Herb. Drugs.

[cit25] Yamashita N., Kondo M., Zhao S., Li W., Koike K., Nemoto K., Kanno Y. (2017). Bioorg. Med. Chem. Lett..

[cit26] Yi L., He Y., Tan S., White L. V., Lan P., Gardiner M. G., Pei Z., Coote M. L., Banwell M. G. (2022). J. Org. Chem..

[cit27] Hagen T. J., Narayanan K., Names J., Cook J. M. (1989). J. Org. Chem..

[cit28] Takasu K., Shimogama T., Saiin C., Kim H.-S., Wataya Y., Brun R., Ihara M. (2005). Chem. Pharm. Bull..

[cit29] Suzuki H., Unemoto M., Hagiwara M., Ohyama T., Yokoyama Y., Murakami Y. (1999). J. Chem. Soc., Perkin Trans. 1.

[cit30] Suzuki H., Tsukakoshi Y., Tachikawa T., Miura Y., Adachi M., Murakami Y. (2005). Tetrahedron Lett..

[cit31] Gaikwad S., Kamble D., Lokhande P. (2018). Tetrahedron Lett..

[cit32] Fischer D. F., Sarpong R. (2010). J. Am. Chem. Soc..

[cit33] Newton J. N., Fischer D. F., Sarpong R. (2013). Angew. Chem., Int. Ed..

[cit34] Jiang B., Dai M. (2021). J. Am. Chem. Soc..

[cit35] Wu F., Zhang J., Song F., Wang S., Guo H., Wei Q., Dai H., Chen X., Xia X., Liu X., Zhang L., Yu J.-Q., Lei X. (2020). ACS Cent. Sci..

[cit36] Hong B., Li C., Wang Z., Chen J., Li H., Lei X. (2015). J. Am. Chem. Soc..

[cit37] Hong B., Luo T., Lei X. (2020). ACS Cent. Sci..

[cit38] Wang S., Chen K., Guo F., Zhu W., Liu C., Dong H., Yu J.-Q., Lei X. (2023). ACS Cent. Sci..

[cit39] Liu W., Hong B., Wang J., Lei X. (2020). Acc. Chem. Res..

[cit40] Chen K., Lei X. (2018). Curr. Opin. Green Sustainable Chem..

[cit41] Paul S., Chotana G. A., Holmes D., Reichle R. C., Maleczka R. E., Smith M. R. (2006). J. Am. Chem. Soc..

[cit42] Chen P., Yang H., Zhang H., Chen W., Zhang Z., Zhang J., Li H., Wang X., Xie X., She X. (2020). Org. Lett..

[cit43] Loach R. P., Fenton O. S., Amaike K., Siegel D. S., Ozkal E., Movassaghi M. (2014). J. Org. Chem..

[cit44] Robbins D. W., Boebel T. A., Hartwig J. F. (2010). J. Am. Chem. Soc..

[cit45] Cheng C., Hartwig J. F. (2014). Science.

[cit46] Harrington P. E., Tius M. A. (2001). J. Am. Chem. Soc..

[cit47] Ihara M., Suzuki M. (2000). Heterocycles.

[cit48] Ihara M., Taniguchi T., Makita K., Takano M., Ohnishi M., Taniguchi N., Fukumoto K., Kabuto C. (2002). J. Am. Chem. Soc..

[cit49] Takasu K., Nishida N., Tomimura A., Ihara M. (2005). J. Org. Chem..

[cit50] Wang J., Ke H., Yang J., Guo N., Hu K., Tang R., Ding Q., Gao L., Lei X. (2023). Chem Catal..

[cit51] Chen K., Wu F., Lei X. (2021). Chin. J. Chem..

[cit52] Zhang J., Wu J., Hong B., Ai W., Wang X., Li H., Lei X. (2014). Nat. Commun..

