# Supplementary material for: Divergent total syntheses of ITHQ-type bis-β-carboline alkaloids by regio-selective formal aza-[4 + 2] cycloaddition and late-stage C–H functionalization
Source: Chem Sci. 2023 Sep 13;14(37):10353–9. doi: 10.1039/d3sc03722c (PMC10530148; doi:10.1039/d3sc03722c)
Supplement: SC-014-D3SC03722C-s001 [file SC-014-D3SC03722C-s001.pdf]

Electronic Supplementary Material (ESI) for Chemical Science.
This journal is © The Royal Society of Chemistry 2023

Supporting Information

Divergent Total Syntheses of ITHQ-type Bis-β-carboline Alkaloids by Regio-selective Formal Aza-[4+2] Cycloaddition and Late-stage C-H Functionalization

Qixuan Wang, Fusheng Guo, Jin Wang, Xiaoguang Lei *

Table of Contents

1. **General Information** 3
2. **Extended Tables and Figures** 4

Scheme S1 Previously reported putative biosynthetic pathway of ITHQ-type bis-β-carboline alkaloids by Yao and Gao 4

Scheme S2 Previous reported two classic syntheses of C-4 methoxy substituted β-carboline alkaloids[5, 6] 4

Scheme S3 Previous reported aza-[4+2] cycloaddition reaction in three types[7-11]. 5

Scheme S3 Example of type II umpolung aza-[4+2] cycloaddition reaction in natural product biosynthesis. . 6

Table S1 Optimization of iridium-catalyzed C-8 C-H borylation reaction of **7a**[a]. 7

Table S2 Comparison of synthetic and literature-reported[12] ^{1}H-NMR data of dehydrocrenatine (**7a**). 8

Table S3 Comparison of synthetic and literature-reported[13] ^{13}C-NMR data of dehydrocrenatine (**7a**). 9

Table S4 Comparison of synthetic and literature-reported[12] ^{1}H-NMR data of dehydrocrenatidine (**7c**)........ 10

Table S5 Comparison of synthetic and literature-reported[13] ^{13}C-NMR data of dehydrocrenatidine (**7c**). 11

Table S6 Comparison of synthetic and literature-reported[14] ^{1}H-NMR data of picrasidine I (**7d**). 12

Table S7 Comparison of synthetic and literature-reported[14] ^{13}C-NMR data of picrasidine I (**7d**). 13

Table S8 Comparison of synthetic and literature-reported[15] ^{1}H-NMR data of picrasidine G (**1**). 14

Table S9 Comparison of synthetic and literature-reported[15] ^{13}C-NMR data of picrasidine G (**1**). 15

Table S10 Comparison of synthetic and literature-reported[15] ^{1}H-NMR data of picrasidine S (**2**). 16

Table S11 Comparison of synthetic and literature-reported[15] ^{13}C-NMR data of picrasidine S (**2**). 17

Table S12 Comparison of synthetic and literature-reported[16] ^{1}H-NMR data of picrasidine T (**3**). 18

Table S13 Comparison of synthetic and literature-reported[17] ^{1}H-NMR data of picrasidine R (**4**). 19

Table S14 Comparison of synthetic and literature-reported[17] ^{13}C-NMR data of picrasidine R (**4**). 20

3. **Experimental Procedures and Characterization Data** 21
4. **NMR Spectrums** 35
5. **Computational Results** 97
6. **References** 127

1. General Information

1) General information for syntheses and purification

All reagents were used as supplied by Sigma-Aldrich, J&K, Alfa Aesar Chemicals, TCI. Unless otherwise stated, tetrahydrofuran and diethyl ether were distilled from sodium/benzophenone ketyl prior to use; the other solvents were distilled from calcium hydride. All reactions were carried out in oven-dried glassware under an argon atmosphere. Flash chromatography was performed using 200-400 mesh silica gel, unless otherwise stated. Yields refer to chromatographically and spectroscopically pure materials, unless otherwise stated.

2) General information for characterization

All NMR spectra were recorded on Bruker 400 MHz spectrometer (ARX400), Bruker 500 MHz spectrometer (AVANCE III) and Bruker 600 MHz spectrometer (AVIIIHD) at ambient temperature, unless otherwise stated. Chemical shifts[1] are reported in parts per million (ppm) relative to Chloroform-*d* (^{1}H, δ 7.26; ^{13}C, δ 77.16), DMSO-d_6 (^{1}H, δ 2.50; ^{13}C, δ 39.52). Data for ^{1}H NMR are reported as follows: chemical shift, integration, multiplicity (s = singlet, d = doublet, t = triplet, q = quartet, m = multiplet) and coupling constants. High-resolution mass spectra were recorded on Bruker Solarix XR FTMS.

3) General information for computational studies

Gaussian 16[2] was used for all DFT calculations. The geometric optimization and vibration analysis of the molecules were performed using WB97XD functional and 6-31G(d) basis set. At the same time, the Goodvibes[3] software developed by Paton laboratory was used to calculate the energy contribution to the vibrational and rotational parts of the free energy by quasi-resonance approximation based on the vibration analysis results. WB97XD functional and 6-311++G(2d, p) basis set were used to calculate the single point energy of the system. The CREST[4] software developed by Grimme lab was used to search for the structure, ensuring that we had the lowest energy transition state.

4) General information for cell culture and cytotoxicity assay

THP-1 (monocytic leukaemia) was cultured in RPMI 1640 medium, MCF-7 (epithelial luminal), HepG2 (hepatoma) and A375 (melanoma) were cultured in DMEM medium. All medium supplemented with 10% (v/v) fetal bovine serum (PAN-Seratech, Cat#ST30-3302) and cells were cultured in an incubator containing 5% CO_2. 10,000 cells per well were seeded into 96-well culture plates and cultured overnight to reach decent cell growth condition, then the compounds were added to the medium at gradient concentrations at 37°C for 48 hours. Finally, cell viability was determined by CellTiter-Glo® luminescent cell viability assay kit (Promega, USA, CAT#G7573) according to the manufacturer's instructions.

2. Extended Tables and Figures

Scheme S1 Previously reported putative biosynthetic pathways of ITHQ-type bis-β-carboline alkaloids by Yao and Gao.[5, 6]

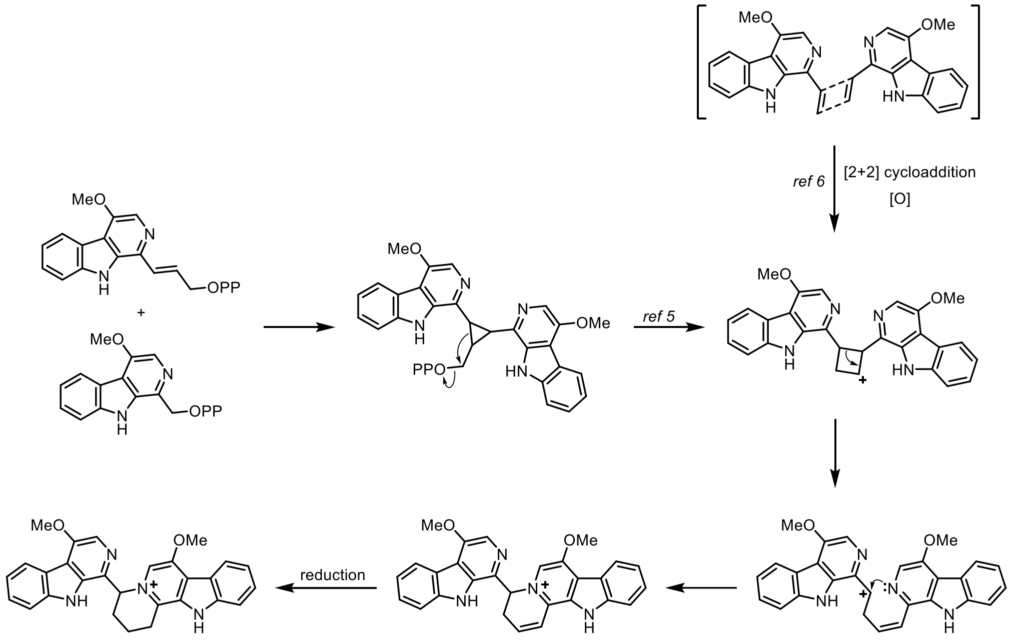

Scheme S2 Previous reported two classic syntheses of C-4 methoxy substituted β-carboline alkaloids[7, 8]

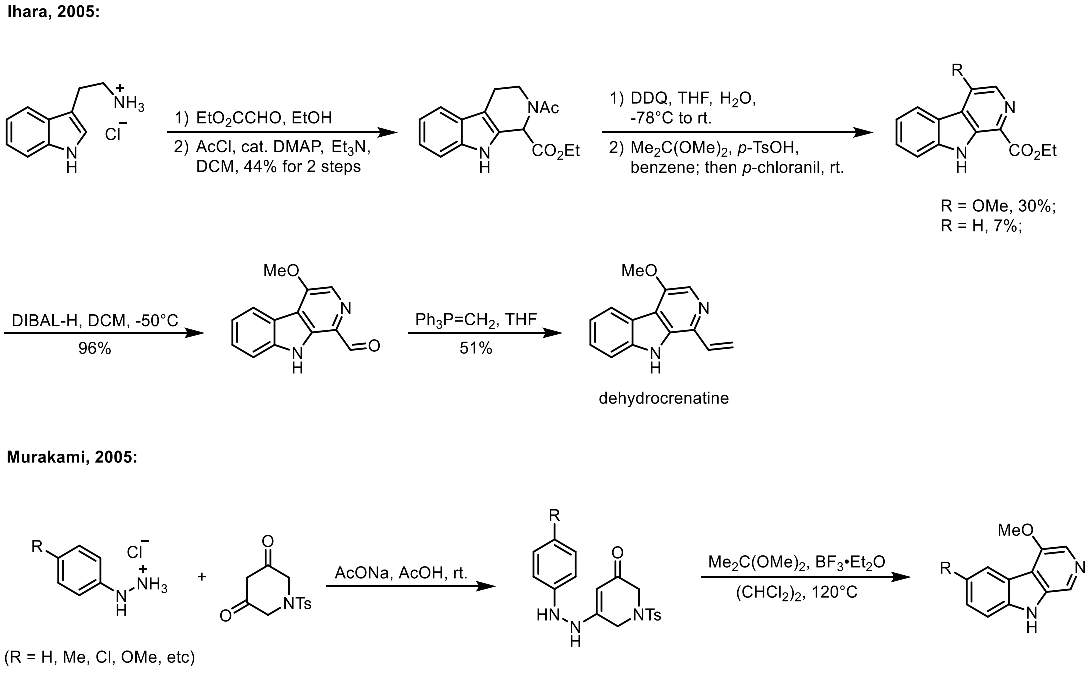

Scheme S3 Previous reported aza-[4+2] cycloaddition reaction in three types[9-13].

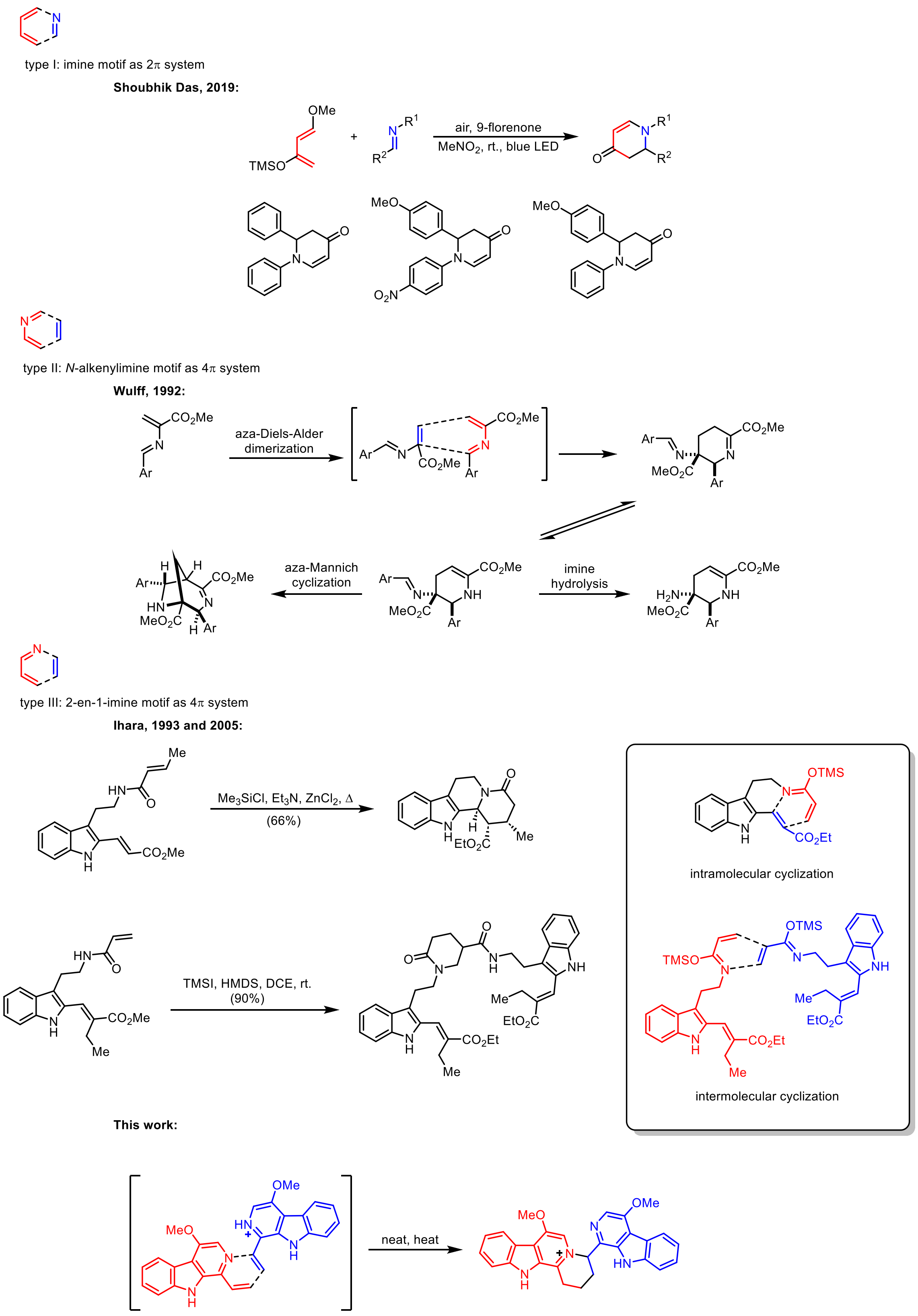

Scheme S4 Example of umpolung type II aza-[4+2] cycloaddition reaction in natural product biosynthesis.

a)

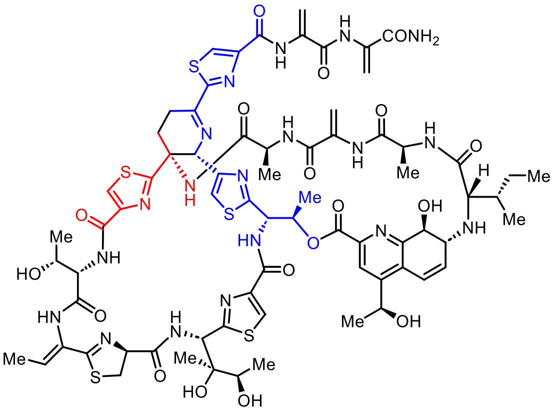

thiostrepton

b)

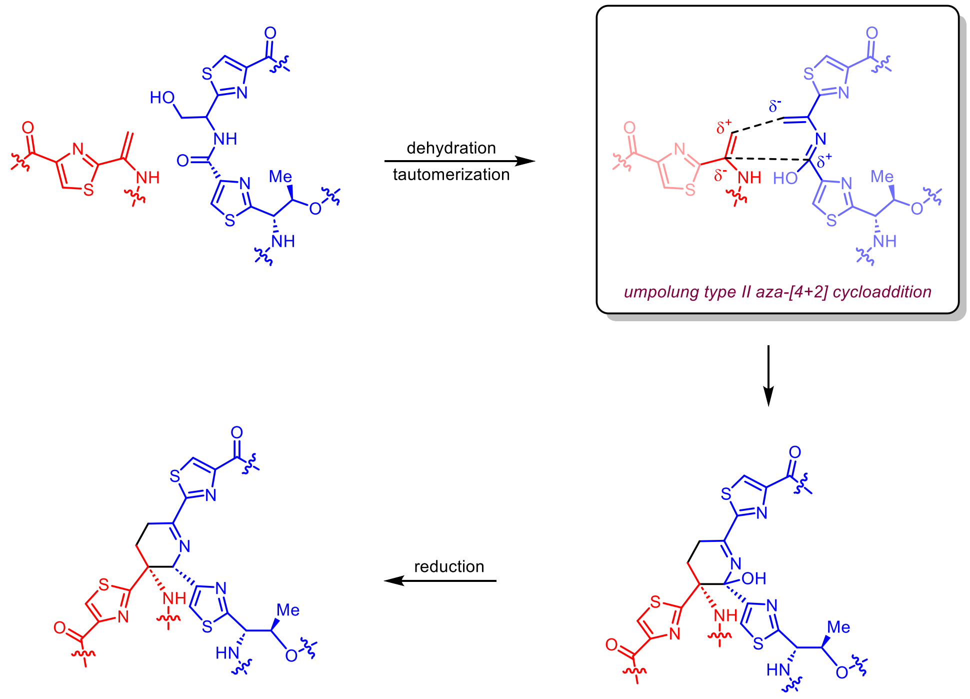

a) Structure of thiostrepton. b) Aza-[4+2] cycloaddition reaction in biosynthetic pathway of thiostrepton.

Table S1 Optimization of iridium-catalyzed C-8 C-H borylation reaction of **7a**[a].

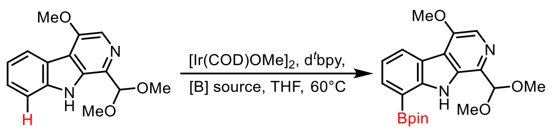

entry	[B] source	[B] equiv	yield[b]
1	HBpin	1.5	51%
2	HBpin	5	10%
3	B_2pin_2	1.5	78%

[a] Reactions were performed with **7a** (0.5 mmol, 1.0 eq), $[Ir(COD)OMe]_2$ (0.0125 mmol, 2.5% eq), d^{t}bpy (0.025 mmol, 5% eq), and boron source in tetrahydrofuran (2.5 mL), heated to 60℃ for 16 h.

[b] Isolation yields.

Table S2 Comparison of synthetic and literature-reported[14] ^{1}H-NMR data of dehydrocrenatine (**5a**).

**Reported** (400 MHz, Chloroform-*d*) (ppm)	**Synthetic** (400 MHz, Chloroform-*d*) (ppm)	**Δδ** (Synthetic-Reported) (ppm)
8.06 (s)	8.13 (s)	0.07
8.30 (d, 8.0)	8.36 (d, 7.9)	0.06
7.23 (t, 8.0)	7.31 (m)	0.08
7.40 (t, 8.0)	7.49 (m)	0.09
7.48 (d, 8.0)	7.48 (m)	0.00
9.65 (br s)	10.19 (s)	0.54
7.15 (dd, 17.5, 11.0)	7.24 (dd, 17.6, 11.2)	0.09
6.22 (dd, 17.5, 2.0)	6.29 (d, 17.5)	0.07
5.48 (dd, 11.0, 2.0)	5.49 (d, 11.2)	0.01
4.10 (s)	4.13 (s)	0.03

Table S3 Comparison of synthetic and literature-reported[15] ^{13}C-NMR data of dehydrocrenatine (**5a**).

**Reported** (101 MHz, Chloroform-*d*) (ppm)	**Synthetic** (101 MHz, Chloroform-*d*) (ppm)	**Δδ** (Synthetic-Reported) (ppm)
151.67	151.64	-0.03
139.07	140.00	0.93
134.79	135.11	0.32
133.14	134.15	1.01
128.36	132.66	4.30
127.55	127.53	-0.02
124.20	124.18	-0.02
121.42	121.18	-0.24
120.65	121.12	0.47
120.46	120.38	-0.08
118.73	118.84	0.11
116.92	117.11	0.19
111.31	111.33	0.02
56.08	56.22	0.14

Table S4 Comparison of synthetic and literature-reported[14] ^{1}H-NMR data of dehydrocrenatidine (**5c**).

**Reported** (400 MHz, Chloroform-*d*) (ppm)	**Synthetic** (400 MHz, Chloroform-*d*) (ppm)	**Δδ** (Synthetic-Reported) (ppm)
8.03 (s)	8.13 (s)	0.10
7.86 (d, 8.0)	7.95 (d, 7.9)	0.09
7.15 (t, 8.0)	7.28 (m)	0.13
6.87 (d, 8.0)	7.00 (d, 7.8)	0.13
9.85 (br s)	8.85 (s)	-1.00
7.15 (dd, 17.5, 11.0)	7.22 (m)	0.07
6.22 (dd, 17.5, 2.0)	6.28 (dd, 17.5, 1.3)	0.06
5.40 (dd, 11.0, 2.0)	5.61 (dd, 11.1, 1.2)	0.21
4.08 (s)	4.19 (s)	0.11
3.92 (s)	4.03 (s)	0.11

Table S5 Comparison of synthetic and literature-reported[15] ^{13}C-NMR data of dehydrocrenatidine (**5c**).

**Reported**<br>(101 MHz, Chloroform-*d*)<br>(ppm)	**Synthetic**<br>(101 MHz, Chloroform-*d*)<br>(ppm)	**Δδ**<br>(Synthetic-Reported)<br>(ppm)
151.32	151.41	0.09
145.81	145.87	0.06
134.44	134.40	-0.04
134.29	134.35	0.06
132.83	133.05	0.22
130.02	129.99	-0.03
122.08	122.19	0.11
121.39	121.66	0.27
120.81	120.98	0.17
118.96	119.06	0.10
116.65	116.79	0.14
116.26	116.33	0.07
107.13	107.23	0.10
56.01	56.20	0.19
55.39	55.59	0.20

Table S6 Comparison of synthetic and literature-reported[16] ^{1}H-NMR data of picrasidine I (**5d**).

**Reported** (400 MHz, DMSO-d_6) (ppm)	**Synthetic** (400 MHz, DMSO-d_6) (ppm)	**Δδ** (Synthetic-Reported) (ppm)
8.07 (s)	8.08 (s)	0.01
7.65 (dd, 7.7, 1.0)	7.68 (d, 7.3)	0.03
7.06 (t, 7.7, 1.0)	7.07 (t, 7.7)	0.01
6.95 (dd, 7.7, 1.0)	6.98 (dd, 7.7, 1.0)	0.03
11.53 (s)	11.59 (s)	0.06
7.61 (dd, 16.9, 10.6)	7.64 (dd, 15.8, 9.6)	0.03
6.30 (dd, 16.9, 2.2)	6.33 (d, 17.0, 2.5)	0.03
5.40 (dd, 10.6, 2.2)	5.43 (d, 10.7, 2.5)	0.03
4.11 (s)	4.11 (s)	0
10.03 (s)	10.10 (s)	0.07

Table S7 Comparison of synthetic and literature-reported[16] ^{13}C-NMR data of picrasidine I (**5d**).

**Reported** (101 MHz, DMSO-d_6) (ppm)	**Synthetic** (101 MHz, DMSO-d_6) (ppm)	**Δδ** (Synthetic-Reported) (ppm)
150.70	150.84	0.14
143.49	143.70	0.21
134.52	134.67	0.15
133.35	133.44	0.09
132.00	131.98	-0.02
129.69	129.82	0.13
121.68	121.80	0.12
120.99	120.99	0
120.31	120.49	0.18
117.62	117.70	0.08
115.20	115.39	0.19
114.01	114.14	0.13
111.38	111.48	0.10
56.07	56.14	0.07

Table S8 Comparison of synthetic and literature-reported[17] ^{1}H-NMR data of picrasidine G (**1**).

**Reported** (400 MHz, DMSO-d_6) (ppm)	**Synthetic** (600 MHz, DMSO-d_6) (ppm)	**Δδ** (Synthetic-Reported) (ppm)
3.60 (ddd, 18.0, 10.0, 8.0)	3.58 (ddd, 19.1, 10.4, 7.5)	-0.02
3.79 (ddd, 18.0, 7.0, 2.0)	3.79 (dd, 19.5, 6.2)	0
1.79 (ddddd, 15.0, 12.0, 10.0, 7.0, 4.0)	1.73 (dtdt, 13.3, 10.3, 6.4, 3.9)	-0.06
2.00 (ddddd, 15.0, 8.0, 3.0, 2.0, 1.0)	1.99 (ddt, 13.8, 7.6, 3.0)	-0.01
2.57 (dddd, 16.0, 4.0, 2.0, 1.0)	2.66 – 2.61 (m)	0.07
2.68 (dddd, 16.0, 12.0, 4.0, 3.0)	2.69 (ddt, 13.6, 8.4, 4.5)	0.01
6.94 (dd, 4.0, 2.0)	6.99 (dd, 4.9, 2.6)	0.05
8.29 (s)	8.38 (s)	0.09
12.29 (s)	13.57 (s)	1.28
8.32 (dd, 8.0, 1.0)	8.31 (d, 7.8)	-0.01
7.45 (td, 8.0, 1.0)	7.45 (ddd, 8.0, 7.1, 1.0)	0
7.77 (td, 8.0, 1.0)	7.77 (ddd, 8.5, 7.1, 1.2)	0
7.86 (dd, 8.0, 1.0)	7.87 (dt, 8.4, 1.0)	0.01
4.01 (s)	4.00 (s)	-0.01
7.82 (s)	7.82 (s)	0
12.03 (s)	12.54 (s)	0.51
7.72 (dd, 8.0, 1.0)	7.73 (dt, 8.3, 1.0)	0.01
7.59 (td, 8.0, 1.0)	7.59 (ddd, 8.3, 7.2, 1.2)	0
7.30 (td, 8.0, 1.0)	7.29 (ddd, 8.0, 7.2, 1.0)	-0.01
8.20 (dd, 8.0, 1.0)	8.20 (d, 7.6)	0
4.04 (s)	4.02 (s)	-0.02

Table S9 Comparison of synthetic and literature-reported[17] ^{13}C-NMR data of picrasidine G (**1**).

**Reported** (100 MHz, DMSO-d_6) (ppm)	**Synthetic** (151 MHz, DMSO-d_6) (ppm)	**Δδ** (Synthetic-Reported) (ppm)
150.51	151.14	0.63
150.05	150.73	0.68
141.71	142.38	0.67
139.30	139.95	0.65
137.36	138.08	0.72
134.26	135.11	0.85
133.52	134.13	0.61
129.68	132.71	3.03
129.61	130.31	0.70
126.91	127.56	0.65
123.65	124.25	0.60
122.67	123.28	0.61
121.23	121.84	0.61
120.62	119.99	-0.63
119.65	119.94	0.29
119.55	119.84	0.29
119.35	119.61	0.26
118.31	118.82	0.51
117.17	117.69	0.52
117.03	117.33	0.30
112.31	112.97	0.66
111.22	111.85	0.63
64.74	64.99	0.25
57.30	57.64	0.34
55.86	56.1	0.24
26.60	26.71	0.11
24.23	24.56	0.33
13.21	13.22	0.01

Table S10 Comparison of synthetic and literature-reported[17] ^{1}H-NMR data of picrasidine S (**2**).

**Reported** (400 MHz, DMSO-d_6) (ppm)	**Synthetic** (600 MHz, DMSO-d_6) (ppm)	**Δδ** (Synthetic-Reported) (ppm)
12.93 (s)	13.06 (s)	0.13
8.18 (s)	8.28 (s)	0.1
7.83 (dd, 8.0, 1.0)	7.86 (d, 7.9, 0.9)	0.03
7.36 (t, 8.0)	7.38 (t, 7.9)	0.02
7.31 (dd, 8.0, 1.0)	7.33 (dd, 8.0, 1.0)	0.02
3.62 (ddd, 18.0, 10.0, 8.0)<br>3.85 (ddd, 18.0, 7.0, 2.0)	3.60 – 3.49 (m)<br>3.84 (dd, 19.3, 6.3)	-0.07<br>-0.01
1.85 (ddddd, 15.0, 12.0, 10.0, 7.0, 4.0)<br>2.23 (ddddd, 15.0, 8.0, 3.0, 2.0, 1.0)	1.74 (dtp, 13.6, 7.1, 3.6)<br>2.03 – 1.92 (m)	-0.11<br>-0.28
2.60 (dddd, 16.0, 4.0, 2.0, 1.0)<br>2.67 (dddd, 16.0, 12.0, 4.0, 3.0)	2.53 (dd, 14.6, 3.4)<br>2.64 (ddt, 14.2, 8.8, 4.4)	-0.07<br>-0.03
7.14 (dd, 4.0, 2.0)	7.04 (dd, 5.1, 2.5)	-0.1
12.13 (s)	12.18 (s)	0.05
7.85 (s)	7.79 (s)	-0.06
7.83 (dd, 8.0, 1.0)	7.79 (d, 7.8)	-0.04
7.25 (t, 8.0)	7.23 (t, 7.8)	-0.02
7.20 (dd, 8.0, 1.0)	7.18 (dd, 8.0, 1.1)	-0.02
4.01 (s)	3.98 (s)	-0.03
4.04 (s)	4.01 (s)	-0.03
4.11 (s)	4.07 (s)	-0.04
4.14 (s)	4.10 (s)	-0.04

Table S11 Comparison of synthetic and literature-reported[17] ^{13}C-NMR data of picrasidine S (**2**).

**Reported** (100 MHz, DMSO-d_6) (ppm)	**Synthetic** (151 MHz, DMSO-d_6) (ppm)	**Δδ** (Synthetic-Reported) (ppm)
150.51	151.09	0.58
149.98	150.76	0.78
145.64	146.37	0.73
145.42	146.10	0.68
138.37	138.96	0.59
134.71	135.65	0.94
133.30	133.94	0.64
132.49	133.01	0.52
132.26	132.64	0.38
129.69	130.11	0.42
121.98	122.78	0.80
120.65	121.09	0.44
120.06	120.75	0.69
119.73	120.14	0.41
119.64	120.09	0.45
119.57	119.86	0.29
117.67	117.86	0.19
116.83	117.60	0.77
115.27	115.90	0.63
114.90	115.45	0.55
109.52	109.94	0.42
107.60	107.87	0.27
64.54	64.92	0.38
57.24	57.65	0.41
55.85	56.09	0.24
55.64	55.87	0.23
55.42	55.63	0.21
27.01	27.05	0.04
24.40	24.57	0.17
13.22	13.12	-0.10

Table S12 Comparison of synthetic and literature-reported[18] ^{1}H-NMR data of picrasidine T (**3**).

**Reported** (400 MHz, DMSO-d_6) (ppm)	**Synthetic** (600 MHz, DMSO-d_6) (ppm)	**Δδ** (Synthetic-Reported) (ppm)
12.83 (s)	13.00 (s)	0.17
11.84 (s)	12.17 (s)	0.33
10.29 (s)	10.68 (s)	0.39
9.96 (s)	10.30 (s)	0.34
8.08 (s)	8.24 (s)	0.16
7.80 (s)	7.78 (s)	0.08
7.77 (dd, 8.0, 1.0)	7.75 (d, 7.9)	-0.02
7.69 (dd, 8.0, 1.0)	7.67 (d, 7.7)	-0.02
7.25 (t, 8.0)	7.26 (t, 7.9)	0.01
7.16 (dd, 8.0, 1.0)	7.18 (d, 7.6)	0.02
7.10 (td, 8.0, 1.0)	7.10 (t, 7.8)	0
7.02 (td, 8.0, 1.0)	7.03 (d, 7.7)	0.01
6.94 (dd, 4.0, 2.0)	6.98 – 6.92 (m)	0.01
4.04 (s)	4.01 (s)	-0.03
3.98 (s)	3.97 (s)	-0.01
3.79 (ddd, 18.0, 7.0, 2.0)	3.79 (dd, 19.2, 6.1)	0
3.59 (ddd, 18.0, 10.0, 8.0)	3.54 (ddd, 18.6, 10.3, 7.3)	-0.05
2.65 (dddd, 16.0, 120, 4.0, 3.0)	2.65 (td, 12.0, 10.1, 7.2)	0
2.60 (dddd, 16.0, 4.0, 2.0, 1.0)	2.59 (d, 12.6)	-0.01
1.99 (ddddd, 15.0, 8.0, 3.0, 2.0, 1.0)	2.01 – 1.91 (m)	-0.03
1.85 (ddddd, 15.0, 12.0, 10.0, 7.0, 4.0)	1.77 – 1.65 (m)	-0.13

Table S13 Comparison of synthetic and literature-reported[19] ^{1}H-NMR data of picrasidine R (**4**).

**Reported**<br>(400 MHz, Chloroform-*d*)<br>(ppm)	**Synthetic**<br>(400 MHz, Chloroform-*d*)<br>(ppm)	**Δδ**<br>(Synthetic-Reported)<br>(ppm)
8.21 (s)	8.19 (s)	-0.02
7.89 (d, 8)	7.88 (d, 7.9)	-0.01
7.23 (t, 8)	7.23 (t, 8.0)	0
6.98 (d, 8)	6.97 (d, 7.9)	-0.01
10.38 (s)	10.38 (s)	0
3.91 (s)	3.90 (s)	-0.01
4.26 (s)	4.25 (s)	-0.01
3.96 (s)	3.96 (s)	0

Table S14 Comparison of synthetic and literature-reported[19] ^{13}C-NMR data of picrasidine R (**4**).

**Reported** (400 MHz, Chloroform-*d*) (ppm)	**Synthetic** (400 MHz, Chloroform-*d*) (ppm)	**Δδ** (Synthetic-Reported) (ppm)
200.8	201.81	1.01
154.0	154.80	0.8
145.5	146.25	0.75
130.8	136.83	6.03
130.3	131.42	1.12
128.2	130.65	2.45
121.0	121.62	0.62
120.7	121.38	0.68
120.6	121.28	0.68
118.4	119.08	0.68
115.5	116.17	0.67
107.2	107.88	0.68
56.1	56.55	0.45
55.3	55.68	0.38
38.6	31.93	-6.67

3. Experimental Procedures and Characterization Data

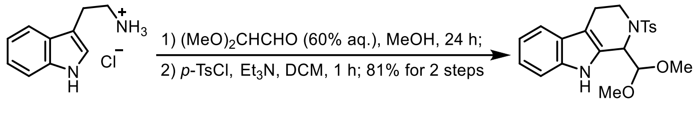

8

Experiment Procedure:

To a suspension of tryptamine hydrochloride salt (30 g, 0.153 mol, 1.0 eq) in methanol (400 mL), 2,2-dimethoxyacetaldehyde (60% aqueous solution, 52 mL, 0.306 mmol, 2.0 eq) was added. The reaction was allowed to stirred at ambient temperature until the reaction was completed (about 24 h). Then methanol and water were removed under vacuum. Saturated sodium bicarbonate aqueous solution (300 mL) and dichloromethane (300 mL) was added to the residues. Then it was extracted by dichloromethane (300 mL × 5). The organic layer was then washed with brines (300 mL), dried over anhydrous sodium sulphate, filtered, and concentrated in vacuo. The residue was dissolved in dichloromethane (400 mL), and cooled to 0°C. To this solution, triethylamine (64 mL, 0.459 mol, 3.0 eq) and *p*-toluenesulfonyl chloride (32 g, 0.168 mol, 1.1 eq) were added. Then the reaction was allowed to warm to room temperature and stirred until the reaction was completed. Saturated sodium bicarbonate aqueous solution (300 mL) was added to the residues. Then it was extracted by dichloromethane (300 mL × 3). The organic layer was then washed with brines (300 mL), dried over anhydrous sodium sulphate, filtered, and concentrated in vacuo. The residue was purified by flash chromatography on silica gel (petroleum ether/dichloromethane = 1:1 to remove excess *p*-toluenesulfonyl chloride, dichloromethane/methanol = 200:1 to elute target product) to afford compound **8** (49.6 g, 81%) as white solid.

Characterization:

^{1}H NMR (400 MHz, Chloroform-*d*) δ 8.44 (s, 1H), 7.72 (m, 1H), 7.70 (m, 1H), 7.37 (d, *J* = 7.8 Hz, 1H), 7.34 (d, *J* = 8.2 Hz, 1H), 7.21 – 7.18 (m, 2H), 7.14 (t, *J* = 7.1 Hz, 1H), 7.05 (t, *J* = 7.5 Hz, 1H), 5.14 (dd, *J* = 3.7, 1.6 Hz, 1H), 4.73 (d, *J* = 3.6 Hz, 1H), 4.20 (ddd, *J* = 14.2, 5.5, 1.2 Hz, 1H), 3.65 (s, 3H), 3.58 (ddd, *J* = 14.3, 12.2, 4.1 Hz, 1H), 3.51 (s, 2H), 2.60 – 2.53 (m, 1H), 2.46 – 2.35 (m, 1H), 2.34 (s, 3H).

^{13}C NMR (101 MHz, Chloroform-*d*) δ 143.51, 138.09, 136.19, 129.83, 128.42, 126.85, 126.45, 122.07, 119.23, 118.16, 111.17, 110.00, 108.51, 57.99, 56.59, 54.21, 41.74, 31.67, 22.74, 21.53, 20.06, 14.22.

HRMS (ESI) $[M + H]^+$ Calculated: 401.1530, Found: 401.1529.

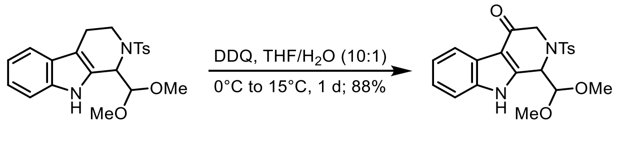

8 9

Experiment Procedure:

A solution of compound **8** (5.64 g, 14 mmol, 1.0 eq) in tetrahydrofuran (280 mL) and water (28 mL) was cooled to 0 °C. 2,3-Dichloro-5,6-dicyano-1,4-benzoquinone (DDQ) (6.36 g, 28 mmol, 2.0 eq) was added at one portion. The deep dark solution was kept at the same temperature for 8 hours, then warmed to 15°C and kept for more than 16 hours. Saturated sodium bicarbonate aqueous solution

(300 mL) was added carefully to the dark red solution. When the carbon dioxide gas stops evolution, tetrahydrofuran was removed under vacuum. The residue was extracted by dichloromethane (300 mL × 5, filtration can be applied if insoluble matters form). The organic layer was then washed with saturated sodium bicarbonate aqueous solution (300 mL × 2) and brines (300 mL), dried over anhydrous sodium sulphate, filtered, and concentrated in vacuo. The residue was purified by flash chromatography on silica gel (dichloromethane/methanol = 200:1, recrystallization by ethyl acetate can be applied to further purification) to afford ketone **9** (5.17 g, 88%) as white solid.

Characterization:

^{1}H NMR (400 MHz, Chloroform-*d*) δ 8.85 (s, 1H), 7.98 (dd, *J* = 8.0, 1.4 Hz, 1H), 7.58 – 7.50 (m, 2H), 7.35 (dd, *J* = 6.8, 1.5 Hz, 1H), 7.22 (pd, *J* = 7.2, 1.5 Hz, 2H), 7.03 (d, *J* = 8.1 Hz, 2H), 5.32 (d, *J* = 4.0 Hz, 1H), 4.79 (d, *J* = 4.1 Hz, 1H), 4.39 (d, *J* = 18.0 Hz, 1H), 4.17 (d, *J* = 18.0 Hz, 1H), 3.62 (s, 3H), 3.44 (s, 3H), 2.15 (s, 3H).

^{13}C NMR (101 MHz, Chloroform-*d*) δ 186.93, 144.32, 144.07, 135.91, 135.45, 129.90, 126.84, 124.20, 123.65, 122.92, 121.61, 112.43, 111.46, 106.58, 57.92, 56.23, 54.57, 51.16, 21.41.

HRMS (ESI) $[M + H]^+$ Calculated: 415.1322, Found: 415.1322.

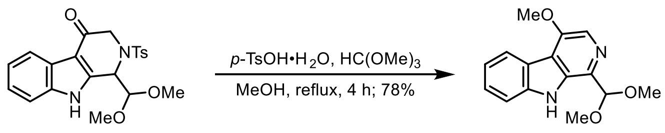

9 **7a**

Experiment Procedure:

A solution of ketone **9** (3.50 g, 8.45 mmol, 1.0 eq) and *p*-toluenesulfonic acid monohydrate (4.82 g, 25.36 mmol, 3.0 eq) in anhydrous methanol (60 mL) and trimethyl orthoformate (60 mL) was heated to reflux (about 60 °C) until the reaction was completed (about 4 hours). Then the reaction was allowed to cool to room temperature, and was carefully added to stirring saturated sodium bicarbonate aqueous solution (200 mL) to quench the acid. When the carbon dioxide gas stops evolution, methanol and trimethyl orthoformate were removed under vacuum. The residue was extracted by dichloromethane (200 mL × 5). The organic layer was then washed with brine (200 mL), dried over anhydrous sodium sulphate, filtered, and concentrated in vacuo. The residue was purified by flash chromatography on silica gel (petroleum ether/dichloromethane = 1:1 to remove methyl *p*-toluenesulfinate, dichloromethane/methanol = 20:1 to elute target product) to afford compound **7a** (1.82 g, 78%) as pale-yellow solid.

Characterization:

^{1}H NMR (400 MHz, Chloroform-*d*) δ 9.15 (s, 1H), 8.33 (dt, *J* = 7.8, 0.9 Hz, 1H), 8.06 (s, 1H), 7.53 – 7.48 (m, 2H), 7.28 (td, *J* = 5.6, 2.7 Hz, 1H), 5.70 (s, 1H), 4.16 (s, 3H), 3.49 (s, 7H).

^{13}C NMR (101 MHz, Chloroform-*d*) δ 152.01, 139.48, 134.72, 133.94, 127.64, 124.19, 120.78, 120.69, 120.23, 118.92, 111.13, 106.44, 56.23, 54.17.

HRMS (ESI) $[M + H]^+$ Calculated: 273.1234, Found: 273.1233.

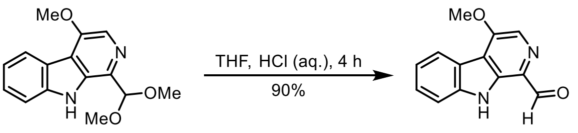

7a **6a**

Experiment Procedure (General Procedure A):

To a solution of compound **7a** (1.20 g, 4.42 mmol, 1.0 eq) in THF (22 mL), 2 N HCl aqueous solution (11 mL, 22 mmol, 5.0 eq) was added. The reaction was allowed to stir at ambient temperature until the reaction was completed (about 4 hours). Then the solution was carefully added to stirring saturated sodium bicarbonate aqueous solution (50 mL) to quench the acid. When the carbon dioxide gas stops evolution, tetrahydrofuran was removed under vacuum. The residue was filtered, dried and was purified by flash chromatography on silica gel (dichloromethane/methanol = 100:1) to afford aldehyde **6a** (900 mg, 90 %) as white solid.

Characterization:

^{1}H NMR (400 MHz, Chloroform-*d*) δ 10.21 (s, 1H), 10.07 (s, 1H), 8.30 (dd, *J* = 7.9, 1.0 Hz, 1H), 8.26 (s, 1H), 7.57 (dd, *J* = 2.6, 1.0 Hz, 1H), 7.56 (d, *J* = 1.0 Hz, 1H), 7.35 (ddd, *J* = 8.1, 5.3, 2.8 Hz, 1H), 4.27 (s, 4H).

^{13}C NMR (101 MHz, Chloroform-*d*) δ 193.98, 154.91, 140.06, 137.11, 131.59, 128.38, 124.25, 123.71, 121.41, 120.11, 118.57, 111.69, 56.71.

HRMS (ESI) $[M + H]^+$ Calculated: 227.0815, Found: 227.0815.

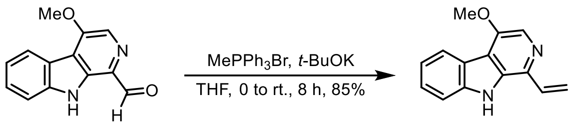

6a **5a**

Experiment Procedure (General Procedure B):

A suspension of methyltriphenylphosphonium bromide (1.10 g, 3.08 mmol, 3.0 eq) in tetrahydrofuran (5 mL) was cooled to 0°C. A solution of potassium *tert*-butoxide (1.0 M tetrahydrofuran solution) (3 mL, 3 mmol, 3.0 eq) was added dropwise. Then the mixture was allowed to warm to room temperature and stirred for another 30 min. During this process, aldehyde **6a** (226 mg, 1mmol, 1.0 eq) was dissolved in tetrahydrofuran (3 mL). The solution was cooled to 0°C. When deprotonation was completed, the nucleophile solution was added dropwise to the electrophile solution. Then the solution was allowed to warm to room temperature and stirred until the reaction was completed (about 8 hours). Saturated sodium bicarbonate aqueous solution (10 mL) was added dropwise to the solution. The mixture was extracted by dichloromethane (10 mL × 5). The organic layer was then washed with brine (20 mL), dried over anhydrous sodium sulphate, filtered, and concentrated in vacuo. The residue was purified by flash chromatography on silica gel (petroleum ether/ethyl acetate = 1:1, recrystallization of hydrochloride salt could be applied if flash chromatography could not fully purification) to afford dehydrocrenatine (**5a)** (192 mg, 85 %) as yellow solid.

Note: For some special substrates, the aldehyde group might partly hydrate. In such case, the crude product can be directly used without purification by chromatography to avoid loss of product.

Characterization:

^{1}H NMR (400 MHz, Chloroform-*d*) δ 10.19 (s, 1H), 8.36 (d, *J* = 7.9 Hz, 1H), 8.13 (s, 1H), 7.55 – 7.43 (m, 2H), 7.31 (t, *J* = 6.9 Hz, 1H), 7.24 (dd, *J* = 17.6, 11.2 Hz, 1H), 6.29 (d, *J* = 17.5 Hz, 1H), 5.49 (d, *J* = 11.2 Hz, 1H), 4.13 (s, 3H).

^{13}C NMR (101 MHz, Chloroform-*d*) δ 151.64, 140.00, 135.11, 134.15, 132.66, 127.53, 124.18, 121.18, 121.12, 120.38, 118.84, 117.11, 111.33, 56.22.

HRMS (ESI) $[M + H]^+$ Calculated: 225.1022, Found: 225.1023.

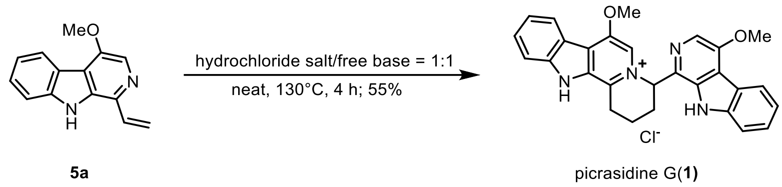

Experiment Procedure (General Procedure C):

Compound **5a** (100 mg, 0.446 mmol, 1.0 eq) was dissolved in dichloromethane (2 mL) and divided into two portions equally. To one portion of the solution (1 mL), hydrochloride acid (4.0 M 1,4-dioxane solution) (112 μL, 0.446 mmol, 2.0 eq) was added. Then the solvent was removed under vacuum (excess hydrochloride should be removed completely). Another portion of the solution was added to the residue, and several drops of methanol was added to fully dissolve the residue. Then the solvent was removed under vacuum again to obtain the equimolar mixture of hydrochloride salt and free base. The mixture was heated to 130°C for 4 hours. Then the mixture was purified by flash chromatography on silica gel (dichloromethane/methanol = 10:1) to afford picrasidine G (**1**) (56 mg, 55%) as white to brown solid.

For gram scale: Compound **5a** (600 mg, 2.68 mmol, 1.0 eq) and its hydrochloride salt (700 mg, 2.68 mmol, 1.0 eq) was dissolved in dichloromethane (10 mL) and methanol (2 mL). Then the solvent was removed under vacuum. The mixture was heated to 130°C for 4 hours. Then the mixture was purified by recrystallization from chloroform to afford picrasidine G (**1**) (920 mg, 71%) as white to brown solid.

Characterization:

^{1}H NMR (600 MHz, DMSO-d_6) δ 13.57 (s, 1H), 12.54 (s, 1H), 8.38 (s, 1H), 8.31 (d, *J* = 7.8 Hz, 1H), 8.20 (d, *J* = 7.6 Hz, 1H), 7.87 (dt, *J* = 8.4, 1.0 Hz, 1H), 7.82 (s, 1H), 7.77 (ddd, *J* = 8.5, 7.1, 1.2 Hz, 1H), 7.73 (dt, *J* = 8.3, 1.0 Hz, 1H), 7.59 (ddd, *J* = 8.3, 7.2, 1.2 Hz, 1H), 7.45 (ddd, *J* = 8.0, 7.1, 1.0 Hz, 1H), 7.29 (ddd, *J* = 8.0, 7.2, 1.0 Hz, 1H), 6.99 (dd, *J* = 4.9, 2.6 Hz, 1H), 4.02 (s, 3H), 4.00 (s, 3H), 3.79 (dd, *J* = 19.5, 6.2 Hz, 1H), 3.58 (ddd, *J* = 19.1, 10.4, 7.5 Hz, 1H), 2.69 (ddt, *J* = 13.6, 8.4, 4.5 Hz, 1H), 2.66 – 2.61 (m, 1H), 1.99 (ddt, *J* = 13.8, 7.6, 3.0 Hz, 1H), 1.73 (dtdt, *J* = 13.3, 10.3, 6.4, 3.9 Hz, 1H).

^{13}C NMR (151 MHz, DMSO-d_6) δ 151.14, 150.73, 142.38, 139.95, 138.08, 135.11, 134.13, 132.71, 130.31, 127.56, 124.25, 123.28, 121.84, 119.99, 119.94, 119.84, 119.61, 118.82, 117.69, 117.33, 112.97, 111.85, 64.99, 57.64, 56.10, 26.71, 24.56, 13.22.

HRMS (ESI) M^+ Calculated: 449.1972, Found: 449.1969.

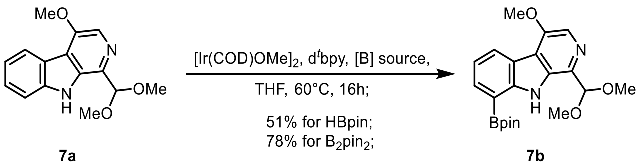

Experiment Procedure:

For pinacol borane as boron source: In a glovebox filled with nitrogen, compound **7a** (136 mg, 0.5 mmol, 1.0 eq), bis-(1,5-cyclooctadiene) dimethoxydiiridium (8 mg, 0.0125 mmol, 2.5% eq) and 4,4-di-*tert*-butyl bipyridine (7 mg, 0.025 mmol, 5% eq) was dissolved in tetrahydrofuran (2.5 mL). To this solution, pinacol borane (96 mg, 0.75 mmol, 1.5 eq) was slowly added. When the hydrogen gas

stops evolution, the reaction was heated to 60°C until the reaction was completed (about 16 h). The reaction was quenched by saturated sodium bicarbonate aqueous solution (10 mL) dropwise. The mixture was extracted by dichloromethane (10 mL × 5). The organic layer was successively washed with 1 N sodium hydroxide solution (50 mL) and brine (50 mL), dried over anhydrous sodium sulphate, filtered, and concentrated in vacuo. The residue was purified by flash chromatography on silica gel (dichloromethane/methanol = 30:1) to afford boronate **7b** (102 mg, 51%) as pale-yellow solid.

For bis(pinacolato)diboron as boron source: In a glovebox filled with nitrogen, compound **7a** (136 mg, 0.5 mmol, 1.0 eq), bis-(1,5-cyclooctadiene) dimethoxydiiridium (8 mg, 0.0125 mmol, 2.5% eq), 4,4-di-*tert*-butyl bipyridine (7 mg, 0.025 mmol, 5% eq) and bis(pinacolato)diboron (190 mg, 0.75 mmol, 1.5 eq) was dissolved in tetrahydrofuran (2.5 mL). Then the reaction was heated to 60°C until the reaction was completed (about 16 h). The reaction was quenched by saturated sodium bicarbonate aqueous solution (10 mL) dropwise. The mixture was extracted by dichloromethane (10 mL × 5). The organic layer was successively washed with 1 N sodium hydroxide solution (50 mL) and brine (50 mL), dried over anhydrous sodium sulphate, filtered, and concentrated in vacuo. The residue was purified by flash chromatography on silica gel (dichloromethane/methanol = 30:1) to afford boronate **7b** (155 mg, 78%) as pale-yellow solid.

Characterization:

^{1}H NMR (400 MHz, Chloroform-*d*) δ 10.27 (s, 1H), 8.48 – 8.31 (m, 1H), 8.08 (s, 1H), 7.94 (dd, *J* = 7.2, 1.3 Hz, 1H), 7.29 (t, *J* = 7.6 Hz, 1H), 5.81 (s, 1H), 4.17 (s, 3H), 3.51 (s, 7H), 1.45 (s, 12H).

^{13}C NMR (151 MHz, Chloroform-*d*) δ 151.91, 145.05, 134.87, 134.49, 133.68, 129.89, 127.59, 126.89, 120.74, 119.63, 119.61, 118.46, 105.35, 84.14, 56.20, 53.41, 25.27.

HRMS (ESI) $[M + H]^+$ Calculated: 399.2086, Found: 399.2086.

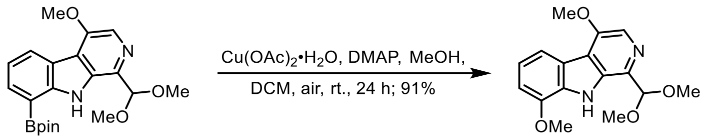

7b **7c**

Experiment Procedure:

Into a solution of boronate **7b** (130 mg, 0.33 mmol, 1.0 eq), cupric acetate monohydrate (78 mg, 0.39 mmol, 1.2 eq) and 4-dimethylaminopyridine (81 mg, 0.66 mmol, 2.0 eq) in anhydrous methanol (1 mL) and dichloromethane (3 mL), dry air was bubbled for 5 min. Then the solution was allowed to stir at room temperature until the reaction was completed (about 24 h). Solvent was removed under vacuum. Saturated sodium bicarbonate aqueous solution (20 mL) and dichloromethane (20 mL) was added to the residues. The mixture was extracted by dichloromethane (20 mL × 3). The organic layer was then washed with brines (20 mL), dried over anhydrous sodium sulphate, filtered, and concentrated in vacuo. The residue was purified by flash chromatography on silica gel (dichloromethane/methanol = 20:1) to afford compound **7c** (91 mg, 91%) as pale-yellow solid.

Characterization:

^{1}H NMR (400 MHz, Chloroform-*d*) δ 9.22 (s, 1H), 8.09 (s, 1H), 7.91 (d, *J* = 7.9 Hz, 1H), 7.21 (t, *J* = 7.9 Hz, 1H), 6.99 (d, *J* = 7.8 Hz, 1H), 5.76 (s, 1H), 4.16 (s, 3H), 4.03 (s, 3H), 3.49 (s, 6H).

^{13}C NMR (101 MHz, Chloroform-*d*) δ 151.99, 145.81, 134.18, 134.11, 130.14, 121.45, 120.66, 120.33, 119.44, 116.24, 107.33, 105.79, 56.19, 55.54, 54.04.

HRMS (ESI) $[M + H]^+$ Calculated: 303.1339, Found: 303.1336

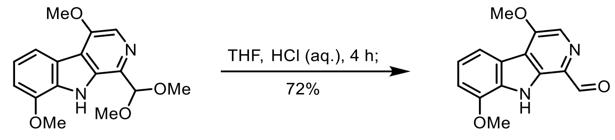

7c **6c**

Aldehyde **6a** was prepared according to general procedure A at 72% yield.

Characterization:

^{1}H NMR (400 MHz, Chloroform-*d*) δ 10.22 (s, 1H), 10.13 (s, 1H), 8.26 (s, 1H), 7.88 (d, *J* = 7.9 Hz, 1H), 7.27 (t, *J* = 7.9 Hz, 1H), 7.03 (dd, *J* = 7.9, 0.8 Hz, 1H), 4.27 (s, 3H), 4.04 (s, 3H).

^{13}C NMR (101 MHz, Chloroform-*d*) δ 193.74, 155.06, 146.27, 136.51, 131.97, 130.66, 123.44, 121.93, 121.12, 119.05, 116.21, 108.23, 77.48, 77.16, 76.84, 56.71, 55.78.

HRMS (ESI) $[M + H]^+$ Calculated: 257.0921, Found: 257.0918.

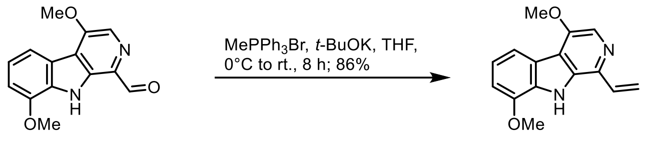

6c dehydrocrenatidine (**5c**)

Dehydrocrenatidine (**5c**) was prepared according to general procedure B at 86% yield.

Characterization:

^{1}H NMR (400 MHz, Chloroform-*d*) δ 8.85 (s, 1H), 8.13 (s, 1H), 7.95 (d, *J* = 7.9 Hz, 1H), 7.34 – 7.23 (m, 1H), 7.25 – 7.16 (m, 1H), 7.00 (d, *J* = 7.8 Hz, 1H), 6.28 (dd, *J* = 17.5, 1.3 Hz, 1H), 5.61 (dd, *J* = 11.1, 1.2 Hz, 1H), 4.19 (s, 3H), 4.03 (s, 3H).

^{13}C NMR (101 MHz, Chloroform-*d*) δ 151.41, 145.87, 134.40, 134.35, 133.05, 129.99, 122.19, 121.66, 120.98, 119.06, 116.79, 116.33, 107.23, 56.20, 55.59.

HRMS (ESI) $[M + H]^+$ Calculated: 255.1128, Found: 255.1125.

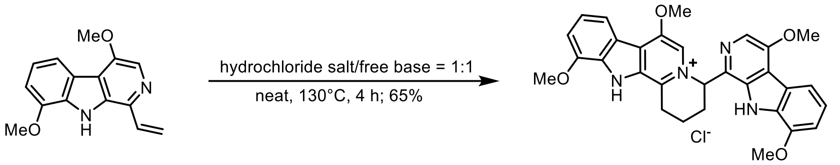

dehydrocrenatidine (**5c**) picrasidine S (**2**)

Picrasidine S (**2**) was prepared according to general procedure C at 65% yield.

Characterization:

^{1}H NMR (600 MHz, DMSO-d_6) δ 13.06 (s, 1H), 12.18 (s, 1H), 8.28 (s, 1H), 7.86 (dd, *J* = 7.9, 0.9 Hz, 1H), 7.79 (d, *J* = 7.8 Hz, 1H), 7.79 (s, 1H) 7.38 (t, *J* = 7.9 Hz, 1H), 7.33 (dd, *J* = 8.0, 1.0 Hz, 1H), 7.23 (t, *J* = 7.8 Hz, 1H), 7.18 (dd, *J* = 8.0, 1.1 Hz, 1H), 7.04 (dd, *J* = 5.1, 2.5 Hz, 1H), 4.10 (s, 3H), 4.07 (s, 3H), 4.01 (s, 3H), 3.98 (s, 3H), 3.84 (dd, *J* = 19.3, 6.3 Hz, 1H), 3.60 – 3.49 (m, 1H), 2.64 (ddt, *J* = 14.2, 8.8, 4.4 Hz, 1H), 2.53 (dd, *J* = 14.6, 3.4 Hz, 1H), 2.03 – 1.92 (m, 1H), 1.74 (dtp, *J* = 13.6, 7.1, 3.6 Hz, 1H).

^{13}C NMR (151 MHz, DMSO-d_6) δ 151.09, 150.76, 146.37, 146.10, 138.96, 135.65, 133.94, 133.01, 132.64, 130.11, 122.78, 121.09, 120.75, 120.14, 120.09, 119.86, 117.86, 117.60, 115.90, 115.45, 109.94, 107.87, 64.92, 57.65, 56.09, 55.87, 55.63, 27.05, 24.57, 13.12.
HRMS (ESI) M^+ Calculated: 509.2183, Found: 509.2174.

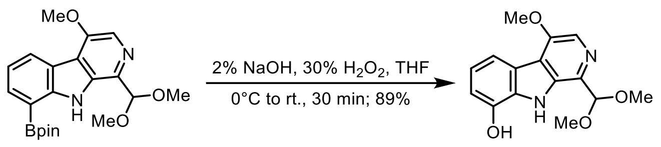

7b **7d**

Experiment Procedure:
A solution of boronate **7b** (1.23g, 3.09 mmol, 1.0 eq) in tetrahydrofuran (30 mL) was cooled to 0°C. To this solution, 0.5 M sodium hydroxide aqueous solution (31 mL, 15.45 mmol, 5.0 eq) and 30% hydrogen peroxide (1.8 g, 15.45 mmol, 5.0 eq) were added. Then the reaction was allowed to warm to room temperature for 30 min until the reaction was completed. Then saturated sodium bicarbonate aqueous solution (20 mL) and saturated sodium bisulfite aqueous solution (30 mL) were added to quench the reaction. The mixture was extracted by dichloromethane (20 mL × 3). The organic layer was then washed with brines (20 mL), dried over anhydrous sodium sulphate, filtered, and concentrated in vacuo. The residue was purified by flash chromatography on silica gel (dichloromethane/methanol = 15:1) to afford compound **7d** (790 mg, 89%) as pale-yellow solid.
Characterization:
^{1}H NMR (400 MHz, DMSO-d_6) δ 10.64 (s, 1H), 9.97 (s, 1H), 8.00 (s, 1H), 7.67 (d, J = 7.8 Hz, 1H), 7.07 (t, J = 7.7 Hz, 1H), 6.92 (d, J = 7.7 Hz, 1H), 5.62 (s, 1H), 4.11 (s, 3H), 3.41 (s, 6H).
^{13}C NMR (101 MHz, DMSO-d_6) δ 151.19, 143.01, 134.51, 133.49, 128.99, 121.39, 120.72, 119.66, 117.86, 114.06, 112.29, 105.30, 56.12, 53.81.
HRMS (ESI) $[M + H]^+$ Calculated: 289.1183, Found: 289.1181.

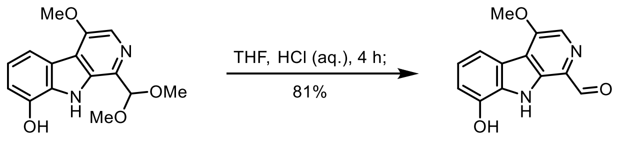

7d **6d**

Aldehyde **6d** was prepared according to general procedure A at 81% yield.
Characterization:
^{1}H NMR (400 MHz, DMSO-d_6) δ 11.54 (s, 1H), 10.13 (s, 1H), 10.00 (s, 1H), 8.38 (s, 1H), 7.71 (dd, J = 7.8, 1.0 Hz, 1H), 7.18 (t, J = 7.8 Hz, 1H), 6.98 (dd, J = 7.8, 1.0 Hz, 1H), 4.26 (s, 3H).
^{13}C NMR (101 MHz, DMSO-d_6) δ 192.82, 154.40, 143.17, 135.32, 131.36, 129.33, 123.49, 122.08, 121.05, 117.86, 114.18, 113.48, 56.93.
HRMS (ESI) $[M + H]^+$ Calculated: 243.0764, Found: 243.0761.

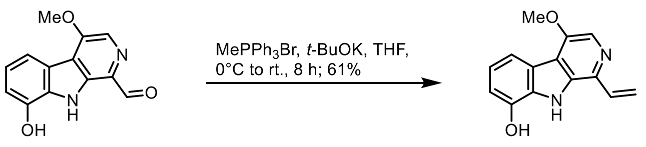

6d picrasidine I (**5d**)

Picrasidine I (**5d**) was prepared according to general procedure B at 61% yield.

Characterization:

^{1}H NMR (400 MHz, DMSO-d_6) δ 11.59 (s, 1H), 10.10 (s, 1H), 8.08 (s, 1H), 7.68 (d, *J* = 7.3 Hz, 1H), 7.64 (dd, *J* = 15.8, 9.6 Hz, 2H), 7.07 (t, *J* = 7.7 Hz, 1H), 6.98 (dd, *J* = 7.7, 1.0 Hz, 1H), 6.33 (dd, *J* = 17.0, 2.5 Hz, 1H), 5.43 (dd, *J* = 10.7, 2.5 Hz, 1H), 4.11 (s, 3H).

^{13}C NMR (101 MHz, DMSO-d_6) δ 150.84, 143.70, 134.67, 133.44, 131.98, 129.82, 121.80, 120.99, 120.49, 117.70, 115.39, 114.14, 111.48, 56.14.

HRMS (ESI) $[M + H]^+$ Calculated: 241.0972, Found: 241.0969.

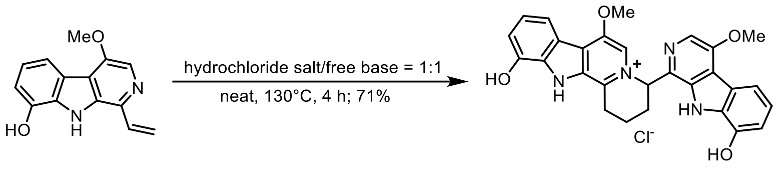

picrasidine I (**5d**) picrasidine T (**3**)

Picrasidine T (**3**) was prepared according to general procedure C at 71% yield.

Characterization:

^{1}H NMR (600 MHz, DMSO-d_6) δ 13.00 (s, 1H), 12.17 (s, 1H), 10.68 (s, 1H), 10.30 (s, 1H), 8.24 (s, 1H), 7.78 (s, 1H), 7.75 (d, *J* = 7.9 Hz, 1H), 7.67 (d, *J* = 7.7 Hz, 1H), 7.26 (t, *J* = 7.9 Hz, 1H), 7.18 (d, *J* = 7.6 Hz, 1H), 7.10 (t, *J* = 7.8 Hz, 1H), 7.03 (d, *J* = 7.7 Hz, 1H), 6.98 – 6.92 (m, 1H), 4.01 (s, 3H), 3.97 (s, 3H), 3.79 (dd, *J* = 19.2, 6.1 Hz, 1H), 3.54 (ddd, *J* = 18.6, 10.3, 7.3 Hz, 1H), 2.65 (td, *J* = 12.0, 10.1, 7.2 Hz, 1H), 2.59 (d, *J* = 12.6 Hz, 1H), 2.01 – 1.91 (m, 1H), 1.77 – 1.65 (m, 1H).

^{13}C NMR (151 MHz, DMSO-d_6) δ 151.16, 150.78, 144.26, 143.80, 138.72, 135.18, 133.86, 132.88, 132.48, 130.07, 122.85, 121.40, 120.89, 120.54, 120.21, 119.21, 118.23, 117.34, 114.50, 113.96, 113.66, 111.75, 64.79, 57.57, 56.12, 26.96, 24.48, 13.26.

HRMS (ESI) M^+ Calculated: 481.1870, Found: 481.1865.

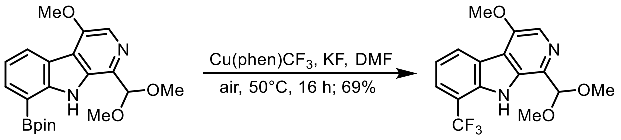

7b **7g**

Experiment Procedure:

Into a solution of boronate **7b** (80 mg, 0.2 mmol, 1.0 eq), (1,10-phenanthroline)(trifluoromethyl) copper (I) (75 mg, 0.24 mmol, 1.2 eq) and potassium fluoride (12 mg, 0.2 mmol, 1.0 eq) in *N*,*N*-dimethylformamide (4 mL), dry air was bubbled for 5 min. Then the solution was heated to 50°C for 16 h. When the reaction was completed, saturated sodium bicarbonate aqueous solution (50 mL) was added. Then the mixture was extracted by dichloromethane (10 mL × 3). The organic layer was then washed with brine (50 mL × 3), dried over anhydrous sodium sulphate, filtered, and concentrated in vacuo. The residue was purified by flash chromatography on silica gel (dichloromethane/methanol = 20:1) to afford compound **7g** (47 mg, 69%) as pale-yellow solid.

Characterization:

^{1}H NMR (400 MHz, Chloroform-*d*) δ 9.50 (s, 1H), 8.49 (d, *J* = 7.9 Hz, 1H), 8.13 (s, 1H), 7.75 (d, *J* = 7.6 Hz, 1H), 7.34 (t, *J* = 7.7 Hz, 1H), 5.74 (s, 1H), 4.18 (s, 3H), 3.50 (s, 6H).

^{13}**C NMR** (101 MHz, Chloroform-*d*) δ 151.78, 135.22 (d, *J* = 1.7 Hz), 134.97, 134.21, 128.08, 124.87 (q, *J* = 271.6 Hz), 124.71 (q, *J* = 4.2 Hz), 122.39, 121.59, 119.67, 118.11, 113.25 (d, *J* = 33.1 Hz), 105.71, 56.32, 53.97.
^{19}**F NMR** (471 MHz, Chloroform-*d*) δ -60.42.
HRMS (ESI) $[M + H]^+$ Calculated: 341.1108, Found: 341.1104.

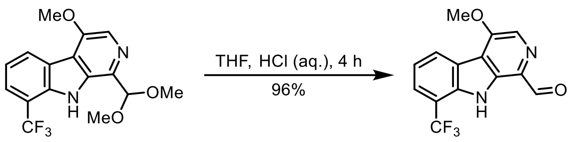

7g **6g**

Aldehyde **6g** was prepared according to general procedure A at 96% yield.
Characterization:
^{1}**H NMR** (400 MHz, Chloroform-*d*) δ 10.28 (s, 1H), 10.21 (s, 1H), 8.46 (d, *J* = 7.9 Hz, 1H), 8.31 (s, 1H), 7.80 (d, *J* = 7.6 Hz, 1H), 7.41 (t, *J* = 7.8 Hz, 1H), 4.29 (s, 3H).
^{13}**C NMR** (101 MHz, Chloroform-*d*) δ 193.73, 154.82, 137.01, 135.84, 131.82, 128.04, 125.33 (q, *J* = 4.4 Hz), 124.51 (q, *J* = 272.7 Hz), 124.21, 121.79, 120.92, 117.77, 114.14 (d, *J* = 33.4 Hz), 56.87.
^{19}**F NMR** (565 MHz, Chloroform-*d*) δ -60.43.
HRMS (ESI) $[M + H]^+$ Calculated: 295.0689, Found: 295.0689.

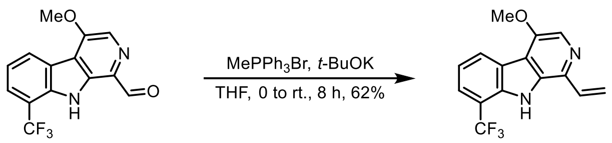

6g **5g**

Compound **5g** was prepared according to general procedure B at 62% yield.
Characterization:
^{1}**H NMR** (400 MHz, Chloroform-*d*) δ 8.62 (s, 1H), 8.50 (d, *J* = 7.9 Hz, 1H), 8.16 (s, 1H), 7.76 (d, *J* = 7.6 Hz, 1H), 7.38 (t, *J* = 7.7 Hz, 1H), 7.15 (dd, *J* = 17.5, 11.1 Hz, 1H), 6.23 (dd, *J* = 17.5, 1.1 Hz, 1H), 5.65 (dd, *J* = 11.1, 1.2 Hz, 1H), 4.19 (s, 3H).
^{13}**C NMR** (101 MHz, Chloroform-*d*) δ 151.12, 135.06, 134.40, 134.10, 132.42, 128.09, 124.77 (q, *J* = 271.3 Hz), 124.56 (q, *J* = 4.3 Hz), 122.98, 122.57, 120.10, 117.80, 117.52, 113.23 (d, *J* = 32.9 Hz), 56.24.
^{19}**F NMR** (471 MHz, Chloroform-*d*) δ -60.24.
HRMS (ESI) $[M + H]^+$ Calculated: 293.0896, Found: 293.0896.

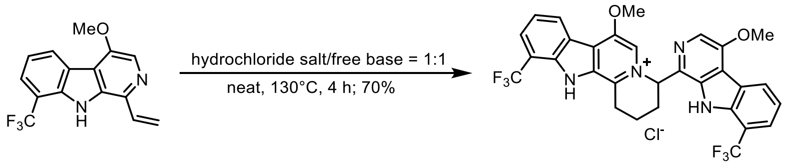

5g **11g**

Compound **11g** was prepared according to general procedure C at 70% yield.
Characterization:

^{1}H NMR (600 MHz, Chloroform-*d*) δ 11.75 (s, 1H), 10.26 (s, 1H), 8.66 (s, 1H), 8.49 (s, 1H), 8.43 (t, *J* = 8.0 Hz, 2H), 7.88 (d, *J* = 7.6 Hz, 1H), 7.78 (s, 1H), 7.71 (d, *J* = 7.7 Hz, 1H), 7.43 (t, *J* = 7.8 Hz, 1H), 7.30 (t, *J* = 7.7 Hz, 1H), 4.07 (s, 3H), 4.06 (s, 3H), 3.88 (dd, *J* = 18.3, 6.3 Hz, 1H), 3.76 – 3.66 (m, 1H), 2.77 – 2.66 (m, 1H), 2.60 – 2.50 (m, 1H), 2.18 – 2.09 (m, 1H), 2.08 – 2.02 (m, 1H).
^{13}C NMR (151 MHz, Chloroform-*d*) δ 151.77, 151.30, 137.38, 137.31, 136.32, 135.69, 134.88, 134.78, 129.35, 127.73, 127.60 (d, *J* = 4.6 Hz), 124.93, 124.20 (q, *J* = 272.2 Hz), 124.03 (q, *J* = 272.0 Hz), 122.90, 121.83, 121.82, 121.07, 121.02, 120.26, 119.69, 118.83, 114.82 (d, *J* = 33.7 Hz), 114.73 (d, *J* = 33.6 Hz), 64.90, 58.08, 56.20, 27.80, 25.30, 13.52.
^{19}F NMR (565 MHz, Chloroform-*d*) δ -60.12, -60.14.
HRMS (ESI) M^+ Calculated: 585.1720, Found: 585.1709.

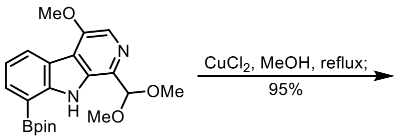

7b **7e**

Experiment Procedure:
To a solution of boronate **7b** (80 mg, 0.2 mmol, 1.0 eq) in anhydrous methanol (4 mL), cupric chloride (80 mg, 0.6 mmol, 3.0 eq) was added. The reaction was heated to reflux until the reaction was completed (about 10 hours). The solvent was removed under vacuum. Saturated sodium bicarbonate aqueous solution (10 mL) and dichloromethane (10 mL) was added to the residues. The mixture was extracted by dichloromethane (10 mL × 3). The organic layer was then washed with brine (10 mL), dried over anhydrous sodium sulphate, filtered, and concentrated in vacuo. The residue was purified by flash chromatography on silica gel (dichloromethane/methanol = 20:1) to afford compound **7e** (58 mg, 95%) as pale-yellow solid.
Characterization:
^{1}H NMR (400 MHz, Chloroform-*d*) δ 9.22 (s, 1H), 8.20 (d, *J* = 7.6 Hz, 1H), 8.09 (s, 1H), 7.50 (dd, *J* = 7.7, 1.0 Hz, 1H), 7.20 (t, *J* = 7.8 Hz, 1H), 5.72 (s, 1H), 4.15 (s, 3H), 3.51 (s, 6H).
^{13}C NMR (101 MHz, Chloroform-*d*) δ 151.90, 136.71, 134.42, 134.32, 126.82, 122.61, 122.11, 121.27, 121.00, 119.17, 116.41, 106.01, 56.25, 54.13.
HRMS (ESI) $[M + H]^+$ Calculated: 307.0844, Found: 307.0843.

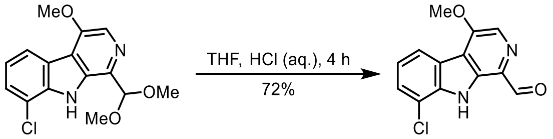

7e **6e**

Aldehyde **6e** was prepared according to general procedure A at 72% yield.
Characterization:
^{1}H NMR (400 MHz, Chloroform-*d*) δ 10.19 (s, 1H), 10.01 (s, 1H), 8.25 (s, 1H), 8.13 (d, *J* = 7.8 Hz, 1H), 7.53 (dd, *J* = 7.8, 1.0 Hz, 1H), 7.26 (t, *J* = 7.8 Hz, 1H), 4.26 (s, 3H).
^{13}C NMR (101 MHz, Chloroform-*d*) δ 193.67, 154.85, 137.31, 136.50, 131.83, 127.59, 123.97, 122.56, 122.17, 121.48, 118.73, 117.09, 56.78.
HRMS (ESI) $[M + H]^+$ Calculated: 261.0425, Found: 261.0422.

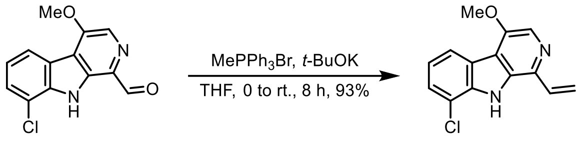

6e **5e**

Compound **5e** was prepared according to general procedure B at 93% yield.

Characterization:

^{1}H NMR (400 MHz, Chloroform-*d*) δ 8.58 (s, 1H), 8.20 (d, *J* = 7.8 Hz, 1H), 8.13 (s, 1H), 7.52 (dd, *J* = 7.8, 1.0 Hz, 1H), 7.26 (d, *J* = 12.1 Hz, 1H), 7.19 (dd, *J* = 17.1, 6.0 Hz, 1H), 6.25 (dd, *J* = 17.5, 1.2 Hz, 1H), 5.63 (dd, *J* = 11.1, 1.2 Hz, 1H), 4.17 (s, 3H).

^{13}C NMR (101 MHz, Chloroform-*d*) δ 151.23, 136.59, 134.17, 134.15, 132.46, 126.68, 122.63, 122.60, 122.24, 121.36, 118.90, 117.35, 116.39, 56.19.

HRMS (ESI) $[M + H]^+$ Calculated: 259.0633, Found: 259.0633.

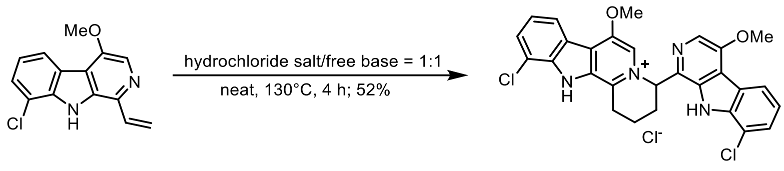

5e **11e**

Compound **11e** was prepared according to general procedure C at 52% yield.

Characterization:

^{1}H NMR (600 MHz, DMSO-*d*$_6$) δ 13.06 (s, 1H), 12.34 (s, 1H), 8.43 (s, 1H), 8.31 (d, *J* = 7.8 Hz, 1H), 8.20 (d, *J* = 7.7 Hz, 1H), 7.90 (d, *J* = 7.7 Hz, 1H), 7.88 (s, 1H), 7.71 (dd, *J* = 7.9, 1.0 Hz, 1H), 7.48 (t, *J* = 7.8 Hz, 1H), 7.33 (t, *J* = 7.8 Hz, 1H), 7.10 (dd, *J* = 5.1, 2.5 Hz, 1H), 4.05 (s, 3H), 4.01 (s, 3H), 3.95 – 3.86 (m, 1H), 3.58 (m, 1H), 2.68 (ddt, *J* = 14.2, 8.6, 3.9 Hz, 1H), 2.61 – 2.54 (m, 1H), 2.03 – 1.95 (m, 1H), 1.73 (ddq, *J* = 13.5, 7.1, 3.6 Hz, 1H).

^{13}C NMR (151 MHz, DMSO-*d*$_6$) δ 151.10, 150.86, 139.53, 139.29, 136.90, 135.66, 134.58, 132.92, 129.93, 127.21, 123.27, 123.13, 122.21, 121.92, 121.28, 120.84, 120.67, 120.31, 118.67, 117.82, 116.99, 116.03, 65.20, 57.91, 56.30, 27.13, 24.89, 12.97.

HRMS (ESI) M^+ Calculated: 517.1193, Found: 517.1186

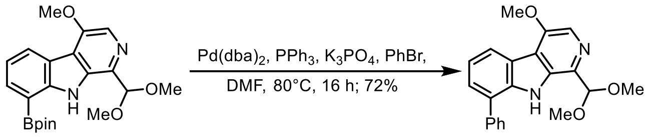

7b **7f**

Experiment Procedure:

A solution of boronate **7b** (80 mg, 0.2 mmol, 1.0 eq), bis(dibenzylideneacetone) palladium (6 mg, 0.01 mmol, 0.05 eq), triphenylphosphine (10 mg, 0.04 mmol, 0.2 eq), anhydrous potassium phosphate (127 mg, 0.6 mmol, 3.0 eq) and phenyl bromide (30 µL, 0.28 mmol, 1.4 eq) in *N*,*N*-dimethylformamide (4 mL) was heated to 80°C until the reaction was completed (about 16 h). When the reaction was completed, saturated sodium bicarbonate aqueous solution (50 mL) was added. Then the mixture was extracted by dichloromethane (10 mL × 3). The organic layer was then washed

with brine (50 mL × 3), dried over anhydrous sodium sulphate, filtered, and concentrated in vacuo. The residue was purified by flash chromatography on silica gel (dichloromethane/methanol = 20:1) to afford compound **7f** (50 mg, 72%) as pale-yellow solid.

Characterization:

^{1}H NMR (400 MHz, Chloroform-*d*) δ 9.41 (s, 1H), 8.33 (d, *J* = 7.8 Hz, 1H), 8.10 (s, 1H), 7.75 – 7.69 (m, 2H), 7.61 – 7.54 (m, 3H), 7.46 (t, *J* = 7.4 Hz, 1H), 7.37 (t, *J* = 7.6 Hz, 1H), 5.71 (s, 1H), 4.18 (s, 3H), 3.45 (s, 6H).

^{13}C NMR (101 MHz, Chloroform-*d*) δ 151.93, 138.79, 137.15, 134.75, 133.83, 129.41, 128.19, 127.80, 127.35, 125.31, 123.35, 121.07, 120.93, 120.75, 119.04, 105.89, 56.22, 53.88.

HRMS (ESI) $[M + H]^+$ Calculated: 349.1547, Found: 349.1544.

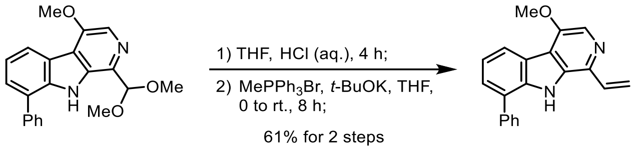

7f **5f**

Compound **5f** was prepared according to general procedure A (without purification) and general procedure B at 61% yield for 2 steps.

Characterization:

^{1}H NMR (400 MHz, Chloroform-*d*) δ 8.57 (s, 1H), 8.33 (dd, *J* = 7.9, 1.2 Hz, 1H), 8.11 (s, 1H), 7.71 – 7.65 (m, 2H), 7.69 (d, *J* = 7.5 Hz, 2H), 7.57 (dd, *J* = 8.4, 6.9 Hz, 2H), 7.54 (dd, *J* = 7.4, 1.2 Hz, 1H), 7.49 – 7.43 (m, 1H), 7.46 (t, *J* = 7.4 Hz, 1H), 7.40 (t, *J* = 7.6 Hz, 1H), 7.09 (dd, *J* = 17.5, 11.1 Hz, 1H), 6.17 (d, *J* = 17.6 Hz, 1H), 5.55 (dd, *J* = 11.1, 1.1 Hz, 1H), 4.18 (s, 3H).

^{13}C NMR (101 MHz, Chloroform-*d*) δ 151.48, 138.59, 137.34, 134.55, 133.79, 132.70, 129.60, 128.36, 128.01, 127.58, 125.65, 123.48, 121.84, 121.70, 121.30, 119.14, 117.09, 56.34.

HRMS (ESI) $[M + H]^+$ Calculated: 301.1335, Found: 301.1336.

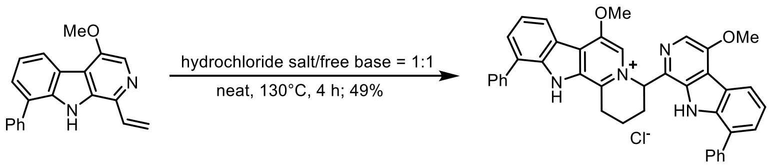

5f **11f**

Compound **11f** was prepared according to general procedure C at 49% yield.

Characterization:

^{1}H NMR (600 MHz, Chloroform-*d*) δ 11.10 (s, 1H), 9.20 (s, 1H), 8.78 (s, 1H), 8.37 (s, 1H), 8.34 – 8.26 (m, 2H), 8.03 – 7.92 (m, 2H), 7.71 (s, 1H), 7.70 – 7.65 (m, 3H), 7.63 – 7.56 (m, 5H), 7.49 (td, *J* = 7.4, 4.2 Hz, 2H), 7.37 (q, *J* = 7.7 Hz, 2H), 4.06 (s, 3H), 4.05 (s, 3H), 3.59 (dd, *J* = 18.3, 6.5 Hz, 1H), 3.39 (dt, *J* = 17.7, 8.5 Hz, 1H), 2.57 (d, *J* = 14.1 Hz, 1H), 2.40 (d, *J* = 14.3 Hz, 1H), 2.09 (s, 1H), 1.92 (s, 1H).

^{13}C NMR (151 MHz, Chloroform-*d*) δ 151.89, 151.51, 139.70, 138.66, 138.08, 137.41, 135.68, 135.57, 134.64, 134.10, 130.42, 129.82, 129.51, 129.38, 128.61, 128.53, 128.43, 127.87, 127.48, 126.79, 124.55, 123.12, 122.83, 122.14, 121.77, 120.93, 120.82, 120.37, 120.02, 119.83, 64.24, 58.12, 56.14, 27.78, 24.70, 13.36.

HRMS (ESI) M^+ Calculated: 601.2598, Found: 601.2605.

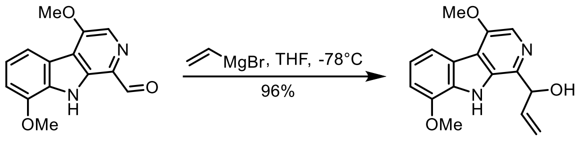

6c 12

Experiment Procedure:

A solution of aldehyde **6c** (14 mg, 0.055 mmol, 1.0 eq) in tetrahydrofuran (1 mL) was cooled to -78°C. To this solution, vinyl magnesium bromide (1.0 M in tetrahydrofuran solution, 270 μL, 0.27 mmol, 5.0 eq) was added. The reaction was kept at this temperature until completed (about 30 min). Then the solution was warmed to room temperature and saturated sodium bicarbonate aqueous solution (2 mL) was added to quench the reaction. The mixture was extracted by dichloromethane (5 mL × 3). The organic layer was then washed with brine (10 mL), dried over anhydrous sodium sulphate, filtered, and concentrated in vacuo. The residue was purified by flash chromatography on silica gel (dichloromethane/methanol = 10:1) to afford alcohol **12** (15 mg, 96%) as pale-yellow solid.

Characterization:

^{1}H NMR (400 MHz, Chloroform-*d*) δ 9.09 (s, 1H), 7.89 (d, *J* = 7.9 Hz, 2H), 7.89 (s, 1H), 7.22 (t, *J* = 7.9 Hz, 1H), 7.00 (d, *J* = 7.8 Hz, 1H), 6.18 (ddd, *J* = 16.9, 10.2, 6.5 Hz, 1H), 5.75 (d, *J* = 6.6 Hz, 1H), 5.63 (d, *J* = 17.1 Hz, 1H), 5.35 (d, *J* = 10.2 Hz, 1H), 4.09 (s, 3H), 4.03 (s, 3H).

^{13}C NMR (101 MHz, Chloroform-*d*) δ 151.97, 146.01, 138.39, 137.05, 133.20, 130.75, 121.54, 121.38, 120.53, 117.69, 117.37, 116.43, 107.84, 73.68, 56.41, 55.72.

HRMS (ESI) $[M + H]^+$ Calculated: 285.1234, Found: 285.1231.

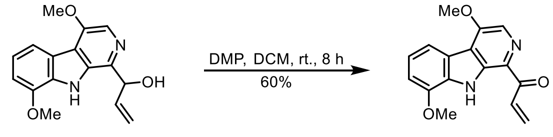

12 10

Experiment Procedure:

To a solution of alcohol **12** (10 mg, 0.035 mmol, 1.0 eq) in dichloromethane (0.8 mL), Dess-Martin periodinane (30 mg, 0.07 mmol, 2.0 eq) was added. Then the reaction was kept at ambient temperature until completed (about 8 h). Saturated sodium bicarbonate aqueous solution (1 mL) and saturated sodium bisulfite aqueous solution (1 mL) was added and stirred for another 2 h. The mixture was extracted by dichloromethane (2 mL × 3). The organic layer was then washed with brine (10 mL), dried over anhydrous sodium sulphate, filtered, and concentrated in vacuo. The residue was purified by flash chromatography on silica gel (dichloromethane/methanol = 100:1) to afford vinyl ketone **10** (6 mg, 60%) as pale-yellow solid.

Characterization:

^{1}H NMR (400 MHz, Chloroform-*d*) δ 10.49 (s, 1H), 8.19 (s, 1H), 8.10 (dd, *J* = 17.5, 10.5 Hz, 1H), 7.88 (d, *J* = 7.9 Hz, 1H), 7.25 (t, *J* = 7.9 Hz, 1H), 7.01 (d, *J* = 7.8 Hz, 1H), 6.63 (dd, *J* = 17.4, 2.1 Hz, 1H), 5.94 (dd, *J* = 10.5, 2.1 Hz, 1H), 4.26 (s, 3H), 4.03 (s, 3H).

^{13}C NMR (101 MHz, Chloroform-*d*) δ 190.27, 154.87, 146.30, 137.77, 131.60, 131.45, 130.58, 128.51, 121.90, 121.60, 121.36, 119.04, 116.20, 108.00, 56.59, 55.77.

HRMS (ESI) $[M + H]^+$ Calculated: 283.1077, Found: 283.1075.

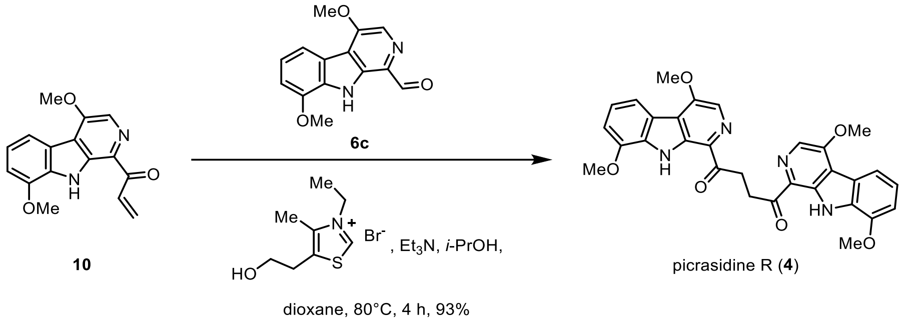

Experiment Procedure:

To a solution of vinyl ketone **10** (5 mg, 0.018 mmol, 1.0 eq), aldehyde **6c** (7 mg, 0.027 mmol, 1.5 eq) and 3-ethyl-5-(2-hydroxyethyl)-4-methylthiazol-3-ium bromide (2.5 mg, 0.009 mmol, 0.5 eq) in 1,4-dioxane (1 mL), isopropanol (5.5 μL, 0.072 mmol, 4.0 eq) and triethylamine (3.5 μL, 0.025 mmol, 2.5 eq) was added. The solution was heated to 80 °C for 4 hours. During reaction, the color of solution was transformed from yellow to green and finally changed backed to yellow again with the precipitation of yellow solid. When the reaction was completed, saturated sodium bicarbonate aqueous solution (5 mL) was added. Then the mixture was extracted by dichloromethane (5 mL × 3). The organic layer was then washed with brine (10 mL × 3), dried over anhydrous sodium sulphate, filtered, and concentrated in vacuo. The residue was purified by flash chromatography on silica gel (dichloromethane/methanol = 20:1) to afford picrasidine R (**4**) (9 mg, 93%) as yellow solid.

Characterization:

^{1}H NMR (400 MHz, Chloroform-*d*) δ 10.38 (s, 1H), 8.19 (s, 1H), 7.88 (d, *J* = 7.9 Hz, 1H), 7.23 (t, *J* = 7.9 Hz, 1H), 6.97 (d, *J* = 7.9 Hz, 1H), 4.25 (s, 3H), 3.96 (s, 3H), 3.90 (s, 2H).

^{13}C NMR (101 MHz, Chloroform-*d*) δ 201.81, 154.80, 146.25, 136.83, 131.42, 130.65, 121.62, 121.38, 121.28, 119.08, 116.17, 107.88, 56.55, 55.68, 31.93.

HRMS (ESI) $[M + H]^+$ Calculated: 539.19251, Found: 539.19210.

4. NMR Spectrums

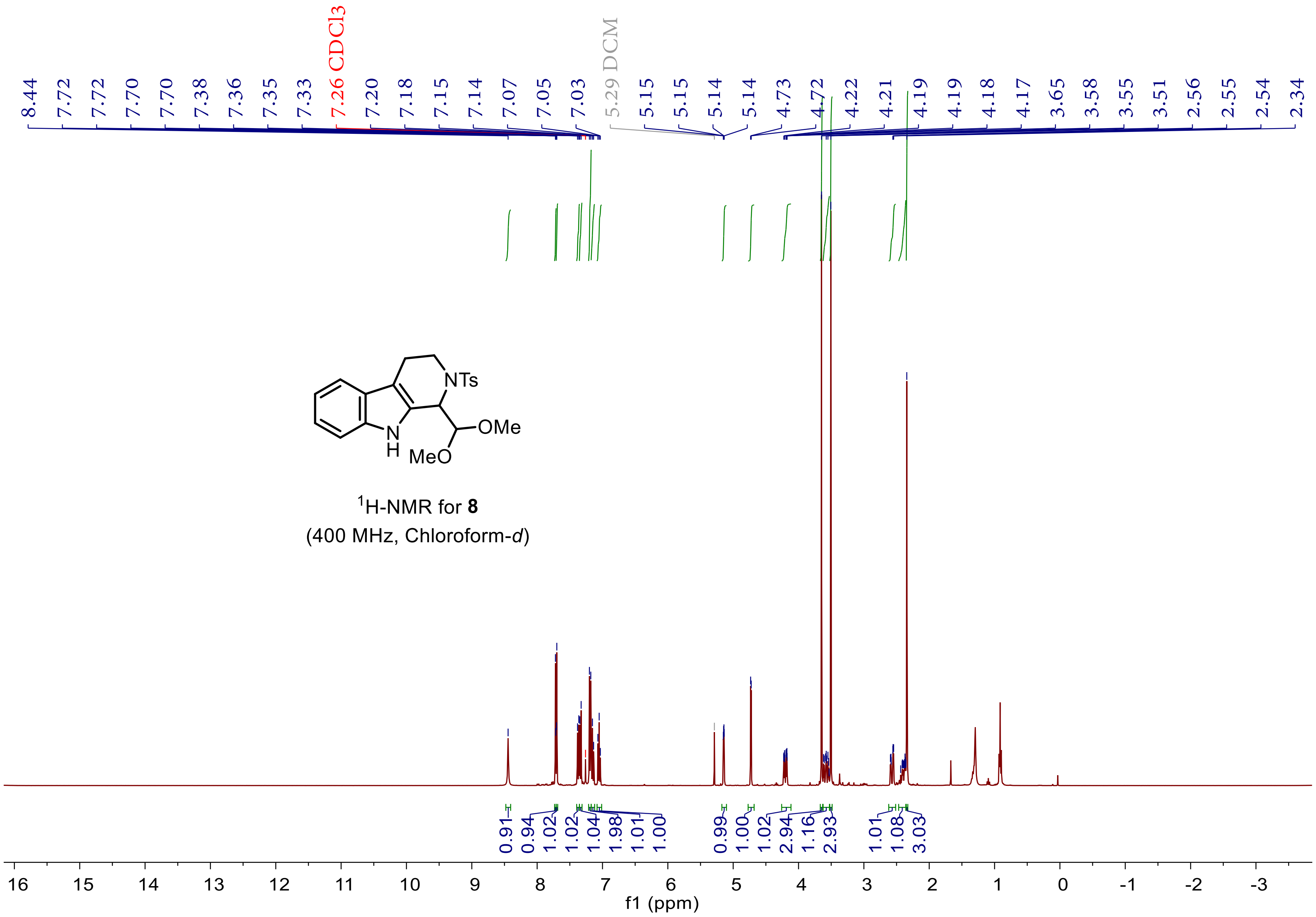

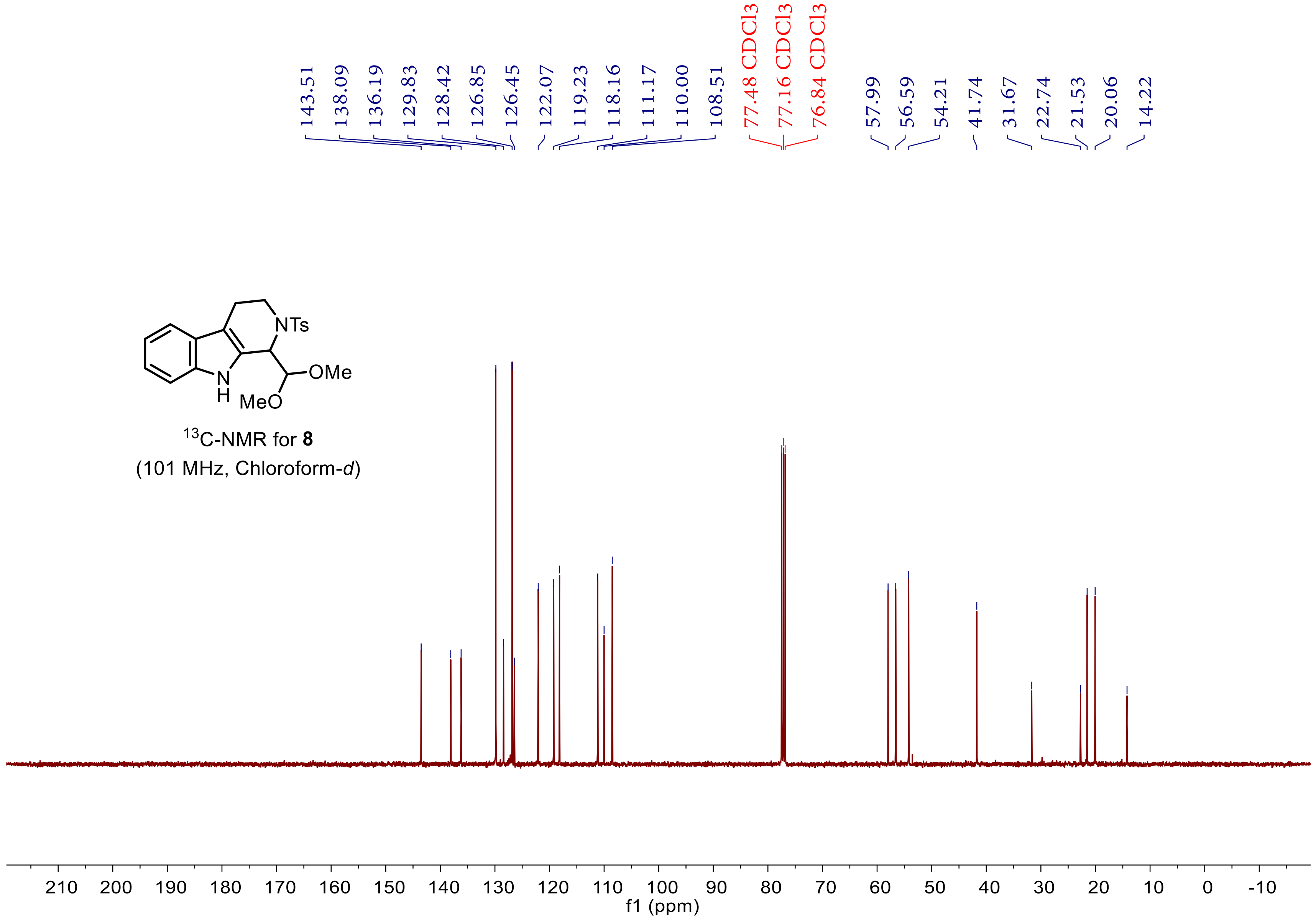
143.51
138.09
136.19
129.83
128.42
126.85
126.45
122.07
119.23
118.16
111.17
110.00
108.51
77.48 CDCl3
77.16 CDCl3
76.84 CDCl3
57.99
56.59
54.21
41.74
31.67
22.74
21.53
20.06
14.22
NTs
OMe
N
H
MeO
13C-NMR for 8
(101 MHz, Chloroform-d)
210 200 190 180 170 160 150 140 130 120 110 100 90 80 70 60 50 40 30 20 10 0 -10
f1 (ppm)

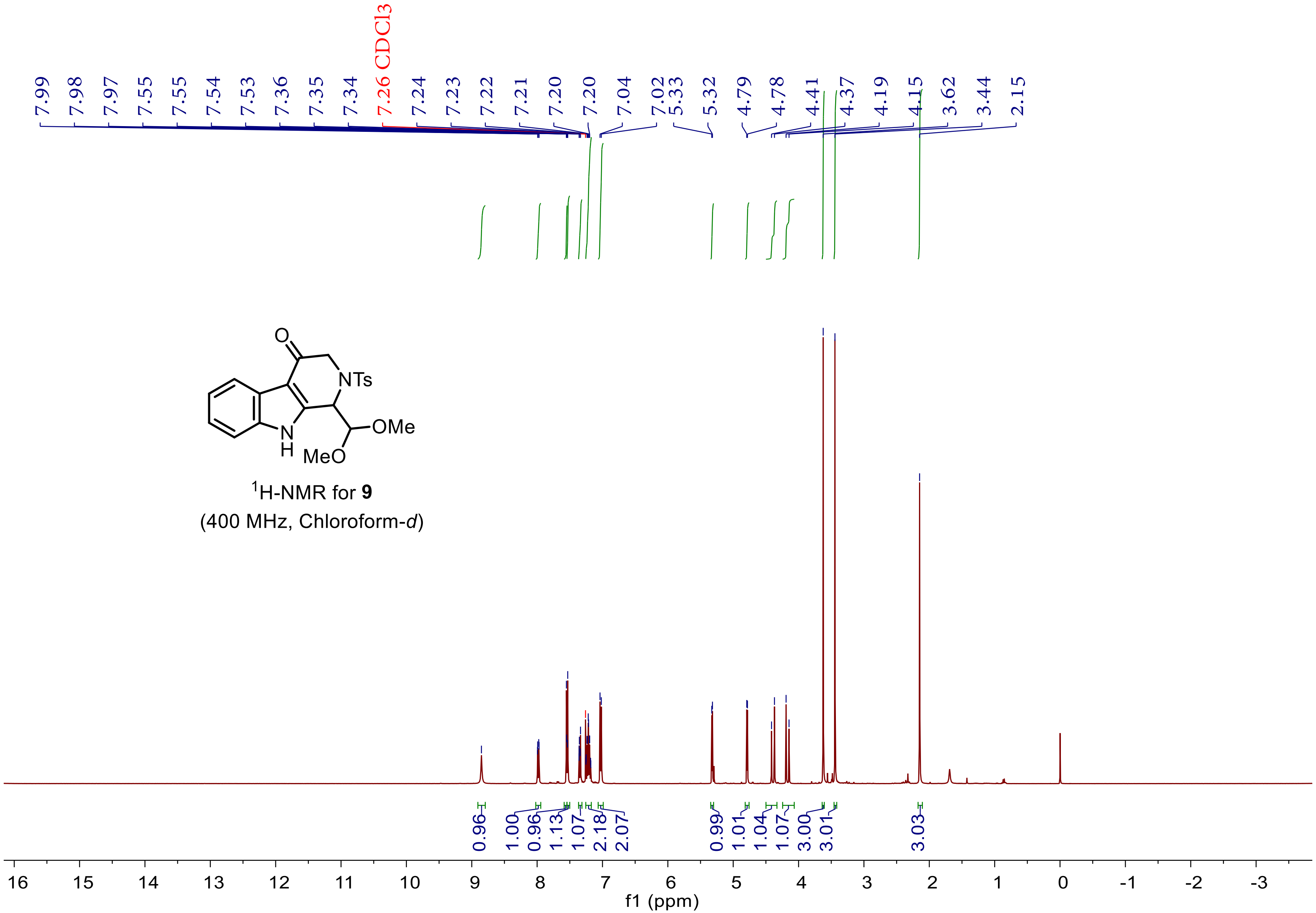
O
NTs
OMe
N
H
MeO
^{1}H-NMR for 9
(400 MHz, Chloroform-d)
7.99 7.98 7.97 7.55 7.55 7.54 7.53 7.36 7.35 7.34 7.26 CDCl3 7.24 7.23 7.22 7.21 7.20 7.20 7.04 7.02 5.33 5.32 4.79 4.78 4.41 4.37 4.19 4.15 3.62 3.44 2.15
0.96 1.00 0.96 1.13 1.07 2.18 2.07 0.99 1.01 1.04 1.07 3.00 3.01 3.03
f1 (ppm)

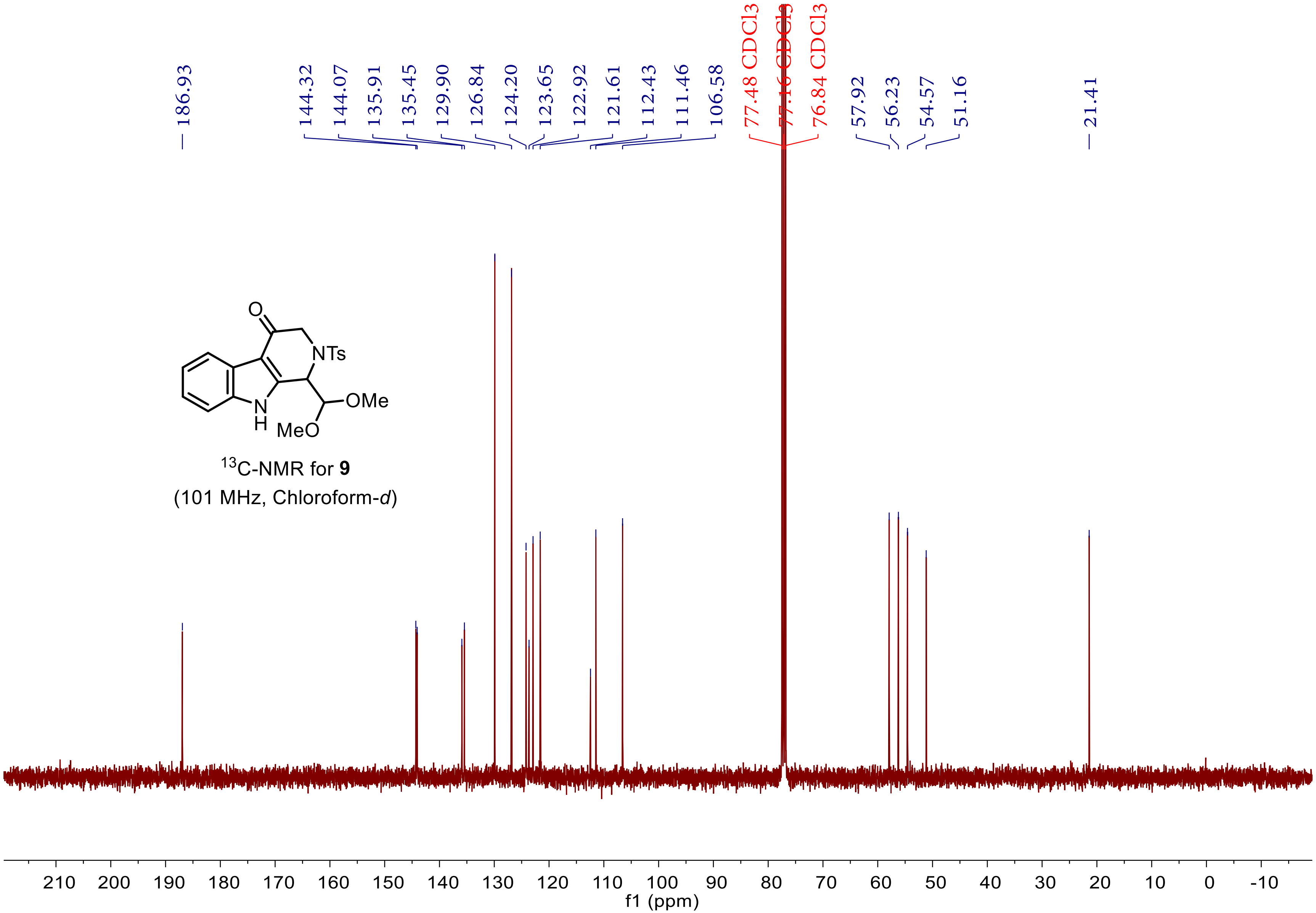

^{13}C-NMR for **9**
(101 MHz, Chloroform-*d*)

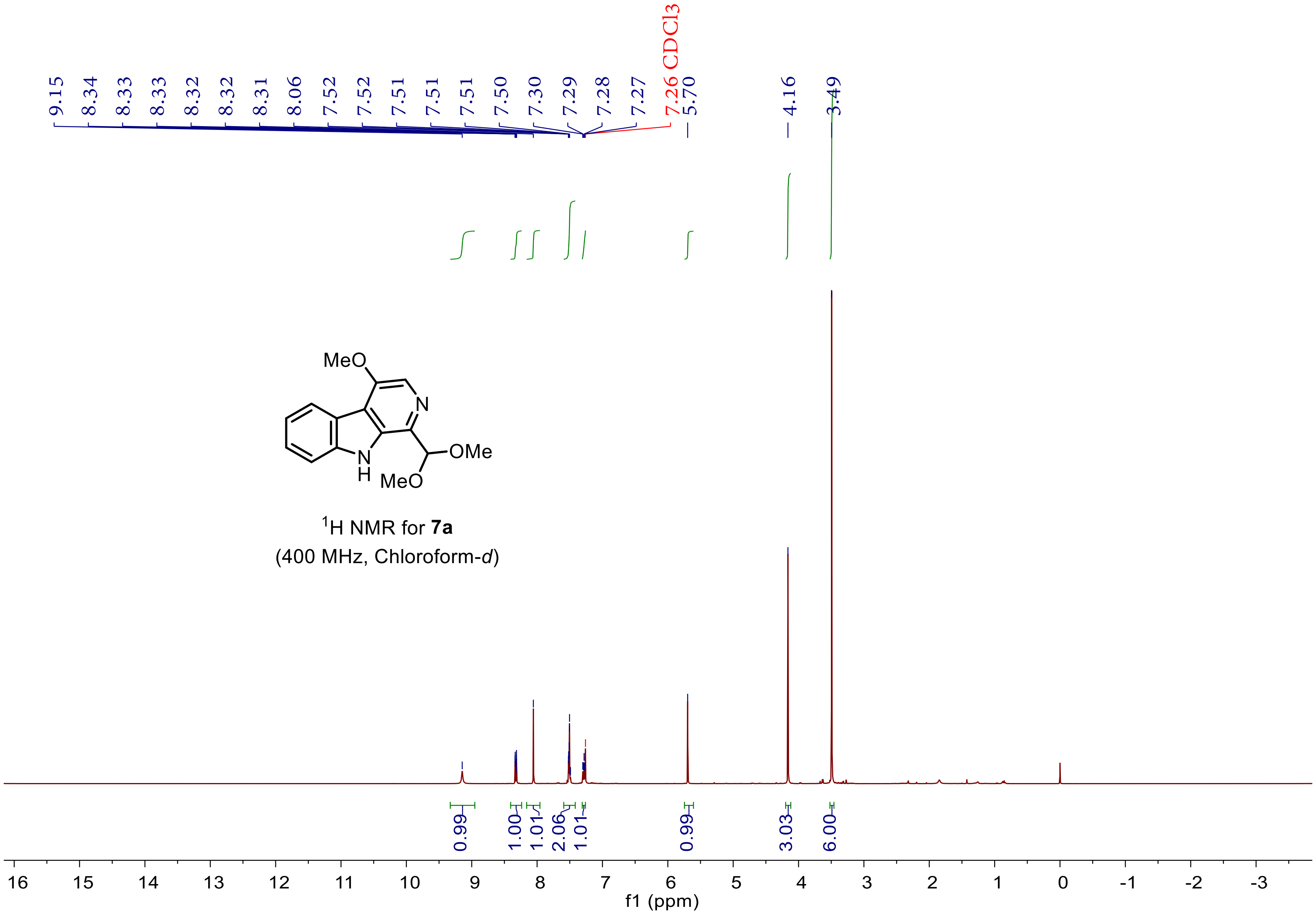

^{1}H NMR for **7a**
(400 MHz, Chloroform-*d*)

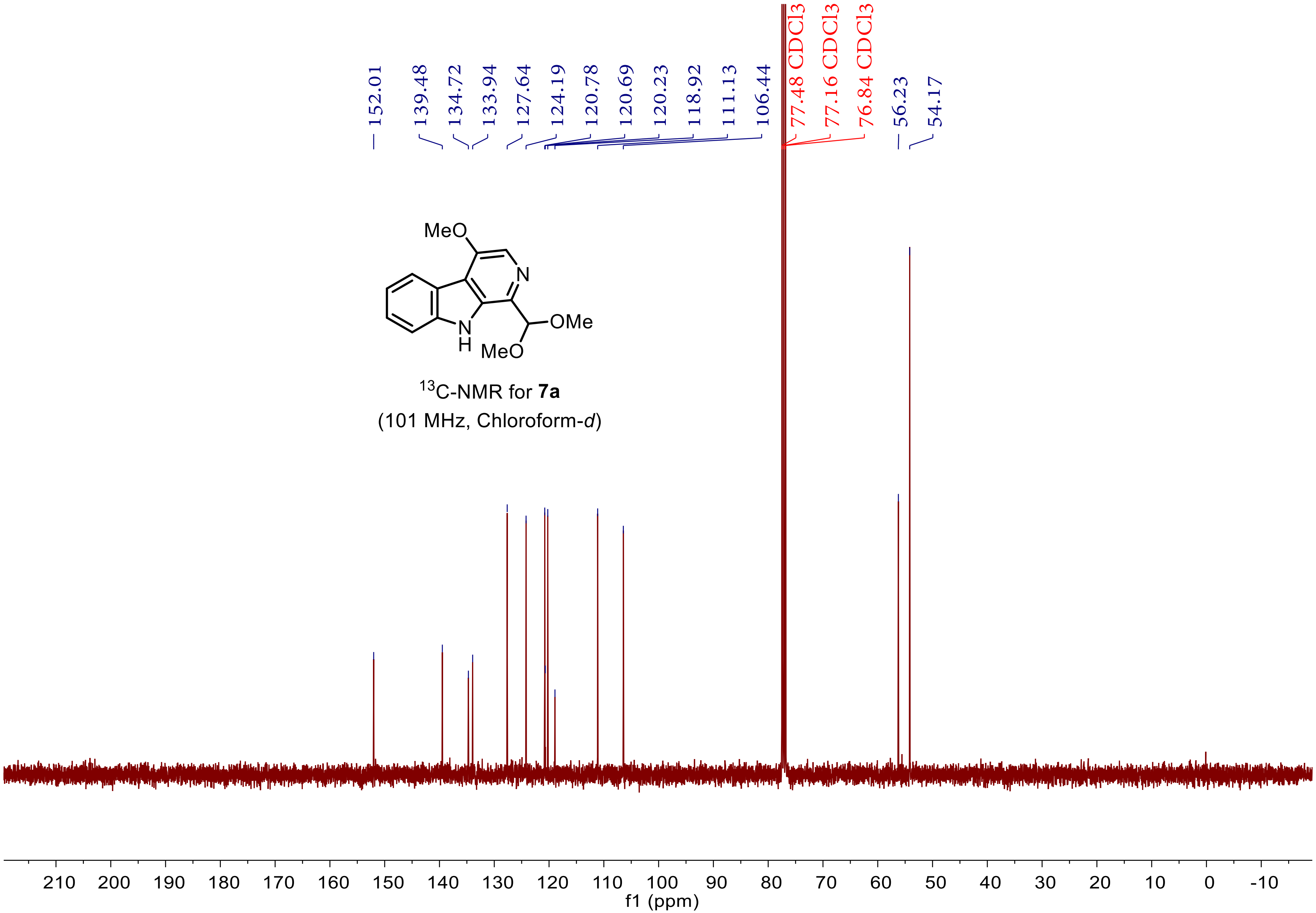

^{13}C-NMR for **7a**

(101 MHz, Chloroform-*d*)

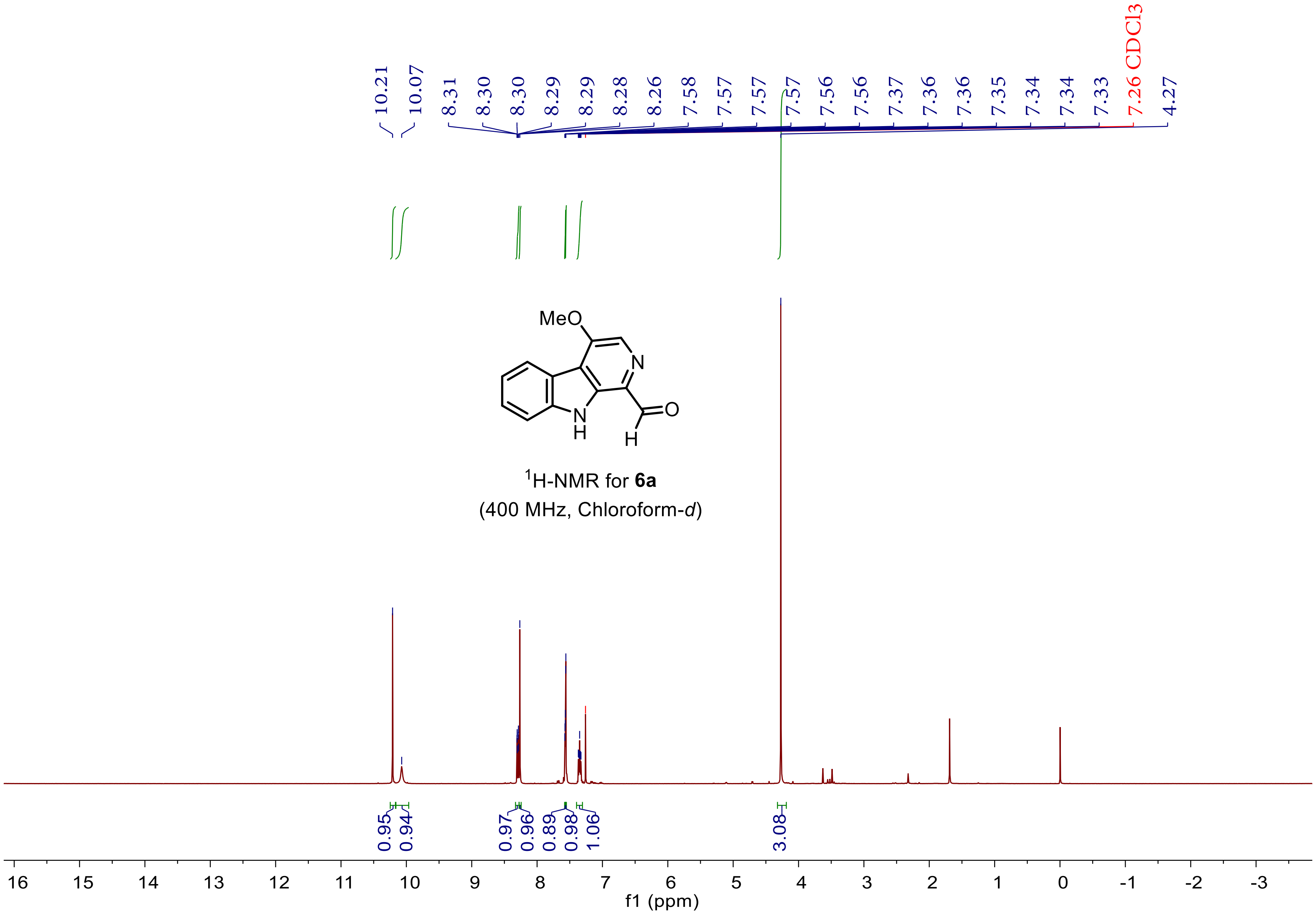

^{1}H-NMR for **6a**
(400 MHz, Chloroform-*d*)

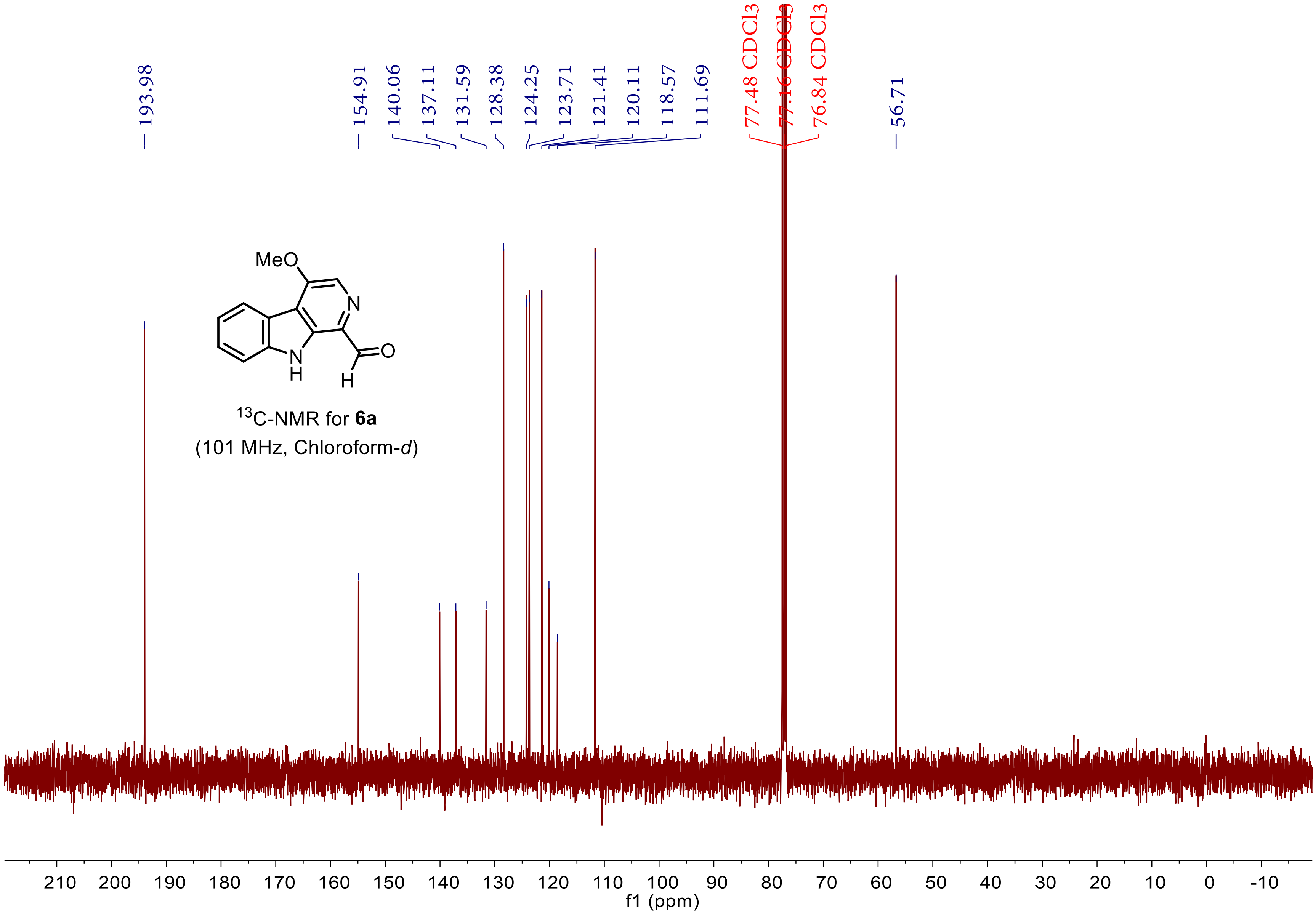
193.98
154.91
140.06
137.11
131.59
128.38
124.25
123.71
121.41
120.11
118.57
111.69
77.48 CDCl3
77.16 CDCl3
76.84 CDCl3
56.71
MeO
N
N
H
H
O
^{13}C-NMR for **6a**
(101 MHz, Chloroform-*d*)
210 200 190 180 170 160 150 140 130 120 110 100 90 80 70 60 50 40 30 20 10 0 -10
f1 (ppm)

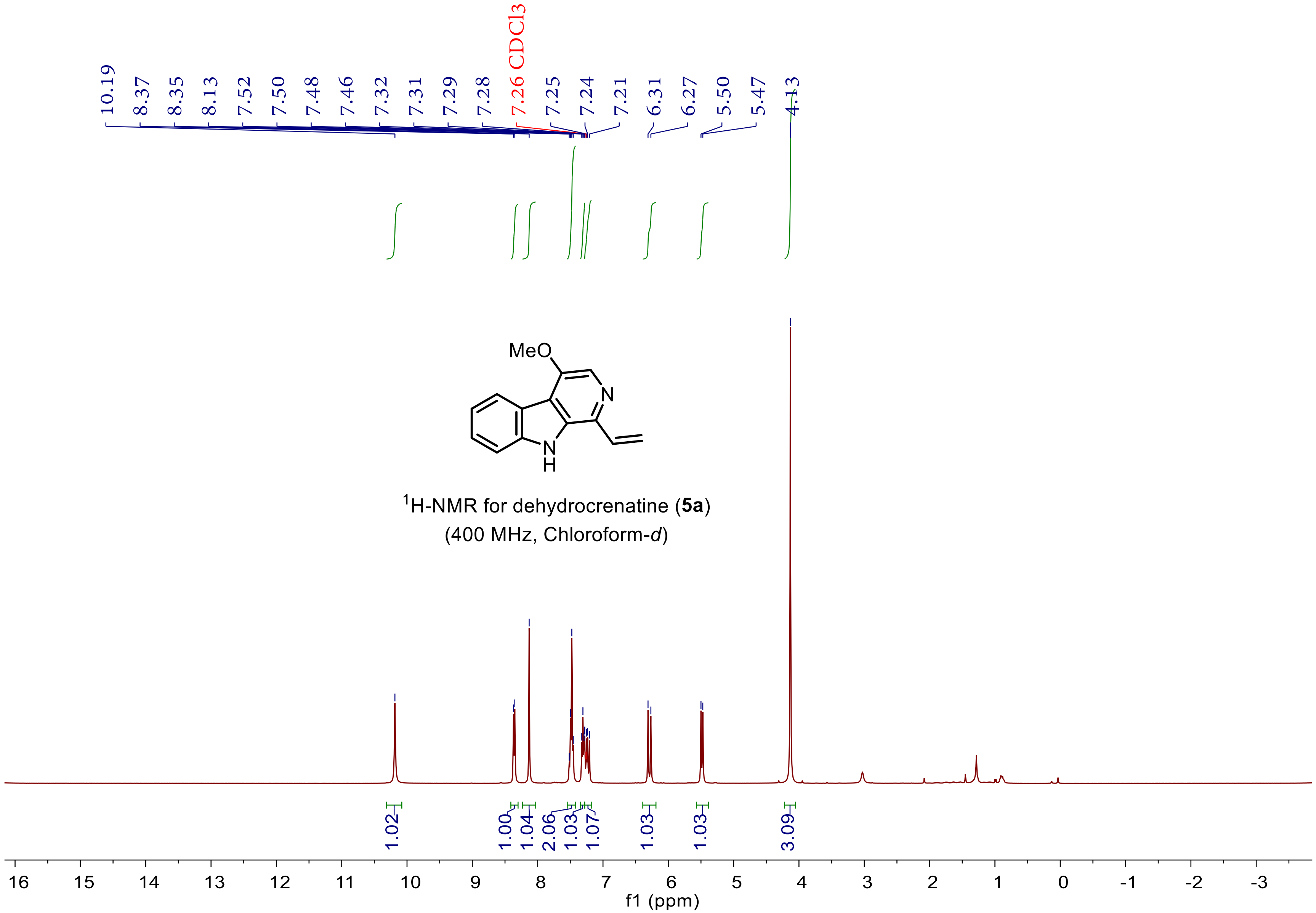
10.19
8.37
8.35
8.13
7.52
7.50
7.48
7.46
7.32
7.31
7.29
7.28
7.26 CDCl3
7.25
7.24
7.21
6.31
6.27
5.50
5.47
4.13
MeO
N
N
H
1H-NMR for dehydrocrenatine (5a)
(400 MHz, Chloroform-d)
1.02
1.00
1.04
2.06
1.03
1.07
1.03
1.03
3.09
16
15
14
13
12
11
10
9
8
7
6
5
4
3
2
1
0
-1
-2
-3
f1 (ppm)

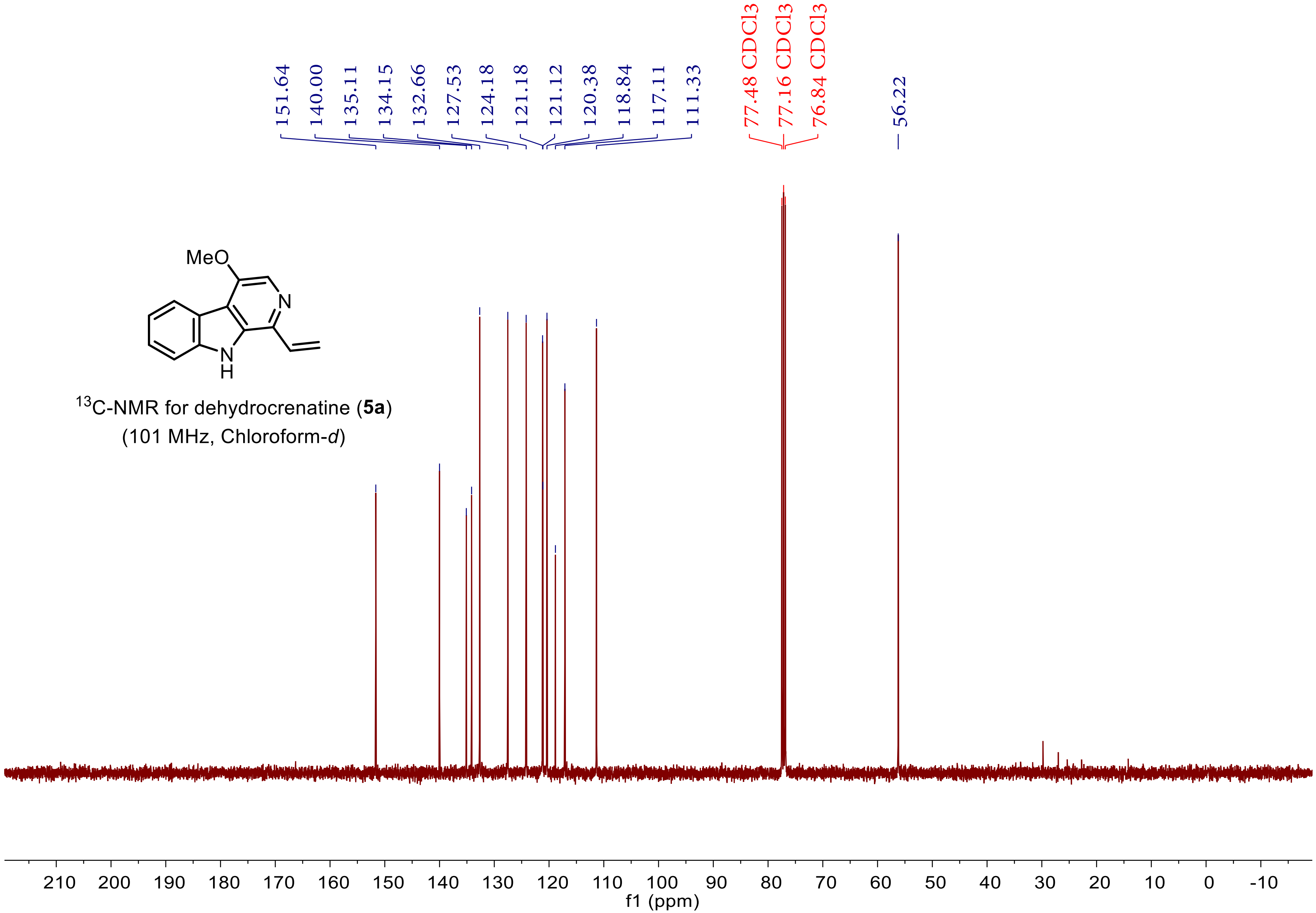

^{13}C-NMR for dehydrocrenatine (**5a**)
(101 MHz, Chloroform-*d*)

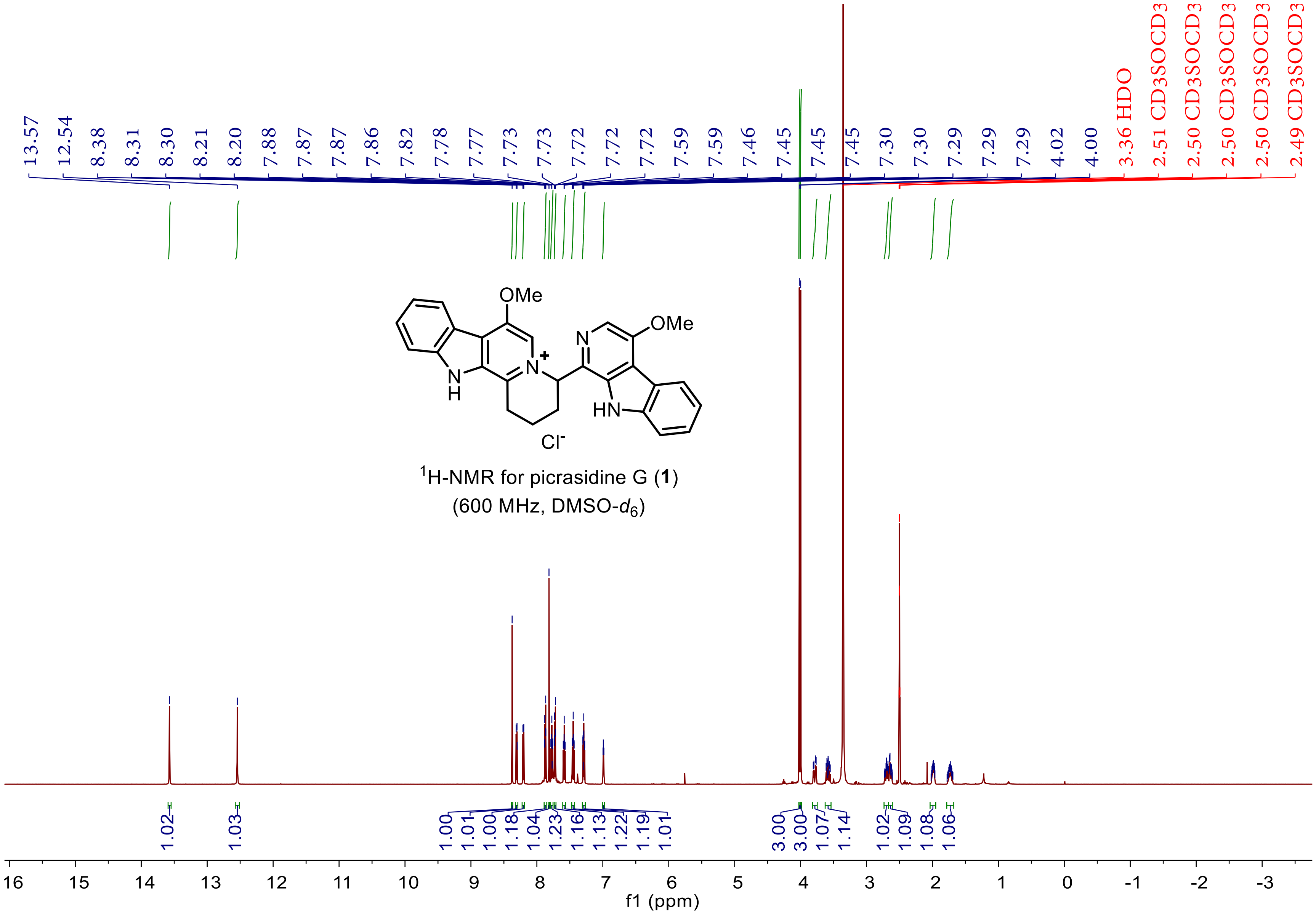

^{1}H-NMR for picrasidine G (**1**)
(600 MHz, DMSO-d_6)

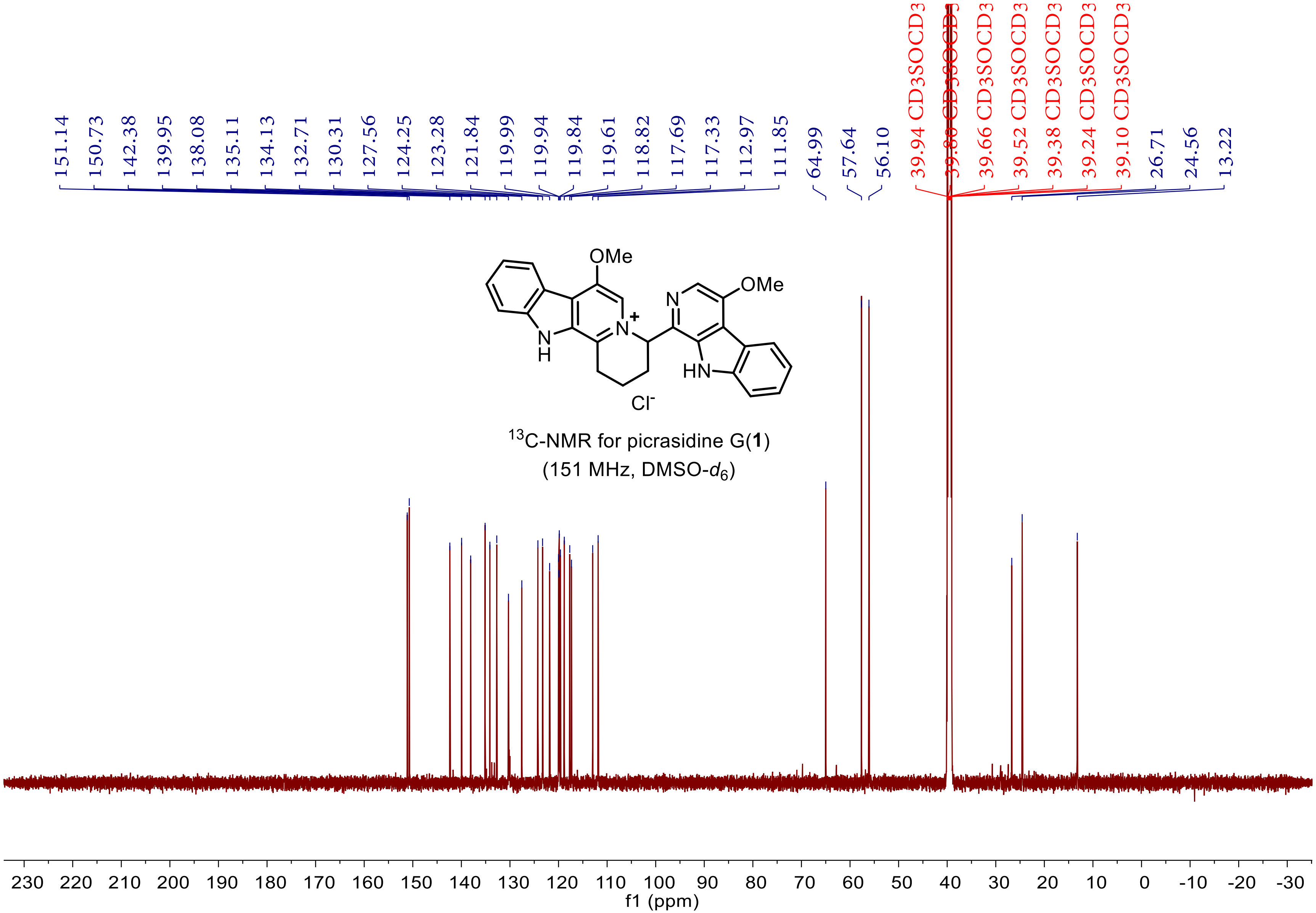

^{13}C-NMR for picrasidine G(**1**)
(151 MHz, DMSO-d_6)

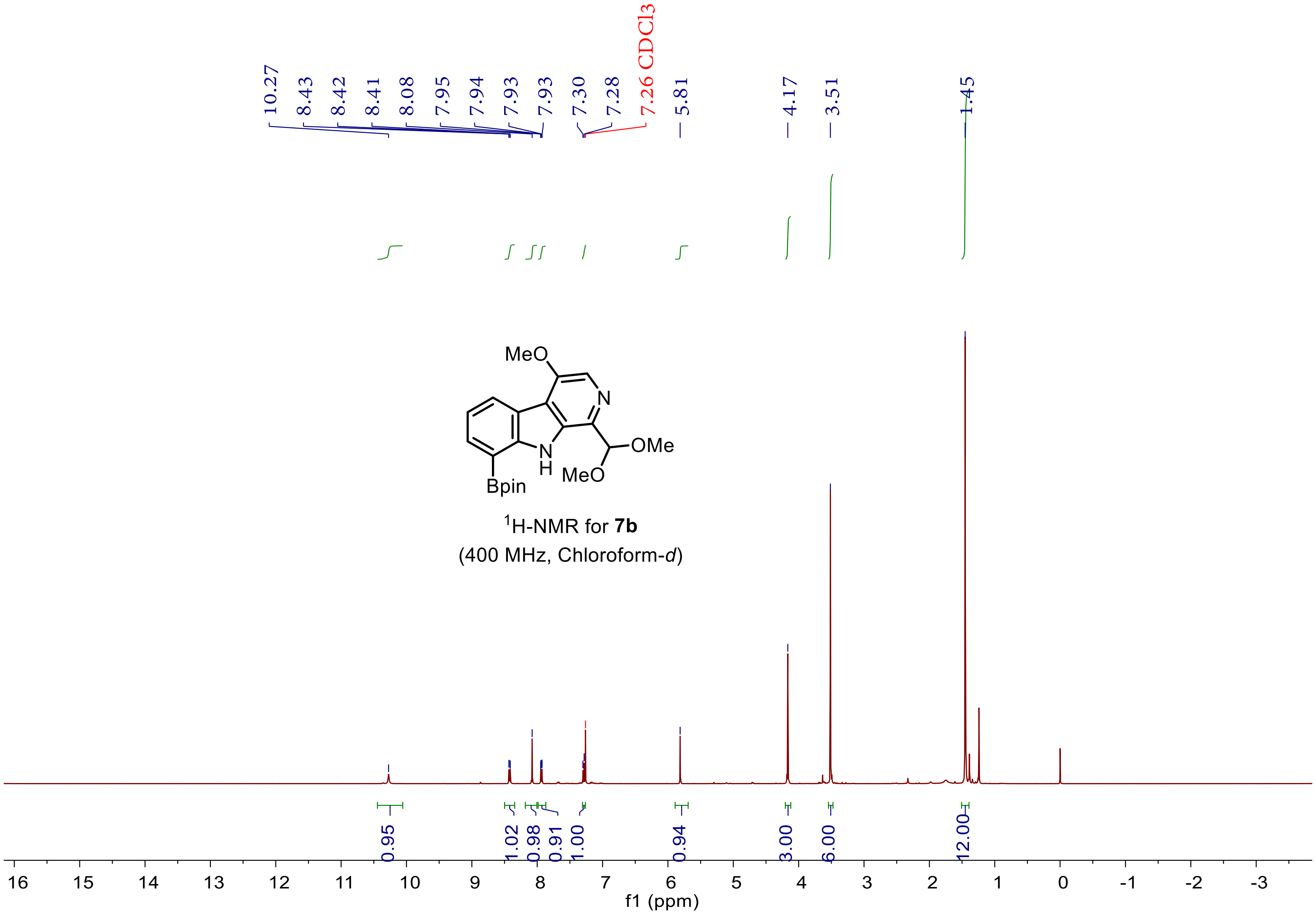
10.27
8.43
8.42
8.41
8.08
7.95
7.94
7.93
7.93
7.30
7.28
7.26 CDCl3
5.81
4.17
3.51
1.45
MeO
N
OMe
N
H
MeO
Bpin
^{1}H-NMR for **7b**
(400 MHz, Chloroform-*d*)
0.95
1.02
0.98
0.91
1.00
0.94
3.00
6.00
12.00
16 15 14 13 12 11 10 9 8 7 6 5 4 3 2 1 0 -1 -2 -3
f1 (ppm)

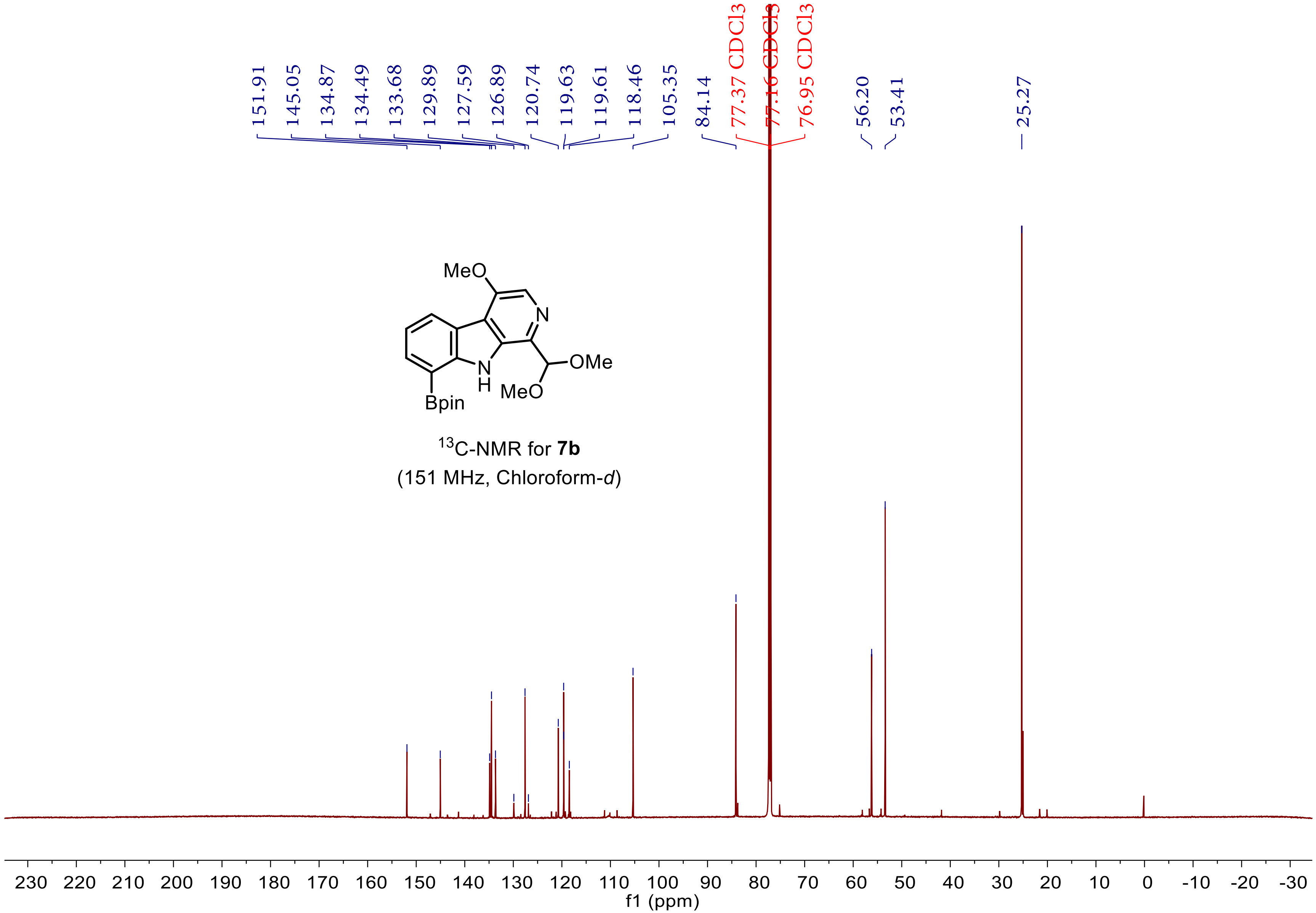

^{13}C-NMR for **7b**
(151 MHz, Chloroform-*d*)

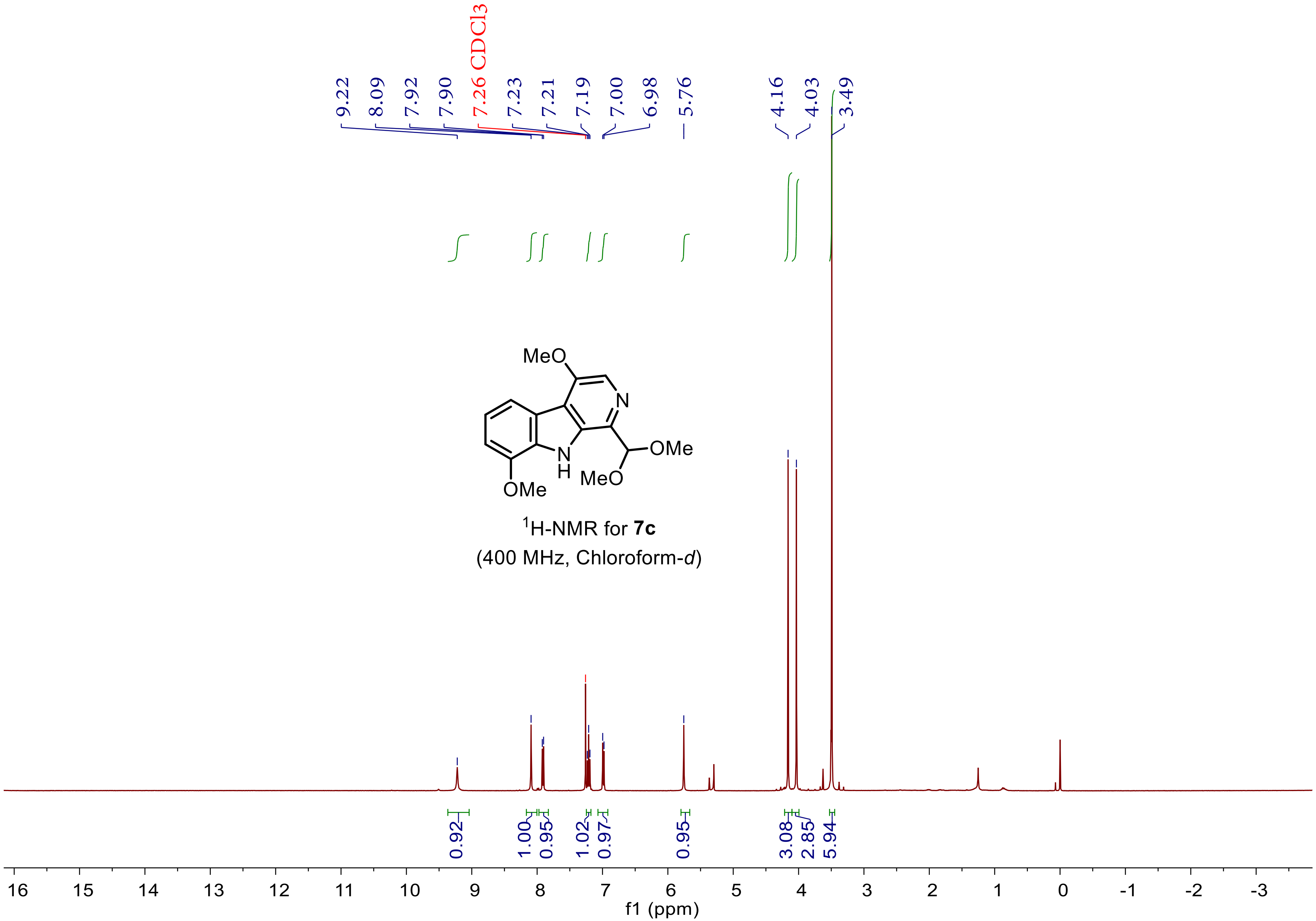

^{1}H-NMR for **7c**

(400 MHz, Chloroform-*d*)

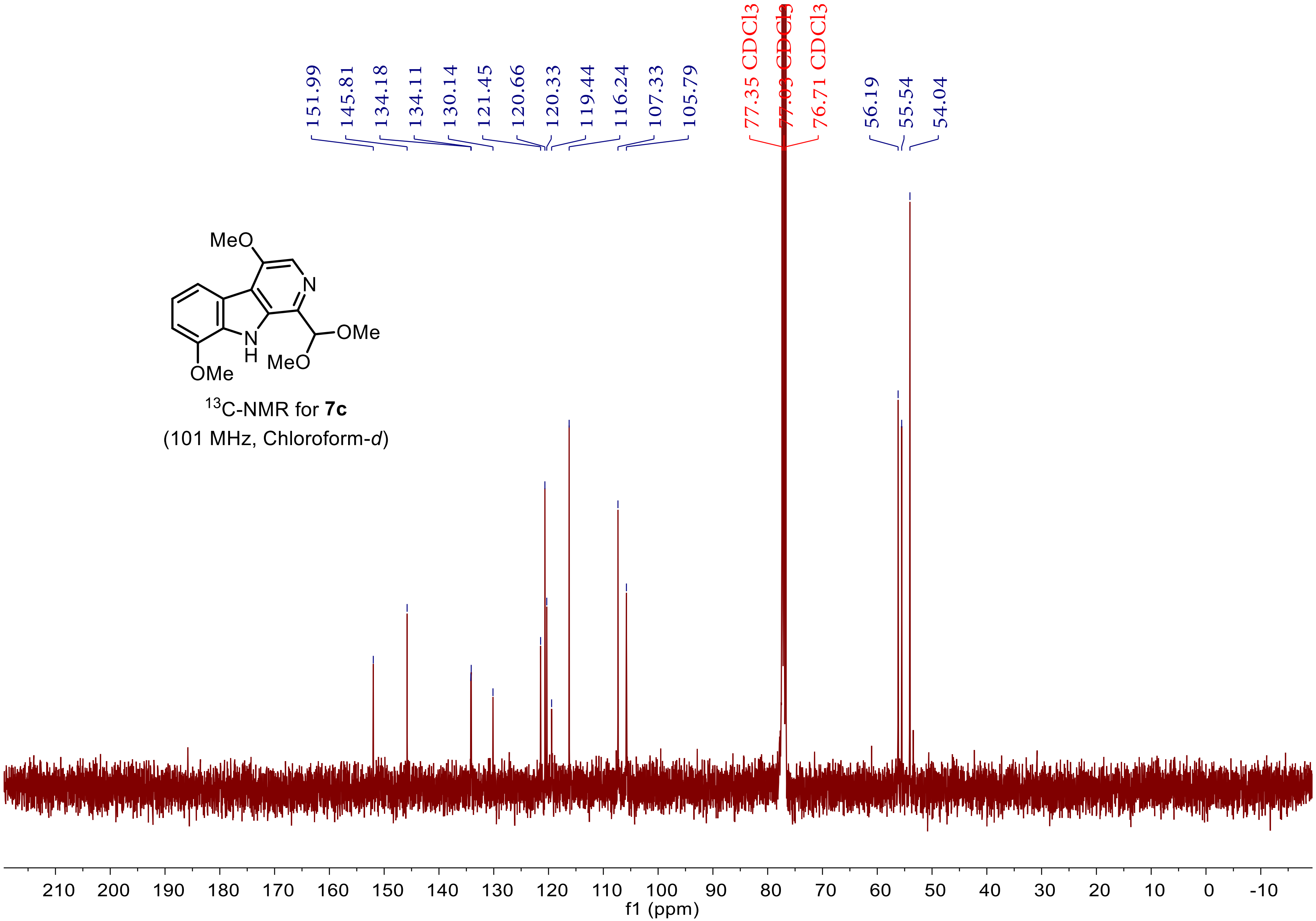

^{13}C-NMR for **7c**
(101 MHz, Chloroform-*d*)

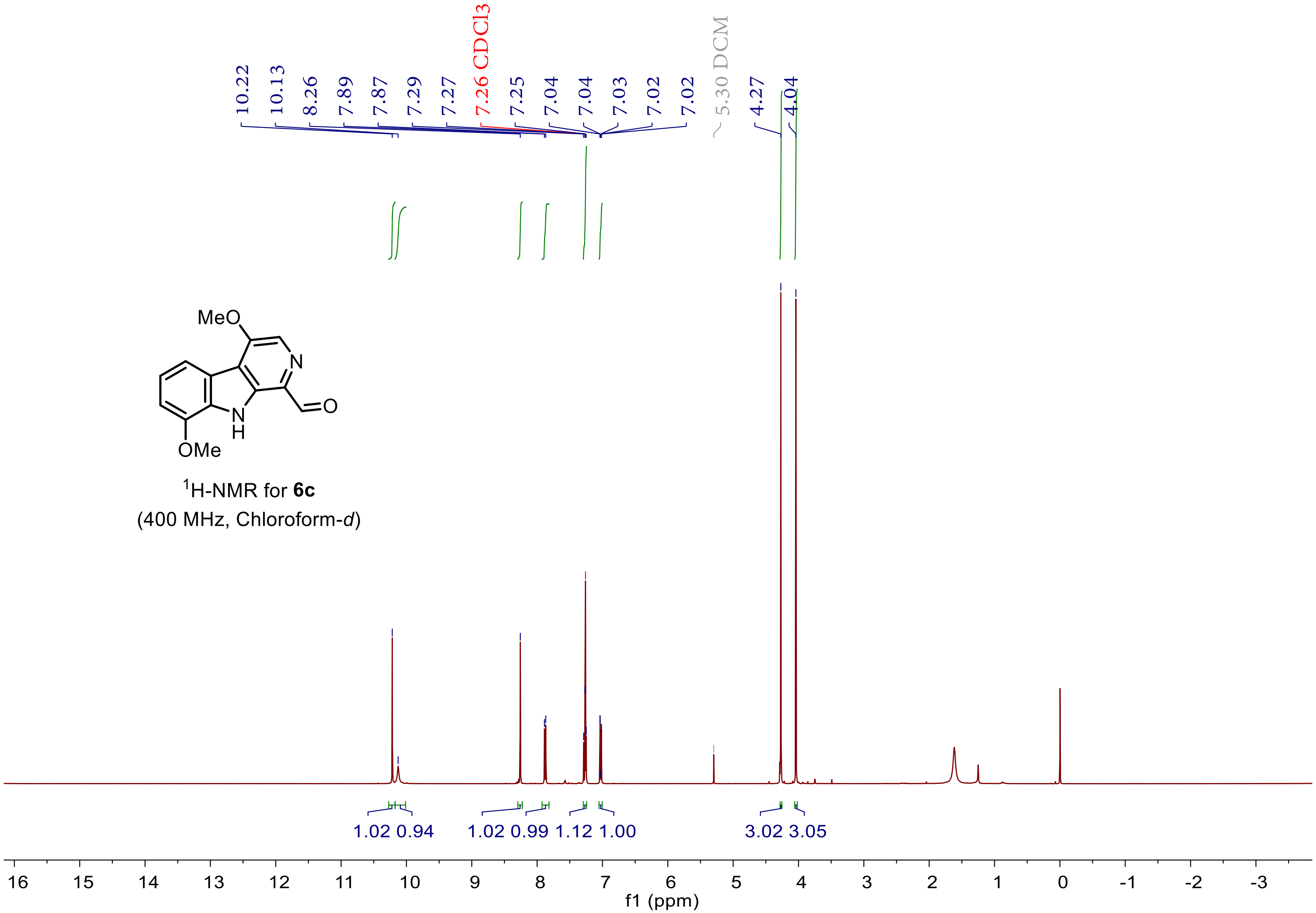

^{1}H-NMR for **6c**
(400 MHz, Chloroform-*d*)

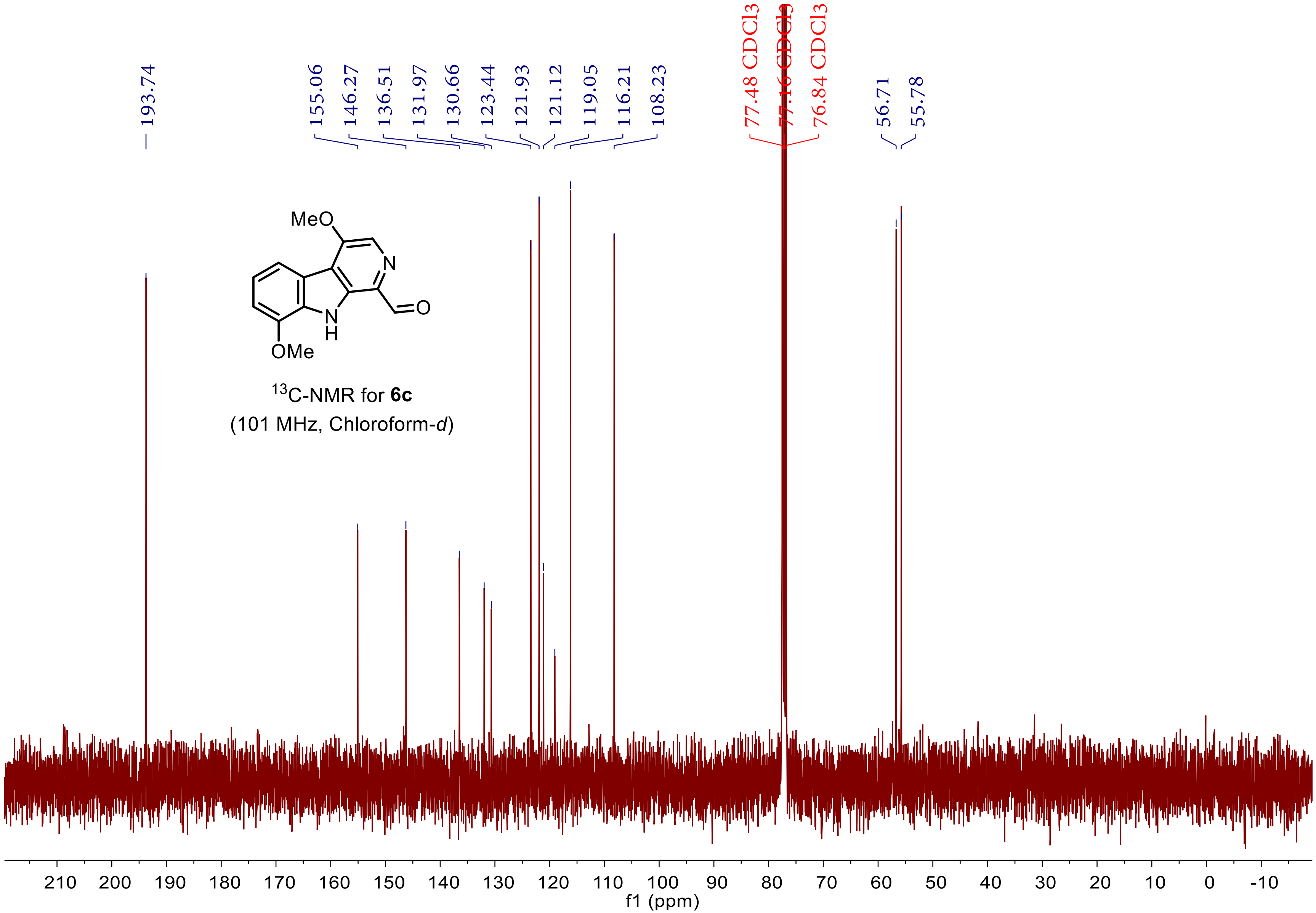
193.74
155.06
146.27
136.51
131.97
130.66
123.44
121.93
121.12
119.05
116.21
108.23
77.48 CDCl3
77.16 CDCl3
76.84 CDCl3
56.71
55.78
MeO
N
N
H
O
OMe
13C-NMR for 6c
(101 MHz, Chloroform-d)
210 200 190 180 170 160 150 140 130 120 110 100 90 80 70 60 50 40 30 20 10 0 -10
f1 (ppm)

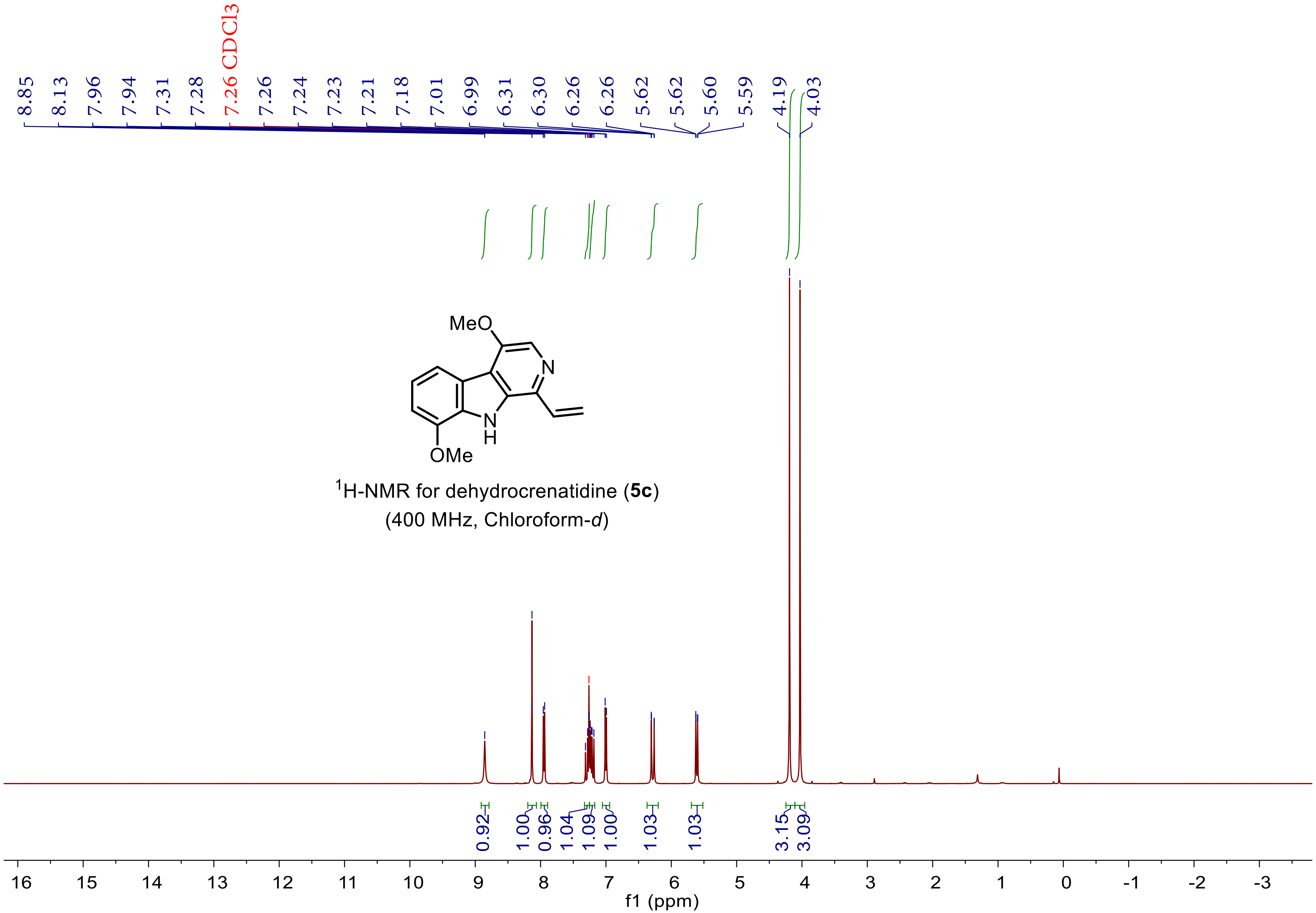

^{1}H-NMR for dehydrocrenatidine (**5c**)
(400 MHz, Chloroform-*d*)

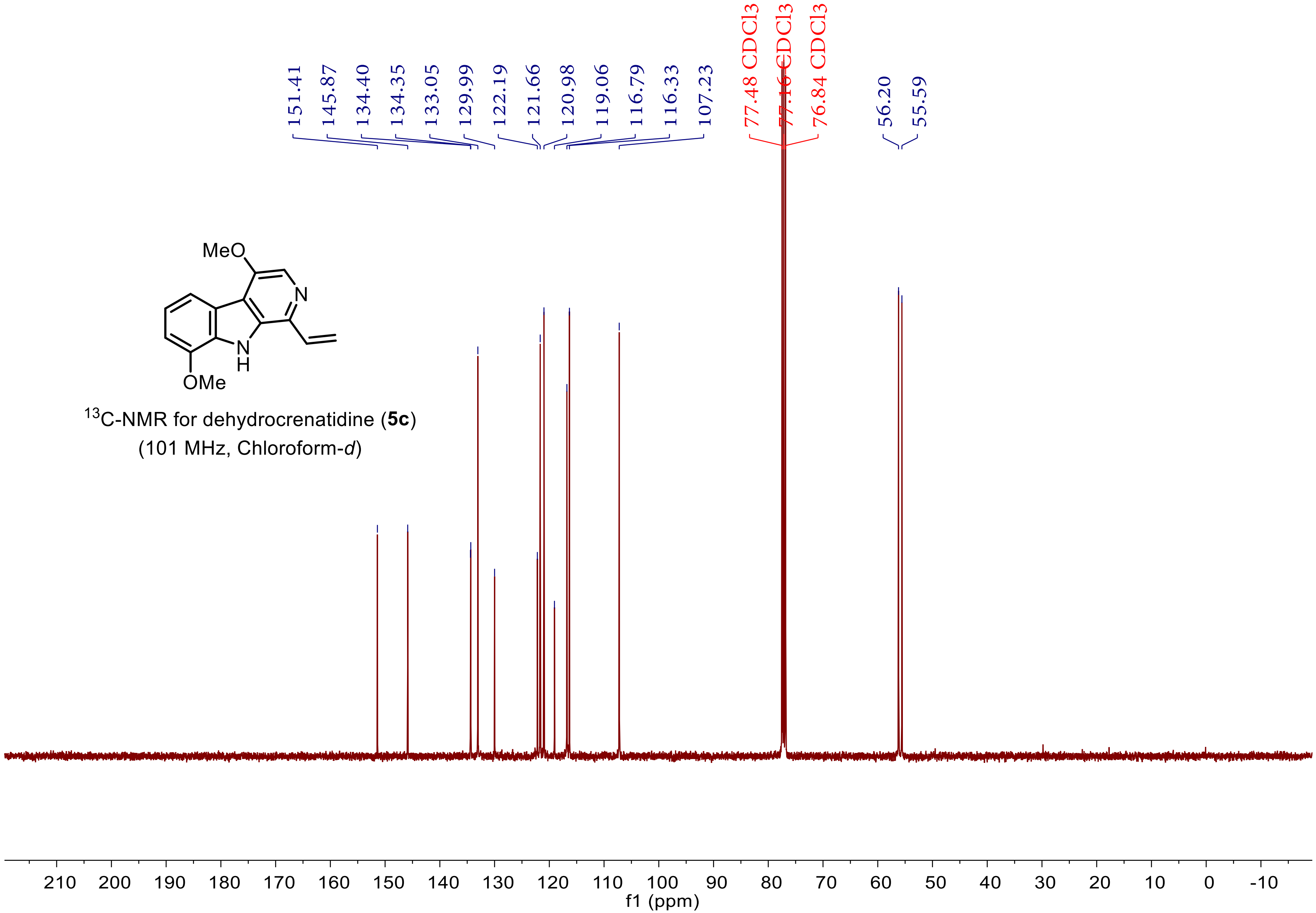

^{13}C-NMR for dehydrocrenatidine (**5c**)
(101 MHz, Chloroform-*d*)

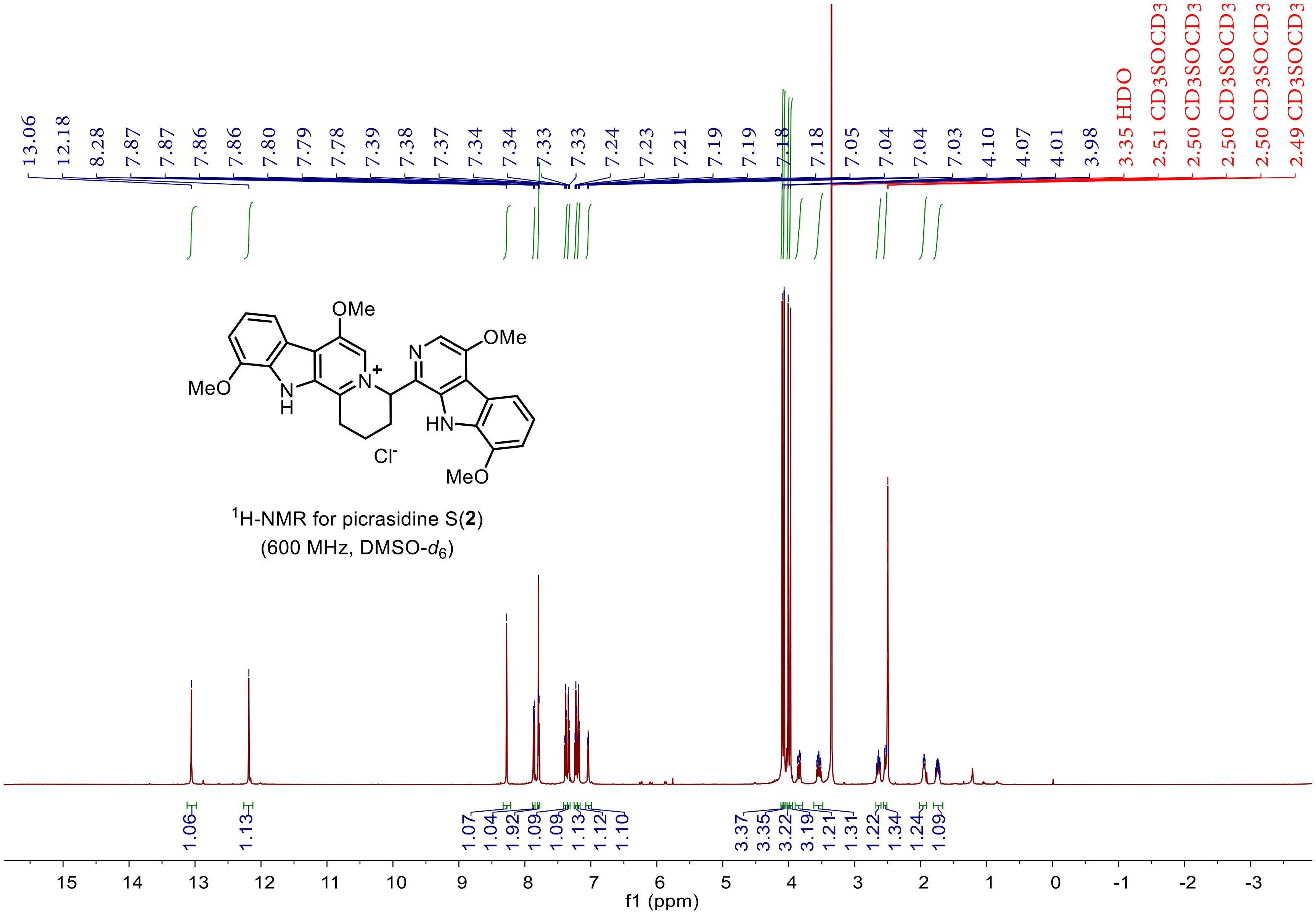

^{1}H-NMR for picrasidine S(**2**)
(600 MHz, DMSO-d_6)

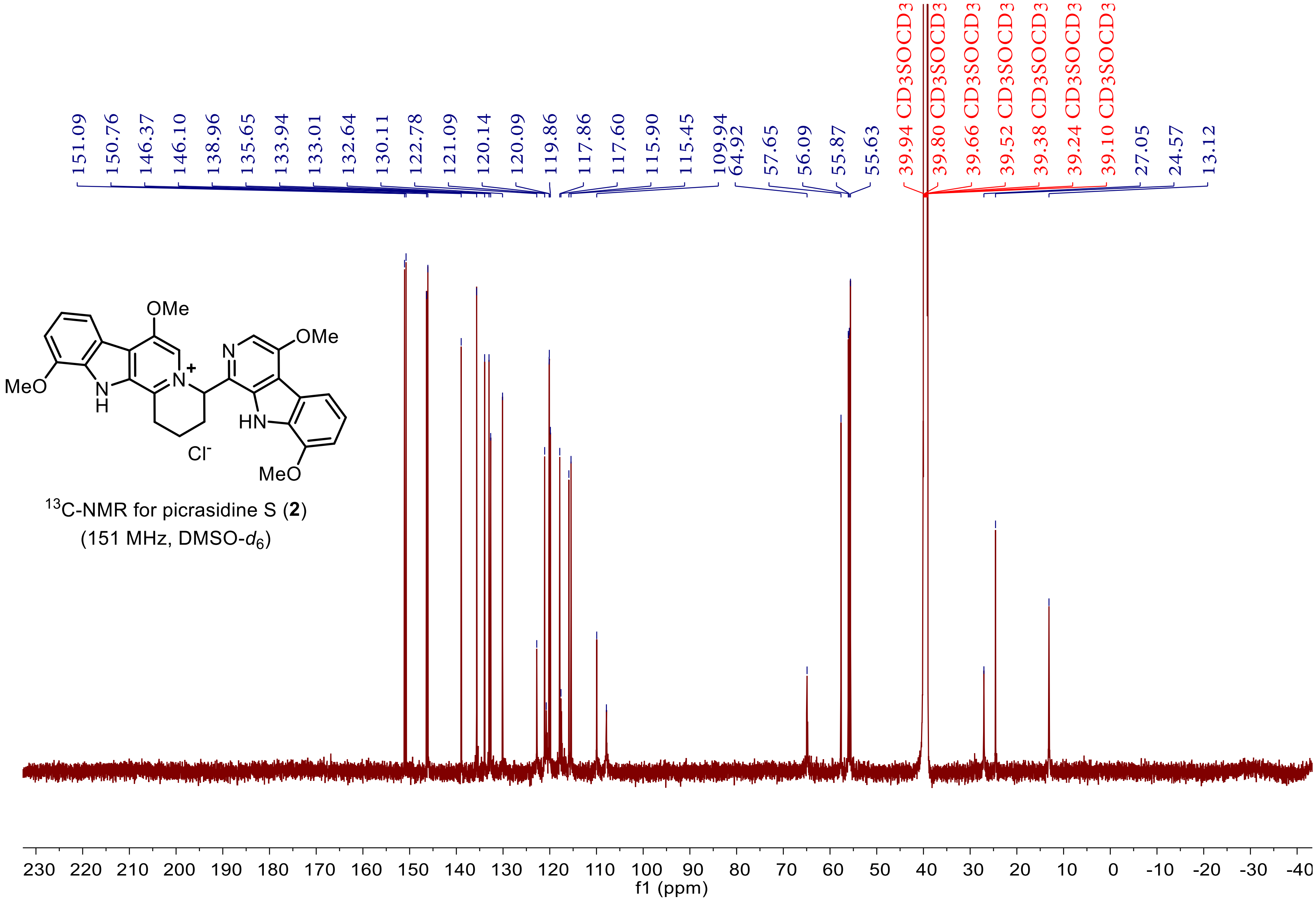

^{13}C-NMR for picrasidine S (**2**)
(151 MHz, DMSO-d_6)

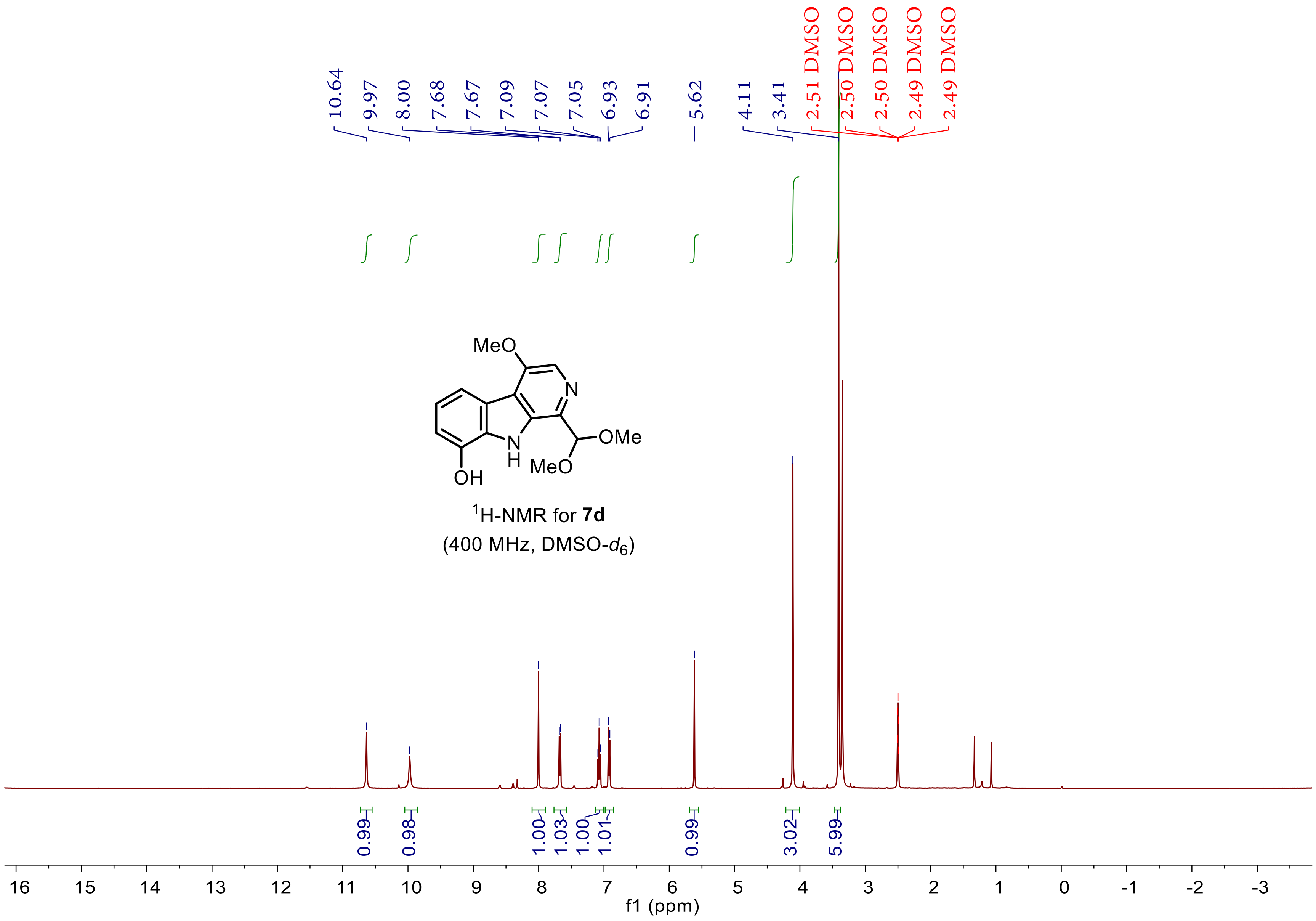
10.64
9.97
8.00
7.68
7.67
7.09
7.07
7.05
6.93
6.91
5.62
4.11
3.41
2.51 DMSO
2.50 DMSO
2.50 DMSO
2.49 DMSO
2.49 DMSO
MeO
N
N
H
OH
OMe
MeO
^{1}H-NMR for **7d**
(400 MHz, DMSO-d_6)
0.99
0.98
1.00
1.03
1.00
1.01
0.99
3.02
5.99
16 15 14 13 12 11 10 9 8 7 6 5 4 3 2 1 0 -1 -2 -3
f1 (ppm)

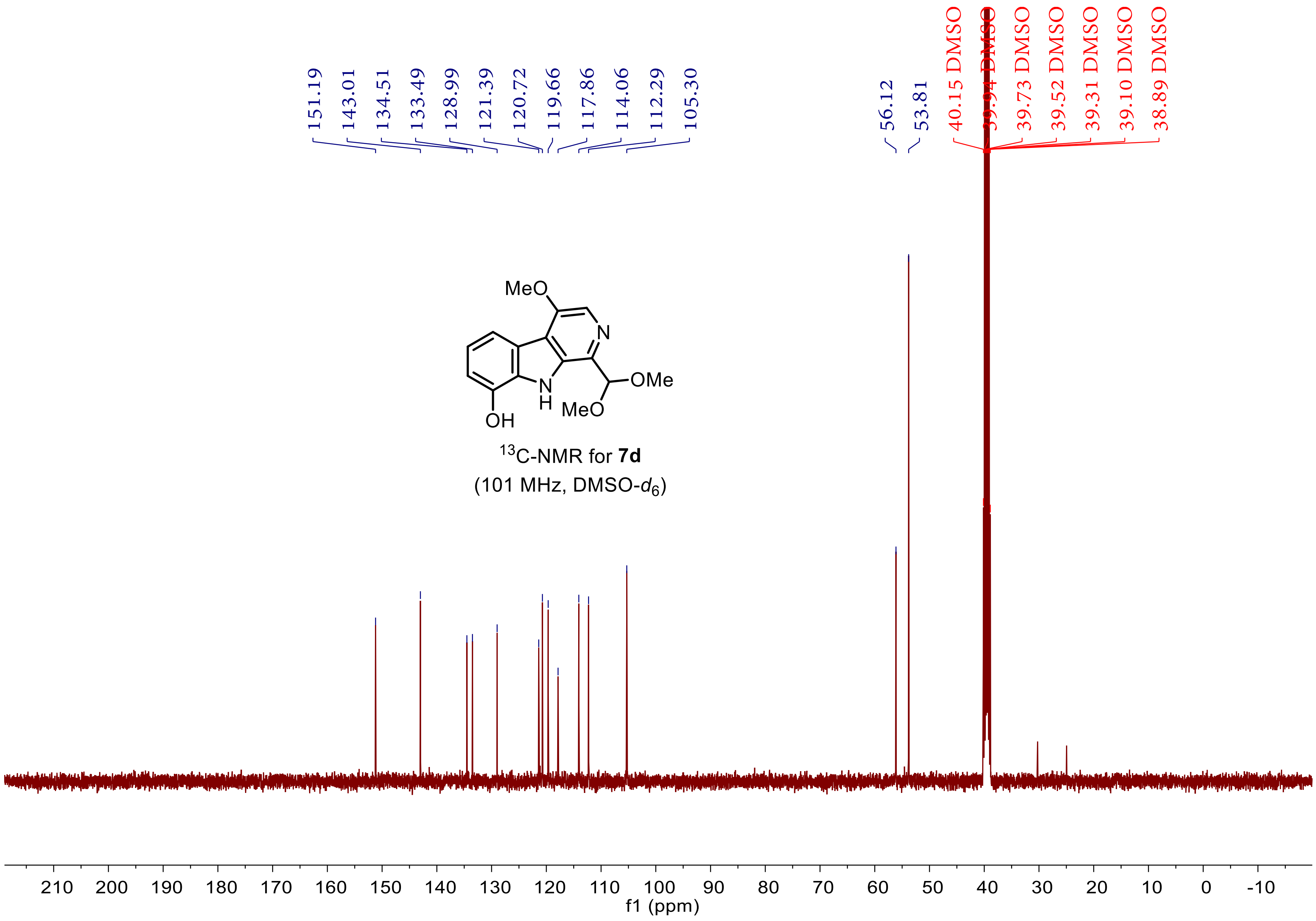
151.19
143.01
134.51
133.49
128.99
121.39
120.72
119.66
117.86
114.06
112.29
105.30
56.12
53.81
40.15 DMSO
39.73 DMSO
39.52 DMSO
39.31 DMSO
39.10 DMSO
38.89 DMSO
MeO
N
OMe
N
H
MeO
OH
^{13}C-NMR for **7d**
(101 MHz, DMSO-d_6)
210 200 190 180 170 160 150 140 130 120 110 100 90 80 70 60 50 40 30 20 10 0 -10
f1 (ppm)

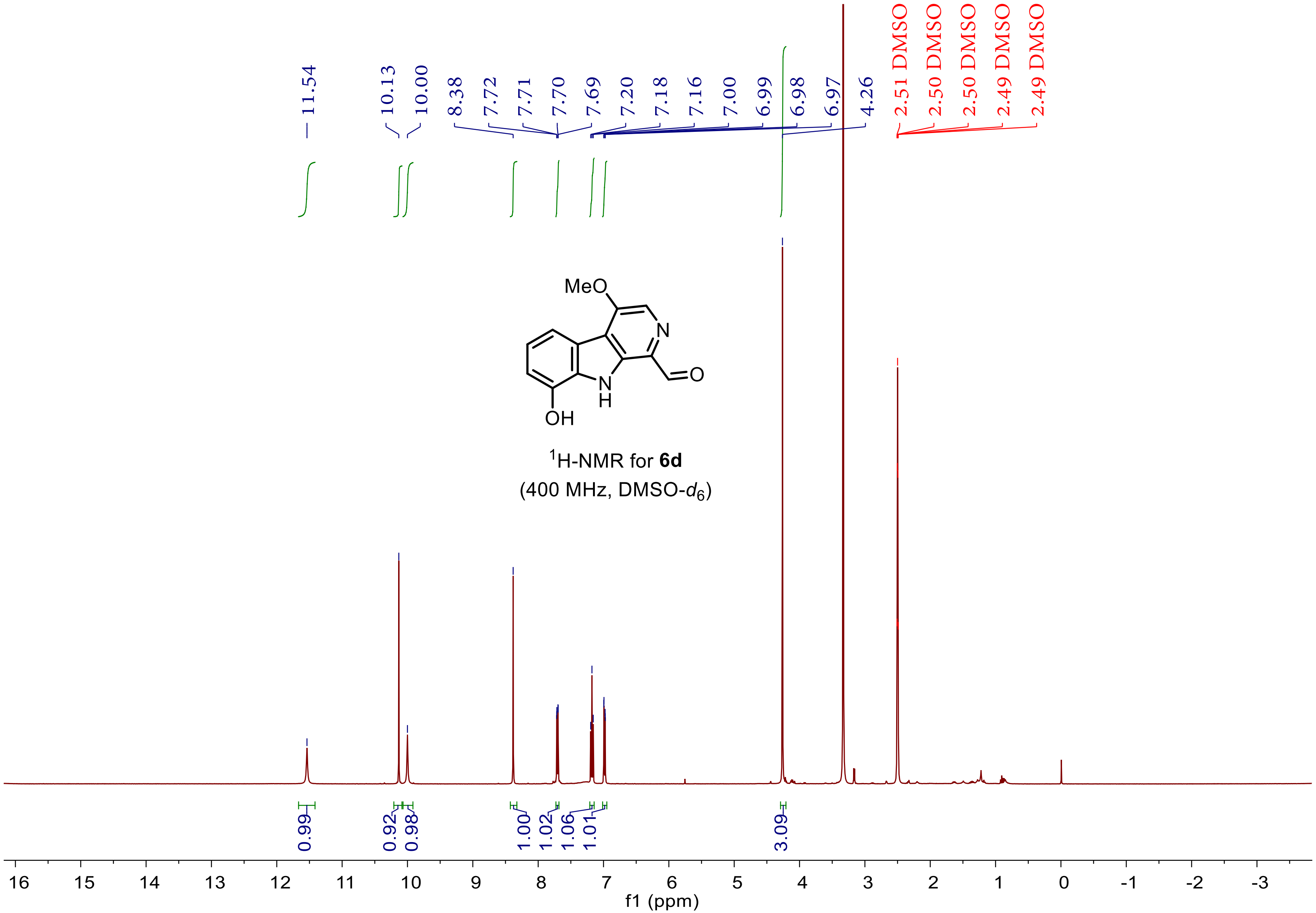

^{1}H-NMR for **6d**
(400 MHz, DMSO-d_6)

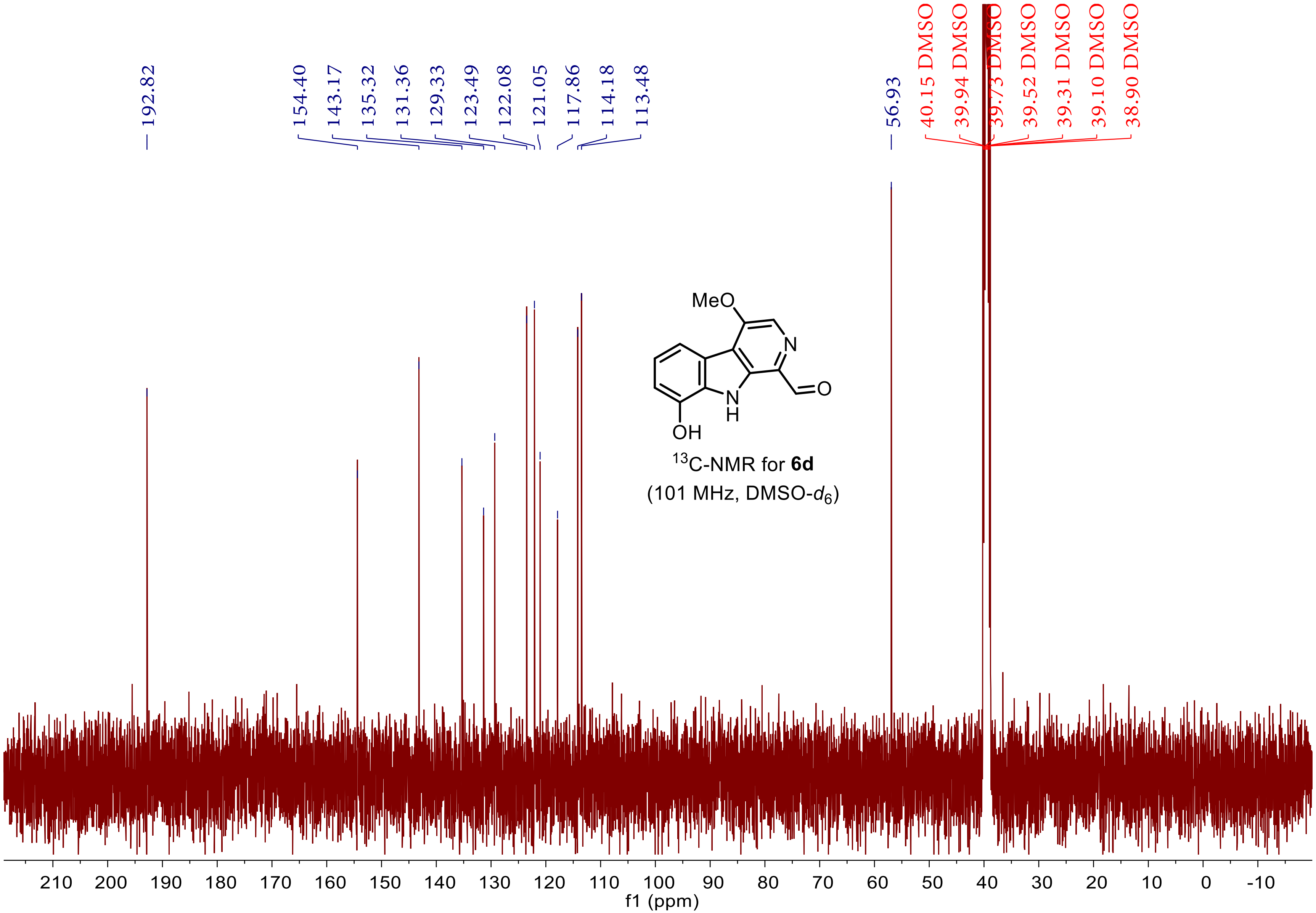

^{13}C-NMR for **6d**
(101 MHz, DMSO-d_6)

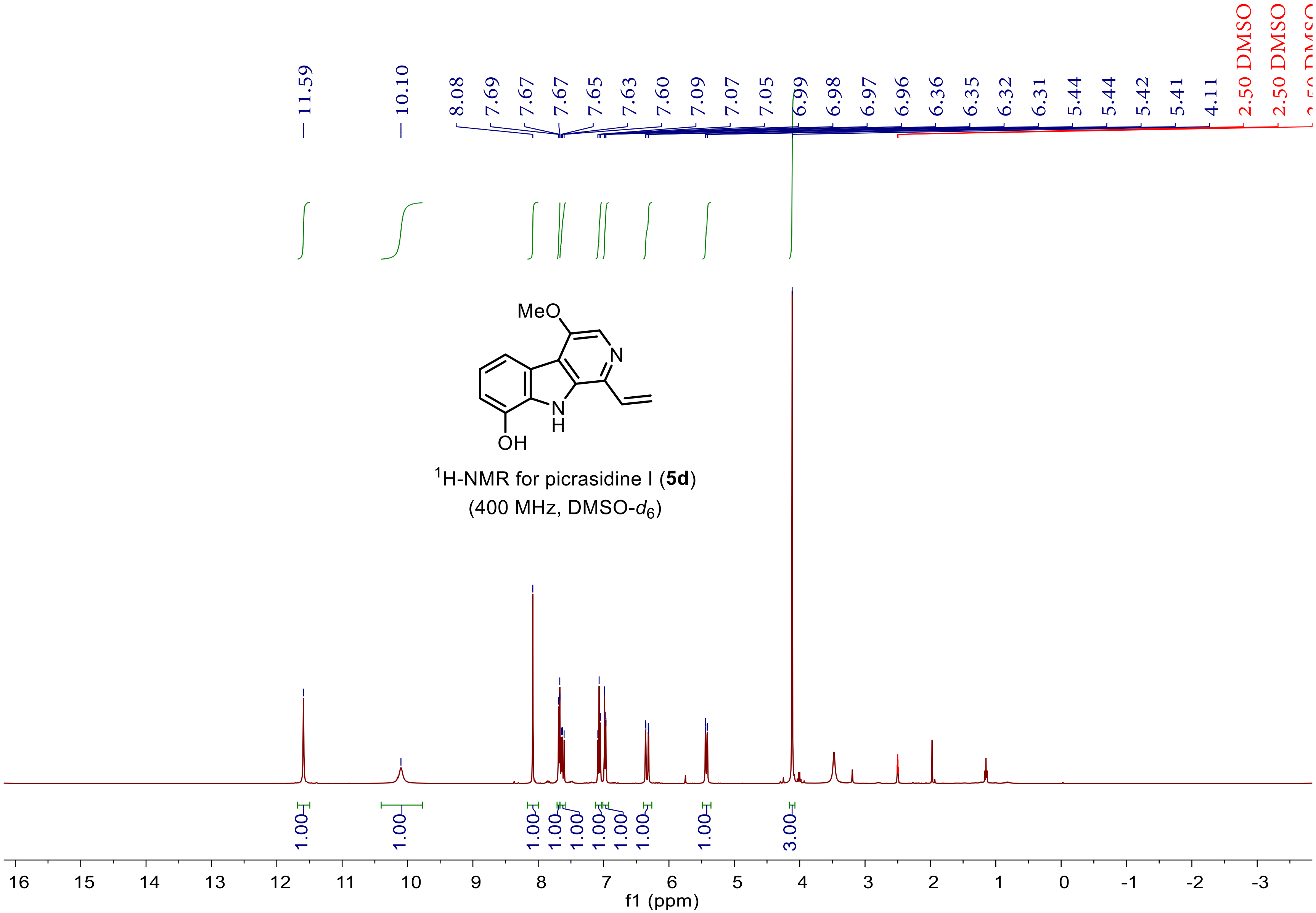

^{1}H-NMR for picrasidine I (**5d**)
(400 MHz, DMSO-d_6)

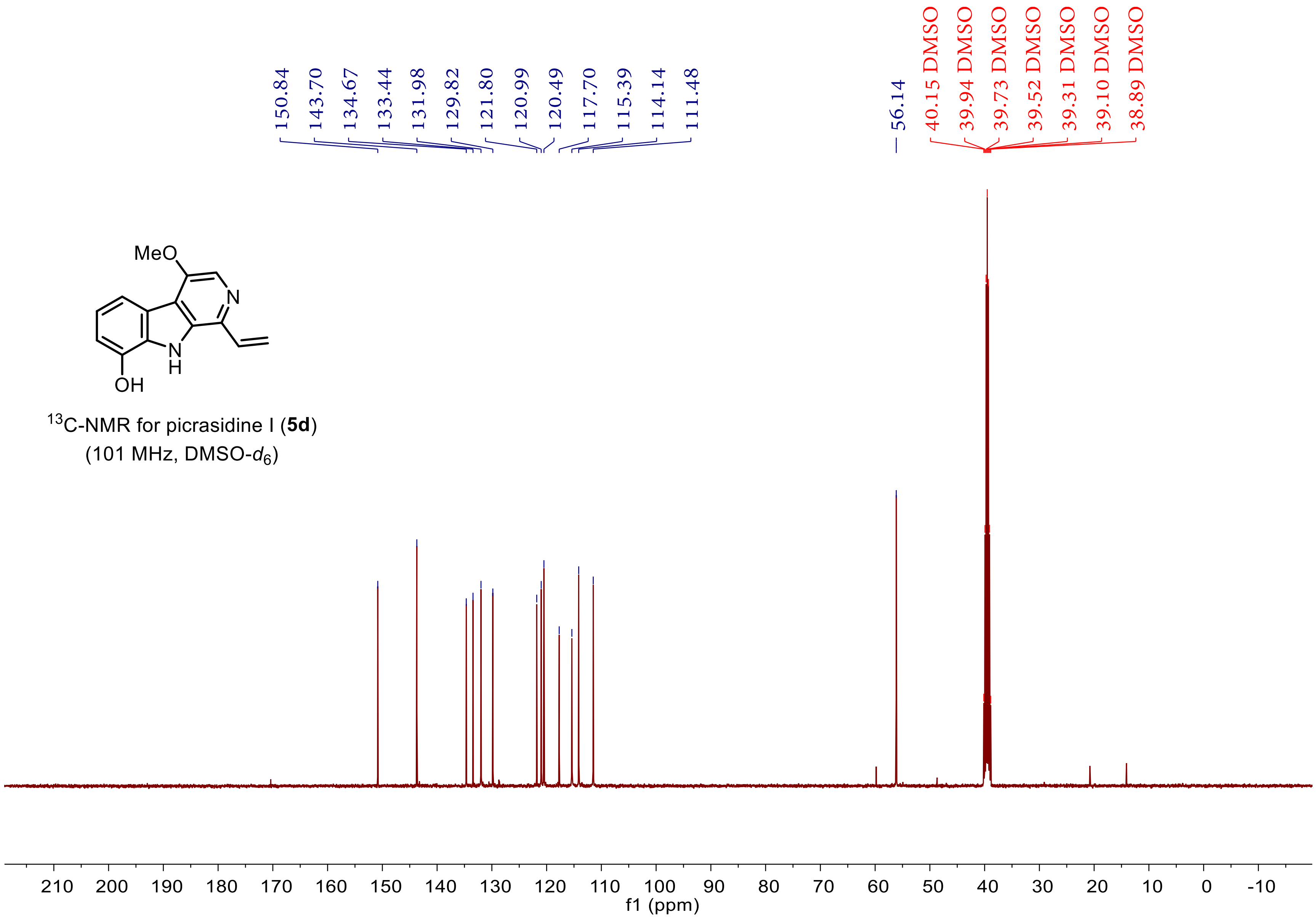

^{13}C-NMR for picrasidine I (**5d**)
(101 MHz, DMSO-d_6)

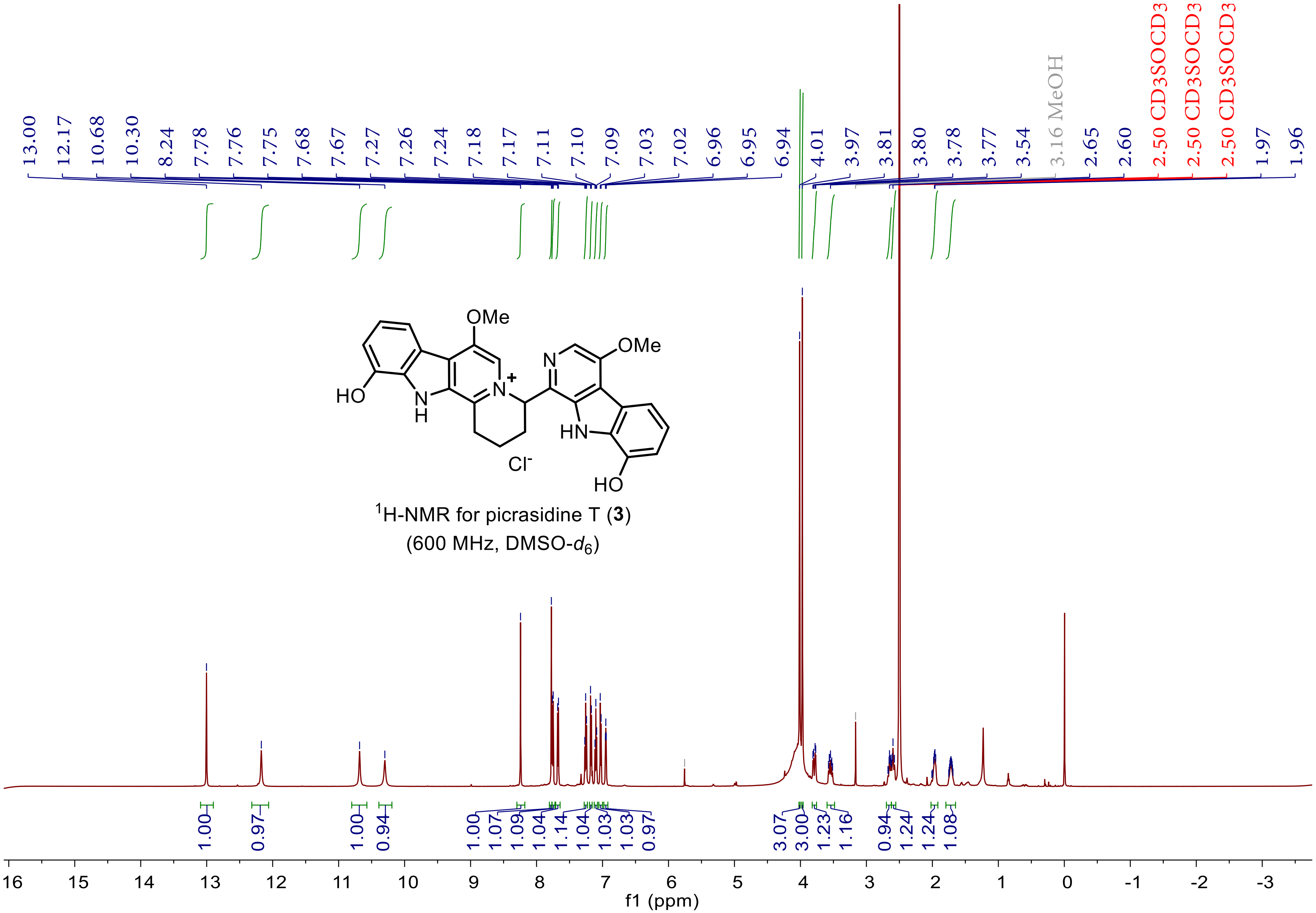

^{1}H-NMR for picrasidine T (**3**)
(600 MHz, DMSO-d_6)

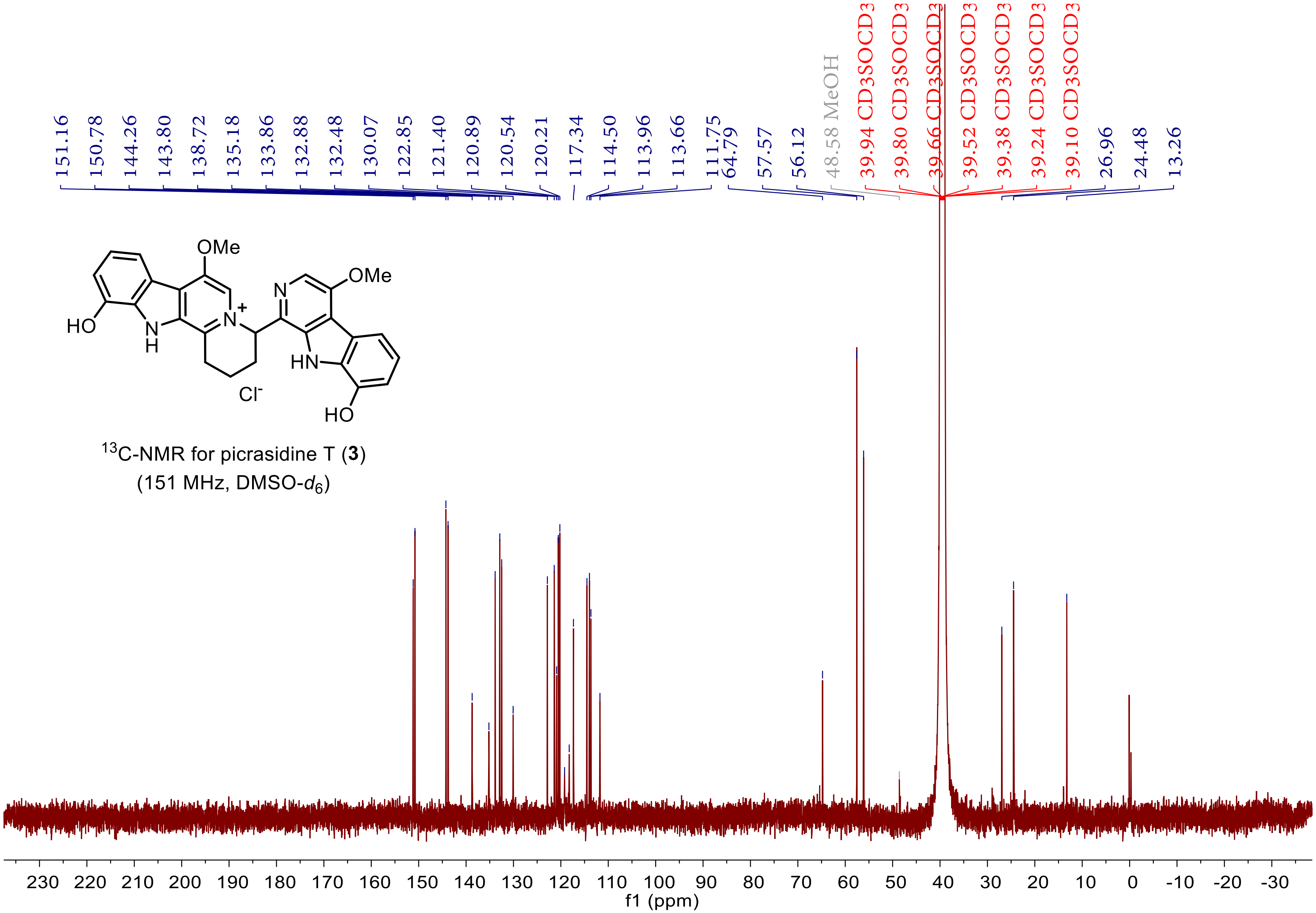

^{13}C-NMR for picrasidine T (**3**)
(151 MHz, DMSO-d_6)

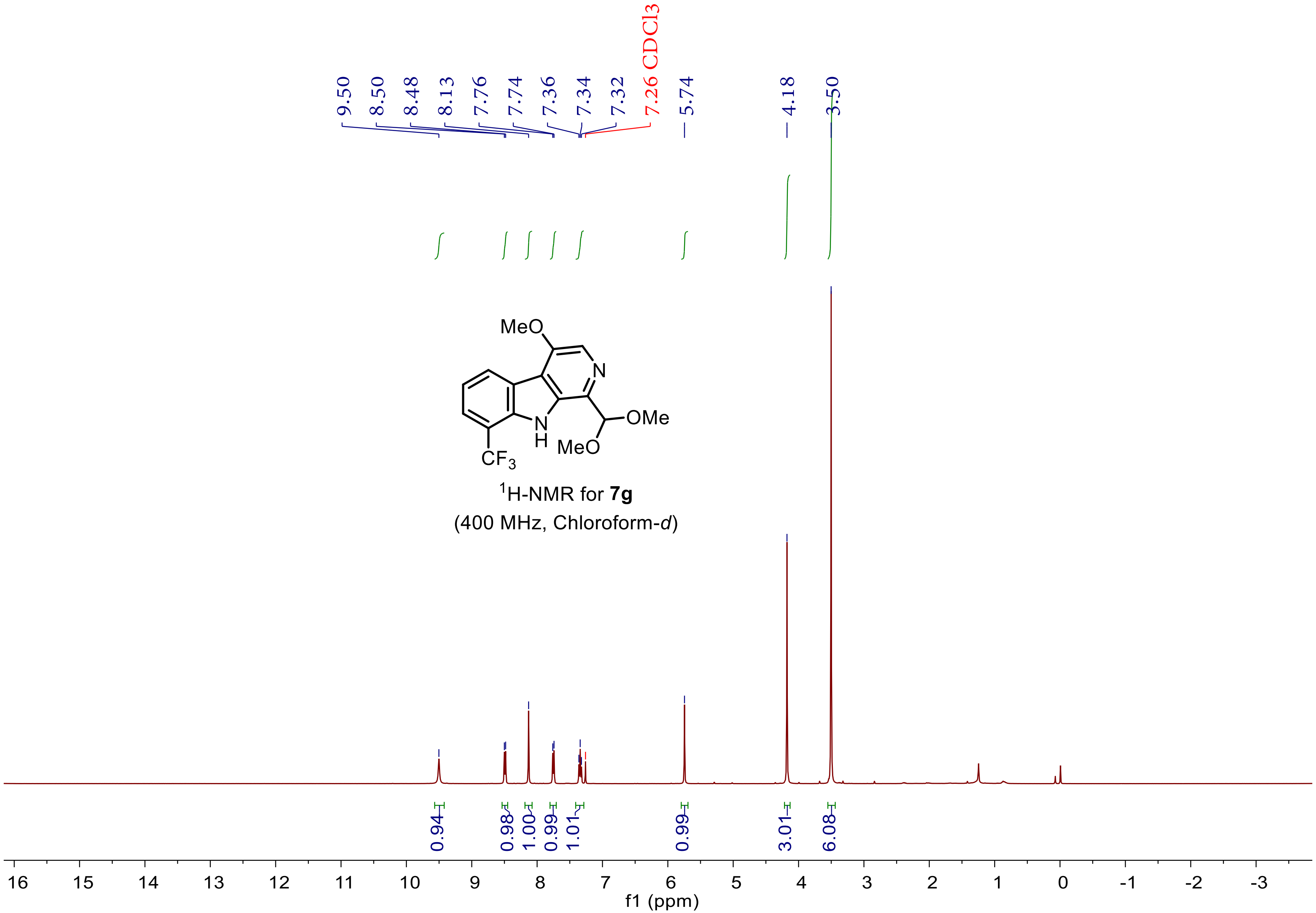

^{1}H-NMR for **7g**
(400 MHz, Chloroform-*d*)

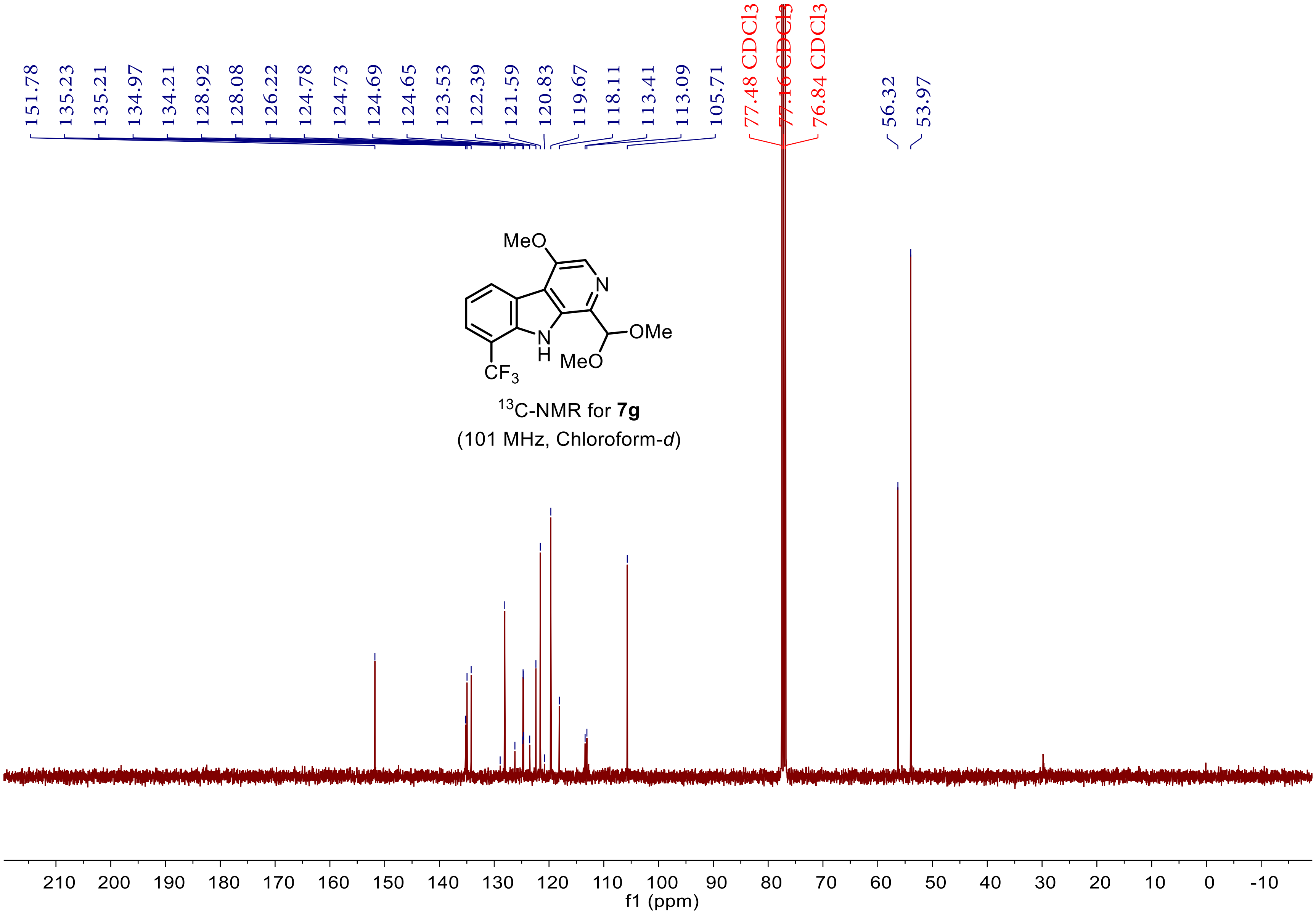

151.78
135.23
135.21
134.97
134.21
128.92
128.08
126.22
124.78
124.73
124.69
124.65
123.53
122.39
121.59
120.83
119.67
118.11
113.41
113.09
105.71
77.48 CDCl3
77.16 CDCl3
76.84 CDCl3
56.32
53.97
MeO
N
OMe
N
H
MeO
CF_3
^{13}C-NMR for **7g**
(101 MHz, Chloroform-*d*)
210 200 190 180 170 160 150 140 130 120 110 100 90 80 70 60 50 40 30 20 10 0 -10
f1 (ppm)

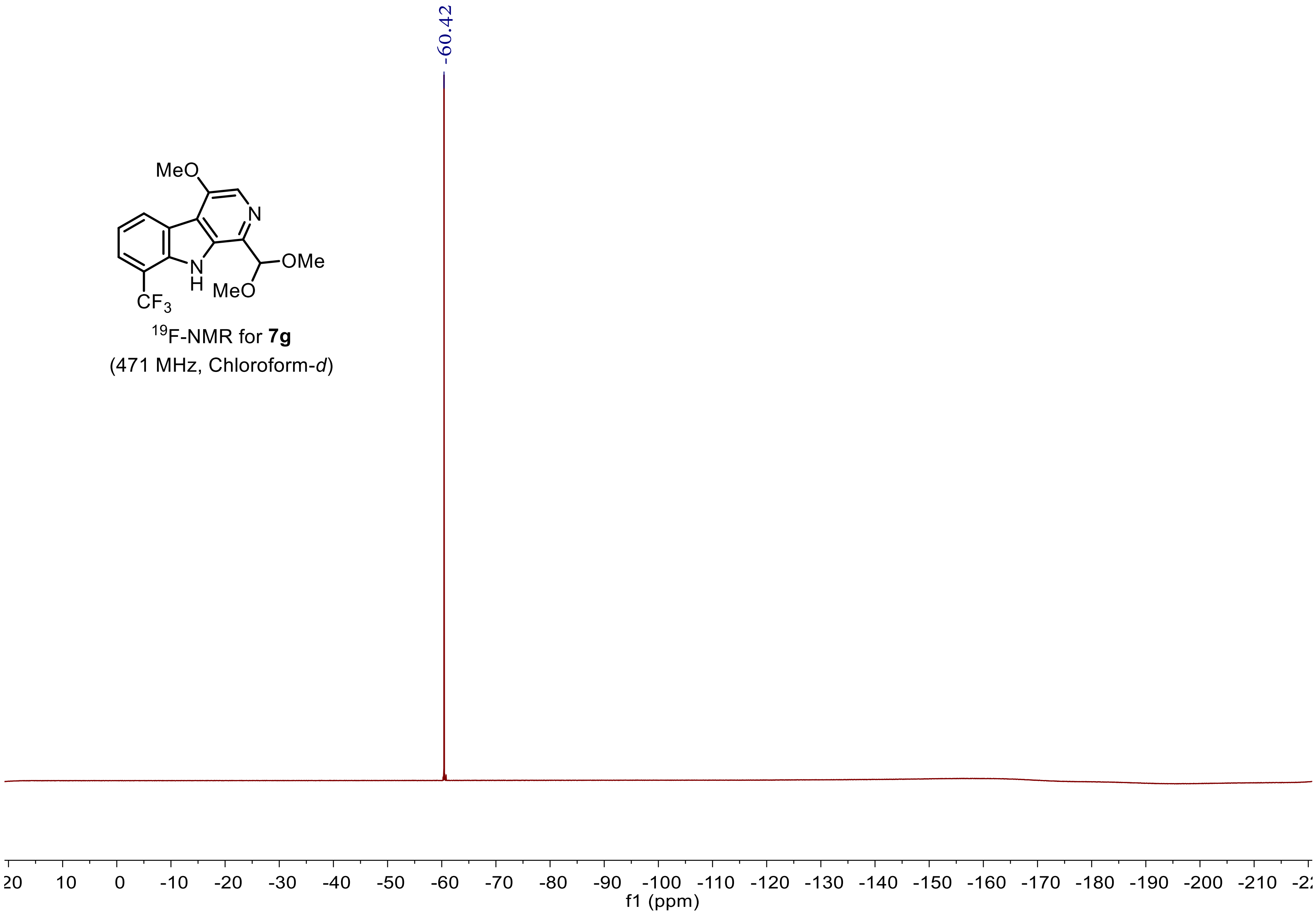
-60.42
MeO
N
OMe
N
H
MeO
CF3
19F-NMR for 7g
(471 MHz, Chloroform-d)
20 10 0 -10 -20 -30 -40 -50 -60 -70 -80 -90 -100 -110 -120 -130 -140 -150 -160 -170 -180 -190 -200 -210
f1 (ppm)

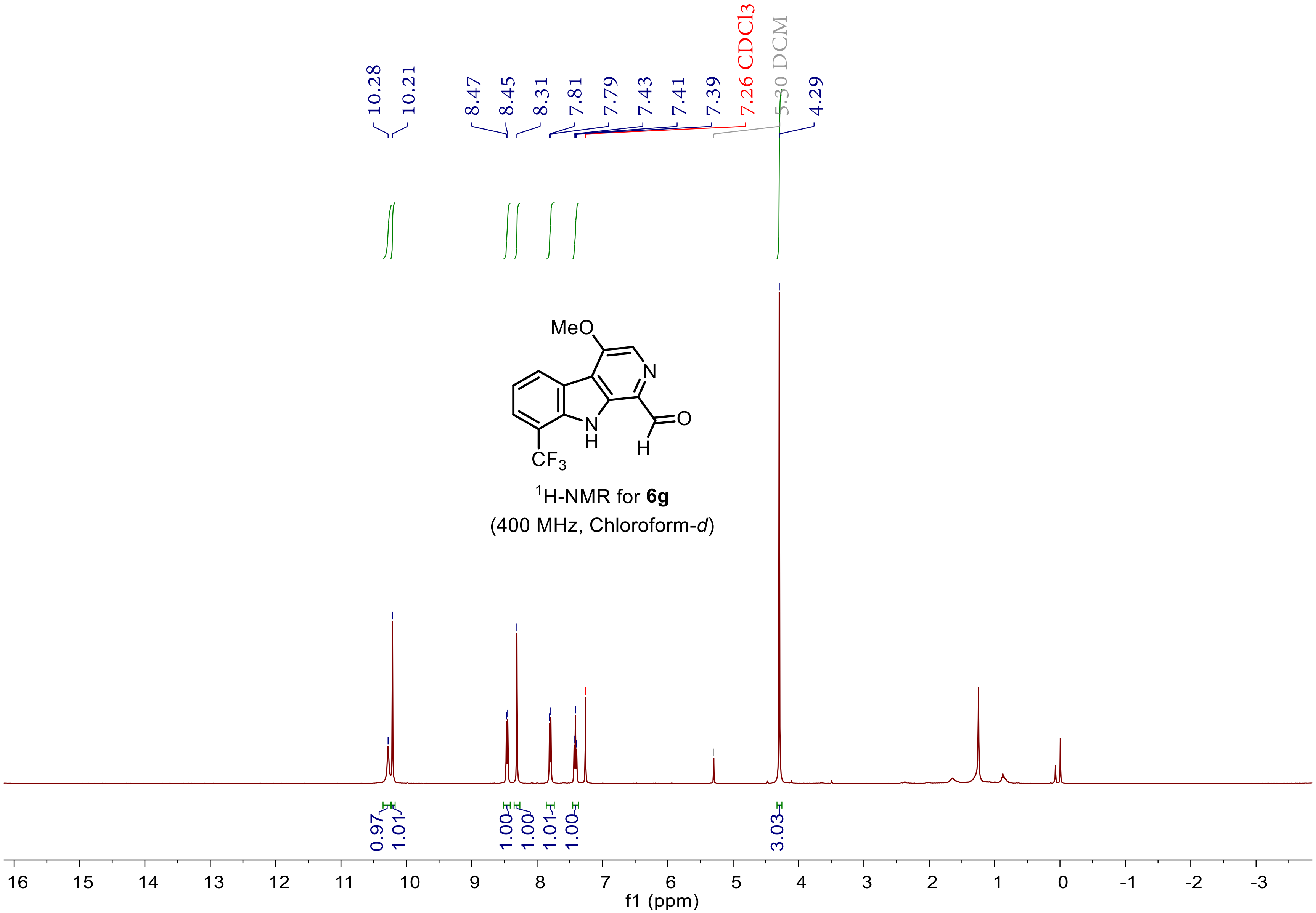

^{1}H-NMR for **6g**

(400 MHz, Chloroform-*d*)

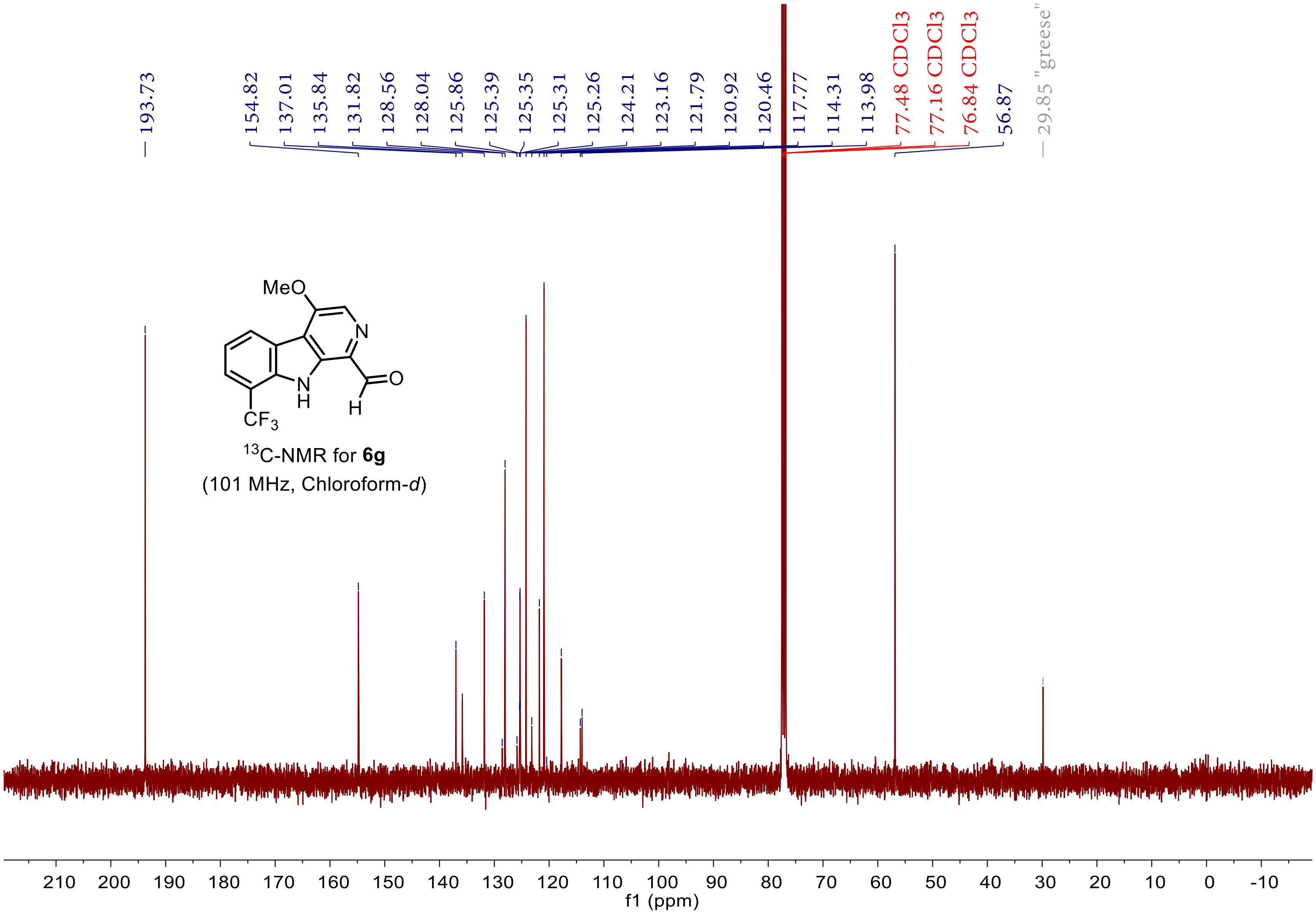

^{13}C-NMR for **6g**
(101 MHz, Chloroform-*d*)

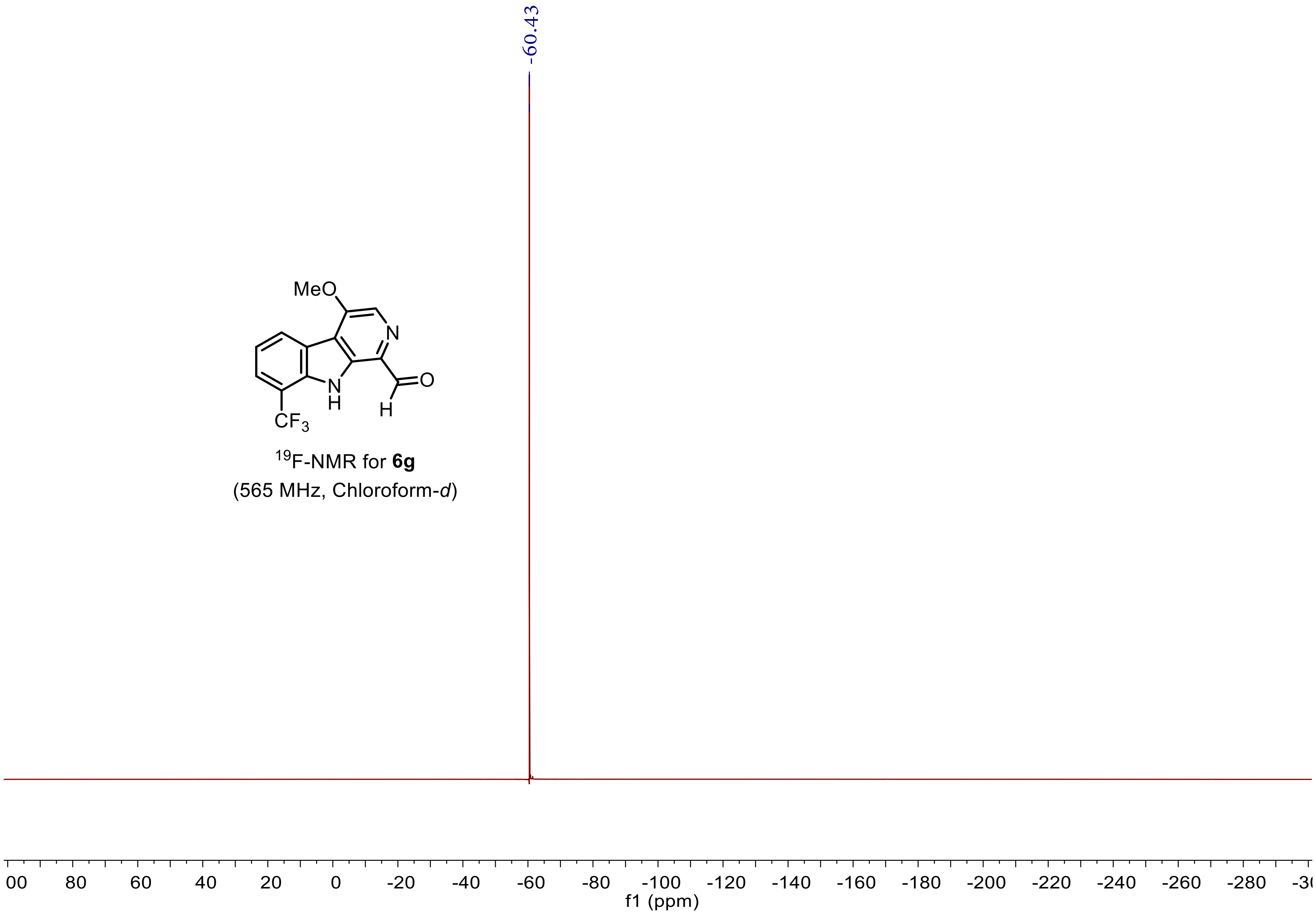

^{19}F-NMR for **6g**
(565 MHz, Chloroform-*d*)

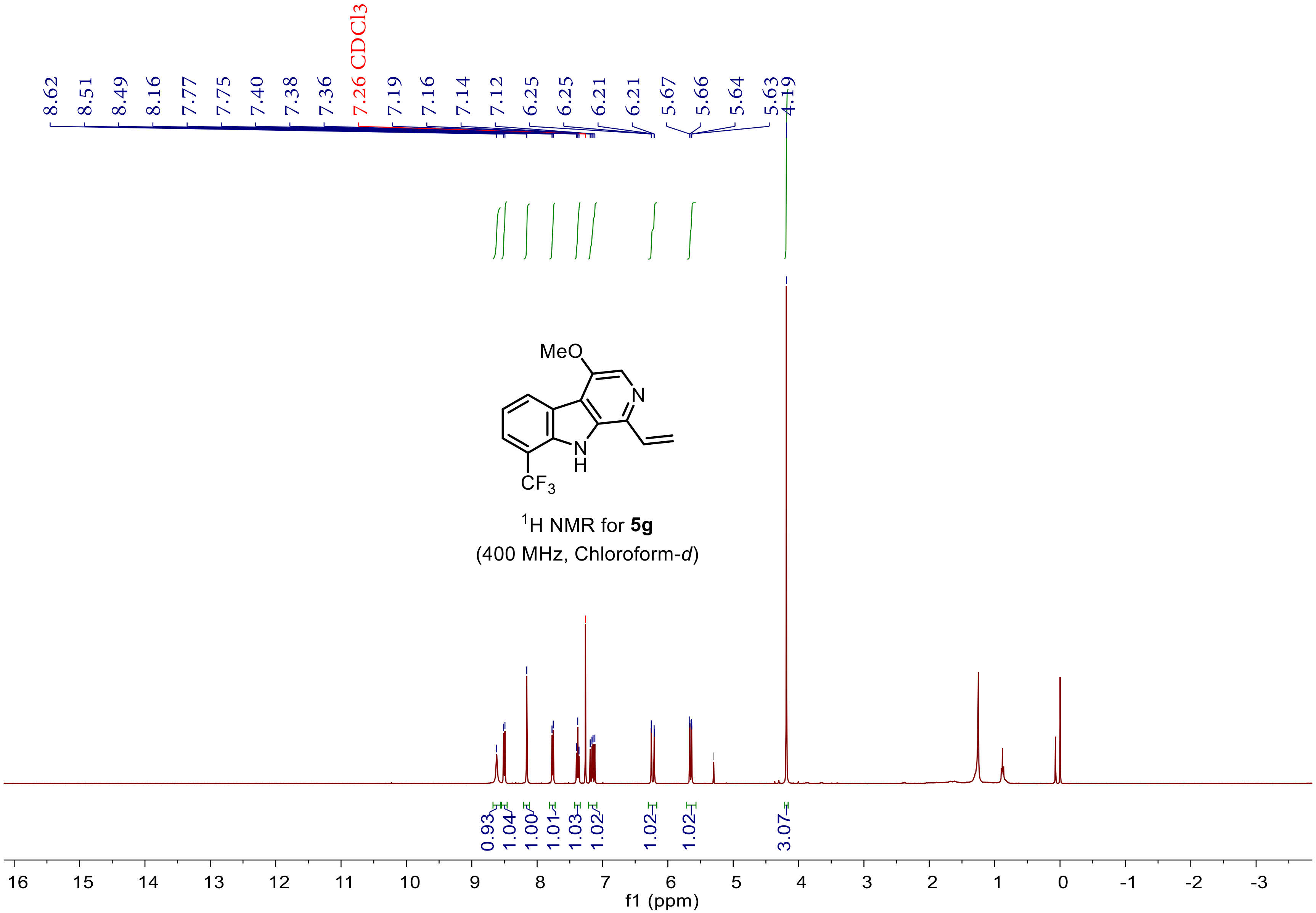

^{1}H NMR for **5g**
(400 MHz, Chloroform-*d*)

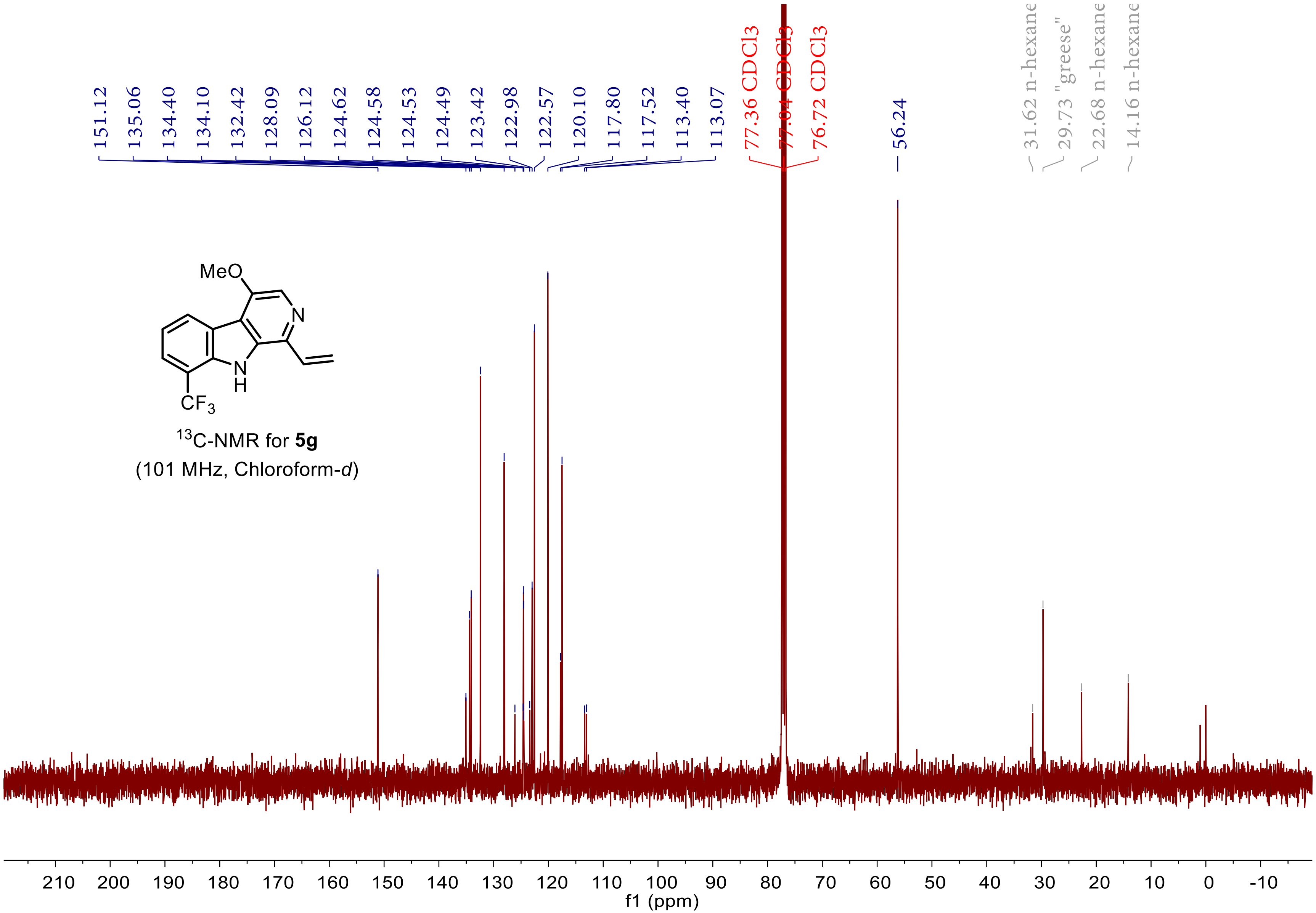

151.12
135.06
134.40
134.10
132.42
128.09
126.12
124.62
124.58
124.53
124.49
123.42
122.98
122.57
120.10
117.80
117.52
113.40
113.07
77.36 CDCl3
77.04 CDCl3
76.72 CDCl3
56.24
31.62 n-hexane
29.73 "greese"
22.68 n-hexane
14.16 n-hexane
MeO
N
N
H
CF3
13C-NMR for 5g
(101 MHz, Chloroform-d)
f1 (ppm)

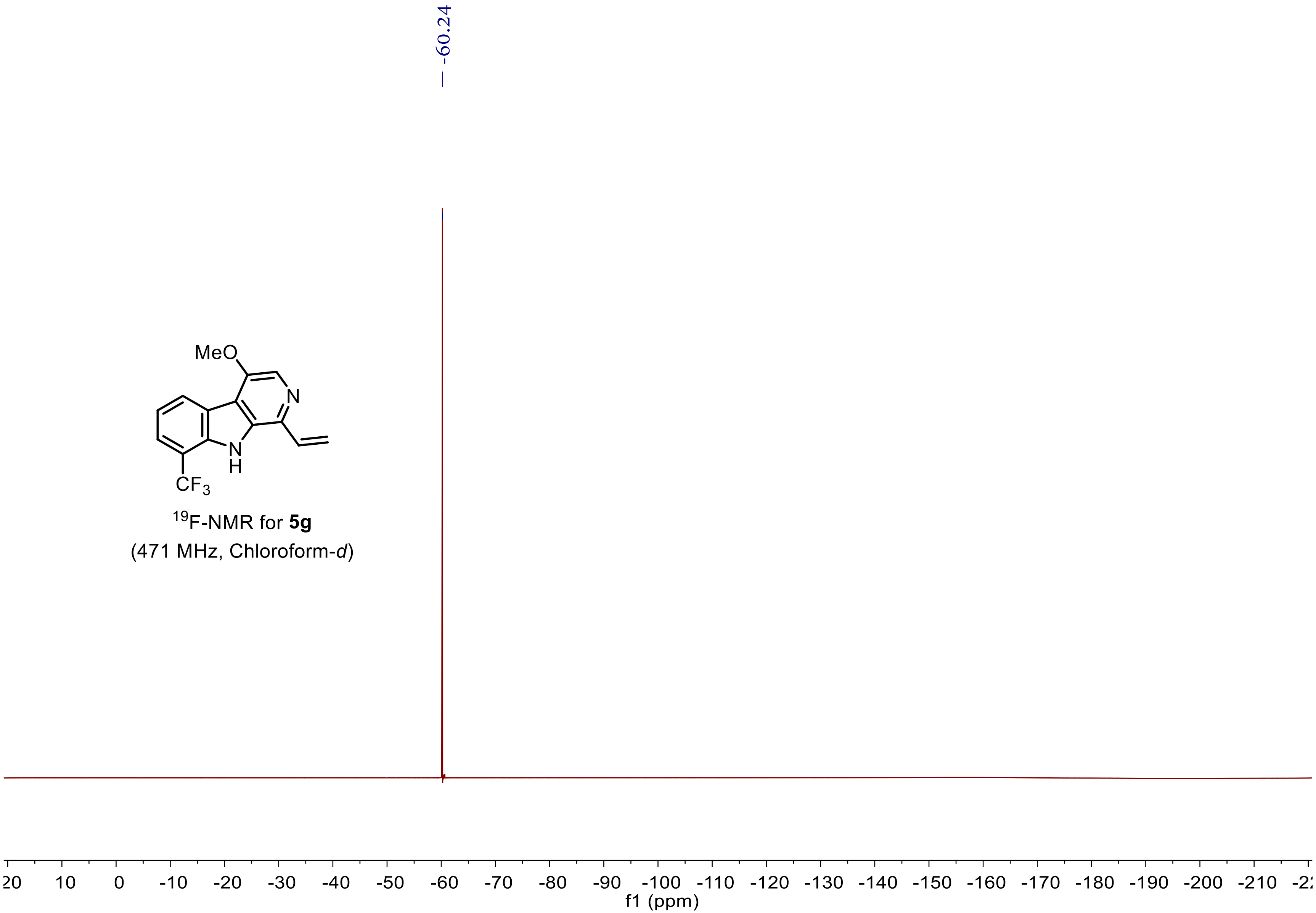

^{19}F-NMR for **5g**
(471 MHz, Chloroform-*d*)

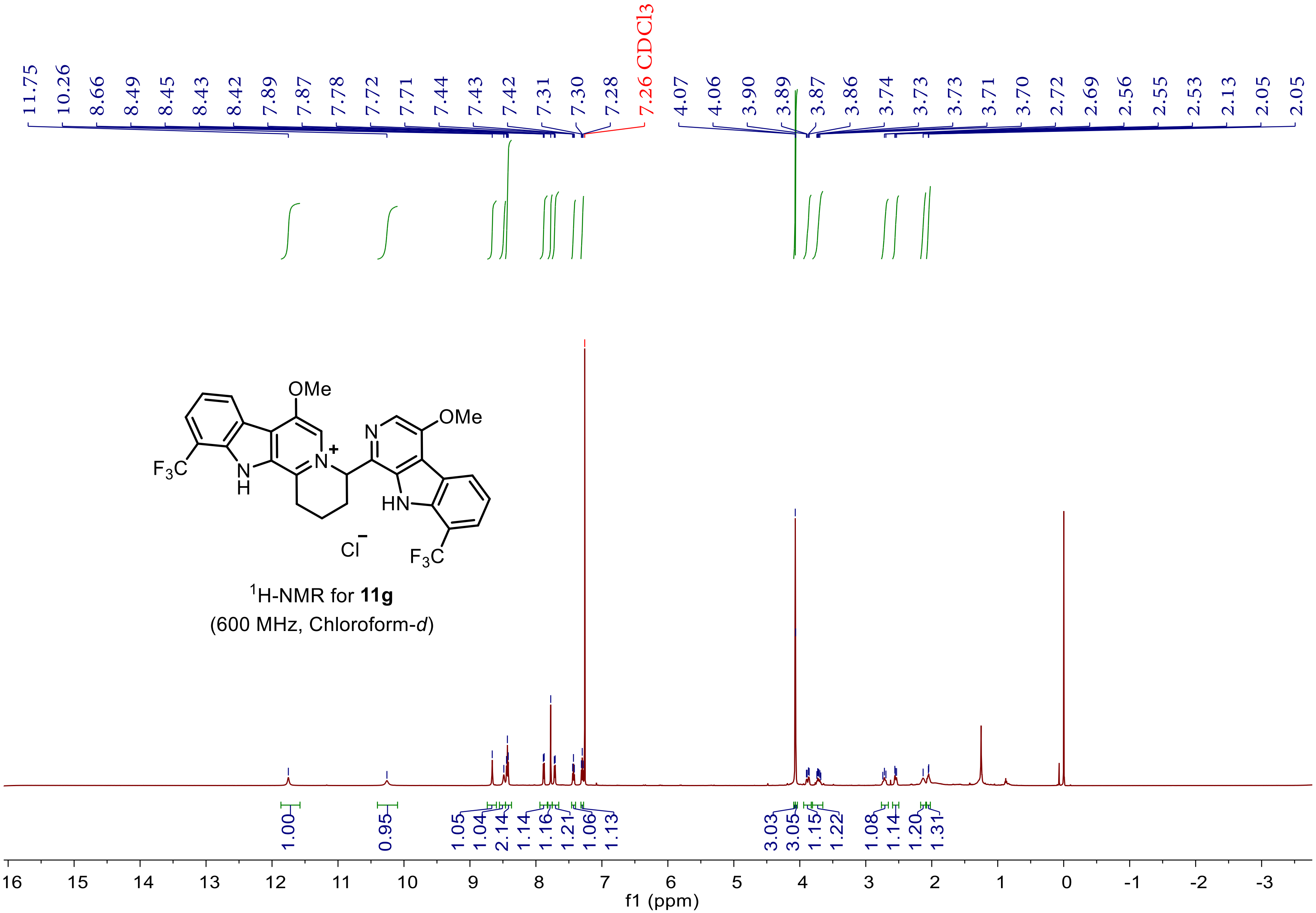

^{1}H-NMR for **11g**
(600 MHz, Chloroform-*d*)

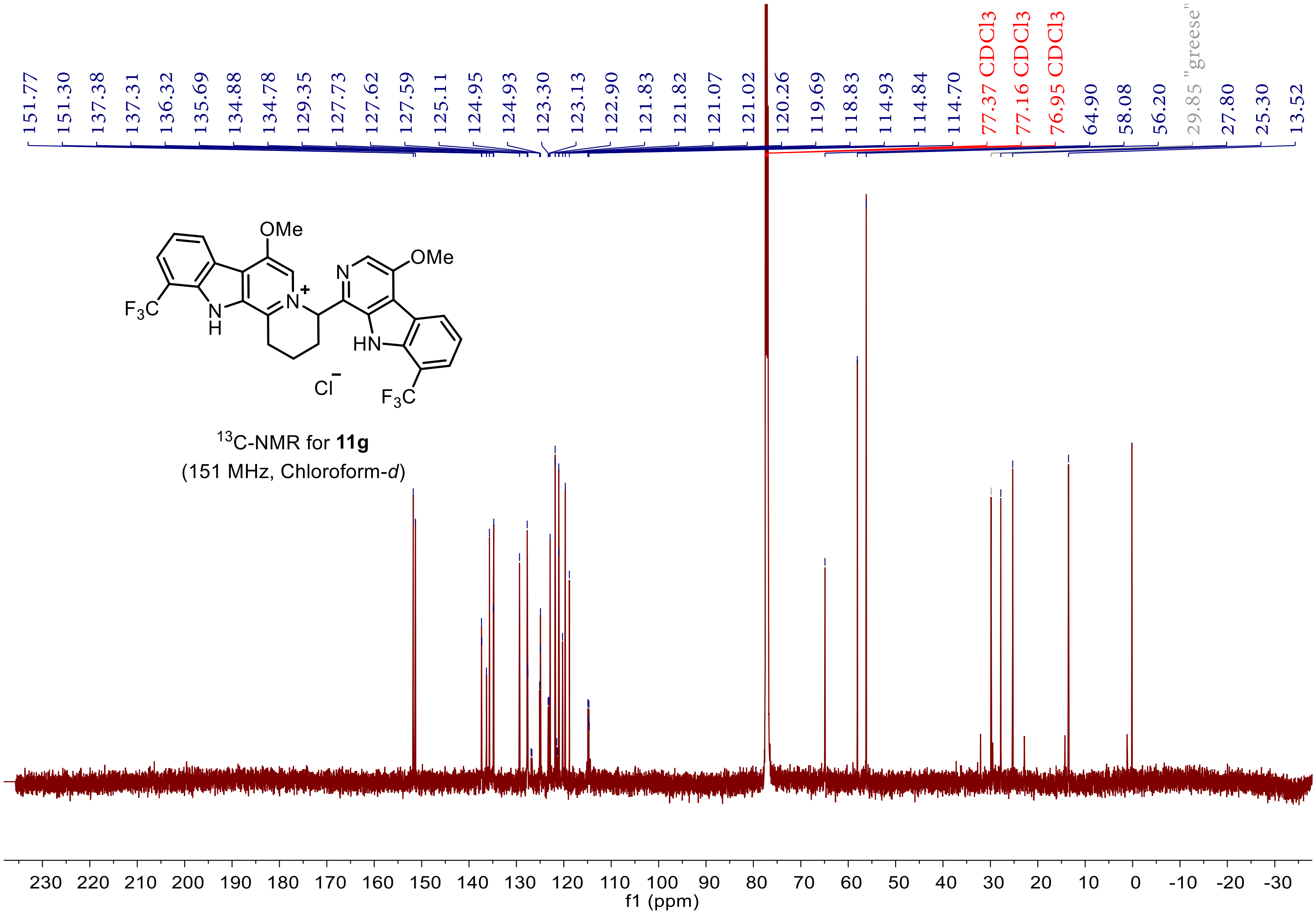
151.77
151.30
137.38
137.31
136.32
135.69
134.88
134.78
129.35
127.73
127.62
127.59
125.11
124.95
124.93
123.30
123.13
122.90
121.83
121.82
121.07
121.02
120.26
119.69
118.83
114.93
114.84
114.70
77.37 CDCl3
77.16 CDCl3
76.95 CDCl3
64.90
58.08
56.20
29.85 "greese"
27.80
25.30
13.52
OMe
OMe
N
N+
F3C
N H
HN
Cl−
F3C
13C-NMR for 11g
(151 MHz, Chloroform-d)
230 220 210 200 190 180 170 160 150 140 130 120 110 100 90 80 70 60 50 40 30 20 10 0 -10 -20 -30
f1 (ppm)

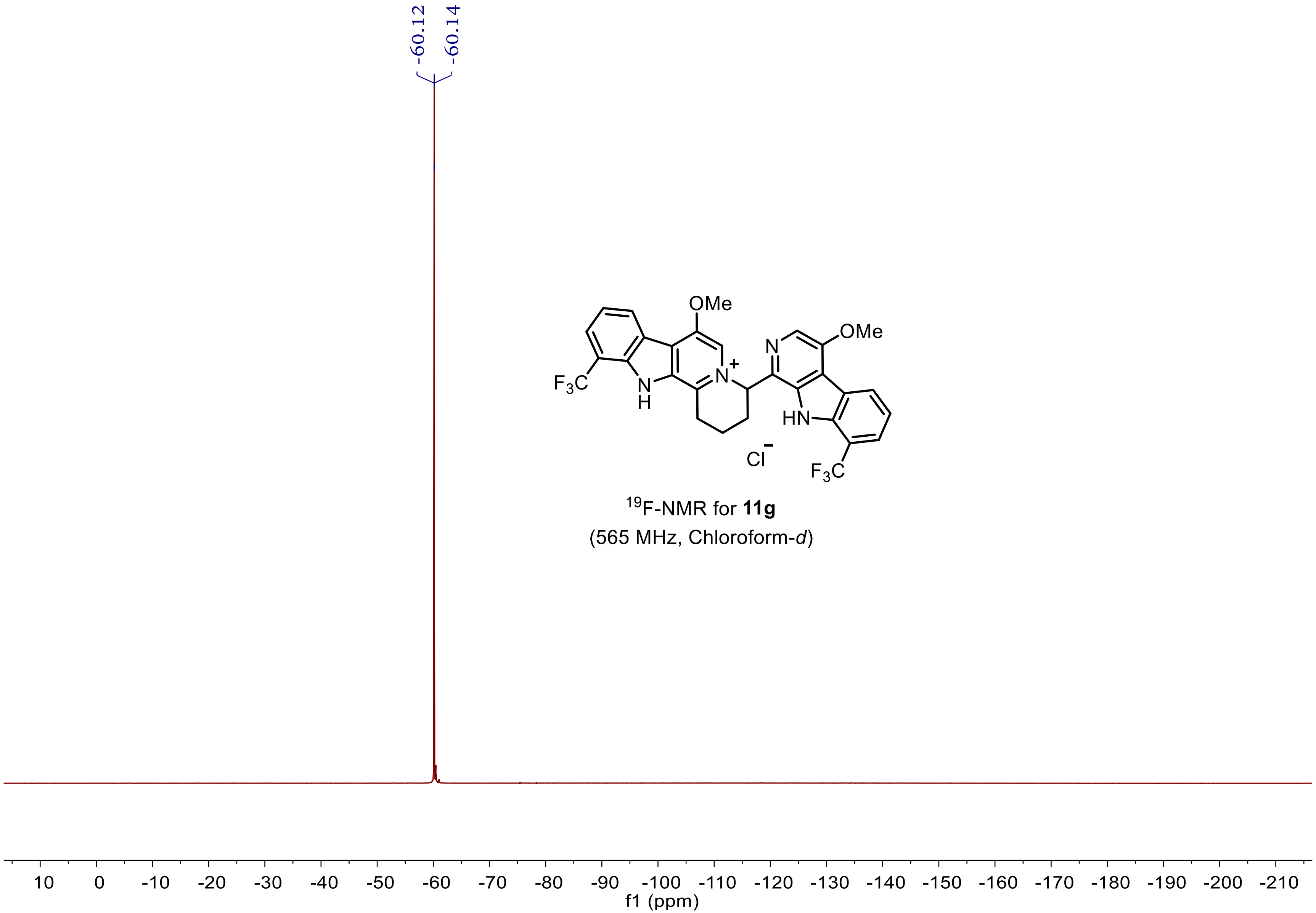

^{19}F-NMR for **11g**
(565 MHz, Chloroform-*d*)

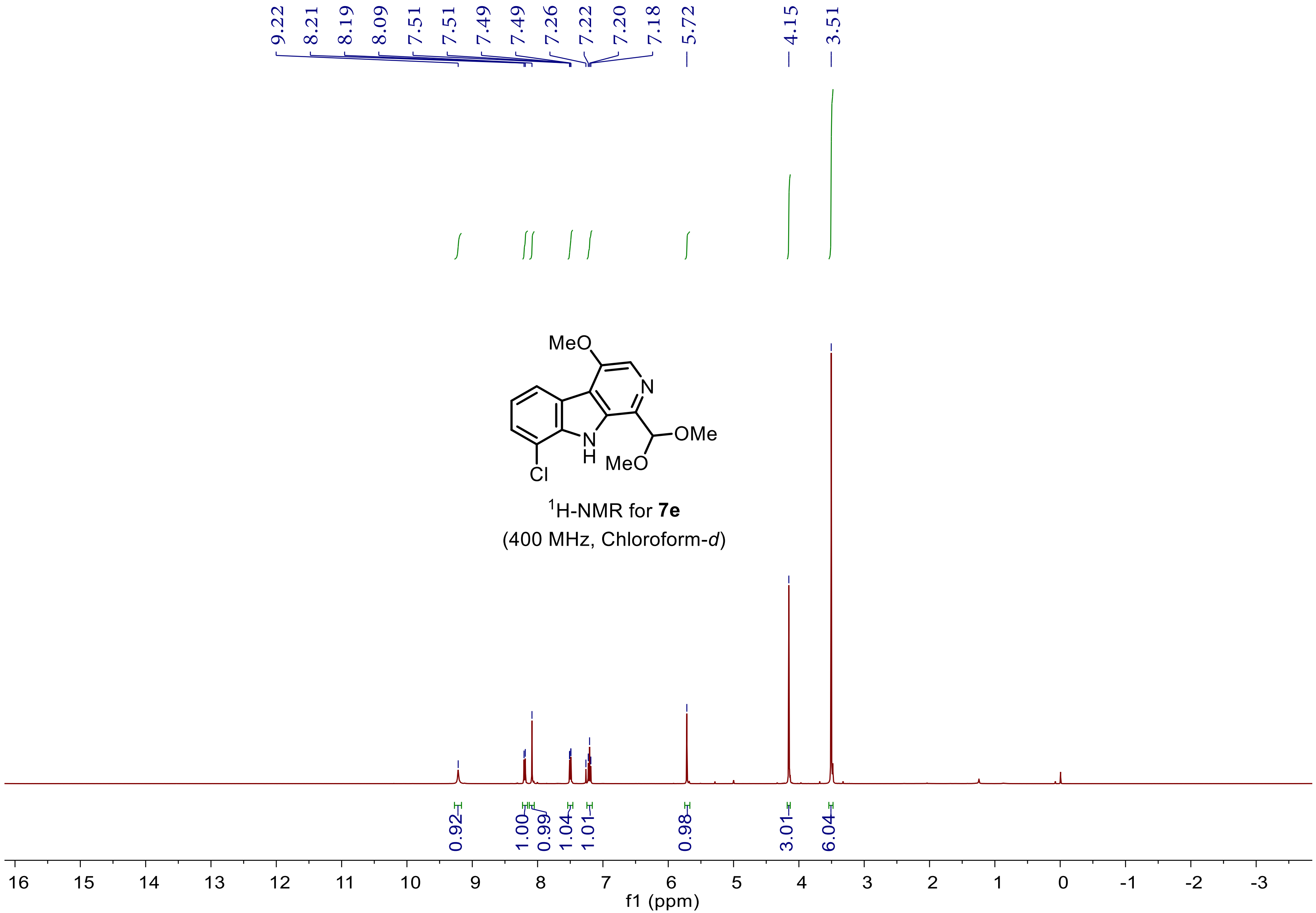
9.22
8.21
8.19
8.09
7.51
7.51
7.49
7.49
7.26
7.22
7.20
7.18
5.72
4.15
3.51
MeO
N
OMe
MeO
N
H
Cl
^{1}H-NMR for **7e**
(400 MHz, Chloroform-*d*)
0.92
1.00
0.99
1.04
1.01
0.98
3.01
6.04
16
15
14
13
12
11
10
9
8
7
6
5
4
3
2
1
0
-1
-2
-3
f1 (ppm)

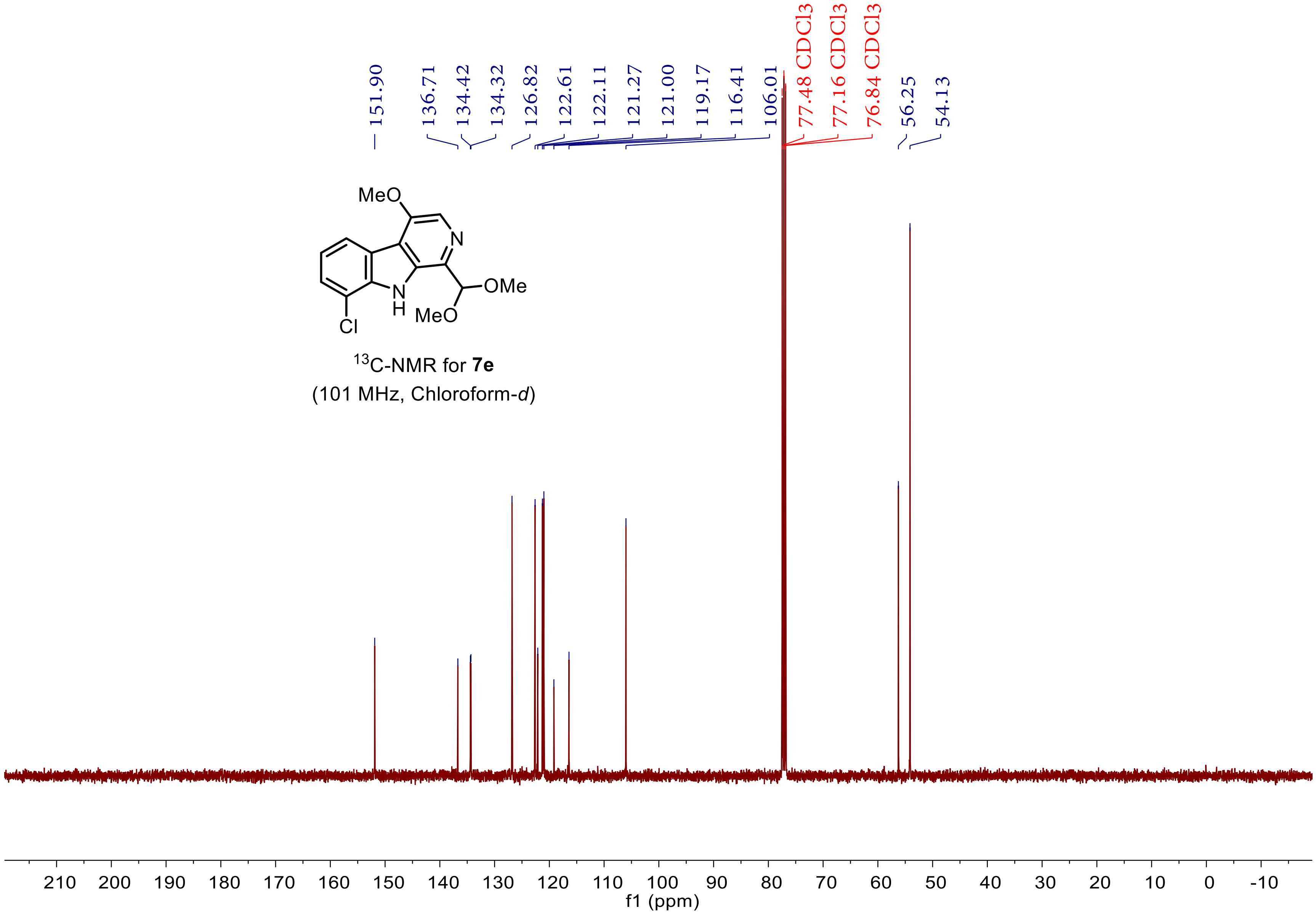

^{13}C-NMR for **7e**

(101 MHz, Chloroform-*d*)

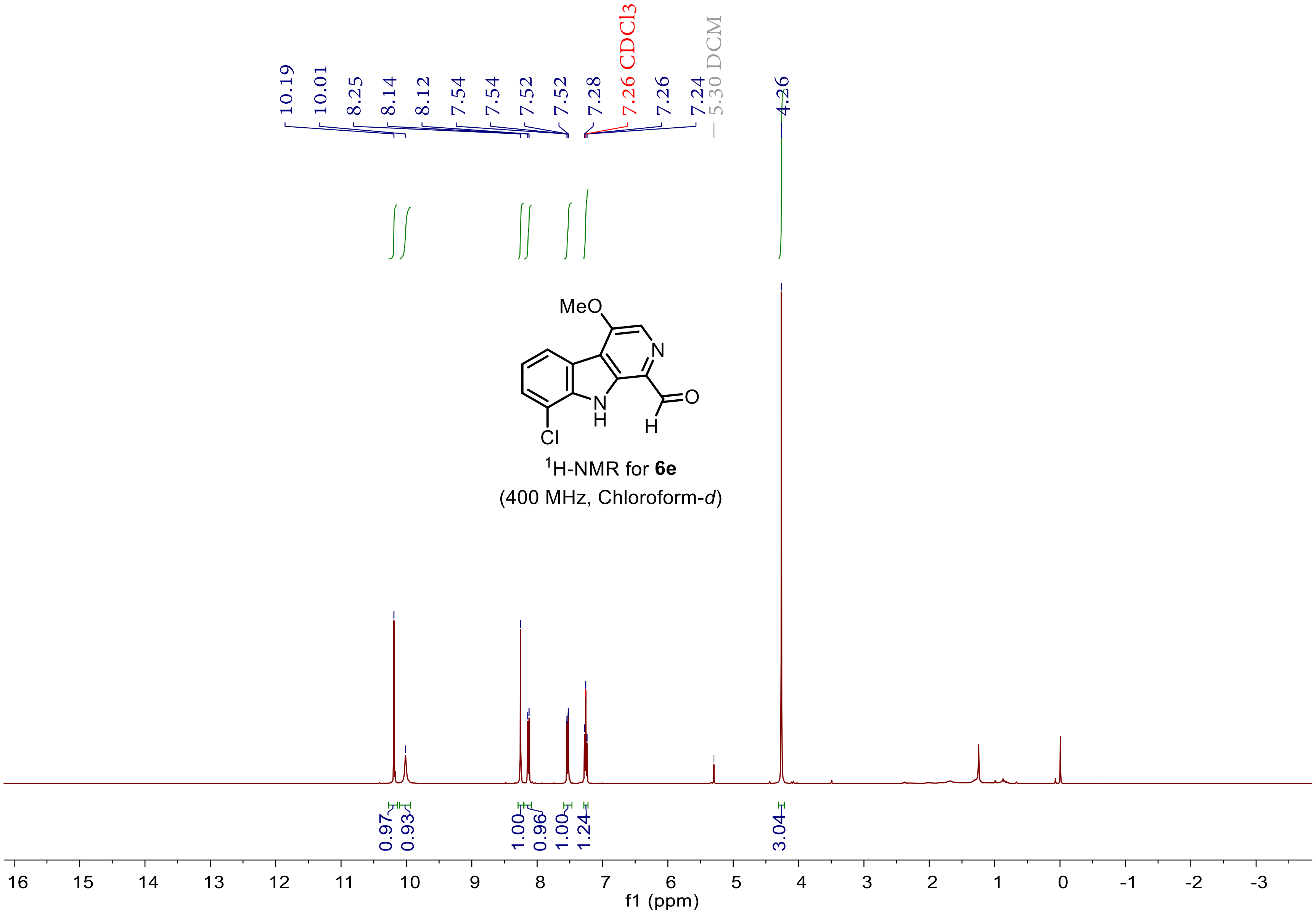

^{1}H-NMR for **6e**
(400 MHz, Chloroform-*d*)

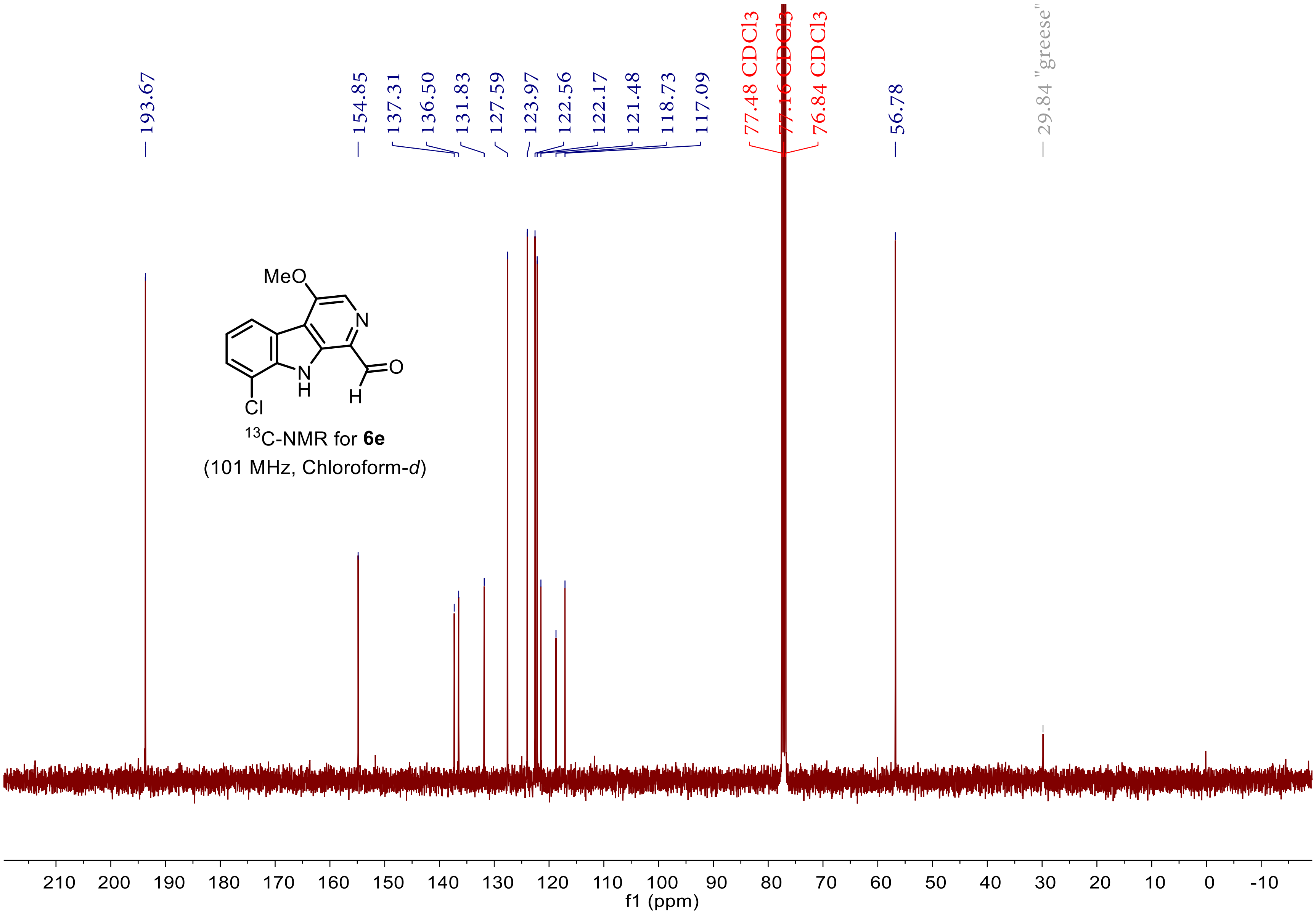
193.67
154.85
137.31
136.50
131.83
127.59
123.97
122.56
122.17
121.48
118.73
117.09
77.48 CDCl3
77.16 CDCl3
76.84 CDCl3
56.78
29.84 "greese"
MeO
N
O
N
H
H
Cl
^{13}C-NMR for **6e**
(101 MHz, Chloroform-*d*)
210 200 190 180 170 160 150 140 130 120 110 100 90 80 70 60 50 40 30 20 10 0 -10
f1 (ppm)

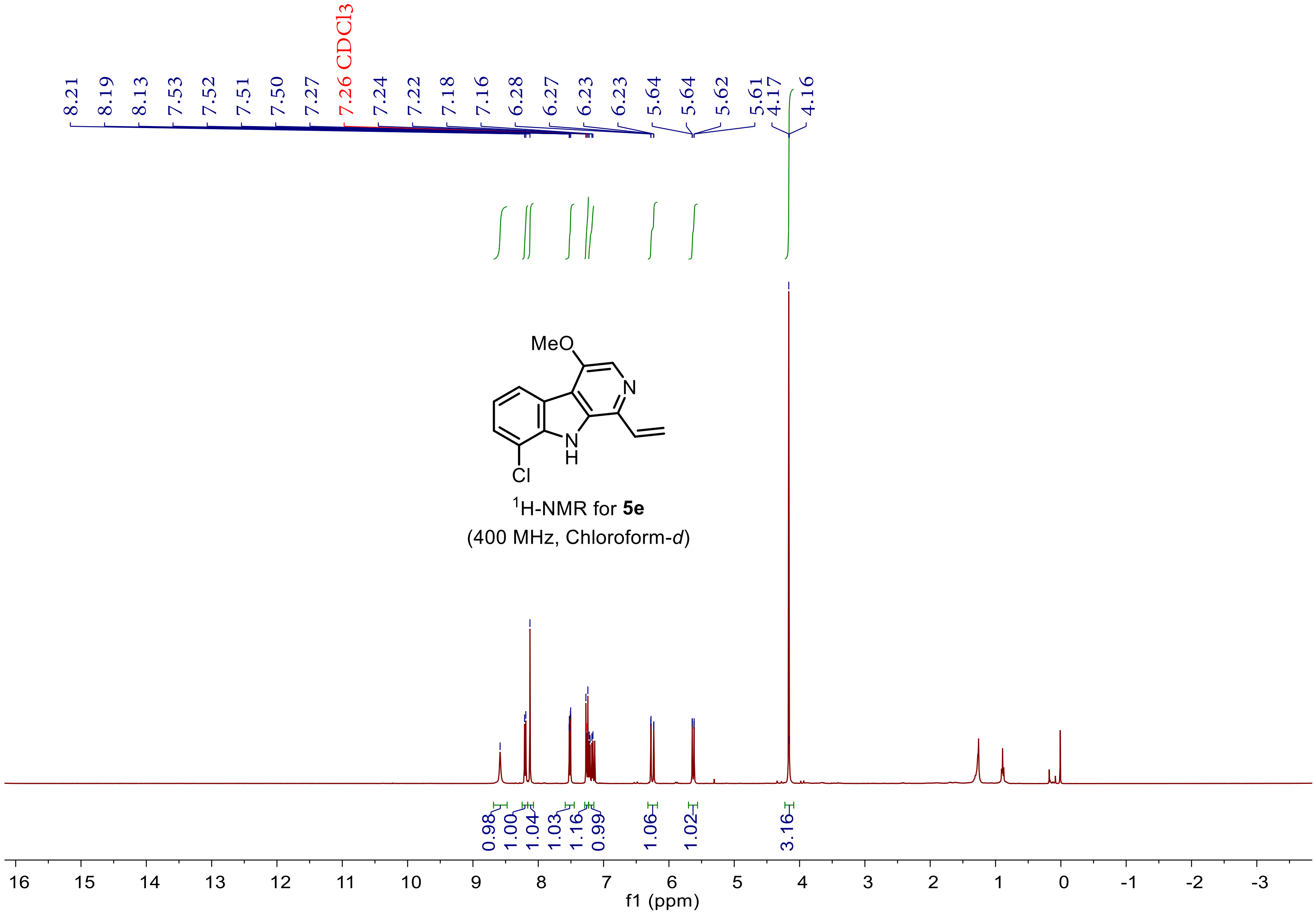

^{1}H-NMR for **5e**
(400 MHz, Chloroform-*d*)

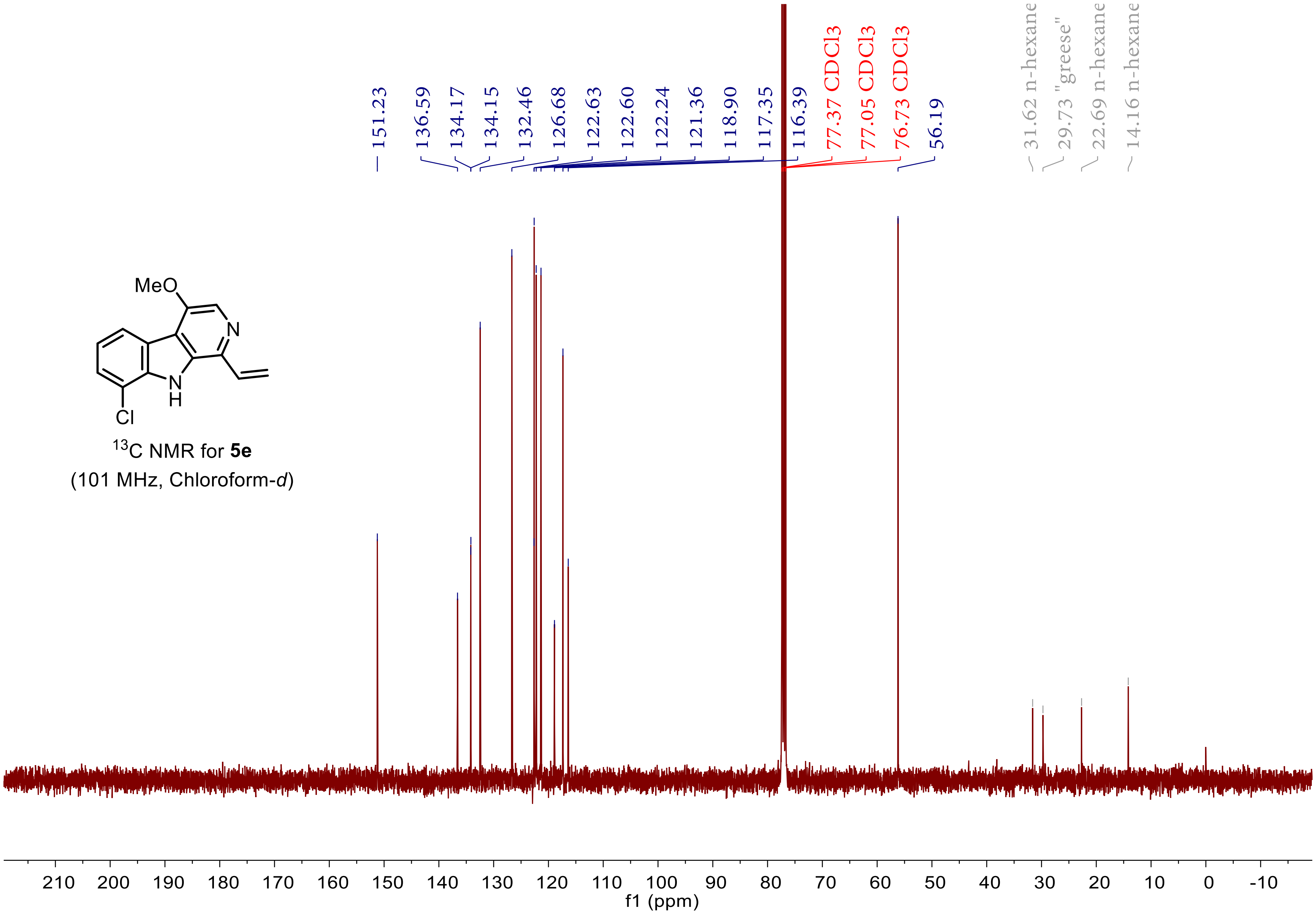

^{13}C NMR for **5e**
(101 MHz, Chloroform-*d*)

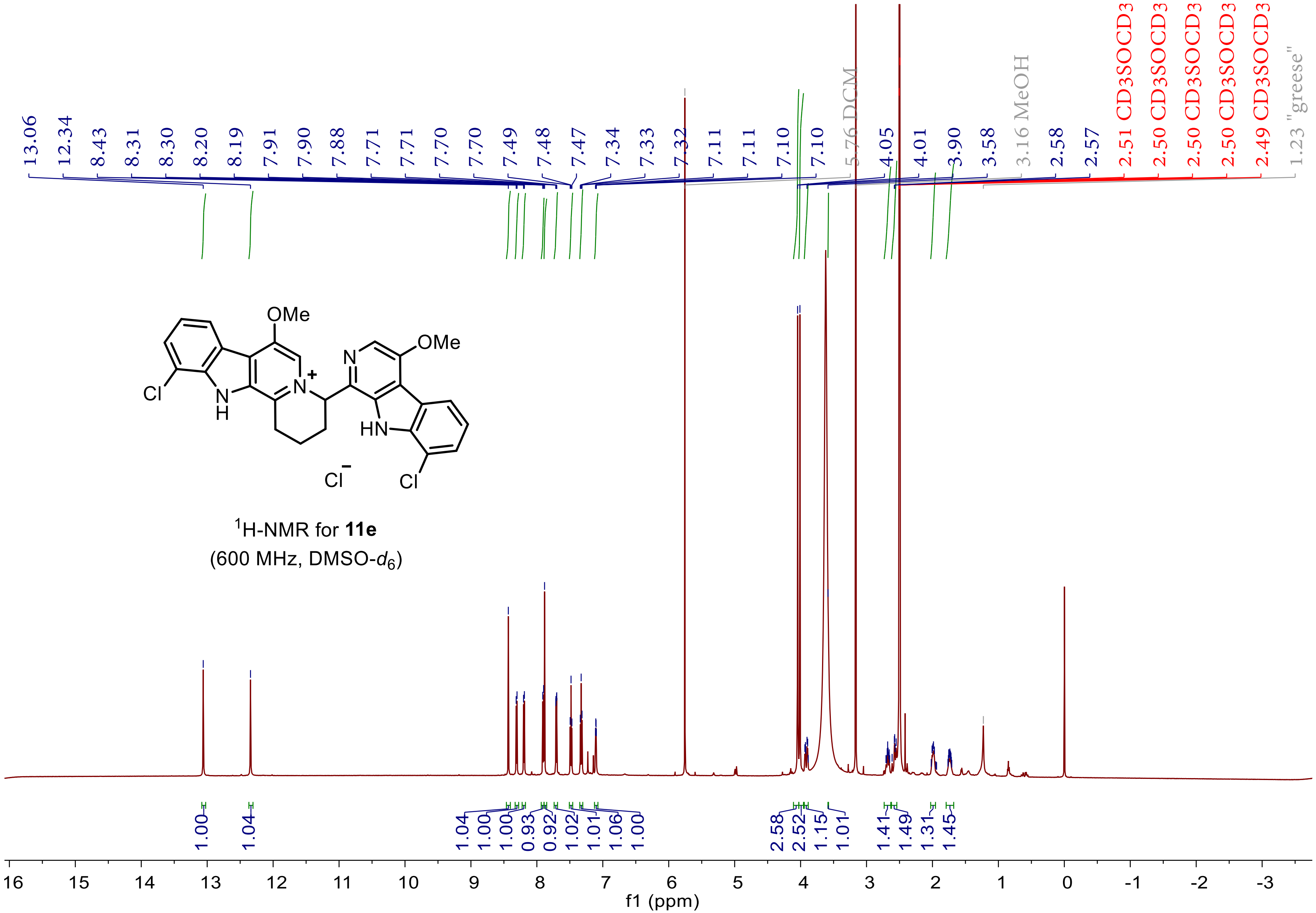

^{1}H-NMR for **11e**
(600 MHz, DMSO-d_6)

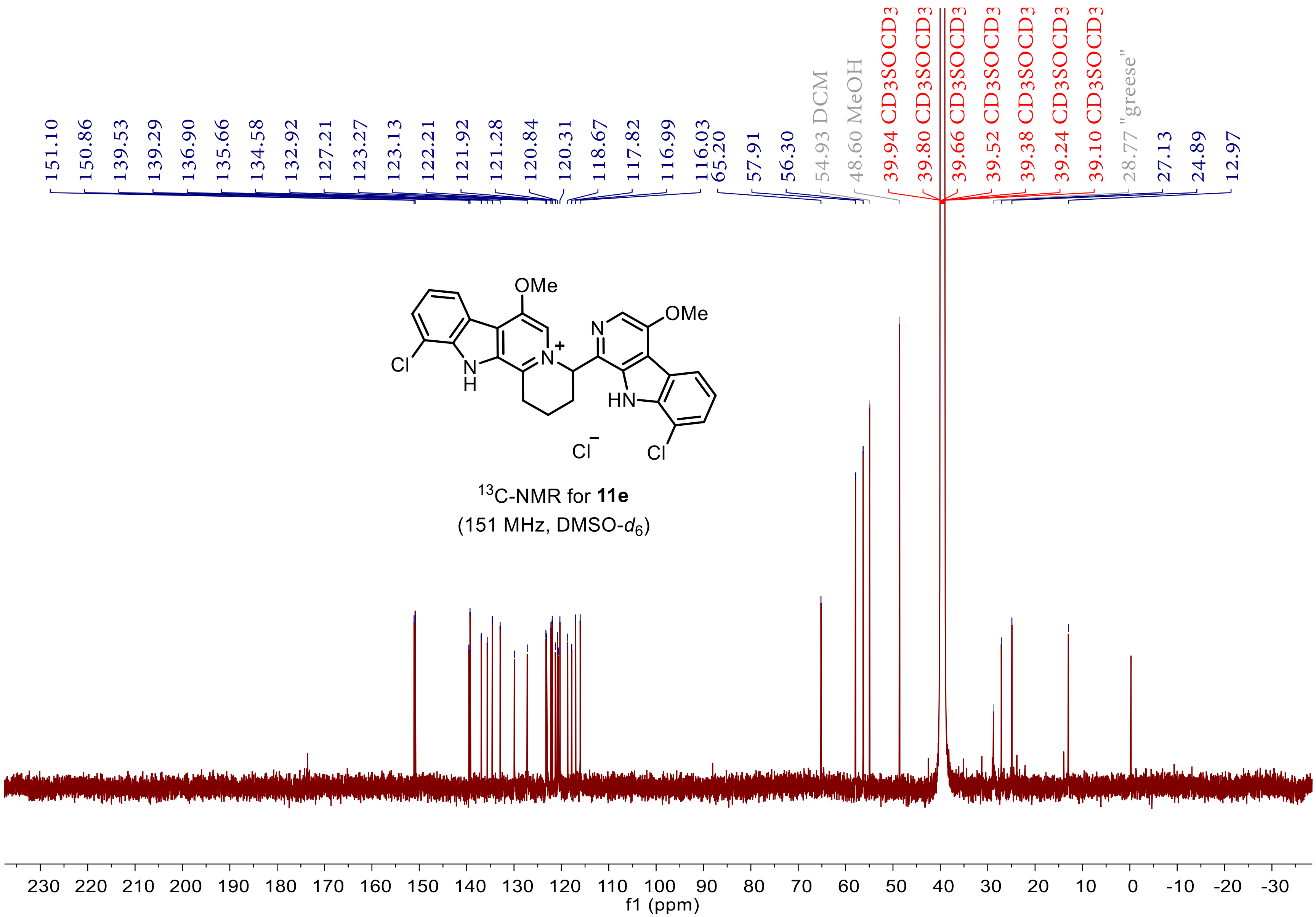

^{13}C-NMR for **11e**

(151 MHz, DMSO-d_6)

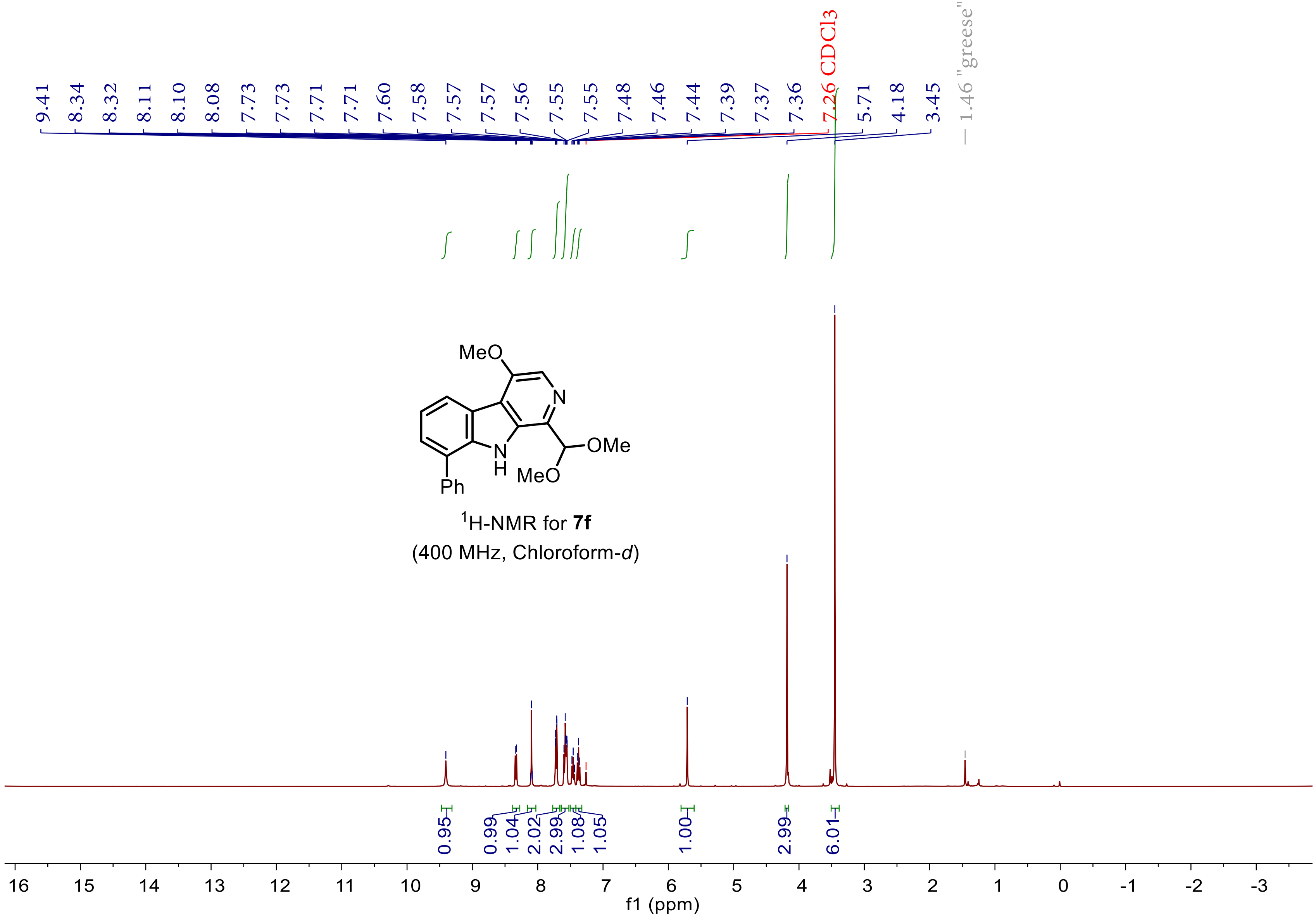

^{1}H-NMR for **7f**

(400 MHz, Chloroform-*d*)

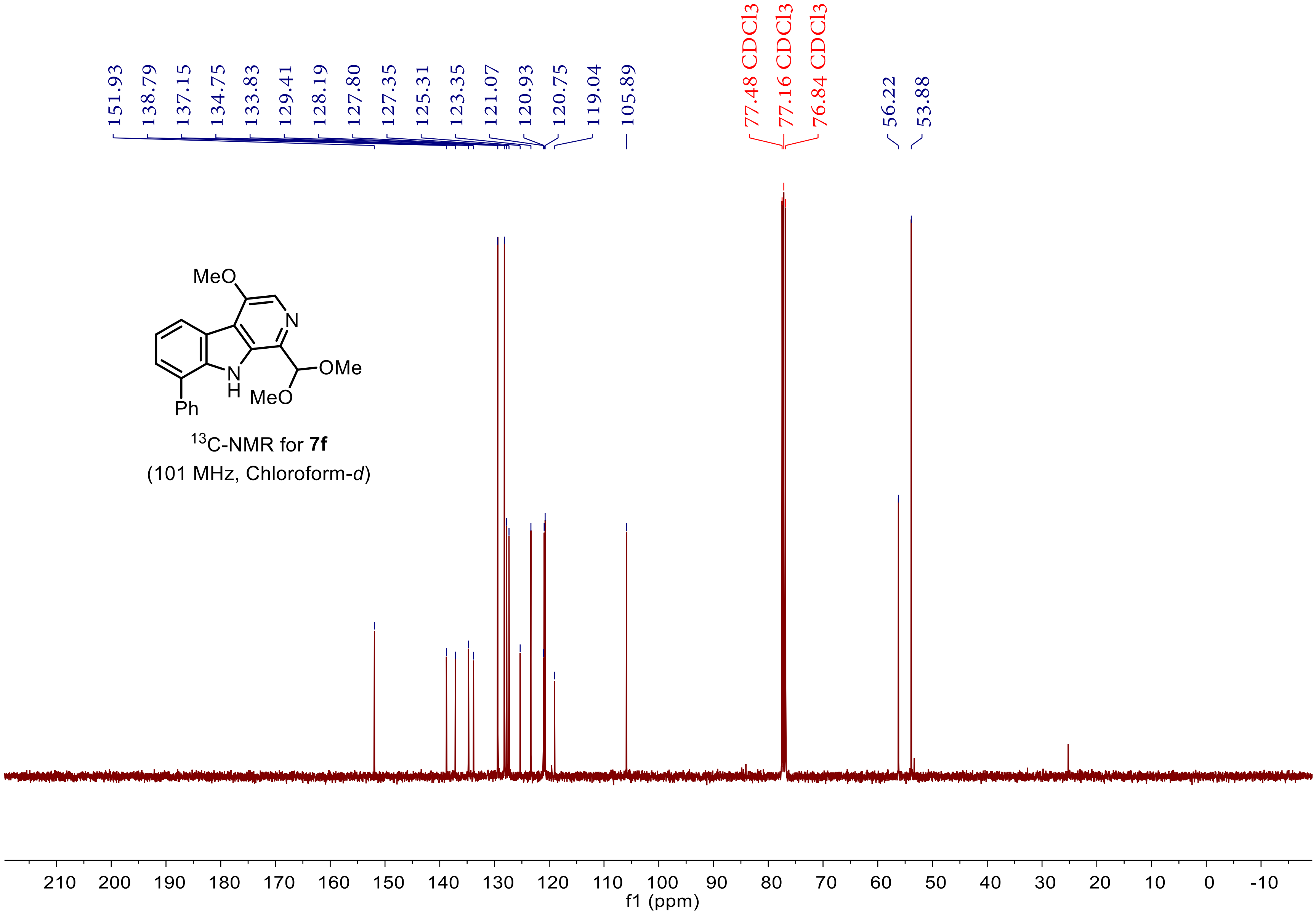
151.93
138.79
137.15
134.75
133.83
129.41
128.19
127.80
127.35
125.31
123.35
121.07
120.93
120.75
119.04
105.89
77.48 CDCl3
77.16 CDCl3
76.84 CDCl3
56.22
53.88
MeO
N
OMe
N
H
MeO
Ph
^{13}C-NMR for **7f**
(101 MHz, Chloroform-*d*)
210 200 190 180 170 160 150 140 130 120 110 100 90 80 70 60 50 40 30 20 10 0 -10
f1 (ppm)

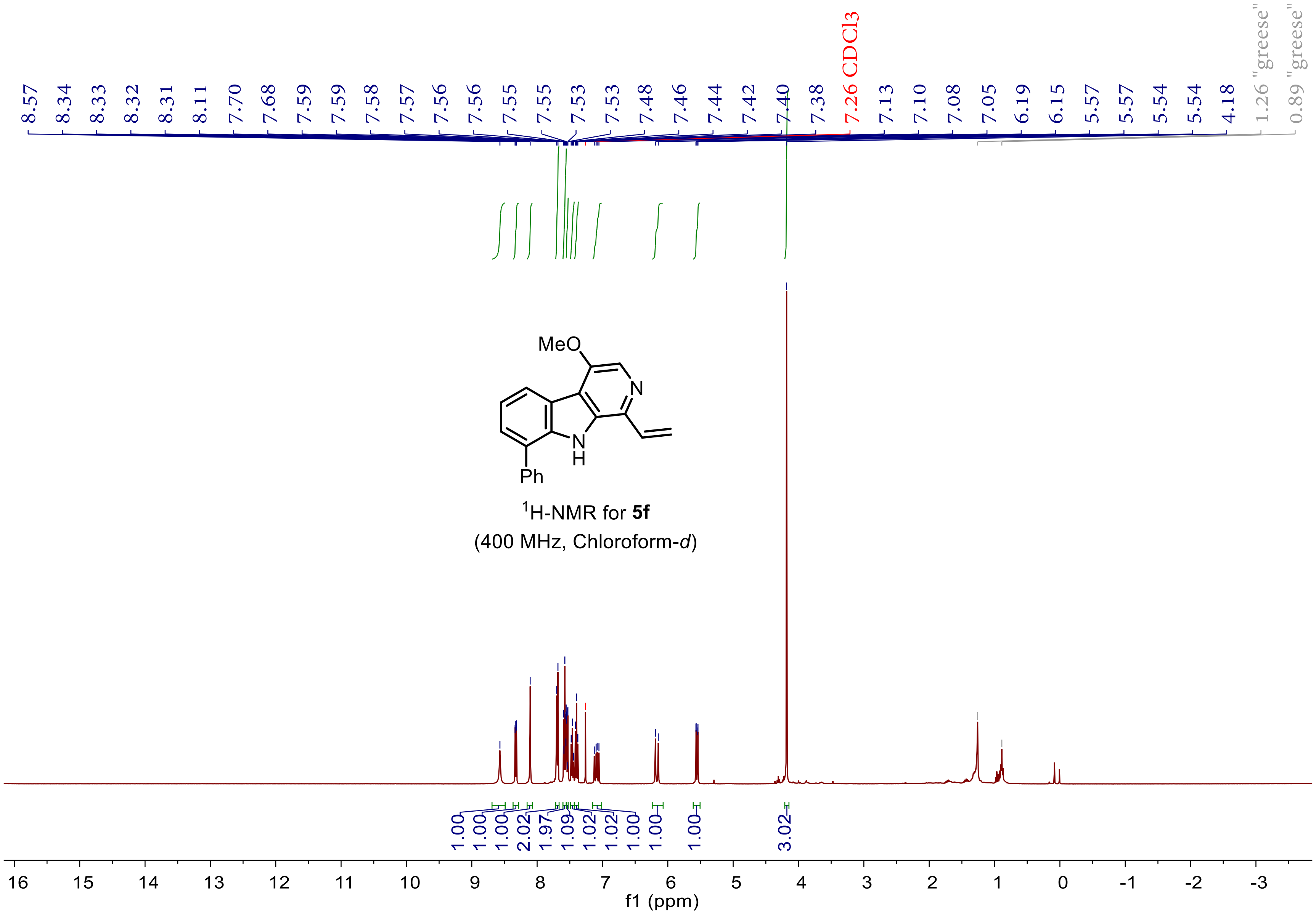

^{1}H-NMR for **5f**

(400 MHz, Chloroform-*d*)

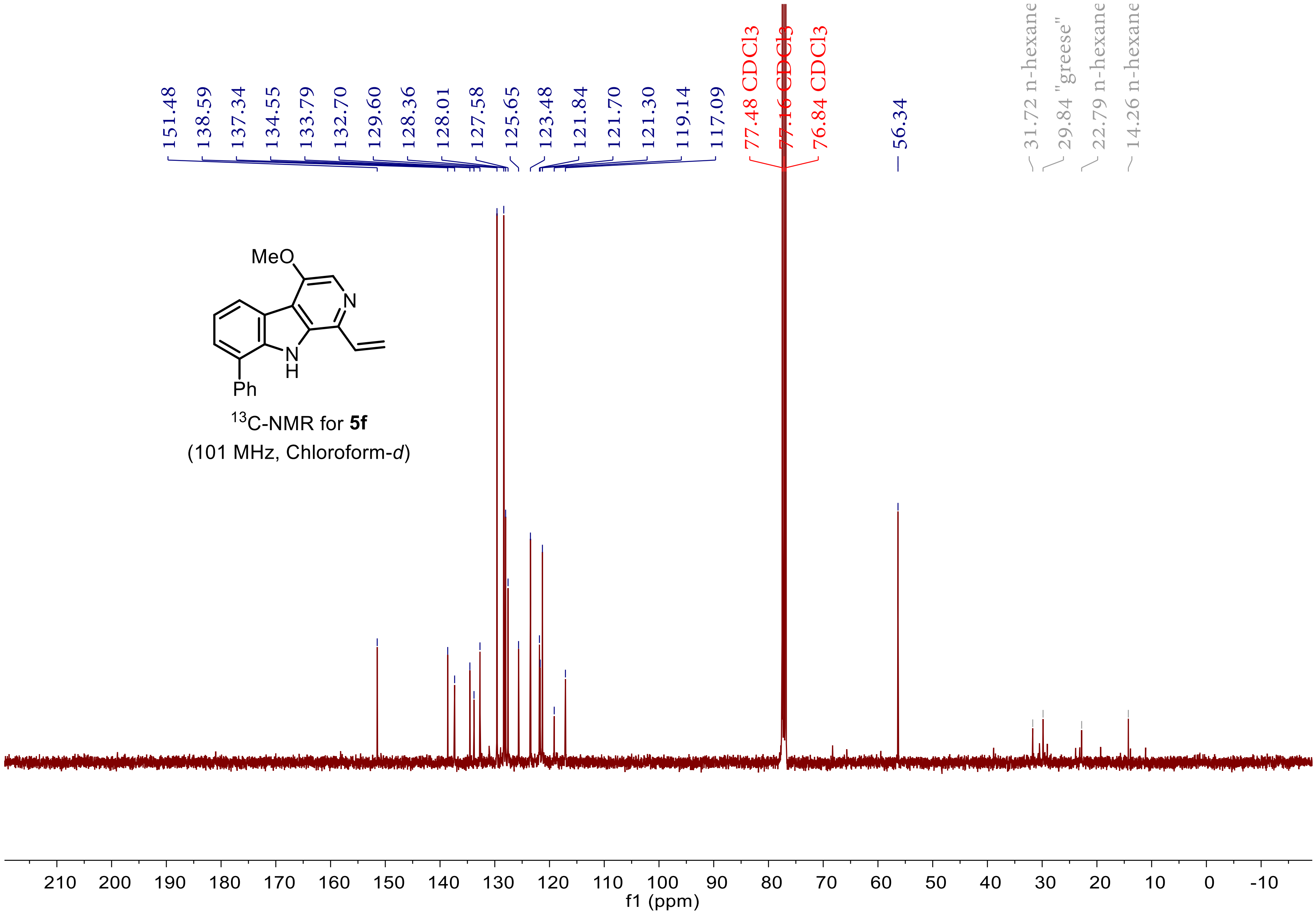
151.48
138.59
137.34
134.55
133.79
132.70
129.60
128.36
128.01
127.58
125.65
123.48
121.84
121.70
121.30
119.14
117.09
77.48 CDCl3
77.16 CDCl3
76.84 CDCl3
56.34
31.72 n-hexane
29.84 "greese"
22.79 n-hexane
14.26 n-hexane
MeO
N
N
H
Ph
^{13}C-NMR for **5f**
(101 MHz, Chloroform-*d*)
210 200 190 180 170 160 150 140 130 120 110 100 90 80 70 60 50 40 30 20 10 0 -10
f1 (ppm)

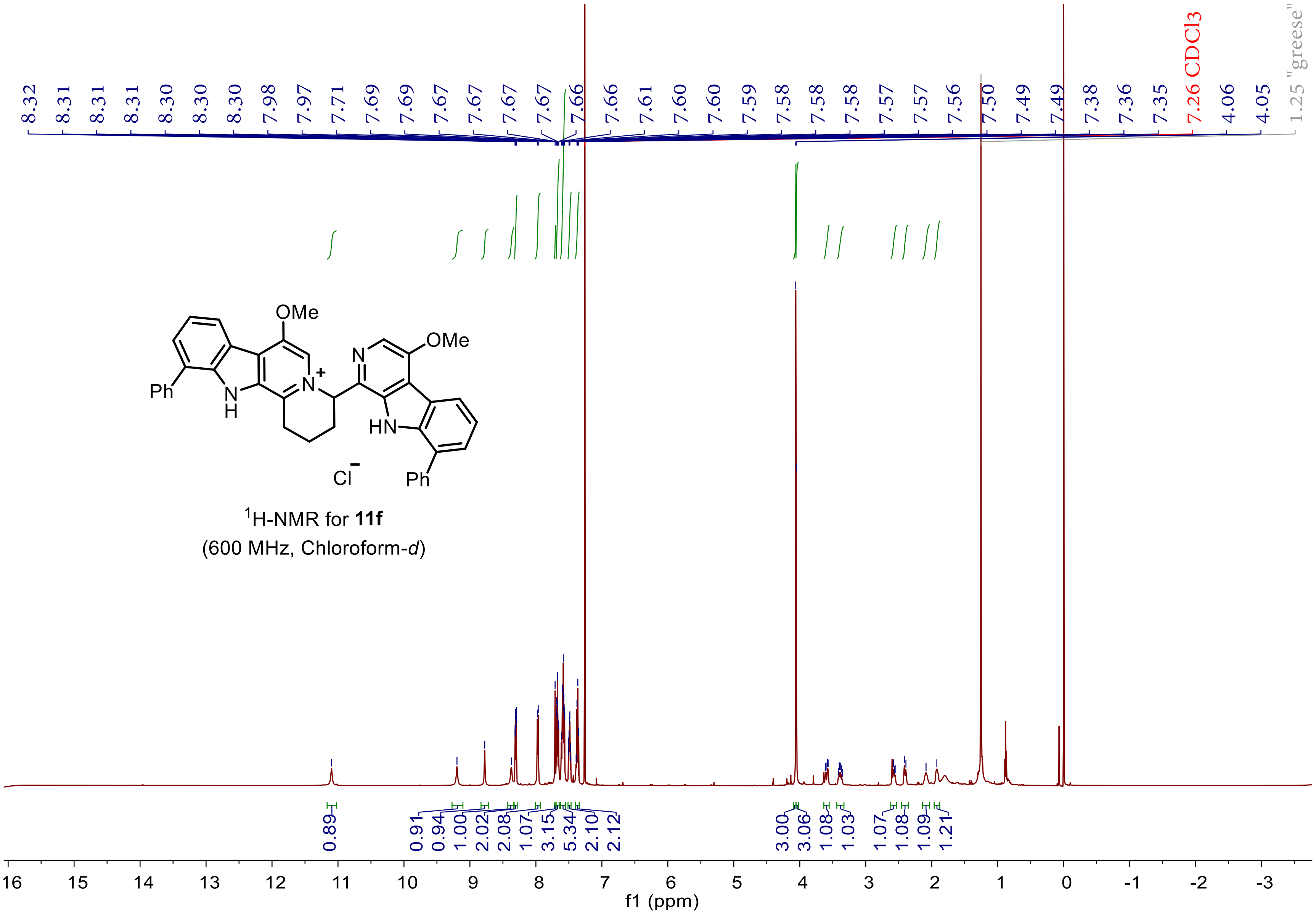

^{1}H-NMR for **11f**
(600 MHz, Chloroform-*d*)

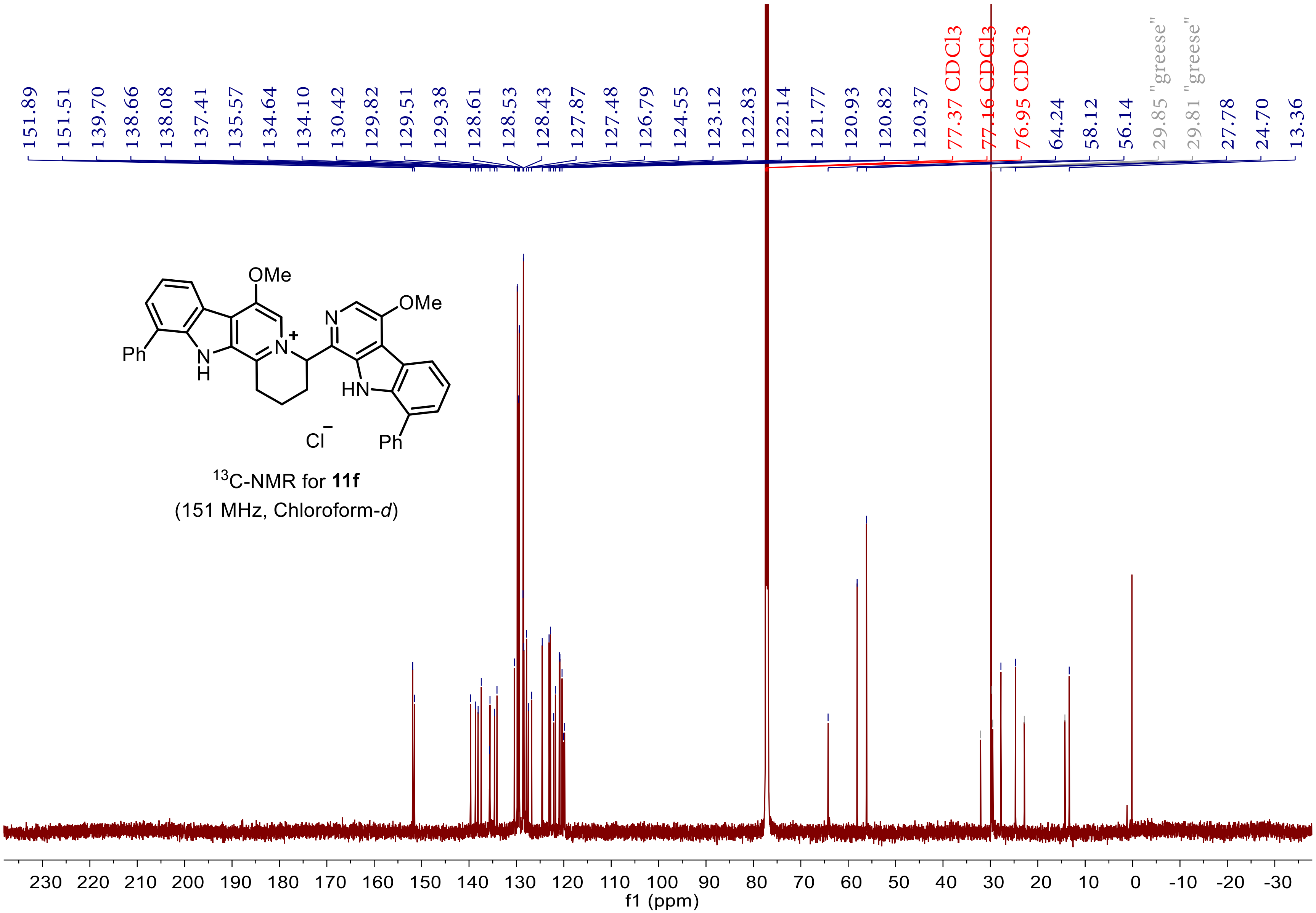

^{13}C-NMR for **11f**
(151 MHz, Chloroform-*d*)

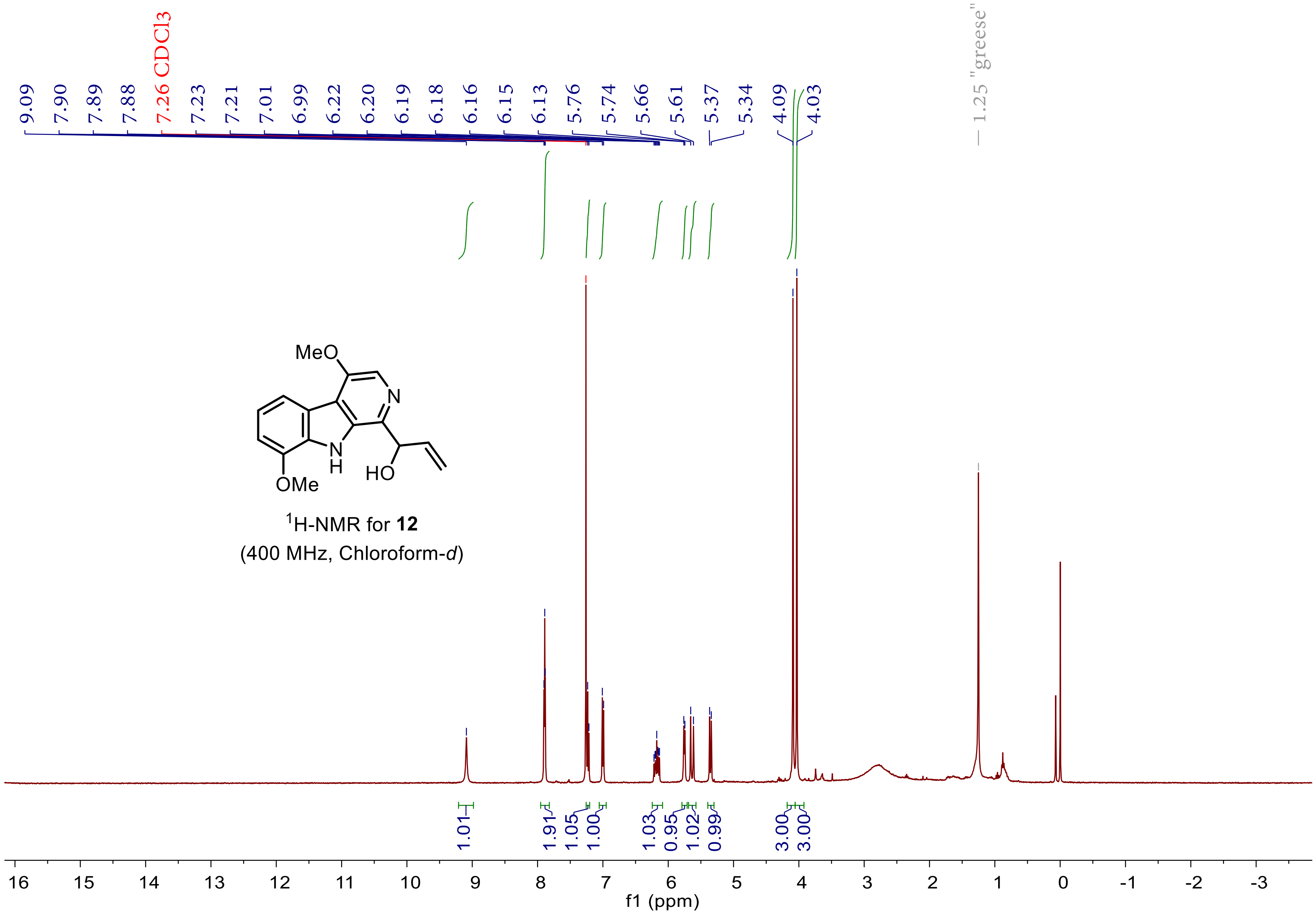

^{1}H-NMR for **12**
(400 MHz, Chloroform-*d*)

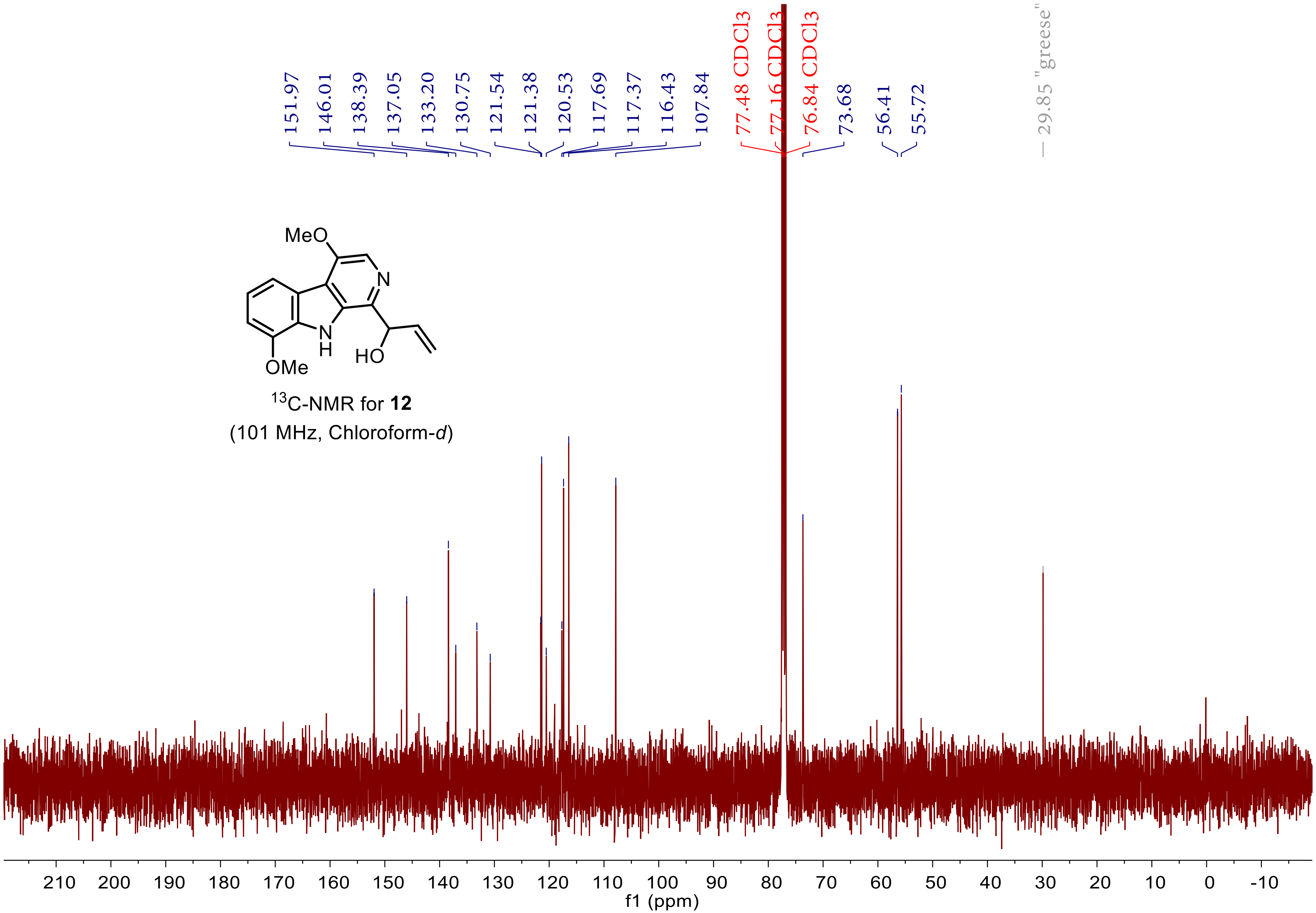
151.97
146.01
138.39
137.05
133.20
130.75
121.54
121.38
120.53
117.69
117.37
116.43
107.84
77.48 CDCl3
77.16 CDCl3
76.84 CDCl3
73.68
56.41
55.72
29.85 "greese"
MeO
N
N
H
HO
OMe
^{13}C-NMR for **12**
(101 MHz, Chloroform-*d*)
210 200 190 180 170 160 150 140 130 120 110 100 90 80 70 60 50 40 30 20 10 0 -10
f1 (ppm)

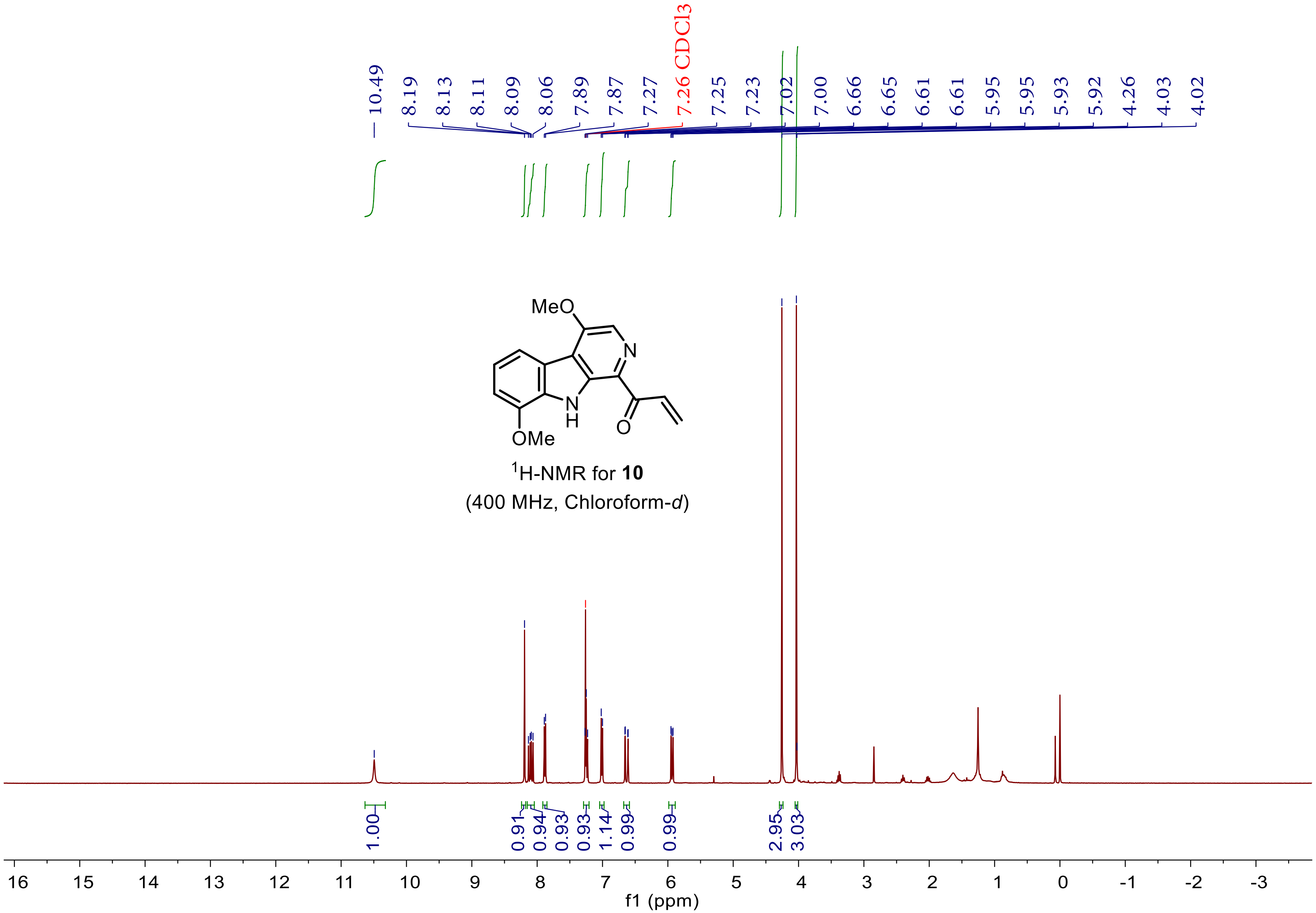

^{1}H-NMR for **10**
(400 MHz, Chloroform-*d*)

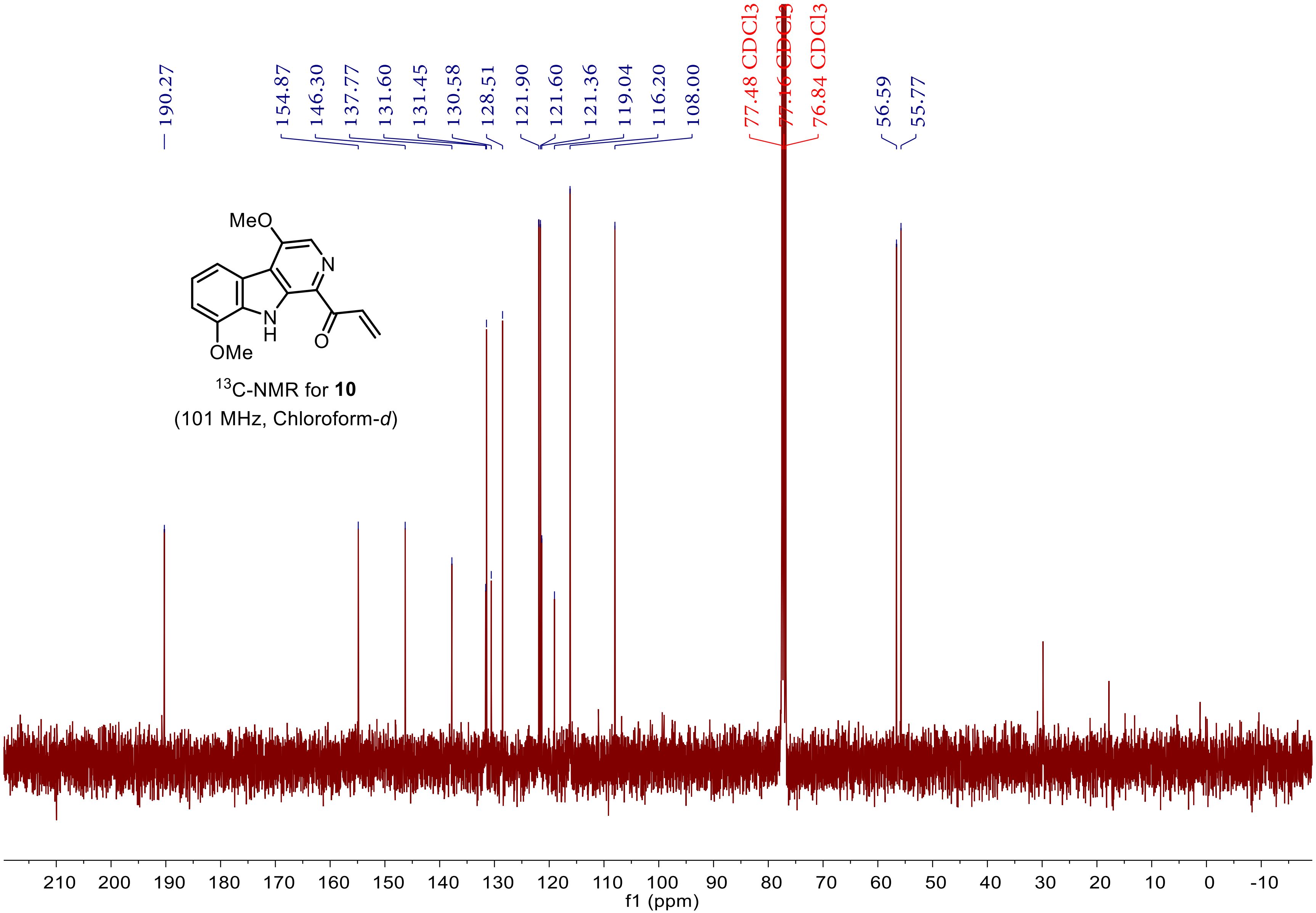

^{13}C-NMR for **10**

(101 MHz, Chloroform-*d*)

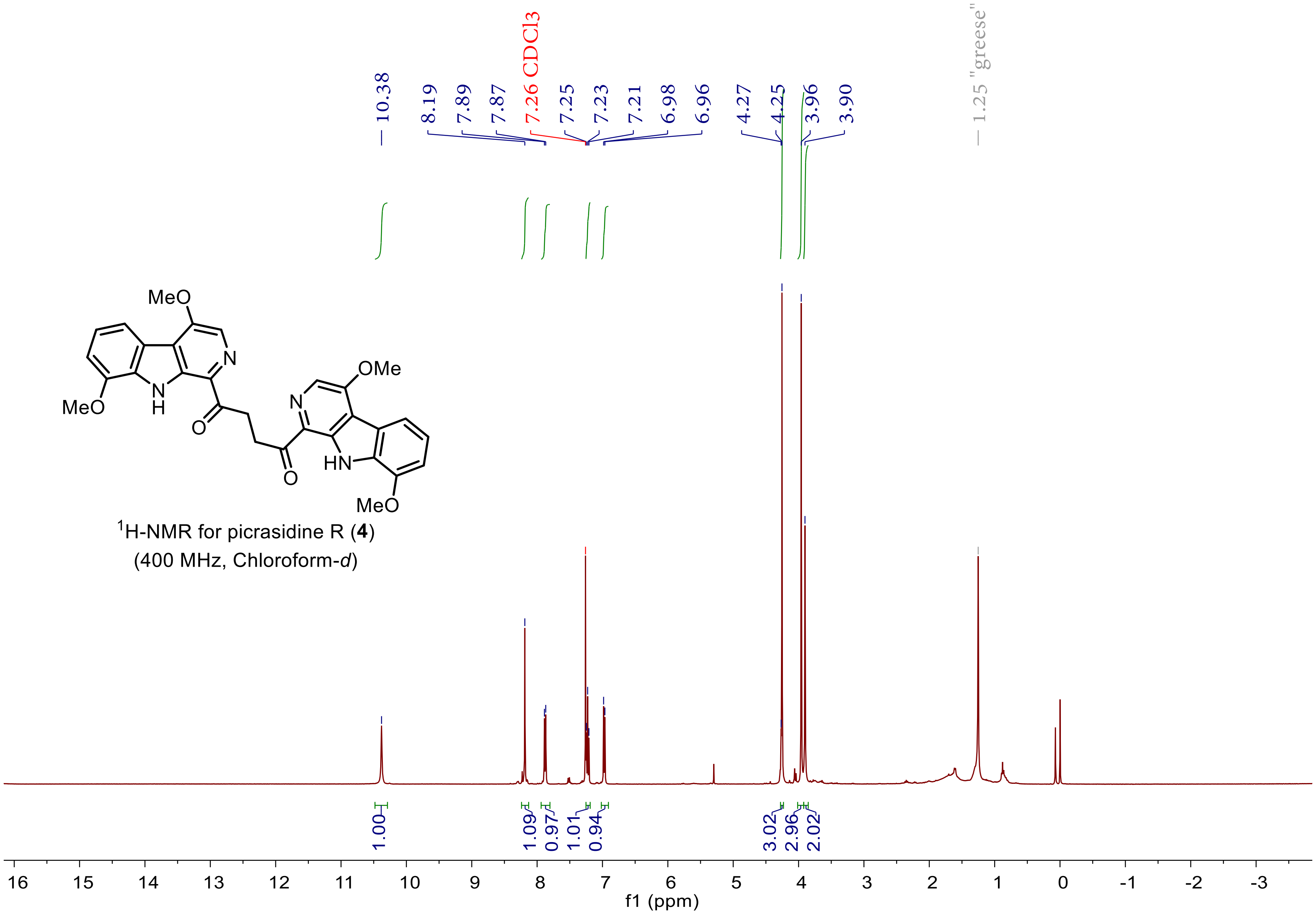

^{1}H-NMR for picrasidine R (**4**)
(400 MHz, Chloroform-*d*)

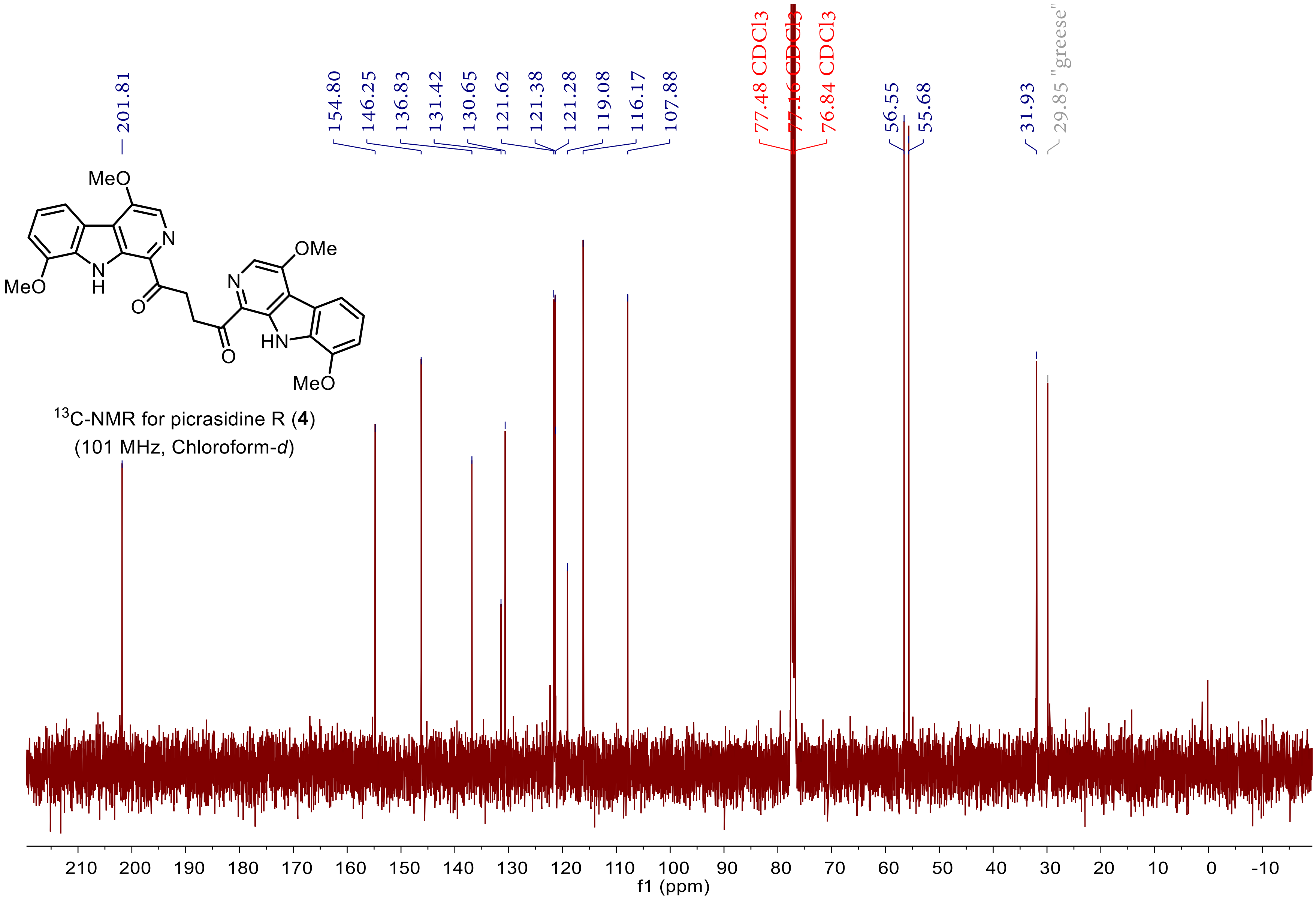

^{13}C-NMR for picrasidine R (**4**)
(101 MHz, Chloroform-*d*)

5. Computational Results

dehydrocrenatine (**5a**):

G[WB97XD/6-311++G(2d,p) // WB97XD/6-31G(d), 403.15K] = -725.206377 hartree.

E[WB97XD/6-311++G(2d,p) // WB97XD/6-31G(d), 403.15K] = -725.380603 hartree.

C	3.85545300	0.17716200	-0.00005700
C	3.92372400	-1.22515600	0.00004900
C	2.77815700	-2.00737100	0.00009700
C	1.54949900	-1.34710300	0.00004000
C	1.46354900	0.06202900	-0.00005800
C	2.63158500	0.82880100	-0.00011300
N	0.27003800	-1.86997200	0.00005100
C	-0.64842000	-0.83825900	-0.00000300
C	0.05432800	0.37762700	-0.00008300
C	-2.05463300	-0.86496600	0.00001300
N	-2.72099500	0.28644300	-0.00005200
C	-2.07109900	1.45697300	-0.00013900
C	-0.68046100	1.56669200	-0.00014000
C	-2.81103600	-2.12833000	0.00008800
C	-4.14134500	-2.20979000	-0.00002900
O	0.02018200	2.72418500	-0.00022600
C	-0.70949900	3.93380400	0.00036400
H	4.77403600	0.75542700	-0.00009800
H	4.89515400	-1.71065000	0.00009000
H	2.83744800	-3.09159000	0.00017400
H	2.56841000	1.91131300	-0.00020000
H	0.04932000	-2.85075100	0.00022200
H	-2.70269300	2.34002000	-0.00021200
H	-2.22633200	-3.04745100	0.00024300
H	-4.74946900	-1.31079300	-0.00018500
H	-4.64673200	-3.17005000	0.00004000
H	-1.33790100	4.01994100	0.89531500
H	0.03302600	4.73226200	0.00078600
H	-1.33783600	4.02086700	-0.89454200

protonated dehydrocrenatine (**5a**·H^+):

G[WB97XD/6-311++G(2d,p) // WB97XD/6-31G(d), 403.15K] = -725.585706 hartree.

E[WB97XD/6-311++G(2d,p) // WB97XD/6-31G(d), 403.15K] = -725.774755 hartree.

C	-2.62634700	-3.20799900	0.48659100
C	-2.91470100	-2.09379500	-0.18837600
C	-2.10402700	-0.87269900	-0.12627500
C	-0.71284600	-0.80311800	-0.07453400
C	-0.02797300	0.43337500	0.00162900
C	-0.76336200	1.63485200	0.01450100
C	-2.14167800	1.54313400	-0.06807000
N	-2.73491600	0.31923400	-0.13773400
N	0.21018400	-1.81221100	-0.10278500
C	1.48121800	-1.26399000	-0.06670000
C	1.37169900	0.14202900	0.01509600
C	2.71854500	-1.90788700	-0.09646600
C	3.84304300	-1.10385900	-0.03858000
C	3.75403200	0.30071900	0.04833200
C	2.52955700	0.93544600	0.07596700
O	-0.07271700	2.77145700	0.08941700
C	-0.78344100	4.00618800	0.09165800
H	-3.26266500	-4.08256700	0.40788600
H	-1.77869500	-3.27670800	1.16334100
H	-3.80488400	-2.05217300	-0.81358500
H	-2.80969000	2.39182200	-0.08114700
H	-3.74820600	0.30295400	-0.16907600
H	0.00113200	-2.78170300	-0.28377200
H	2.79816800	-2.98769000	-0.16135800
H	4.82338700	-1.56862900	-0.05957200
H	4.66571200	0.88611900	0.09355700
H	2.45221100	2.01406200	0.14198400
H	-1.45190300	4.06692600	0.95743700
H	-0.02401700	4.78228800	0.16292800
H	-1.34800500	4.13011800	-0.83896400

TS_A-1:

G[WB97XD/6-311++G(2d,p) // WB97XD/6-31G(d), 403.15K] = -1450.755881 hartree.

E[WB97XD/6-311++G(2d,p) // WB97XD/6-31G(d), 403.15K] = -1451.158862 hartree.

C	-1.19378800	-3.89588000	-1.10740500
C	-2.25373500	-3.12090400	-1.60545700
C	-2.08819000	-1.77936300	-1.91577700
C	-0.82695900	-1.23152900	-1.70115100
C	0.24381600	-1.98710800	-1.18764900
C	0.05944500	-3.34205500	-0.89811200
N	-0.38828900	0.07203400	-1.90964500
C	0.91598800	0.18330200	-1.53605900
C	1.35304300	-1.07039000	-1.07017100
C	1.76119700	1.32466500	-1.46320100
N	3.00424300	1.18209800	-0.94142200
C	3.42898600	0.00405200	-0.51364200
C	2.63926800	-1.16625700	-0.54210100
C	1.33088300	2.63348100	-1.77908400
C	2.04656000	3.74677200	-1.33647700
C	1.66935100	3.92925900	0.52080400
C	1.91168100	2.75754400	1.26328700
C	1.02074100	1.68196000	1.46041600
C	-0.35620800	1.57473200	1.10190100
C	-1.08382300	0.39439000	1.27085500
C	-0.47368600	-0.74985900	1.84968800
C	0.82414400	-0.62840000	2.26049400
N	1.50268300	0.54800100	2.04976300
N	-1.17308600	2.50431100	0.49127500
C	-2.42642000	1.95091900	0.30096000
C	-2.40535700	0.61905400	0.76029500
C	-3.56653500	2.52140300	-0.26979300
C	-4.68796200	1.71621400	-0.37281100
C	-4.68264700	0.38006700	0.07351300
C	-3.55268100	-0.17921700	0.63978100
O	3.01241700	-2.35631700	-0.05876100
C	4.34290100	-2.52008300	0.41833400
O	-1.23052000	-1.85072600	1.95375900
C	-0.62490700	-3.01325500	2.49288400
H	-1.35909800	-4.94694200	-0.89430800
H	-3.22320200	-3.58353400	-1.76000400
H	-2.90911300	-1.18149400	-2.29725700
H	0.88451400	-3.94083200	-0.52826200
H	-0.97913400	0.82449800	-2.22567600
H	4.44676800	-0.02331200	-0.13559800

H	0.33917700	2.77004700	-2.20348000
H	3.11909400	3.60722200	-1.23350400
H	1.74268200	4.72107400	-1.71247100
H	2.38450900	4.72994300	0.69525400
H	0.66272400	4.32794800	0.45591200
H	2.92662500	2.60378900	1.61794800
H	1.39642300	-1.41695900	2.72598500
H	2.47183700	0.57177900	2.33484800
H	-0.99443900	3.49234900	0.44830700
H	-3.57796700	3.55108700	-0.61310500
H	-5.59443100	2.12584600	-0.80684100
H	-5.58464800	-0.21436500	-0.02655700
H	-3.53891200	-1.20753700	0.98000700
H	4.53170100	-1.88007700	1.28794300
H	4.42444900	-3.56564700	0.71133900
H	5.07097600	-2.30141700	-0.36884800
H	-0.34585400	-2.85623200	3.54179200
H	-1.37470600	-3.79978100	2.42642400
H	0.25536000	-3.29729500	1.90444300

INT$_{A}$-1:

G[WB97XD/6-311++G(2d,p) // WB97XD/6-31G(d), 403.15K] = -1450.756151 hartree.

E[WB97XD/6-311++G(2d,p) // WB97XD/6-31G(d), 403.15K] = -1451.161019 hartree.

C	2.08124900	3.44540700	-1.19222700
C	2.95880100	2.45899500	-1.66950000
C	2.51444100	1.18118700	-1.97626400
C	1.16289400	0.92196700	-1.77941000
C	0.26927400	1.89148700	-1.29130500
C	0.73461500	3.17637900	-1.00162000
N	0.45631200	-0.26374400	-1.98370600
C	-0.83813800	-0.09514600	-1.63432200
C	-1.00955500	1.22858000	-1.18836100
C	-1.89917400	-1.05516800	-1.52607900
N	-3.10803700	-0.63731900	-1.01859600
C	-3.27566600	0.60288700	-0.62816900
C	-2.24457200	1.58644900	-0.65760000
C	-1.73326600	-2.41029600	-1.72740800
C	-2.68580200	-3.35111800	-1.17081500
C	-2.47713600	-3.47250500	0.45806900
C	-2.52295000	-2.20301600	1.18771200
C	-1.46777700	-1.34281400	1.44414500
C	-0.06836800	-1.50744300	1.14451000
C	0.86653400	-0.49733000	1.33440700
C	0.47320000	0.75889500	1.88782800
C	-0.82850200	0.90208600	2.25694100
N	-1.73424700	-0.11328200	2.01150400
N	0.56905500	-2.59063600	0.57005400
C	1.91285500	-2.30051100	0.42537500
C	2.13708100	-0.98464600	0.87768500
C	2.93715800	-3.09038700	-0.10309200
C	4.19883600	-2.52417200	-0.16974200
C	4.44058100	-1.20889900	0.27272600
C	3.42378100	-0.43196000	0.79588200
O	-2.36704700	2.82018100	-0.18107000
C	-3.61226600	3.24398300	0.36966600
O	1.43728600	1.68786400	2.00833000
C	1.05407200	2.95799100	2.49897300
H	2.46533100	4.43712900	-0.97706800
H	4.00756200	2.70139800	-1.80625400
H	3.19437600	0.41554800	-2.33416200
H	0.05381300	3.94138600	-0.64549700
H	0.88436400	-1.13373600	-2.26083300
H	-4.26805200	0.86103800	-0.26990800

H	-0.79137100	-2.77955700	-2.12497900
H	-3.70972200	-2.99329300	-1.28581400
H	-2.57895700	-4.35955700	-1.57381100
H	-3.30841800	-4.11133100	0.76449600
H	-1.57062200	-4.05878000	0.61448300
H	-3.50696600	-1.86899900	1.50339500
H	-1.24243800	1.78804800	2.71556900
H	-2.68165800	0.03303000	2.32701500
H	0.19648700	-3.52313300	0.52785800
H	2.75689000	-4.10688100	-0.43934500
H	5.02126700	-3.10937200	-0.56860400
H	5.44510200	-0.80443300	0.20447800
H	3.60240500	0.58244100	1.13157500
H	-3.88582600	2.62850200	1.23334800
H	-3.45550100	4.27332700	0.68719200
H	-4.40435700	3.20830800	-0.38360900
H	0.71158000	2.88990500	3.53890900
H	1.94410300	3.58342800	2.44803700
H	0.26391600	3.39080800	1.87263500

TS$_A$-2:

G[WB97XD/6-311++G(2d,p) // WB97XD/6-31G(d), 403.15K] = -1450.749369 hartree.

E[WB97XD/6-311++G(2d,p) // WB97XD/6-31G(d), 403.15K] = -1451.156570 hartree.

C	1.69378600	3.64474200	-1.21485500
C	2.67208000	2.74994600	-1.68341200
C	2.36223400	1.43341900	-1.98300600
C	1.04201200	1.03359500	-1.78791100
C	0.04962600	1.90940000	-1.30420300
C	0.38311900	3.23983900	-1.02485700
N	0.46465600	-0.21527500	-1.97839000
C	-0.84327100	-0.17332300	-1.61110400
C	-1.14711500	1.11565800	-1.18043600
C	-1.77313000	-1.26229000	-1.44366300
N	-2.95142000	-0.99925900	-0.75582300
C	-3.27891300	0.26490100	-0.43613100
C	-2.42067300	1.34997200	-0.61784500
C	-1.50928900	-2.57932500	-1.69345200
C	-2.38513800	-3.57768600	-1.07150100
C	-2.33017700	-3.47881200	0.53704900
C	-2.43791100	-2.10032400	1.06815000
C	-1.34292600	-1.27918300	1.43515400
C	0.04312800	-1.44982700	1.14461200
C	0.97543600	-0.42344500	1.33353300
C	0.56766800	0.82336100	1.87498300
C	-0.74429100	0.95739000	2.24102700
N	-1.62688700	-0.06862600	2.00229000
N	0.69866300	-2.52592800	0.59236600
C	2.02881000	-2.20944300	0.40502200
C	2.24331600	-0.89149900	0.85706100
C	3.05023100	-2.97752600	-0.15950400
C	4.29743600	-2.38578800	-0.26207600
C	4.53002400	-1.06884400	0.18176100
C	3.51699800	-0.31412500	0.74201100
O	-2.69160100	2.61128900	-0.27604300
C	-3.96133800	2.91398600	0.28710400
O	1.51140100	1.76358000	1.99472300
C	1.11636700	3.04104600	2.46771000
H	1.97243400	4.67320300	-1.00879400
H	3.69009400	3.09972000	-1.82083300
H	3.11743800	0.74048100	-2.33900200
H	-0.37476600	3.93330700	-0.67694000
H	0.96886700	-1.03659800	-2.27307300
H	-4.27056300	0.39447600	-0.01385200

H	-0.58407500	-2.87769200	-2.17787300
H	-3.42937000	-3.36915900	-1.31809600
H	-2.14113800	-4.60135000	-1.36133900
H	-3.17328300	-4.07250100	0.89637500
H	-1.43275600	-3.99491600	0.88638800
H	-3.39489600	-1.82872800	1.50214400
H	-1.16977200	1.84269100	2.68927300
H	-2.58697100	0.08394800	2.27628500
H	0.28424000	-3.40726900	0.34658900
H	2.87862600	-3.99484600	-0.49712100
H	5.11721800	-2.95204400	-0.69239200
H	5.52401700	-0.64561600	0.08282700
H	3.68701900	0.70186700	1.07693000
H	-4.10802800	2.38168600	1.23459600
H	-3.95730200	3.98758900	0.46818800
H	-4.76725600	2.66018100	-0.40831700
H	0.76586100	2.98131900	3.50492100
H	2.00633300	3.66604600	2.41824500
H	0.33595600	3.46396900	1.82500300

INT$_A$-2:

G[WB97XD/6-311++G(2d,p) // WB97XD/6-31G(d), 403.15K] = -1450.778144 hartree.

E[WB97XD/6-311++G(2d,p) // WB97XD/6-31G(d), 403.15K] = -1451.188244 hartree.

C	0.87054200	3.92267200	-1.19342400
C	1.99224100	3.22851200	-1.68829900
C	1.92732400	1.88213100	-2.00024600
C	0.70690700	1.23652800	-1.79142000
C	-0.42619200	1.91055700	-1.28127000
C	-0.33777900	3.28035800	-0.99024700
N	0.37128900	-0.09145800	-1.97303000
C	-0.91901000	-0.28684800	-1.55613300
C	-1.44822700	0.91296500	-1.13143200
C	-1.57089800	-1.55569100	-1.29339900
N	-2.60312100	-1.47141400	-0.31305200
C	-3.29458600	-0.24997400	-0.12065100
C	-2.76221400	0.92803400	-0.53063000
C	-1.16495300	-2.79597600	-1.61263900
C	-1.84100900	-3.92285600	-0.88705800
C	-1.88478000	-3.64823900	0.64370400
C	-2.16333500	-2.15901300	0.93422600
C	-1.00008400	-1.32444500	1.46761800
C	0.35787900	-1.39353800	1.12841400
C	1.22811600	-0.29788100	1.33158600
C	0.75325000	0.87876000	1.93787900
C	-0.56356200	0.89144300	2.34991100
N	-1.35374600	-0.18933400	2.10102800
N	1.06637400	-2.38557800	0.49767100
C	2.34579500	-1.93740400	0.23037500
C	2.49166800	-0.63481100	0.74906900
C	3.37775900	-2.57960600	-0.45661500
C	4.56091500	-1.87669100	-0.61032700
C	4.72421600	-0.57445600	-0.09776100
C	3.70152800	0.05611000	0.58435200
O	-3.33608900	2.14168100	-0.42980500
C	-4.62701800	2.20627600	0.14597600
O	1.61120300	1.88999900	2.06229100
C	1.11258500	3.14787400	2.50326300
H	0.95709900	4.98366300	-0.98081200
H	2.92450900	3.76381800	-1.83739300
H	2.79176900	1.34651200	-2.37966100
H	-1.20678100	3.81657900	-0.62377800
H	1.00733500	-0.81148400	-2.27552100
H	-4.27857000	-0.35137700	0.32382300

H	-0.33174600	-2.96879400	-2.28633000
H	-2.87862400	-4.00298200	-1.23370600
H	-1.36161900	-4.88498700	-1.08386600
H	-2.67897500	-4.25160200	1.09054600
H	-0.96057900	-3.96678900	1.13696600
H	-2.99035700	-2.07541800	1.64712800
H	-1.04493200	1.72559600	2.83807900
H	-2.33967700	-0.07843300	2.30571500
H	0.65493800	-3.20117200	0.07545600
H	3.26278400	-3.58512300	-0.84818600
H	5.38623300	-2.34344200	-1.13838100
H	5.67029600	-0.06409200	-0.24216900
H	3.81450100	1.06029700	0.97438700
H	-4.61497600	1.85027700	1.18513900
H	-4.91817000	3.25558700	0.12415900
H	-5.34322300	1.61221900	-0.43323600
H	0.75814500	3.08566900	3.53830500
H	1.95603900	3.83361900	2.44940200
H	0.31560100	3.49413200	1.83679500

P_A (picrasidine G (**1**)):

G[WB97XD/6-311++G(2d,p) // WB97XD/6-31G(d), 403.15K] = -1450.832869 hartree.

E[WB97XD/6-311++G(2d,p) // WB97XD/6-31G(d), 403.15K] = -1451.239608 hartree.

C	-5.96844600	2.11587000	1.22907000
C	-6.62882700	1.71904500	0.04929500
C	-6.06344900	0.82260600	-0.84108900
C	-4.80099500	0.32345500	-0.51903400
C	-4.12410600	0.71087800	0.65788200
C	-4.71903500	1.62069900	1.54516800
N	-4.00989100	-0.57562500	-1.21535400
C	-2.84270600	-0.77562800	-0.52270500
C	-2.87772300	0.00672200	0.64741300
C	-1.76661400	-1.59616500	-0.85858300
N	-0.72134700	-1.58961800	-0.00484000
C	-0.72053000	-0.86784500	1.15651300
C	-1.77711500	-0.05529900	1.51690200
C	-1.79039800	-2.43788100	-2.10703300
C	-0.73752900	-3.53998500	-2.10948100
C	0.59119700	-2.94230500	-1.67476300
C	0.52073500	-2.42731500	-0.24043700
C	1.76174900	-1.66532200	0.15153300
C	2.11287800	-0.42307900	-0.39411300
C	3.31067600	0.20312900	-0.01016900
C	4.14012900	-0.45053000	0.90919700
C	3.70502600	-1.68257500	1.40772100
N	2.54978000	-2.25470800	1.04202000
N	1.45804200	0.39614700	-1.28940200
C	2.21035700	1.54699200	-1.49071500
C	3.38209900	1.46292600	-0.71206400
C	1.93977800	2.64765100	-2.30072700
C	2.87736300	3.66997800	-2.31491700
C	4.05118400	3.60221800	-1.54714200
C	4.31401300	2.50492800	-0.74313700
O	-1.82594900	0.68310000	2.62748000
C	-0.70317000	0.66833500	3.50334000
O	5.28124600	0.16991900	1.24683500
C	6.15706600	-0.47681900	2.15778600
H	-6.45362500	2.82048800	1.89554000
H	-7.61060100	2.12731900	-0.16769900
H	-6.58168900	0.52261300	-1.74569500
H	-4.20308900	1.91904300	2.45023900
H	-4.25404500	-0.99312300	-2.09873400
H	0.17018600	-0.97448900	1.75706600

H	-1.64197500	-1.77811400	-2.97366800
H	-1.03092200	-4.34323400	-1.42375000
H	-0.66365000	-3.97714400	-3.10808900
H	1.39429000	-3.68344700	-1.69962800
H	0.88809600	-2.13442700	-2.35571700
H	0.43639300	-3.27346000	0.44749700
H	4.29243500	-2.23764700	2.13076600
H	0.55370600	0.22800600	-1.69526700
H	1.03595700	2.70576700	-2.89932400
H	2.69890000	4.54179900	-2.93635100
H	4.76078500	4.42177800	-1.58780400
H	5.21832000	2.44448600	-0.14856400
H	0.19606700	1.02287800	2.98740000
H	-0.95401800	1.35044800	4.31349800
H	-0.53784100	-0.33639700	3.90731900
H	6.49111800	-1.44283500	1.76341000
H	7.01301000	0.18774500	2.26753200
H	5.67702800	-0.61920700	3.13257200
H	-2.79493900	-2.86448000	-2.20828800

TS_B-1:

G[WB97XD/6-311++G(2d,p) // WB97XD/6-31G(d), 403.15K] = -1450.756507 hartree.

E[WB97XD/6-311++G(2d,p) // WB97XD/6-31G(d), 403.15K] = -1451.155307 hartree.

C	6.25026000	1.65154500	-1.22555800
C	6.41466400	0.67815800	-2.22917600
C	5.56962400	-0.41363600	-2.33250700
C	4.53973100	-0.51007100	-1.39598800
C	4.35827400	0.45801500	-0.38490700
C	5.22898000	1.55312000	-0.29951400
N	3.56591900	-1.48446600	-1.26170600
C	2.75368600	-1.16886800	-0.20517800
C	3.21199200	0.03260400	0.36948800
C	1.61964600	-1.84331800	0.27506200
N	0.97630100	-1.28946600	1.30592300
C	1.39645500	-0.14911700	1.90275100
C	2.51455500	0.55211800	1.46949600
C	1.14695600	-3.10234200	-0.33744300
C	1.95181600	-4.14347300	-0.55345000
C	-1.55323500	-2.55907700	1.23638300
C	-0.57006200	-2.01601600	2.07573800
C	-2.58297700	-1.81082000	0.62632500
N	-3.71202900	-2.43979800	0.20540500
C	-4.82453600	-1.85265700	-0.36462800
C	-4.85833400	-0.50194600	-0.56008900
C	-3.69944100	0.23927900	-0.19831300
C	-2.60022800	-0.41536400	0.36026400
C	-3.33913600	1.61920300	-0.35701000
C	-2.00929800	1.73186000	0.10191300
N	-1.58927700	0.49898200	0.55555600
C	-4.00217500	2.75177700	-0.85509900
C	-3.31716500	3.95049200	-0.88858300
C	-1.98455100	4.04086000	-0.43801500
C	-1.31131900	2.94170200	0.06278000
O	2.96980000	1.69096600	2.00807700
C	2.28560600	2.23097700	3.12888500
O	-5.87654600	0.19363100	-1.09431100
C	-7.03255000	-0.52764000	-1.48371200
H	6.93824700	2.48897200	-1.18180000
H	7.22678600	0.78539700	-2.94109600
H	5.70436400	-1.16042200	-3.10795800
H	5.09718000	2.29798800	0.47668000
H	3.42184700	-2.26642000	-1.88088000
H	0.81378000	0.17226400	2.75916900

H	0.10146600	-3.14757500	-0.62261300
H	2.99297700	-4.14417200	-0.23880600
H	1.58251800	-5.04652900	-1.02850800
H	-1.57604600	-3.63721600	1.10656000
H	-0.03592200	-2.72635500	2.69990600
H	-0.80437500	-1.08815200	2.59444500
H	-3.76764200	-3.43262500	0.38756300
H	-5.62401400	-2.53177000	-0.62173400
H	-0.62618400	0.26058400	0.73339800
H	-5.02503500	2.67553700	-1.20470800
H	-3.80753200	4.83982700	-1.26984800
H	-1.47485900	4.99797800	-0.48564200
H	-0.28299300	3.01342900	0.40391600
H	1.24927900	2.48312100	2.87337300
H	2.30364600	1.53322800	3.97361300
H	2.82420000	3.13866300	3.39604900
H	-7.48668300	-1.03346100	-0.62304100
H	-7.72815000	0.21042000	-1.88012500
H	-6.79269500	-1.26044500	-2.26378900

INT$_B$-1:

G[WB97XD/6-311++G(2d,p) // WB97XD/6-31G(d), 403.15K] = -1450.760046 hartree.

E[WB97XD/6-311++G(2d,p) // WB97XD/6-31G(d), 403.15K] = -1451.161727 hartree.

C	6.18330100	1.84199600	-1.20517300
C	6.53246500	0.75806600	-2.03546800
C	5.80287300	-0.41760000	-2.05074200
C	4.69568800	-0.48637200	-1.20362400
C	4.32988300	0.59107900	-0.36645700
C	5.08791000	1.77209900	-0.36791500
N	3.80205100	-1.52468000	-1.01300900
C	2.86518000	-1.14804600	-0.08922900
C	3.16015800	0.16238800	0.33961900
C	1.76350700	-1.87086500	0.37751300
N	0.96776800	-1.24493700	1.26676500
C	1.23523700	0.00978500	1.73708900
C	2.31674700	0.75084900	1.29429000
C	1.48758400	-3.24338700	-0.08873600
C	2.38928500	-4.22035800	0.01777200
C	-1.26430400	-2.54024800	0.95500900
C	-0.26710100	-1.94473900	1.86868600
C	-2.35691300	-1.88041900	0.42478300
N	-3.45321900	-2.58429400	-0.01162000
C	-4.65558000	-2.05450500	-0.46147200
C	-4.82924800	-0.71048900	-0.53868500
C	-3.70700200	0.11102400	-0.20400400
C	-2.52686900	-0.46831500	0.22760600
C	-3.47432800	1.52749300	-0.31456500
C	-2.12427000	1.73210900	0.03527900
N	-1.57167100	0.51841700	0.41134400
C	-4.26505100	2.61981600	-0.69769800
C	-3.68118100	3.87288100	-0.73893200
C	-2.32464300	4.05491100	-0.41059600
C	-1.52751900	2.99286000	-0.01861900
O	2.60788200	1.99084800	1.69879300
C	1.78495700	2.59633300	2.68957300
O	-5.94395200	-0.06693800	-0.94597900
C	-7.05041300	-0.86520200	-1.31660800
H	6.78791000	2.74206300	-1.22800600
H	7.39964600	0.84758700	-2.68177000
H	6.08039100	-1.24665600	-2.69294300
H	4.81234000	2.60040600	0.27438400
H	3.79466300	-2.40034900	-1.51254200
H	0.53720100	0.38569500	2.47094600

H	0.51431700	-3.41927100	-0.53466800
H	3.35128400	-4.07118000	0.50261200
H	2.17656000	-5.21585100	-0.35790600
H	-1.29510200	-3.62575400	0.94207600
H	0.15789700	-2.71713500	2.51414300
H	-0.69520400	-1.17119800	2.51074200
H	-3.42510900	-3.58447200	0.11960900
H	-5.40457700	-2.78637500	-0.72714500
H	-0.58535200	0.33442500	0.32085000
H	-5.30628200	2.47317500	-0.96003300
H	-4.27363700	4.73215700	-1.03513300
H	-1.89458500	5.04997700	-0.46665500
H	-0.47731900	3.13429700	0.22144600
H	0.75270500	2.69921900	2.33502800
H	1.80818400	2.01944800	3.62060400
H	2.21001100	3.58342200	2.86186500
H	-7.38944400	-1.48216400	-0.47472300
H	-7.84212300	-0.17362800	-1.60210200
H	-6.80244400	-1.50953100	-2.16975000

TS_B-2:

G[WB97XD/6-311++G(2d,p) // WB97XD/6-31G(d), 403.15K] = -1450.743365 hartree.

E[WB97XD/6-311++G(2d,p) // WB97XD/6-31G(d), 403.15K] = -1451.146923 hartree.

C	-4.80024000	2.35129100	0.50234000
C	-4.18455700	2.90921600	1.64073300
C	-3.10167900	2.30482100	2.25359400
C	-2.63952400	1.11230500	1.69372700
C	-3.24541300	0.53769400	0.55507000
C	-4.34383600	1.16954600	-0.04738500
N	-1.58342800	0.31016400	2.08332900
C	-1.48117300	-0.74669900	1.21868600
C	-2.49415500	-0.64690100	0.26724100
C	-0.49816900	-1.76781900	1.21079400
N	-0.54082400	-2.62496100	0.14878900
C	-1.59962900	-2.60964900	-0.75381100
C	-2.58183400	-1.65513600	-0.72429600
C	0.53785200	-1.82682100	2.17739200
C	1.35423000	-2.91148000	2.26627500
C	2.04208300	-2.98143300	-0.00849100
C	0.67594900	-3.37101100	-0.43449200
C	2.63309500	-1.73304500	-0.21784200
N	3.95146300	-1.55894800	0.10978300
C	4.63219500	-0.36245200	0.20494700
C	3.98959000	0.81199900	-0.05028600
C	2.63071300	0.73507400	-0.46802800
C	2.00368200	-0.50817100	-0.60283800
C	1.68480900	1.74103100	-0.85408400
C	0.51686700	1.05190900	-1.24247200
N	0.73769200	-0.29859400	-1.10474000
C	1.70652100	3.14514800	-0.89185600
C	0.56586000	3.80732700	-1.29987800
C	-0.59486100	3.09921900	-1.67565100
C	-0.63929600	1.71828500	-1.65986700
O	-3.61839000	-1.57725000	-1.57035700
C	-3.73686000	-2.56480200	-2.58288300
O	4.52608200	2.04454200	0.03196000
C	5.88896000	2.13988200	0.40367500
H	-5.64859500	2.86003400	0.05709400
H	-4.57100500	3.83781900	2.04831500
H	-2.63216400	2.74132000	3.12893300
H	-4.81618100	0.73195700	-0.91939700
H	-1.00729800	0.46844000	2.89353900
H	-1.57822800	-3.40390900	-1.48730600

H	0.79423000	-0.90812100	2.69508500
H	1.00690900	-3.90155100	1.99546500
H	2.24293100	-2.88080200	2.88890900
H	2.70650000	-3.80999600	0.21444600
H	0.51400500	-4.43070400	-0.22462400
H	0.54561200	-3.23696200	-1.51137400
H	4.46248000	-2.38983000	0.37182200
H	5.67106800	-0.44500000	0.48952200
H	0.06583900	-0.99991000	-1.36910000
H	2.59983200	3.68518900	-0.60117600
H	0.55660500	4.89168400	-1.33234700
H	-1.47674800	3.65264700	-1.98209800
H	-1.53616100	1.17677700	-1.94307900
H	-2.87436700	-2.53701400	-3.25959900
H	-3.83744300	-3.56390700	-2.14377000
H	-4.64121900	-2.31731600	-3.13614400
H	6.53055800	1.62630500	-0.32284100
H	6.12659900	3.20266000	0.40996800
H	6.05328800	1.72226500	1.40493300

INT_B-2:

G[WB97XD/6-311++G(2d,p) // WB97XD/6-31G(d), 403.15K] = -1450.793822 hartree.

E[WB97XD/6-311++G(2d,p) // WB97XD/6-31G(d), 403.15K] = -1451.201277 hartree.

C	5.32888400	1.97250400	-1.00353700
C	5.05566100	1.87370400	-2.38156200
C	4.07255400	1.02852600	-2.86467200
C	3.35990900	0.27766900	-1.92742300
C	3.62059800	0.36138500	-0.54183600
C	4.62397300	1.22527400	-0.07859000
N	2.33438700	-0.62829900	-2.11891400
C	1.94528400	-1.12824200	-0.90492300
C	2.70450200	-0.54733000	0.08962600
C	0.85730900	-2.05361300	-0.66722900
N	0.64861200	-2.29690200	0.69657900
C	1.47890700	-1.79799800	1.72325500
C	2.47589400	-0.91922600	1.45984400
C	0.03961600	-2.54807700	-1.62597500
C	-1.14353400	-3.39743400	-1.27963800
C	-1.67844600	-2.99416400	0.11595700
C	-0.51959000	-3.05675700	1.11813900
C	-2.44689100	-1.68707700	0.04864500
N	-3.71370300	-1.80524400	-0.39999800
C	-4.60875000	-0.80237300	-0.62180800
C	-4.23515900	0.50420100	-0.38020500
C	-2.93035000	0.72656900	0.09525100
C	-2.05122500	-0.37310600	0.31288800
C	-2.21886700	1.91731900	0.43373800
C	-0.93798600	1.49566700	0.84960100
N	-0.87093200	0.12445600	0.78646900
C	-2.53254500	3.28835300	0.41463200
C	-1.55654200	4.18149900	0.80262700
C	-0.27662000	3.73801000	1.20381900
C	0.05685900	2.39778500	1.23588500
O	3.29780200	-0.35496000	2.36989100
C	3.11588700	-0.72010600	3.72311000
O	-5.01289300	1.57416100	-0.56007100
C	-6.33637600	1.37740500	-1.04257800
H	6.11115100	2.64489100	-0.66655800
H	5.63085600	2.47221300	-3.08079700
H	3.86606900	0.95263900	-3.92766800
H	4.83859400	1.29318500	0.98187700
H	1.97733700	-0.91615900	-3.01459500
H	1.25171200	-2.17588900	2.71005300

H	0.21060300	-2.28955900	-2.66310800
H	-0.90086700	-4.46885300	-1.24410200
H	-1.93029000	-3.28880300	-2.03521800
H	-2.39570100	-3.75213700	0.45653800
H	-0.25718400	-4.11436100	1.24399000
H	-0.84410600	-2.68098900	2.09428700
H	-4.03326200	-2.74425000	-0.60855600
H	-5.58017500	-1.10807300	-0.98156800
H	-0.04878400	-0.41899500	1.01181500
H	-3.51338300	3.62355400	0.09928500
H	-1.76757500	5.24532500	0.79629100
H	0.46754400	4.47383600	1.49118000
H	1.04449500	2.05736200	1.53007200
H	2.11140600	-0.44635300	4.07247100
H	3.27286100	-1.79669400	3.86385700
H	3.86245700	-0.16627200	4.29090500
H	-6.91974000	0.76904300	-0.34225800
H	-6.77561200	2.37067400	-1.11387600
H	-6.32395500	0.91078200	-2.03405400

P_B (isopicrasidine G):

G[WB97XD/6-311++G(2d,p) // WB97XD/6-31G(d), 403.15K] = -1450.824437 hartree.

E[WB97XD/6-311++G(2d,p) // WB97XD/6-31G(d), 403.15K] = -1451.232591 hartree.

C	5.77582300	1.87957700	-0.92045700
C	5.39623600	1.95330900	-2.27644900
C	4.29704900	1.27211800	-2.76751000
C	3.57217400	0.50222500	-1.85637800
C	3.93529900	0.41942000	-0.49358200
C	5.05737000	1.11895100	-0.02095000
N	2.44721500	-0.27538100	-2.06764200
C	2.06783100	-0.84355900	-0.88160000
C	2.96867700	-0.43818600	0.11962200
C	0.97645100	-1.67772100	-0.63522700
N	0.81042200	-2.07330500	0.63898300
C	1.66831200	-1.72125600	1.65280200
C	2.76214300	-0.90865800	1.43028400
C	0.01596000	-2.06183800	-1.72343200
C	-0.90341900	-3.21287700	-1.33373900
C	-1.49793300	-2.94348900	0.05356000
C	-0.36086200	-2.89699100	1.06427000
C	-2.46873600	-1.76952000	0.01632000
N	-3.67427400	-2.12142000	-0.42297500
C	-4.64047300	-1.21815600	-0.61640900
C	-4.46195900	0.14640600	-0.38522400
C	-3.20963300	0.55068300	0.08466700
C	-2.20902300	-0.41755900	0.29467000
C	-2.65644200	1.84074100	0.43038000
C	-1.33406400	1.60088400	0.84125100
N	-1.05376100	0.24243900	0.71409400
C	-3.15978700	3.14462500	0.43245500
C	-2.33093000	4.17134600	0.85860700
C	-1.01591100	3.91246000	1.27524100
C	-0.49490800	2.62591500	1.27034400
O	3.63263900	-0.52065200	2.36333600
C	3.45288600	-0.97682500	3.69939800
O	-5.38563200	1.10537900	-0.57339300
C	-6.67134800	0.71493100	-1.02829100
H	6.64718500	2.43117100	-0.58491700
H	5.98471400	2.56171300	-2.95550000
H	4.01422600	1.33420700	-3.81289100
H	5.34249800	1.05665800	1.02266600
H	1.94807000	-0.34310700	-2.94026200
H	1.42503200	-2.13228900	2.62216100

H	-0.57934900	-1.17075600	-1.96602300
H	-0.34389300	-4.15608700	-1.32341700
H	-1.70965200	-3.31180200	-2.06328600
H	-2.10950400	-3.80588000	0.33375500
H	0.02016300	-3.90760800	1.23735300
H	-0.70052700	-2.49111700	2.02055300
H	-5.58514300	-1.61260900	-0.97564800
H	-0.33381800	-0.18364600	1.27305600
H	-4.17729800	3.33685100	0.11250100
H	-2.70188900	5.19078500	0.87260700
H	-0.39093400	4.73634300	1.60534300
H	0.52660600	2.42991900	1.58344400
H	2.49741500	-0.62538000	4.10499700
H	3.50882700	-2.06985600	3.74794400
H	4.27176600	-0.54443300	4.27112200
H	-7.15104100	0.03337700	-0.31676800
H	-7.25238600	1.63357700	-1.10021900
H	-6.61382800	0.24044200	-2.01458300
H	0.59481500	-2.31635800	-2.61850000

TS$_{A'}$:

G[WB97XD/6-311++G(2d,p) // WB97XD/6-31G(d), 403.15K] = -1450.348298 hartree.

E[WB97XD/6-311++G(2d,p) // WB97XD/6-31G(d), 403.15K] = -1450.739861 hartree.

C	2.52363700	3.01652800	-1.32233000
C	3.29028400	1.90945700	-1.72350800
C	2.70255200	0.68374400	-1.99376100
C	1.32078400	0.58768200	-1.83832800
C	0.53611800	1.68351500	-1.42075700
C	1.14958600	2.91522200	-1.17287400
N	0.48892000	-0.50142400	-2.02339500
C	-0.80007000	-0.14972000	-1.70039500
C	-0.81582800	1.19169000	-1.32693500
C	-1.95733900	-0.97478300	-1.55480200
N	-3.09238000	-0.40624200	-1.07246700
C	-3.11976900	0.89559700	-0.74952000
C	-2.01201500	1.72744900	-0.81256800
C	-1.94413300	-2.37409900	-1.68269500
C	-2.99519400	-3.10844100	-1.08344800
C	-2.95601800	-2.95569500	0.73396200
C	-2.86426100	-1.61585500	1.18754600
C	-1.66955400	-0.87558300	1.48711800
C	-0.33410800	-1.31219200	1.25697400
C	0.75006000	-0.45337300	1.46600900
C	0.50591400	0.84607200	1.93132500
	-0.81684400	1.18882800	2.18642300
N	-1.85457500	0.37349500	1.95484800
N	0.15794800	-2.51467100	0.77314700
C	1.52373700	-2.41067900	0.59400700
C	1.94511500	-1.14216900	1.04358800
C	2.41646700	-3.33076100	0.04378200
C	3.75120000	-2.95573700	-0.03633200
C	4.18809500	-1.70281600	0.42198000
C	3.29387400	-0.79007700	0.96271100
O	-1.96440100	3.00628700	-0.38110100
C	-3.05362200	3.46025600	0.40144200
O	1.57470100	1.65621800	2.08324000
C	1.32093300	3.02016500	2.34932500
H	3.01746700	3.96444400	-1.13055800
H	4.36634500	2.01480900	-1.82508900
H	3.29688500	-0.17622700	-2.28576800
H	0.55025400	3.76790500	-0.87234400
H	0.81549600	-1.44130200	-2.17545400
H	-4.06721500	1.25594000	-0.36311300

H	-1.03051700	-2.87691900	-1.99236500
H	-3.98035000	-2.66853300	-1.22549500
H	-2.98405000	-4.18689200	-1.24424900
H	-3.91183300	-3.42445400	0.96527400
H	-2.14064200	-3.62177800	1.01284900
H	-3.77739700	-1.07736300	1.41133800
H	-1.07502900	2.17260000	2.56639900
H	-0.40836400	-3.19366800	0.29594900
H	2.08362300	-4.30550300	-0.30137000
H	4.47175700	-3.65028800	-0.45825700
H	5.23963800	-1.44442600	0.34459000
H	3.61765100	0.18951900	1.29478800
H	-3.24347400	2.76914900	1.23129200
H	-2.76205000	4.43922100	0.78418700
H	-3.96151500	3.56692100	-0.20455300
H	0.90546800	3.15862800	3.35553600
H	2.28510300	3.52455300	2.28079700
H	0.63335100	3.44018000	1.60595000

INT$_{A'}$:

G[WB97XD/6-311++G(2d,p) // WB97XD/6-31G(d), 403.15K] = -1450.408582 hartree.

E[WB97XD/6-311++G(2d,p) // WB97XD/6-31G(d), 403.15K] = -1450.800974 hartree.

C	-5.84537100	2.16658700	0.89388700
C	-6.51498800	1.63057900	-0.22175700
C	-5.96555700	0.59959400	-0.96549200
C	-4.71941900	0.11281100	-0.56591100
C	-4.02892100	0.64218900	0.54964900
C	-4.60975400	1.68282100	1.28693100
N	-3.94826700	-0.89451300	-1.10702200
C	-2.78498200	-1.00703900	-0.38328200
C	-2.79608100	-0.09065500	0.64314700
C	-1.71034500	-1.94978500	-0.63073900
N	-0.57398100	-1.67130700	0.14749500
C	-0.62764200	-0.85465200	1.29091500
C	-1.67942900	-0.04990500	1.54986000
C	-1.76852600	-2.95448700	-1.53206100
C	-0.59205300	-3.85027500	-1.78244800
C	0.69035900	-3.09920800	-1.43159600
C	0.63306700	-2.49474500	-0.02158600
C	1.88643700	-1.67840700	0.21615200
C	2.05342000	-0.38366200	-0.30222600
C	3.25911200	0.31068700	-0.12458100
C	4.29462000	-0.32957500	0.56773000
C	4.04593100	-1.61118600	1.05106700
N	2.87544700	-2.25349200	0.88131900
N	1.17947100	0.41459300	-1.00938300
C	1.78224600	1.63246500	-1.25678000
C	3.09657900	1.60744900	-0.73881000
C	1.27071900	2.75776300	-1.90376800
C	2.10571200	3.85765000	-2.02807800
C	3.41802600	3.84518800	-1.52701700
C	3.92170900	2.72655500	-0.88198700
O	-1.79149600	0.80904400	2.59853700
C	-0.66560300	0.92541500	3.43618800
O	5.44961200	0.35961500	0.71770400
C	6.50472200	-0.27143700	1.41282000
H	-6.30953100	2.97286600	1.45363700
H	-7.48353500	2.03180600	-0.50495900
H	-6.48511800	0.18502800	-1.82440600
H	-4.09016800	2.09138900	2.14658500
H	-4.15768900	-1.40185400	-1.94997200
H	0.25911000	-0.89244700	1.90684100

H	-2.67730000	-3.09879500	-2.10642300
H	-0.65565600	-4.77788800	-1.19389900
H	-0.55908900	-4.15738600	-2.83433600
H	1.56749800	-3.75180200	-1.47135400
H	0.84912300	-2.29167000	-2.15695000
H	0.62985200	-3.30910900	0.71486300
H	4.80057200	-2.16115200	1.60389800
H	0.19720000	0.20955900	-1.10946600
H	0.25720800	2.77143100	-2.29239300
H	1.73503300	4.74909900	-2.52540000
H	4.04232100	4.72510600	-1.64670000
H	4.93222300	2.70909300	-0.48890700
H	0.21918800	1.24367600	2.86795500
H	-0.91348300	1.68146900	4.18189900
H	-0.44495400	-0.02514900	3.94084300
H	6.81888200	-1.19363300	0.90863800
H	7.33101300	0.43998000	1.41509500
H	6.21975700	-0.50226700	2.44677400

$TS_{B'}$:

G[WB97XD/6-311++G(2d,p) // WB97XD/6-31G(d), 403.15K] = -1450.336450 hartree.

E[WB97XD/6-311++G(2d,p) // WB97XD/6-31G(d), 403.15K] = -1450.725952 hartree.

C	-4.28830400	3.00733200	0.17016800
C	-3.42461400	3.57688700	1.12278100
C	-2.45159900	2.82344400	1.75856100
C	-2.35674300	1.47511400	1.41333300
C	-3.21399600	0.88571100	0.45619500
C	-4.19354500	1.66753700	-0.16781100
N	-1.46864700	0.51501500	1.85396100
C	-1.71111100	-0.66363500	1.18727500
C	-2.78053800	-0.48158800	0.32114500
C	-0.97691600	-1.89095200	1.23279000
N	-1.31667100	-2.86250400	0.34930100
C	-2.41475800	-2.71245400	-0.46032900
C	-3.17000400	-1.56865700	-0.50871800
C	0.19733500	-2.07477100	1.98052500
C	0.95797600	-3.21644700	1.73900600
C	1.54647500	-3.12029600	-0.16479200
C	0.36945100	-3.23933100	-0.91366900
C	2.41974500	-1.91961600	-0.21788000
N	3.70128100	-2.13021600	0.06258300
C	4.58318100	-1.11708300	0.09282100
C	4.23259400	0.20781500	-0.13697600
C	2.88746700	0.47282600	-0.41879200
C	1.98422400	-0.60264100	-0.47226500
C	2.13830300	1.68217100	-0.66579800
C	0.80236600	1.27797000	-0.86841800
N	0.73456100	-0.09892000	-0.78879400
C	2.46510300	3.04140800	-0.70499400
C	1.45226400	3.96122600	-0.92830000
C	0.12453700	3.54092900	-1.11667700
C	-0.22094800	2.19886400	-1.09740100
O	-4.26979000	-1.36810600	-1.27376200
C	-4.64262600	-2.40572300	-2.15430400
O	5.07434600	1.26978500	-0.10088000
C	6.43380900	1.01448100	0.17902800
H	-5.03916100	3.63020400	-0.30597900
H	-3.52000700	4.63126600	1.36421400
H	-1.77717800	3.26870300	2.48316700
H	-4.85405600	1.22299300	-0.90397400
H	-0.69387800	0.68440800	2.47306800
H	-2.63722900	-3.57371900	-1.08175200

H	0.64430900	-1.22499100	2.48905000
H	0.41781700	-4.14612600	1.57968700
H	1.91616200	-3.31658500	2.24270100
H	2.13639600	-4.02619000	-0.04612500
H	0.00043100	-4.23107700	-1.14734600
H	0.06092300	-2.46425800	-1.60582300
H	5.60633000	-1.39938200	0.32088100
H	-0.13258200	-0.60317600	-0.70267900
H	3.49160500	3.35683900	-0.55396000
H	1.68457300	5.02144300	-0.95586700
H	-0.65382400	4.28078500	-1.27761600
H	-1.24890900	1.87992900	-1.23755100
H	-3.84219200	-2.62431700	-2.87278000
H	-4.89497400	-3.32125700	-1.60457700
H	-5.52346500	-2.04851200	-2.68844900
H	6.88013000	0.35548300	-0.57616800
H	6.93379600	1.98336200	0.15498100
H	6.56007300	0.56399600	1.17164300

INT$_{B'}$:

G[WB97XD/6-311++G(2d,p) // WB97XD/6-31G(d), 403.15K] = -1450.404793 hartree.

E[WB97XD/6-311++G(2d,p) // WB97XD/6-31G(d), 403.15K] = -1450.797719 hartree.

C	5.60699900	1.88696700	-1.00071800
C	5.32385700	1.78093700	-2.37503500
C	4.30218600	0.96993900	-2.83969200
C	3.56174200	0.26304900	-1.89060300
C	3.82860200	0.35973100	-0.50511700
C	4.87129900	1.18487000	-0.06227200
N	2.50591600	-0.60783600	-2.06309300
C	2.08310200	-1.04976300	-0.83506200
C	2.86861900	-0.48827500	0.14676800
C	0.97342300	-1.94925800	-0.58619400
N	0.70051300	-2.08628400	0.78464900
C	1.54860800	-1.60830400	1.79686800
C	2.60458800	-0.80904200	1.52536100
C	0.21351700	-2.52722200	-1.54021300
C	-0.97303800	-3.37694300	-1.19589200
C	-1.57162700	-2.95165900	0.16010300
C	-0.45445300	-2.87702000	1.20588300
C	-2.48555100	-1.74010300	0.01724100
N	-3.70686200	-2.04927900	-0.40620900
C	-4.63860700	-1.10792600	-0.61986100
C	-4.40972200	0.24846400	-0.41669500
C	-3.13814000	0.61926300	0.02771600
C	-2.17290600	-0.38582700	0.24463100
C	-2.52111300	1.88726300	0.33470700
C	-1.19948500	1.58861800	0.72733500
N	-1.01837900	0.22540900	0.68817600
C	-2.95095200	3.21759500	0.30458000
C	-2.05363500	4.21320800	0.65807600
C	-0.73713300	3.89770600	1.03671600
C	-0.28897500	2.58683700	1.07828300
O	3.45717400	-0.27016800	2.43576100
C	3.18423900	-0.53367300	3.79138600
O	-5.31008700	1.24203700	-0.61174900
C	-6.59621500	0.87796700	-1.06471400
H	6.41751700	2.53165900	-0.67481100
H	5.91878100	2.34572300	-3.08647600
H	4.08524900	0.88803200	-3.90057100
H	5.08729900	1.26340200	0.99741300
H	2.04924000	-0.80459900	-2.93764800
H	1.26109700	-1.90651300	2.79559300

H	0.44910000	-2.35072400	-2.58390900
H	-0.69834400	-4.44143200	-1.14367600
H	-1.75003400	-3.29609400	-1.96213400
H	-2.24165600	-3.75012800	0.49272100
H	-0.12782900	-3.90172200	1.42958900
H	-0.84027300	-2.44146800	2.13438600
H	-5.60100600	-1.47511200	-0.96264700
H	-0.14213900	-0.24964300	0.83591700
H	-3.96620600	3.45380600	0.00564300
H	-2.36735300	5.25236800	0.63908400
H	-0.05358000	4.69947600	1.30061600
H	0.73022500	2.34125100	1.36179200
H	2.19100000	-0.15958700	4.07567300
H	3.23856400	-1.60975900	4.00567900
H	3.94859600	-0.01074900	4.36673000
H	-7.10094500	0.21858100	-0.34752700
H	-7.15744600	1.80837800	-1.15761000
H	-6.55023500	0.38144600	-2.04209800

6. References

1. H. E. Gottlieb, V. Kotlyar and A. Nudelman, NMR Chemical Shifts of Common Laboratory Solvents as Trace Impurities, *J Org Chem*, 1997, **62**, 7512-7515.
2. M. J. Frisch, G. W. Trucks, H. B. Schlegel, G. E. Scuseria, M. A. Robb, J. R. Cheeseman, G. Scalmani, V. Barone, G. A. Petersson, H. Nakatsuji, X. Li, M. Caricato, A. V. Marenich, J. Bloino, B. G. Janesko, R. Gomperts, B. Mennucci, H. P. Hratchian, J. V. Ortiz, A. F. Izmaylov, J. L. Sonnenberg, Williams, F. Ding, F. Lipparini, F. Egidi, J. Goings, B. Peng, A. Petrone, T. Henderson, D. Ranasinghe, V. G. Zakrzewski, J. Gao, N. Rega, G. Zheng, W. Liang, M. Hada, M. Ehara, K. Toyota, R. Fukuda, J. Hasegawa, M. Ishida, T. Nakajima, Y. Honda, O. Kitao, H. Nakai, T. Vreven, K. Throssell, J. A. Montgomery Jr., J. E. Peralta, F. Ogliaro, M. J. Bearpark, J. J. Heyd, E. N. Brothers, K. N. Kudin, V. N. Staroverov, T. A. Keith, R. Kobayashi, J. Normand, K. Raghavachari, A. P. Rendell, J. C. Burant, S. S. Iyengar, J. Tomasi, M. Cossi, J. M. Millam, M. Klene, C. Adamo, R. Cammi, J. W. Ochterski, R. L. Martin, K. Morokuma, O. Farkas, J. B. Foresman and D. J. Fox, Gaussian 16 Rev. B.01.*Journal*, 2016.
3. G. Luchini, J. V. Alegre-Requena, I. Funes-Ardoiz and R. S. Paton, GoodVibes: automated thermochemistry for heterogeneous computational chemistry data, *F1000Research*, 2020, **9**.
4. P. Pracht, F. Bohle and S. Grimme, Automated exploration of the low-energy chemical space with fast quantum chemical methods, *Phys Chem Chem Phys*, 2020, **22**, 7169-7192.
5. W. H. Jiao, H. Gao, C. Y. Li, F. Zhao, R. W. Jiang, Y. Wang, G. X. Zhou and X. S. Yao, Quassidines A-D, Bis-beta-carboline Alkaloids from the Stems of Picrasma quassioides, *Journal of Natural Products*, 2010, **73**, 167-171.
6. J. Zhang, S.-s. Zhao, J. Xie, J. Yang, G.-d. Chen, D. Hu, W.-g. Zhang, C.-x. Wang, X.-s. Yao and H. Gao, N-methoxy-β-carboline alkaloids with inhibitory activities against Aβ42 aggregation and acetylcholinesterase from the stems of Picrasma quassioides, *Bioorganic Chemistry*, 2020, **101**, 104043.
7. K. Takasu, T. Shimogama, C. Saiin, H.-S. Kim, Y. Wataya, R. Brun and M. Ihara, Synthesis and evaluation of beta-carbolinium cations as new antimalarial agents based on pi-delocalized lipophilic cation (DLC) hypothesis, *Chem Pharm Bull*, 2005, **53**, 653-661.
8. H. Suzuki, Y. Tsukakoshi, T. Tachikawa, Y. Miura, M. Adachi and Y. Murakami, A new synthesis of 4-oxygenated β-carboline derivatives by Fischer indolization, *Tetrahedron Letters*, 2005, **46**, 3831-3834.
9. J. Kollmann, Y. Zhang, W. Schilling, T. Zhang, D. Riemer and S. Das, A simple ketone as an efficient metal-free catalyst for visible-light-mediated Diels–Alder and aza-Diels–Alder reactions, *Green Chemistry*, 2019, **21**, 1916-1920.
10. G. Wulff and H. T. Klinken, Preparation of novel heterocyclic amino acids from N-(arylmethylene)dehydroalanine methyl esters, *Tetrahedron*, 1992, **48**, 5985-5990.
11. M. Ihara, T. Taniguchi, K. Makita, M. Takano, M. Ohnishi, N. Taniguchi, K. Fukumoto and C. Kabuto, Synthesis of polycyclic cyclobutane derivatives by tandem intramolecular Michael-aldol reaction under two complementary conditions: TBDMSOTf-Et3N and TMSI-(TMS)2NH, *Journal of the American Chemical Society*, 2002, **115**, 8107-8115.
12. M. Ihara and M. Suzuki, Short Synthesis of (±)-Tacamonine by the Intramolecular Double Michael Reaction, *Heterocycles*, 2000, **52**.
13. K. Takasu, N. Nishida, A. Tomimura and M. Ihara, Convenient Synthesis of Substituted

Piperidinones from α,β-Unsaturated Amides: Formal Synthesis of Deplancheine, Tacamonine, and Paroxetine, *The Journal of Organic Chemistry*, 2005, **70**, 3957-3962.
14. T. Ohmoto and K. Koike, Studies on the constituents of Picrasma quassioides BENNET. II. On the alkaloidal constituents, *Chemical and Pharmaceutical Bulletin*, 1983, **31**, 3198-3204.
15. K. Koike, Y. Sakamoto and T. Ohmoto, A13C NMR study of β-carboline alkaloids, *Organic Magnetic Resonance*, 1984, **22**, 471-473.
16. T. Ohmoto, K. Koike, T. Higuchi and K. Ikeda, Studies on the alkaloids from Picrasma quassioides Bennet. IV. Structures of picrasidines I, J, and K, *Chemical and Pharmaceutical Bulletin*, 1985, **33**, 3356-3360.
17. K. Koike and T. Ohmoto, Studies on the alkaloids from Picrasma quassioides BENNET. IX. Structures of two .BETA.-carboline dimeric alkaloids, picrasidines-G and -S, *Chemical and Pharmaceutical Bulletin*, 1987, **35**, 3305-3308.
18. K. Koike, T. Ohmoto and T. Higuchi, Picrasidine-T, a dimeric β-carboline alkaloid from Picrasma quassioides, *Phytochemistry*, 1987, **26**, 3375-3377.
19. K. Koike and T. Ohmoto, Studies on the alkaloids from Picrasma quassioides Bennet. VII. Structures of .BETA.-carboline dimer alkaloids, picrasidines-H and -R, *Chemical and Pharmaceutical Bulletin*, 1986, **34**, 2090-2093.
